# Worldwide trends in diabetes since 1980: a pooled analysis of 751 population-based studies with 4·4 million participants

**DOI:** 10.1016/S0140-6736(16)00618-8

**Published:** 2016-04-09

**Authors:** NCD Risk Factor Collaboration (NCD-RisC)

## Abstract

**Background:**

One of the global targets for non-communicable diseases is to halt, by 2025, the rise in the age-standardised adult prevalence of diabetes at its 2010 levels. We aimed to estimate worldwide trends in diabetes, how likely it is for countries to achieve the global target, and how changes in prevalence, together with population growth and ageing, are affecting the number of adults with diabetes.

**Methods:**

We pooled data from population-based studies that had collected data on diabetes through measurement of its biomarkers. We used a Bayesian hierarchical model to estimate trends in diabetes prevalence—defined as fasting plasma glucose of 7·0 mmol/L or higher, or history of diagnosis with diabetes, or use of insulin or oral hypoglycaemic drugs—in 200 countries and territories in 21 regions, by sex and from 1980 to 2014. We also calculated the posterior probability of meeting the global diabetes target if post-2000 trends continue.

**Findings:**

We used data from 751 studies including 4 372 000 adults from 146 of the 200 countries we make estimates for. Global age-standardised diabetes prevalence increased from 4·3% (95% credible interval 2·4–7·0) in 1980 to 9·0% (7·2–11·1) in 2014 in men, and from 5·0% (2·9–7·9) to 7·9% (6·4–9·7) in women. The number of adults with diabetes in the world increased from 108 million in 1980 to 422 million in 2014 (28·5% due to the rise in prevalence, 39·7% due to population growth and ageing, and 31·8% due to interaction of these two factors). Age-standardised adult diabetes prevalence in 2014 was lowest in northwestern Europe, and highest in Polynesia and Micronesia, at nearly 25%, followed by Melanesia and the Middle East and north Africa. Between 1980 and 2014 there was little change in age-standardised diabetes prevalence in adult women in continental western Europe, although crude prevalence rose because of ageing of the population. By contrast, age-standardised adult prevalence rose by 15 percentage points in men and women in Polynesia and Micronesia. In 2014, American Samoa had the highest national prevalence of diabetes (>30% in both sexes), with age-standardised adult prevalence also higher than 25% in some other islands in Polynesia and Micronesia. If post-2000 trends continue, the probability of meeting the global target of halting the rise in the prevalence of diabetes by 2025 at the 2010 level worldwide is lower than 1% for men and is 1% for women. Only nine countries for men and 29 countries for women, mostly in western Europe, have a 50% or higher probability of meeting the global target.

**Interpretation:**

Since 1980, age-standardised diabetes prevalence in adults has increased, or at best remained unchanged, in every country. Together with population growth and ageing, this rise has led to a near quadrupling of the number of adults with diabetes worldwide. The burden of diabetes, both in terms of prevalence and number of adults affected, has increased faster in low-income and middle-income countries than in high-income countries.

**Funding:**

Wellcome Trust.

## Introduction

Diabetes is an important cause of mortality, morbidity, and health-system costs in the world.^1^, ^2^ Therefore, there is an urgent need to implement population-based interventions that prevent diabetes, enhance its early detection, and use lifestyle and pharmacological interventions to prevent or delay its progression to complications. To motivate such actions, one of the global targets set after the 2011 UN High-Level Meeting on Non-Communicable Diseases (NCDs) is to halt, by 2025, the rise in the age-standardised adult prevalence of diabetes at its 2010 levels.^3^ Valid and consistent estimates of diabetes prevalence over time are needed to evaluate the effect of interventions, compare trends in different countries, and measure progress towards the agreed target.

A previous study estimated trends in mean fasting plasma glucose from 1980 to 2008 and reported diabetes prevalence, but only as a secondary outcome and estimated based on mean fasting plasma glucose.^4^ The International Diabetes Federation (IDF) periodically reports diabetes prevalence,^5^, ^6^ but does not analyse trends; uses some sources that are based solely on self-reported diabetes; and does not fully account for differences in diabetes definitions in different data sources,^7^ even though diabetes prevalence varies depending on whether it is defined based on fasting plasma glucose, 2 h plasma glucose in an oral glucose tolerance test (2hOGTT), or haemoglobin A1c (HbA_1c_).^8^ Furthermore, it is not known how trends in prevalence, together with population growth and ageing, have affected the number of adults with diabetes. Our aim was to estimate worldwide trends in the prevalence and number of adults with diabetes. We also estimated the probability of achieving the global diabetes target.

**Research in context**

**Evidence before this study**

We searched MEDLINE (via PubMed) for articles published between Jan 1, 1950, and Dec 11, 2013, with the search terms (“Blood Glucose”[MAJR] OR “Diabetes Mellitus”[MAJR:NoExp] OR “Diabetes Mellitus, Type 2”[MAJR:NoExp] OR “Diabetes Mellitus, Type 1”[MAJR:NoExp] OR “Prediabetic state”[MAJR] OR “Hyperglycemia”[MAJR] OR “Hemoglobin A, Glycosylated”[MAJR]) AND (“Humans”[Mesh]). Articles were screened according to the inclusion and exclusion criteria described in the appendix (pp 2–6).

A few studies have reported diabetes trends in one or a few countries. A previous study reported diabetes prevalence trends to 2008 as a secondary outcome, which was estimated from mean fasting plasma glucose. This study was done before the global target on diabetes was agreed, hence there are no recent data. The International Diabetes Federation periodically reports diabetes prevalence but does not analyse trends, uses some sources that are only based on self-reported diabetes, and does not fully account for differences in diabetes definitions in different data sources.

**Added value of this study**

This study provides the lengthiest and most complete estimates of trends in adult diabetes prevalence worldwide. We achieved this level of detail by reanalysing and pooling hundreds of population-based sources with actual measurements of at least one diabetes biomarker and systematically converting all data sources to a common definition of diabetes. We also systematically projected recent trends into the future, and assessed the probability of achieving the global diabetes target.

**Implications of all the available evidence**

Since 1980, age-standardised diabetes prevalence in adults increased or at best remained unchanged in every country. The burden of diabetes, in terms of both prevalence and number of adults affected, has increased faster in low-income and middle-income countries than in high-income countries. If post-2000 trends continue, the probability of meeting the global diabetes target is lower than 1% for men and is 1% for women worldwide.

## Methods

### Study design and definitions

We estimated trends in diabetes prevalence from 1980 to 2014, in 200 countries and territories organised into 21 regions, mostly on the basis of geography and national income (appendix pp 11–12). The exception was a region consisting of high-income English-speaking countries because cardiometabolic risk factors, especially body-mass index (BMI), an important risk factor for diabetes, have similar trends in these countries, which can be distinct from other countries in their geographical region. As the primary outcome, diabetes was defined as fasting plasma glucose of 7·0 mmol/L or higher, history of diagnosis with diabetes, or use of insulin or oral hypoglycaemic drugs. This definition of diabetes is used in the Global Monitoring Framework for NCDs;^3^ it also relies more directly on data from population-based health-examination surveys, which, for logistical reasons, are more likely to measure fasting plasma glucose than 2hOGTT. Our analysis covered men and women aged 18 years or older, consistent with the Global Monitoring Framework for NCDs.^3^

Our study had three stages, each described in detail below and in the appendix (pp 2–8). First, we identified, accessed, and reanalysed population-based health-examination surveys that had measured at least one diabetes biomarker. We then converted diabetes prevalence in sources that had defined diabetes through 2hOGTT or HbA_1c_ or used a cutoff other than 7·0 mmol/L for fasting plasma glucose, to a corresponding prevalence based on the primary outcome as defined above. Finally, we applied a statistical model to the pooled data to estimate trends for all countries and years.

### Data sources

We included data sources that were representative of a national, subnational, or community population and that had measured at least one of the following diabetes biomarkers: fasting plasma glucose, 2hOGTT, and HbA_1c_. We did not use data from sources that relied entirely on self-reported history of diagnosis because this approach would miss undiagnosed diabetes, which forms a substantial share of all people with diabetes, especially in communities with little access to health care.^9^, ^10^, ^11^ Our methods for identifying and accessing data sources are described in the appendix (pp 2–6).

History of diabetes diagnosis was established with survey-specific questions, such as “have you ever been told by a doctor or other health professional that you have diabetes?” or the combination of “do you now have, or have you ever had diabetes?” and “were you told by a doctor that you had diabetes?” Similarly, the use of diabetes drugs was established with survey-specific questions, such as “are you currently taking medication for diabetes or high blood sugar?” or the combination of “do you currently inject insulin for diabetes?” and “are you currently taking any medicines, tablets, or pills for diabetes?” Some surveys also verified medications with use of visual inspection or medical records, or had used these approaches to establish the use of diabetes drugs.

### Conversion to a consistent definition of diabetes

9% of our data were from sources that had reported the prevalence of diabetes based on 2hOGTT or HbA_1c_ but not fasting plasma glucose. Another 29% of data were from a previous global pooling study^4^ or extracted from published reports and papers, and had used fasting plasma glucose but reported only mean plasma concentrations or a prevalence based on a cutoff other than 7·0 mmol/L (eg, ≥7·8 mmol/L). To correct for incomparability of definitions of diabetes, we used regressions that converted prevalence from these sources to our primary outcome. The dependent variable in each of these regressions, which are described in detail elsewhere^8^ and presented in the appendix (pp 7–8 and 30–79), was the primary outcome (prevalence of fasting plasma glucose ≥7·0 mmol/L or history of diabetes diagnosis or use of diabetes drugs), and the main independent variable was a prevalence based on the definitions in at least one study that did not report the primary outcome but had some form of data on diabetes and glycaemia. The coefficients of these regressions were estimated from data sources with individual-level data, which could be used to calculate prevalence with both definitions. Details of conversion (or cross-walking) regressions, and their specification and coefficients, are presented in the appendix (pp 30–79). Datapoints based on fewer than 25 people were excluded. All regressions included terms for age, sex, country, income (natural logarithm of per-capita gross domestic product adjusted for purchasing power and inflation), and the year of study. When we used more than 400 datapoints to estimate the regression coefficients, the regressions also included regional random effects. Finally, we included interaction terms in the regressions if the interaction terms provided a better fit to the data as determined by the Bayesian Information Criterion.

### Statistical analysis

The statistical model used to estimate diabetes prevalence by country, year, age, and sex is described in detail in a statistical paper and in related substantive papers.^12^, ^13^, ^14^ In summary, we used a hierarchical probit model in which diabetes levels and trends in countries were nested in regional levels and trends, which were in turn nested in those of super-regions and worldwide. In this structure, estimates of diabetes levels and trends for each country and year were informed by the country's own data, if available, and by data from other years in the same country and in other countries, especially those in the same region, with data for similar time periods. The hierarchical structure borrows information to a greater degree when data are non-existent or weakly informative (eg, because they have a small sample size or are not national), and to a lesser extent in data-rich countries and regions.

The model incorporated non-linear time trends and age patterns, allowing the age pattern of diabetes to vary across populations such that the rise in prevalence with age would be steeper where diabetes prevalence is higher.^15^ The model accounted for the fact that prevalence in subnational and community studies might systematically differ from nationally representative surveys, and also tends to have larger variation relative to the true values than national studies do. These features were implemented by including data-driven fixed-effect and random-effect terms for subnational and community data. The fixed effects adjust for systematic differences between subnational or community studies and national studies. The random effects allow national data to have larger influence on the estimates than subnational or community data with similar sample sizes. The model also accounted for rural–urban differences in diabetes prevalence, through the use of data-driven fixed effects for rural-only and urban-only studies. These rural and urban effects were weighted by the difference between study-level and country-level urbanisation. The statistical model also included country-level covariates that help predict diabetes prevalence, including average number of years of education, proportion of national population living in urban areas, a summary measure of availability of different food types for human consumption, and age-standardised adult mean BMI.^16^ The covariate on food availability was constructed from the food balance sheets of the Food and Agriculture Organization of the UN, with use of principal component analysis. Details of the variables, data, and methods are provided elsewhere.^13^, ^17^

We fitted this Bayesian model with the Markov chain Monte Carlo (MCMC) algorithm. Convergence was monitored and 5000 post-burn-in samples were obtained from the posterior distribution of model parameters, which were then used to obtain the posterior distributions of diabetes prevalence. The reported credible intervals (CrI) represent the 2·5–97·5 percentiles of the posterior distributions.

We report the posterior probability that an estimated increase or decrease represents a truly increasing or decreasing trend. Posterior probability would be 0·50 in a country or region in which an increase is statistically indistinguishable from a decrease; a larger posterior probability indicates more certainty in a change in prevalence. Additionally, we calculated the posterior probability of meeting the global target of no increase in diabetes prevalence if post-2000 trends continue. All analyses were done separately by sex. We used the WHO standard population for age standardisation.

We examined how well our model estimates diabetes prevalence in countries and years without data by withholding some of the data from the model and calculating the differences between the held-out data and the estimates—ie, the error in estimates (appendix pp 9–10). This validation test shows that our model performed very well; the median errors were close to zero globally, and the median absolute errors were small (appendix pp 80–84).

### Role of funding source

The funder of the study had no role in study design, data collection, analysis, interpretation, or writing of the report. Country and Regional Data Group members, BZ, JB, and MDC had full access to the data in the study and the corresponding author had final responsibility for the decision to submit for publication.

## Results

We used data from 751 population-based measurement surveys and studies, which included 4 372 000 participants aged 18 years or older. The studies covered 146 (73%) of the 200 countries and territories for which estimates were made (appendix pp 97–98). These 146 countries contained 90% of the world's adult population in 2014. Regionally, the average number of data sources per country ranged from less than one in central Africa to 24 in the high-income Asia Pacific region. 21 (39%) of the 54 countries without data were in sub-Saharan Africa, 11 (20%) in the Caribbean, seven (13%) in central Europe, four (7%) in central Asia, and the remaining 11 (20%) in other regions. Nearly a third of surveys and studies (242) were from years before 2000, and the other two-thirds (509) for 2000 and later.

**Figure 1:**
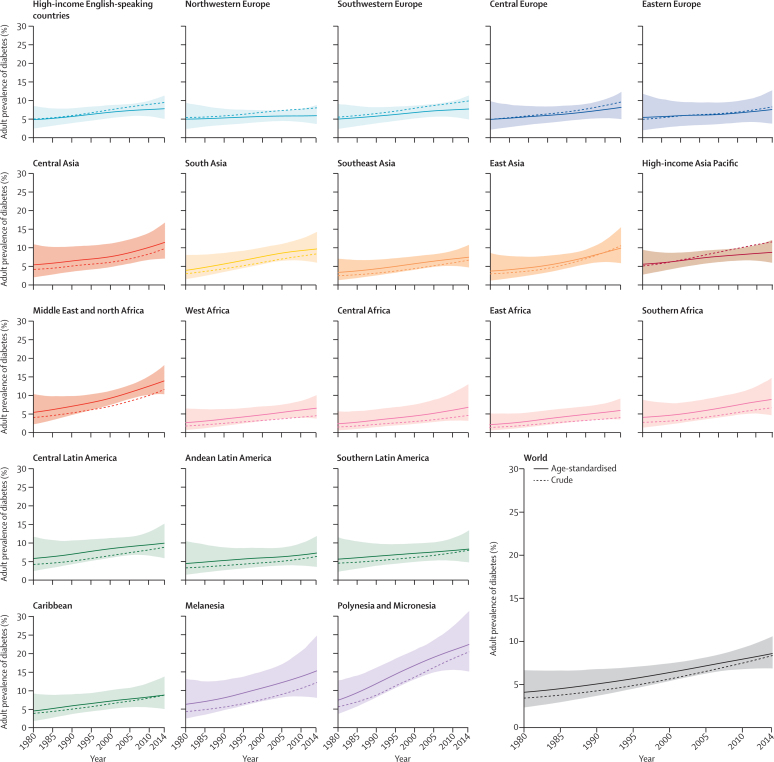
Trends in age-standardised and crude prevalence of diabetes for men by region The lines (solid for age-standardised and dashed for crude) show the posterior mean estimates; the shaded area shows the 95% credible intervals for age-standardised prevalence. For trends and numerical results by country see appendix pp 85–94, 103–170.

**Figure 2:**
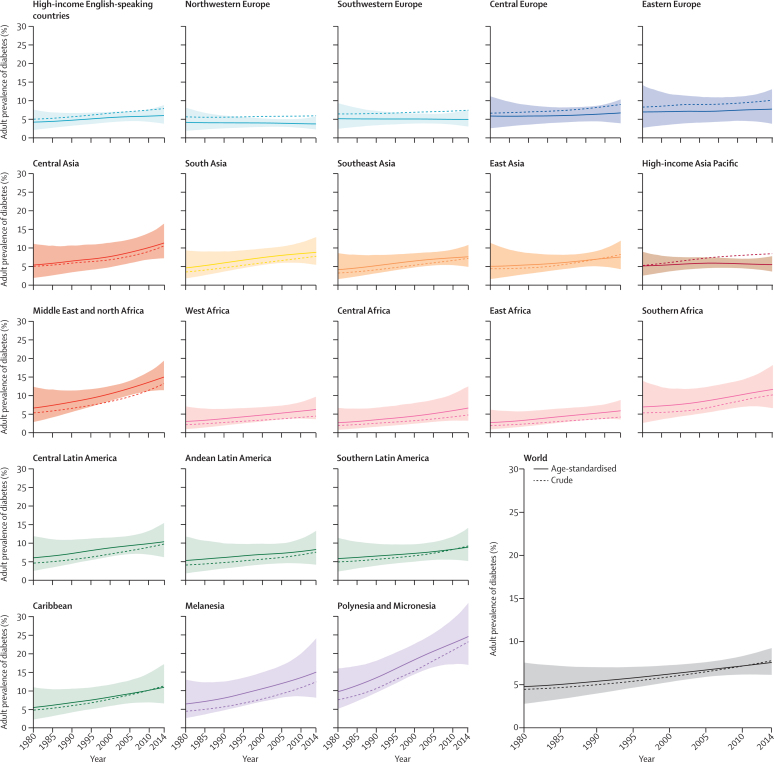
Trends in age-standardised and crude prevalence of diabetes for women by region The lines (solid for age-standardised and dashed for crude) show the posterior mean estimates; the shaded area shows the 95% credible intervals for age-standardised prevalence. For trends and numerical results by country see appendix pp 85–94, 103–170.

From 1980 to 2014, worldwide age-standardised adult diabetes prevalence increased from 4·3% (95% CrI 2·4–7·0) to 9·0% (7·2–11·1) in men (figure 1) and from 5·0% (2·9–7·9) to 7·9% (6·4–9·7) in women (figure 2); the posterior probabilities that these were true increases were 0·994 and 0·954, respectively. Over these years, crude adult prevalence increased from 3·6% (2·0–5·9) to 8·8% (7·0–10·8) in men, and from 4·7% (2·7–7·4) to 8·2% (6·6–9·9) in women (Figure 1, Figure 2).

Age-standardised diabetes prevalence in women in 2014 was lowest in northwestern and southwestern Europe, which each had a prevalence of less than 5% (figure 2). The lowest prevalence in adult men was also in northwestern Europe, at 5·8% (95% CrI 3·6–8·7). Crude adult prevalence in northwestern Europe was 5·9% (3·8–8·6) for women and 7·9% (5·1–11·5) for men in 2014. At the other extreme, age-standardised diabetes prevalence was higher than 20% in adult men and women in Polynesia and Micronesia, and around 15% in Melanesia and in the Middle East and north Africa.

Over the 35 years of analysis, there was almost no change in age-standardised diabetes prevalence in women in northwestern and southwestern Europe, and only a small non-significant increase in central and eastern Europe (figure 2). Adult men in northwestern Europe also had a smaller rise in prevalence than did other regions (figure 1). By contrast, age-standardised prevalence in Polynesia and Micronesia rose by 15·0 (95% CrI 5·5–25·9) percentage points in adult men (posterior probability >0·999) and by 14·9 (4·5–26·2) percentage points in adult women (posterior probability 0·998). Crude adult prevalence increased more than age-standardised prevalence in regions that had substantial ageing—eg, in high-income regions.

**Figure 3:**
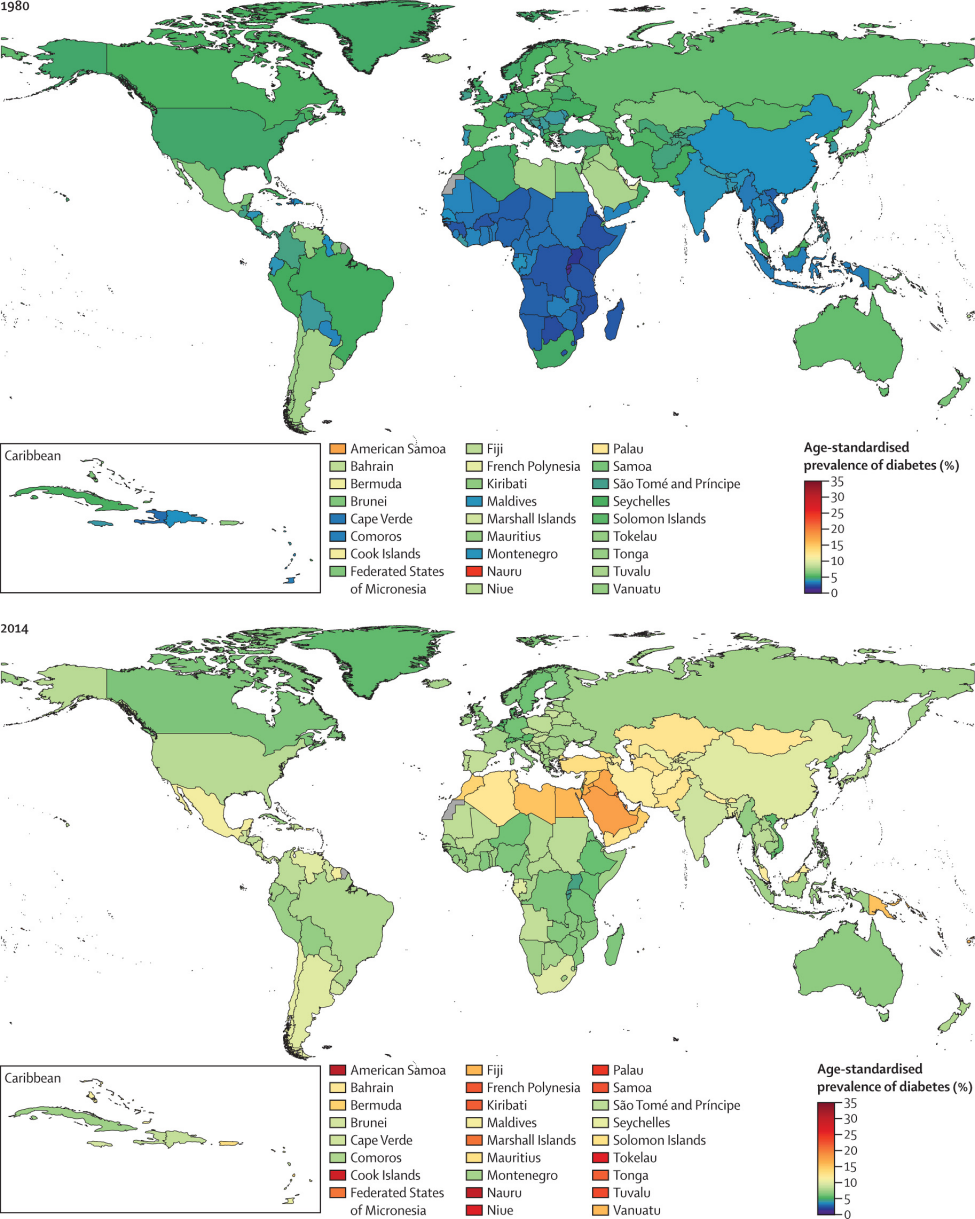
Age-standardised prevalence of diabetes in adult men by country in 1980 and 2014

**Figure 4:**
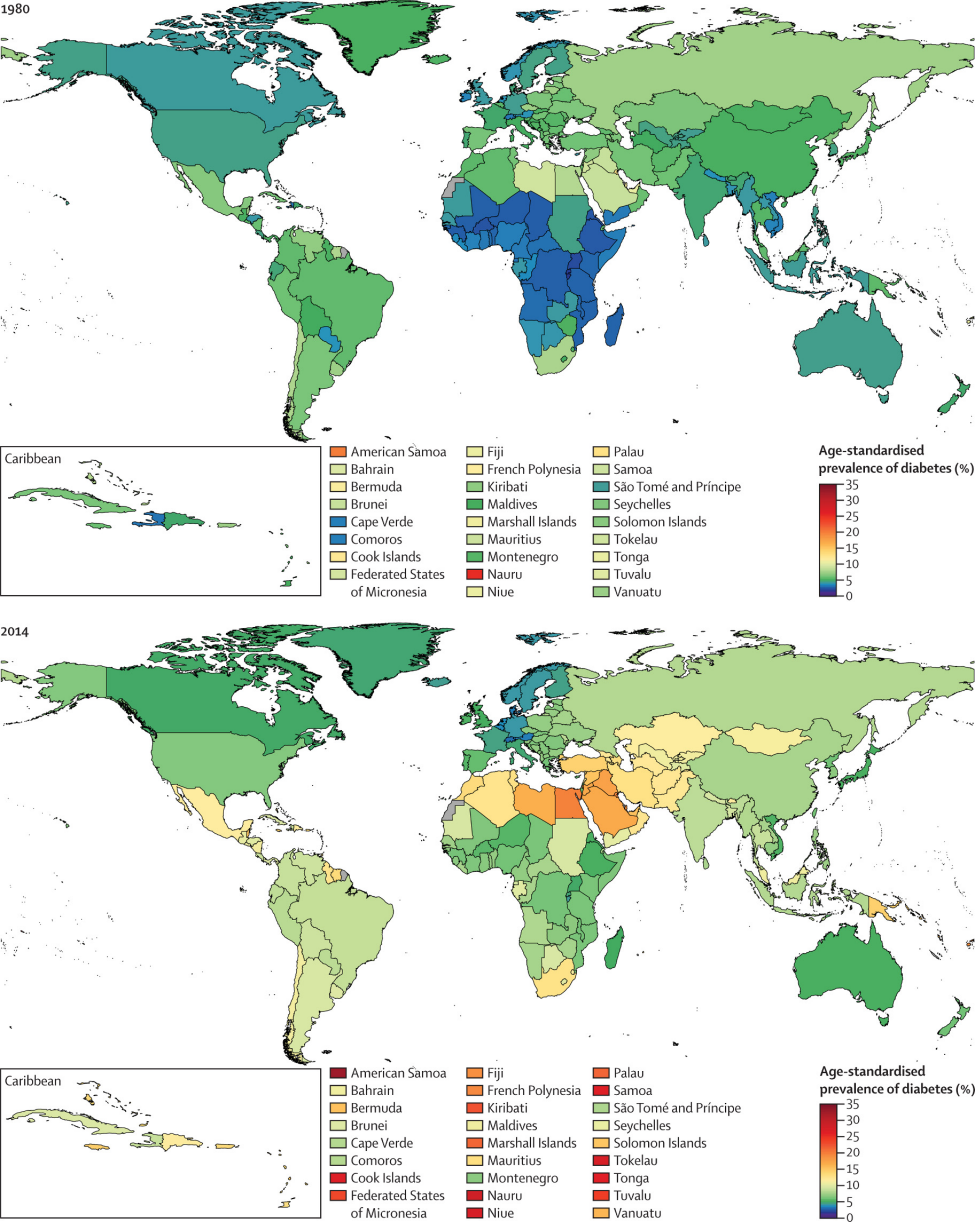
Age-standardised prevalence of diabetes in adult women by country in 1980 and 2014

In 1980, age-standardised adult diabetes prevalence was lower than 3% in men in 32 countries and in women in 23 countries (Figure 3, Figure 4). In the same year, age-standardised prevalence was higher than 12% in adult men and women in a few islands in Polynesia and Micronesia and women in Kuwait, reaching 25% in men and women in Nauru. By 2014, women in only one country had an age-standardised adult prevalence lower than 3% and women in only nine countries had one lower than 4%, with the lowest prevalence estimated in some countries in northwestern Europe such as Switzerland, Austria, Denmark, Belgium, and the Netherlands (figure 4). In the same year, age-standardised prevalence in adult men was higher than 4% in every country (figure 3); the lowest estimated prevalences were in the same northwestern European countries as those for women and in a few countries in east Africa and southeast Asia. At the other extreme, age-standardised adult diabetes prevalence in 2014 was 31% (95% CrI 19–44) in men and 33% (21–47) in women in American Samoa, and was also higher than 25% in men and women in some other islands in Polynesia and Micronesia (Figure 3, Figure 4; appendix pp 85–94).

**Figure 5:**
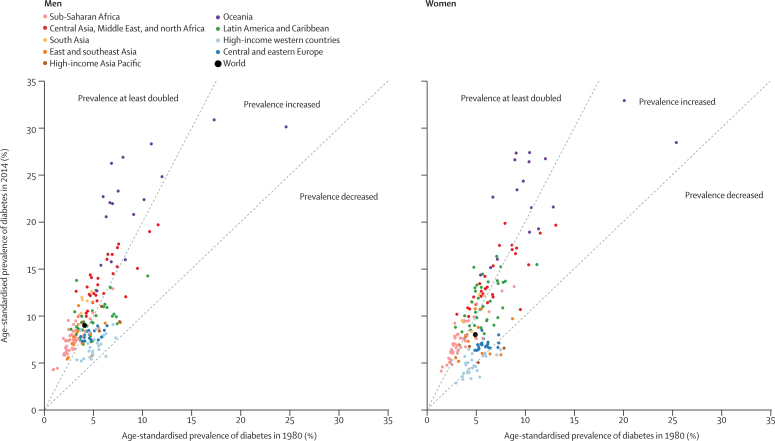
Comparison of age-standardised prevalence of diabetes in adults in 1980 and 2014

No country had a statistically significant decrease in diabetes prevalence from 1980 to 2014 (figure 5), although the relative increase over these 35 years was lower than 20% in nine countries for men, mostly in northwestern Europe, and in 39 countries for women. Over the same period, age-standardised adult prevalence of diabetes at least doubled for men in 120 countries and for women in 87 countries, with a posterior probability of 0·887 or higher. The largest absolute increases in age-standardised adult prevalence were in Oceania, exceeding 15 percentage points in some countries, followed by the Middle East and north Africa (figure 5).

**Figure 6:**
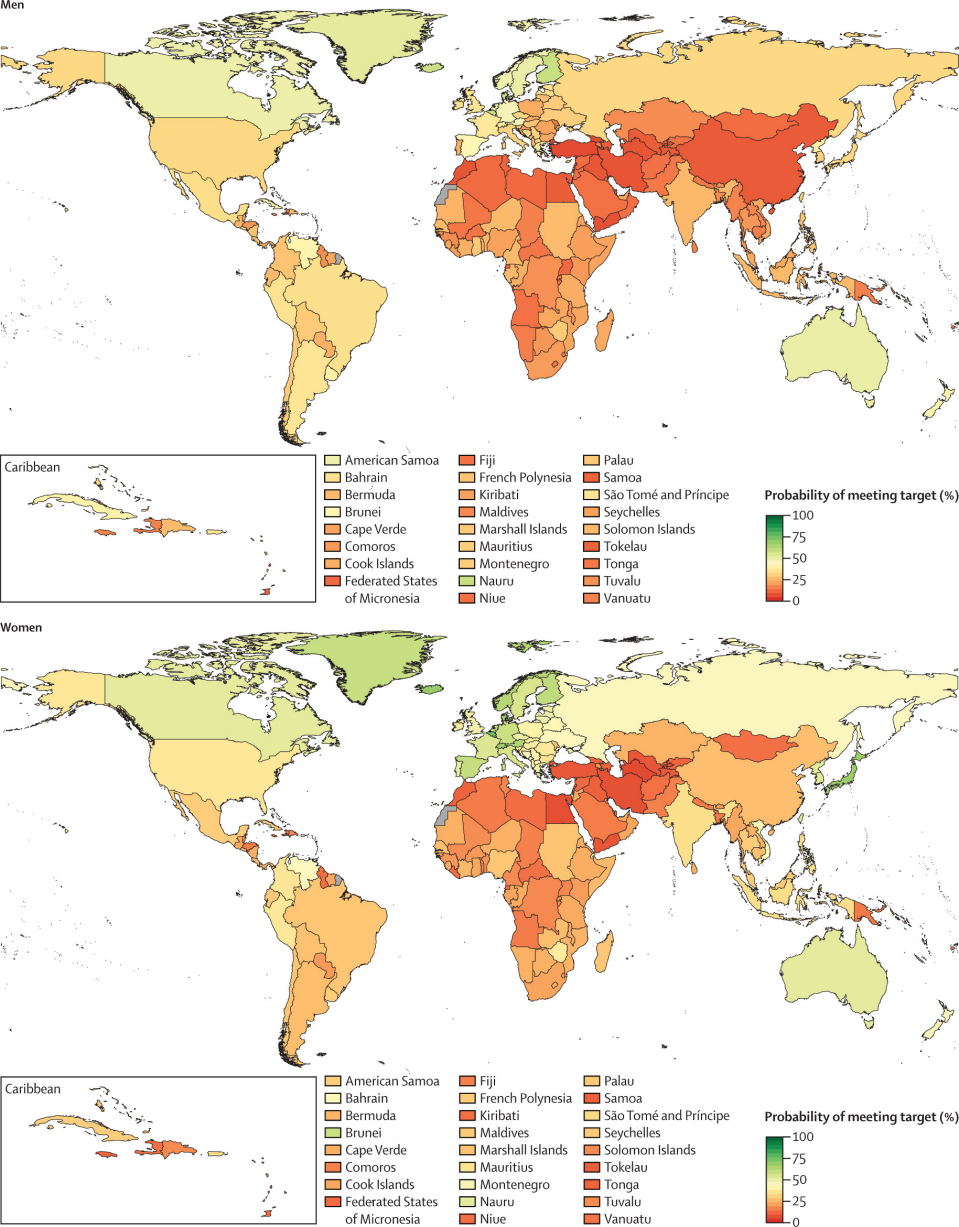
Probability of achieving the target of halting the rise of diabetes in adults by 2025 at its 2010 levels by sex and country if post-2000 trends continue

Worldwide, if post-2000 trends continue, the probability of meeting the global diabetes target for men is lower than 1%; for women it is 1%. Only nine countries, mostly in northwestern Europe, had a 50% or higher probability of meeting the global target for men, as did 29 countries for women (figure 6). Rather, if post-2000 trends continue, age-standardised prevalence of diabetes in 2025 will be 12·8% (95% CrI 8·3–19·6) in men and 10·4% (7·1–15·1) in women. The number of adults with diabetes will surpass 700 million.

**Figure 7:**
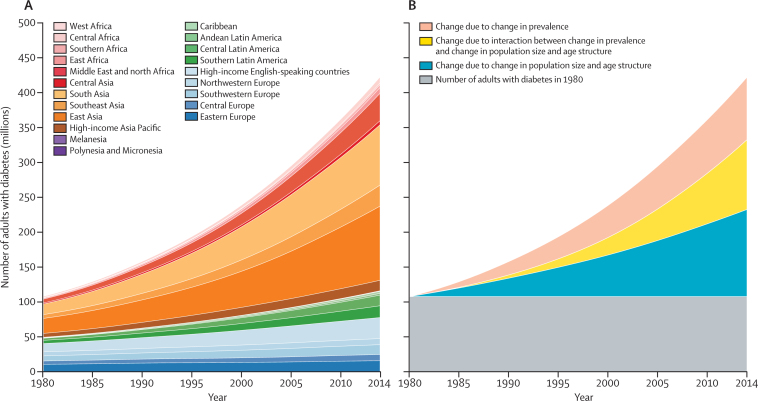
Trends in the number of adults with diabetes by region (A) and decomposed into the contributions of population growth and ageing, rise in prevalence, and interaction between the two (B) For results by region see appendix pp 101–102.

The number of adults with diabetes in the world increased from 108 million in 1980, to 422 million in 2014 (figure 7). East Asia and south Asia had the largest rises of absolute numbers, and had the largest number of people with diabetes in 2014: 106 million and 86 million, respectively. 39·7% (n=124·8 million) of the rise in the number of people with diabetes was due to population growth and ageing, 28·5% (n=89·7 million) due to the rise in age-specific prevalences, and the remaining 31·8% (n=99·9 million) due to the interaction of the two—ie, an older and larger population with higher age-specific prevalences (figure 7).

**Figure 8:**
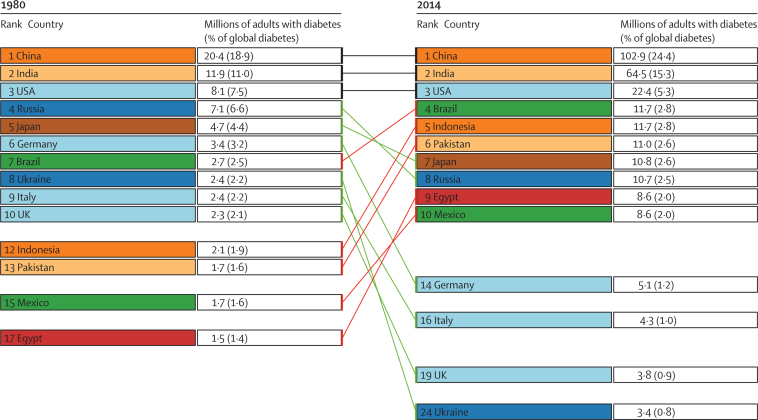
Ten countries with the largest number of adults with diabetes in 1980 and 2014 Colours for each country indicate its region.

Half of adults with diabetes in 2014 lived in five countries: China, India, the USA, Brazil, and Indonesia (figure 8). These countries also accounted for one half of the world's adult population in 2014. Although the top three countries on this list remained unchanged from 1980 to 2014, the global share of adults with diabetes who live in China and India increased, by contrast with the USA, where the share decreased. The changes in the share of adults with diabetes from India and the USA might be partly because of the changes in their shares. However, the share of the adult population of China remained virtually unchanged, while its share of the adult population with diabetes increased. Low-income and middle-income countries, including Indonesia, Pakistan, Mexico, and Egypt, replaced European countries, including Germany, Ukraine, Italy, and the UK, on the list of the top ten countries with most adults with diabetes (figure 8).

## Discussion

We used population-based data to document the global diabetes epidemic since 1980. Over this period, age-standardised diabetes prevalence in adults increased or at best remained unchanged in every country. It more than doubled in men and increased by 60% in women worldwide, shifting from an excess prevalence in women in 1980, to a higher male prevalence in 2014.^18^, ^19^ This rise in prevalence has been compounded by population growth and ageing, nearly quadrupling the number of adults with diabetes over these 35 years. The burden of diabetes, both in terms of prevalence and number of adults with diabetes, increased at a greater rate in low-income and middle-income countries than in high-income countries. The highest national prevalences—generally those in Oceania, and the Middle East and north Africa—are now five to ten times greater than the lowest prevalences, which are in some western European countries.

Our estimates of diabetes prevalence for 2013 for the world as a whole are similar to those by the IDF for the same year,^5^, ^6^ but there were differences between our estimates and those from the IDF in some countries and regions. In particular, we estimated a higher age-standardised prevalence of diabetes in most countries in the Middle East and north Africa than the IDF. The IDF does not estimate trends, hence we could not compare trend estimates. Our finding that diabetes prevalence was low in much of Asia and sub-Saharan Africa in the 1980s and 1990s is consistent with other studies that found low prevalences in these regions in those decades.^20^, ^21^ Our finding that diabetes prevalence did not increase in continental western Europe (especially in northwestern Europe) is also consistent with reports from Sweden,^22^ Germany,^23^ and Switzerland^24^ that covered a subset of our analysis years. Similarly, our estimated increases for high-income English-speaking countries are consistent with studies that had analysed repeated population-based surveys in the USA^25^ and the UK.^26^, ^27^ A meta-analysis of 15 studies in Japan reported no increase in diabetes prevalence,^28^ as observed in our analysis. Several recent reports have also documented similar increases in diabetes prevalence to those observed in our analysis in China,^29^, ^30^ India,^31^, ^32^, ^33^, ^34^ Iran,^35^ Turkey,^36^ and Saudi Arabia.^37^ Finally, our finding of the slower rise in diabetes prevalence in women compared with men is consistent with historical data in a few high-income countries.^18^, ^19^

The larger rise in diabetes prevalence in low-income and middle-income countries than in high-income countries, and the mostly flat trends in Europe (especially in northwestern Europe), might be caused by several factors. First, adiposity, which is an important risk factor for diabetes, has increased substantially more, and is now higher, in many low-income and middle-income countries than in continental Europe and high-income Asia Pacific countries, especially in women.^16^ Second, regional differences in diabetes could be due in part to differences in genetic susceptibility or phenotypic variations arising from inadequate fetal and childhood nutrition and growth; earlier onset of β-cell dysfunction might be one differentiating characteristic of Asian populations compared with European populations.^38^, ^39^, ^40^, ^41^, ^42^ Third, better-resourced health systems in Europe and other high-income countries might identify people at high risk of diabetes at an earlier stage, and use lifestyle and dietary modification or drugs to prevent or delay its onset.^43^, ^44^, ^45^ At the moment, information on the proportion of people with, or at risk of, diabetes who are diagnosed and receive treatment is limited to a few countries. Consistent information on diagnosis and treatment coverage will become increasingly important as universal health coverage becomes a central theme of global health efforts, and should be a focus of future analyses. Finally, in addition to total caloric intake and adiposity, dietary composition and physical activity might affect diabetes risk and contribute to differences in regional trends.^46^ These and other potential reasons for the divergent trends in diabetes prevalence should be investigated. Furthermore, the shift in diabetes burden from women towards men might be due to men having higher prevalences of some risk factors for diabetes, such as smoking, or being at risk of diabetes at lower BMI levels than are women.^18^, ^19^

The strengths of our study include its scope of making consistent and comparable estimates of trends in diabetes prevalence and of the probabilities of meeting the global diabetes target. We used an unprecedented amount of population-based data, which came from countries in which 90% of the global adult population lives. We used only data from studies that had measured a diabetes biomarker to avoid bias in self-reported data. Data were analysed according to a common protocol, and the characteristics and quality of data sources were rigorously verified through repeated checks by Collaborating Group members from each country. We pooled data using a statistical model that took into account the epidemiological features of diabetes, including non-linear time trends and age associations, and used all available data while giving more weight to national data than to subnational and community sources.

Despite our extensive efforts to identify and access worldwide population-based data, some countries had no or few data sources, especially those in sub-Saharan Africa, the Caribbean, central Asia, and central Europe. Estimates for these countries relied mostly or entirely on the statistical model, which shares information across countries and regions through its hierarchy and through predictive covariates. The absence or scarcity of data is reflected in wider uncertainty intervals of our estimates for these countries and regions (Figure 1, Figure 2; appendix pp 103–170). Diabetes was reported using a definition other than our primary outcome in some data sources, either because fasting plasma glucose was not measured or because individual-level data could not be accessed. To overcome this issue, we systematically used the reported metrics to estimate our primary outcome; the cross-walking regressions used for this purpose had good predictive accuracy. The share of studies that used a portable device (instead of laboratory analysis) for measuring diabetes biomarkers has increased over time. We do not expect the rise in the use of portable devices to affect the estimated levels and trends because their higher use in population-based research is partly due to increasing similarity between their measurements and those in laboratory-based tests,^47^, ^48^ facilitated by more advanced technologies and better standardisation. Further, although our primary outcome is consistent with the NCD Global Monitoring Framework, diabetes prevalence based on fasting plasma glucose alone is lower than that based on the combination of fasting plasma glucose and 2hOGTT.^8^ Age-standardised adult diabetes prevalence would be 10·0% (95% CrI 8·0–12·5) for men and 8·8% (7·2–10·7) for women, worldwide, if we applied the cross-walking regression (similar to those in appendix pp 34–79) to convert our estimates to prevalence of diabetes defined as fasting plasma glucose of 7·0 mmol/L or higher, or 2hOGTT of 11·1 mmol/L or higher, or history of diagnosis with diabetes or use of insulin or oral hypoglycaemic drugs. Finally, the survey data did not separate type 1 and type 2 diabetes because distinguishing between these disorders is difficult in adults.^49^, ^50^, ^51^ However, most (85–95%) cases of diabetes in adults are type 2,^50^, ^52^ so the observed rise in diabetes prevalence in adults is quite likely due to increases in type 2 diabetes.

Diabetes and its macrovascular and microvascular complications account for more than 2 million deaths every year,^1^ and are the seventh leading cause of disability worldwide.^53^ Diabetes is also a risk factor for tuberculosis, another disease with large burden in low-income and middle-income countries.^54^ Diabetes and its complications impose substantial economic costs on patients, their families, health systems, and national economies because of direct costs of treatment and loss of work and wages.^2^ On the basis of estimates for the number of people with diabetes in 2014 in this study, and cost estimates from a systematic review,^2^ the direct annual cost of diabetes in the world is Intl$825 billion, with China ($170 billion), the USA ($105 billion), India ($73 billion), and Japan ($37 billion) having the largest costs. Nearly 60% of the global costs are borne by low-income and middle-income countries, where substantial parts of treatment costs are paid out-of-pocket,^2^ which affects treatment utilisation and adherence and leads to financial hardship for patients and their families.

Glucose reduction with lifestyle modification and drugs in people with diabetes, especially if started early, can delay progression to microvascular complications.^55^, ^56^, ^57^ Although evidence is mixed from trials on the macrovascular benefits of intensive glucose lowering,^55^, ^58^, ^59^, ^60^ long-term glycaemic control and lowering blood pressure and serum cholesterol also reduce the risk of adverse cardiovascular outcomes.^61^, ^62^ However, the effectiveness of these interventions at the population level has been slight, both because many diabetes cases remain undiagnosed^9^, ^10^, ^11^ and because adherence to treatment is typically lower in general populations than in those enrolled in clinical trials.^63^, ^64^, ^65^

Efforts to reduce the global health and economic burden of diabetes should emphasise prevention of diabetes or delaying its onset, through enhancing healthy behaviours and diets at the population level, and early detection and management of high-risk individuals. There has been little success in preventing obesity,^16^ the most important risk factor for diabetes, at the population level although the global target on obesity could engender new efforts and policy innovations. As these policies are implemented, identifying people at high risk of diabetes—especially those with impaired glucose tolerance—through the primary care system, using advice and support to induce and maintain lifestyle change, possibly together with drugs such as metformin, might be the only short-term approach for global diabetes prevention.^44^, ^64^, ^66^, ^67^ Such programmes have been implemented in a few high-income and middle-income countries,^45^, ^68^, ^69^ but their success elsewhere requires a financially accessible primary care system that prioritises diabetes prevention and management and is staffed and resourced to support lifestyle change and improve access to and adherence to medication.^10^, ^43^, ^45^, ^64^, ^68^, ^70^

Correspondence to: Prof Majid Ezzati, School of Public Health, Imperial College London, London W2 1PG, UK majid.ezzati@imperial.ac.uk

**This online publication has been corrected. The corrected version first appeared at thelancet.com on October 26, 2016**
