# Department of Error

**DOI:** 10.1016/S0140-6736(16)32060-8

**Published:** 2017-02-04

*NCD Risk Factor Collaboration (NCD-RisC). Worldwide trends in diabetes since 1980: a pooled analysis of 751 population-based studies with 4·4 million participants.* Lancet *2016; **387**: 1513–30*—In this Article, Catherine Pelletier (Public Health Agency of Canada, Canada) has been added to the NCD Risk Factor Collaboration Pooled Analysis and Writing group (p 1527), with an asterisk to indicate equal contribution. This correction was made to the online version as of Oct 26, 2016.

