# Supplementary material for: Worldwide trends in diabetes since 1980: a pooled analysis of 751 population-based studies with 4·4 million participants
Source: Lancet. 2016 Apr 9;387(10027):1513–30. doi: 10.1016/S0140-6736(16)00618-8 (PMC5081106; doi:10.1016/S0140-6736(16)00618-8)
Supplement: Supplementary appendix [file mmc1.pdf]

THE LANCET

Supplementary appendix

This appendix formed part of the original submission and has been peer reviewed. We post it as supplied by the authors.

Supplement to: NCD Risk Factor Collaboration (NCD-RisC). Worldwide trends in diabetes since 1980: a pooled analysis of 751 population-based studies with 4·4 million participants. *Lancet* 2016; published online April 6. http://dx.doi.org/10.1016/S0140-6736(16)00618-8.

Appendix

Worldwide trends in diabetes since 1980: a pooled analysis of 751 population-based studies with 4.4 million participants

NCD Risk Factor Collaboration (NCD-RisC)

Table of Contents

Appendix 1: Data sources 2

Appendix 2: Converting among various diabetes definitions 7

Appendix 3: Validation of statistical model 9

Appendix Table 1: List of analysis regions and "super-regions", and countries in each region. 11

Appendix Table 2: Data sources used in the analysis. 13

Appendix Table 3: Availability of diabetes and glycaemia metrics in sources that did not report the primary outcome (prevalence defined by FPG ≥7.0 mmol/L or history of diabetes diagnosis or use of diabetes medication), and choices of predictors for use in conversion regressions described in Appendix 2. 30

Appendix Table 4: Model specifications and regression coefficients to estimate the primary outcome (prevalence defined by FPG ≥7.0 mmol/L or history of diabetes diagnosis or use of diabetes medication) from other metrics 34

Appendix Table 5: Results of model validation 80

Appendix Table 6: Age-standardised diabetes prevalence by sex and country in people aged 18 years and older in 1980, 1990, 2000, 2010 and 2014. 85

Appendix Figure 1: Flowchart of secondary search for data sources. 95

Appendix Figure 2: Number of data sources by country. 97

Appendix Figure 3: Number of data sources by region and year. 99

Appendix Figure 4: Regional trends in the number of adults with diabetes, decomposed into the contributions of population growth and ageing, rise in prevalence, and interaction of the two 101

Appendix Figure 5: Trends in age-standardised and crude prevalence of diabetes by sex and country in people aged 18 years and older 103

References 171

Appendix 1: Data sources

Data inclusion and exclusion

Data sources were included in NCD-RisC database if:

- measured data on fasting glucose, 2-h plasma glucose in an oral glucose tolerance test (2hOGTT) or haemoglobin A1c (HbA1c) were available;
- study participants were ten years of age and older;
- data were collected using a probabilistic sampling method with a defined sampling frame;
- data were representative of the general population at the national, subnational, or community level;
- data were collected in or after 1950;
- data were from the countries and territories listed in Appendix Table 1.

We excluded all data sources that included only diabetes diagnosis history or medication status without measurement of at least one of the above biomarkers. We excluded all 2hOGTT data if the data sources had not used a standard glucose load. We excluded all HbA1c data from before 2000 to minimise the use of non-standard assays. We also excluded data sources on population subgroups whose glycaemic status may differ systematically from the general population, including:

- studies that had included or excluded people based on their health status or cardiovascular risk;
- ethnic minorities;
- specific educational, occupational, or socioeconomic subgroups of the population; and
- those recruited through health facilities, with the exception noted below.

If a study had measured one biomarker only among participants with a high level of another, we excluded the dependent biomarker from it. For example, we excluded fasting plasma

glucose (FPG) in studies in which FPG was only measured in participants with HbA1c above a pre-specified value.

We used school-based data in countries where secondary school enrolment was 70% or higher, and used data whose sampling frame was health insurance schemes in countries where at least 80% of the population were insured. In addition, we used data collected through general practice and primary care system in high-income countries with universal insurance, because contact with the primary care systems tends to be at least as good as response rates for population-based surveys.

In this paper, we used data from the NCD-RisC database for years 1980 through 2014, and among subjects aged 18 years and older.

Primary source for data access

We used multiple routes for identifying and accessing data. We accessed publicly available population-based multi-country and national measurement surveys (e.g., Demographic and Health Surveys, and surveys identified via the Inter-University Consortium for Political and Social Research and European Health Interview & Health Examination Surveys Database) as well as the World Health Organization (WHO) STEPwise approach to Surveillance (STEPS) surveys. We requested, via WHO and its regional offices, from ministries of health and other national health agencies to identify and access population-based surveys. Requests were also sent via the World Heart Federation to its national partners. We made a similar request to the NCD Risk Factor Collaboration (NCD-RisC), a worldwide network of health researchers and practitioners working on NCD risk factors.

Anonymised individual record data from sources included in NCD-RisC were re-analysed by the Pooled Analysis and Writing Group or by data holders according to a common protocol. Within each survey, we included participants aged 18 years and older who were not pregnant and had fasted at least for 6 hours prior to measurement as a part of the survey instructions. We dropped participants with implausible FPG (defined as FPG <2.5 mmol/L or FPG >30 mmol/L; <0.5% of all FPG data), 2hOGTT (2hOGTT <2 mmol/L or 2hOGTT >30 mmol/L; <0.2% of all 2hOGTT data), or HbA1c (HbA1c <3% or HbA1c >18%; <0.1% of all HbA1c data).

In data sources that had measured FPG and had information on self-reported diagnosis and/or medication history, prevalence of diabetes as defined in Methods, and associated standard errors and sample sizes, were calculated by sex and age group (18-19 years, 20-29 years, followed by 10-year age groups and 80+ years). If only 2hOGTT or HbA1c had been measured, we estimated prevalence using the available biomarkers, and converted to the primary outcome as described in Appendix 2. All analyses incorporated appropriate sample weights and complex survey design when applicable. To ensure summaries were prepared according to the study protocol, the Pooled Analysis and Writing Group provided computer code to NCD-RisC members who requested assistance. All submitted data were checked by at least two independent members of the Pooled Analysis and Writing Group. Questions and clarifications were discussed with the Collaborating Group members and resolved before data were incorporated in the database.

Finally, we obtained data not accessed through the above routes by extracting from published reports of all additional national health surveys identified through the above-described strategies and one subnational STEPS survey. We restricted the extractions to those reports

that had reported data by sex and in age groups no wider than 20 years. We also used data from a previous pooling study which did not overlap with those accessed through the above routes.[1] Similarly, if a source did not report prevalence of diabetes as defined in Methods, we converted the metrics reported in the source to the primary outcome.

Secondary search for additional data sources

To identify any major sources not accessed through the above routes, we searched Medline (via PubMed) for articles published between 1st January 1950 and 11th December 2013 using the search terms ("Blood Glucose"[MAJR] OR "Diabetes Mellitus"[MAJR:NoExp] OR "Diabetes Mellitus, Type 2"[MAJR:NoExp] OR "Diabetes Mellitus, Type 1"[MAJR:NoExp] OR "Prediabetic state"[MAJR] OR "Hyperglycemia"[MAJR] OR "Hemoglobin A, Glycosylated"[MAJR]) AND ("Humans"[Mesh]) (search for adults for articles published between 1980 and 2009 was done in a previous analysis;[1] those for children for all years and for adults for years prior to 1980 and after 2009 were done here). We excluded USA from the search because multiple nationally representative health examination surveys with individual records were publicly accessible and accessed before the search had begun. Articles identified through this were screened according to the inclusion and exclusion criteria described above. The number of articles identified and retained is summarised in Appendix Figure 1. We contacted the corresponding authors of all eligible studies and invited them to join NCD-RisC. We did similar searches for other cardio-metabolic risk factors including body mass index (BMI), blood pressure, and serum cholesterol. All eligible studies were invited to join NCD-RisC and were requested to analyse data on all cardio-metabolic risk factors. We extracted data from those articles whose authors could not be contacted or did not respond to the invitation. As described above, we restricted the extractions to those articles that had reported data by sex and in age groups no wider than 20 years.

Data management

For each data source accessed through the primary and secondary data access process, we recorded information about the study population, period of measurement, sampling approach, and measurement methods. This information was used to establish that each data source was population-based, and to assess whether it covered the whole country, multiple subnational regions, or one or a small number of communities, and whether it was rural, urban, or combined.

We carefully checked all data sources for their study quality indicators in the inclusion and exclusion criteria. We identified duplicate data sources by comparing studies from the same country and year. Additionally, NCD-RisC members received the list of all data sources in the database and were asked to ensure that the included data from their country met the inclusion criteria and that there were no duplicates. Data sources used in the analysis are listed in Appendix Table 2.

Appendix 2: Converting among various diabetes definitions

As described in Methods, our primary outcome was prevalence of diabetes defined as FPG ≥7.0 mmol/L or history of diabetes diagnosis or use of diabetes medication, which was available in 62% of all data used. 9% of our data reported prevalence of diabetes based on 2hOGTT or HbA1c, mean 2hOGTT, and/or mean HbA1c. Another 29% of data, from a previous global pooling[1] or extracted from published studies, were based on FPG but reported only mean FPG or an FPG-based diabetes prevalence using an definition that differed from our primary outcome, e.g. diabetes defined as FPG ≥7.8 mmol/L. In order to use these data, we developed conversion regressions to estimate the primary outcome from the data available. The dependent variable in each of these regressions was the primary outcome (prevalence of FPG ≥7.0 mmol/L or history of diabetes diagnosis or use of diabetes medication), and the main independent variable was the diabetes metric(s) available in at least one study which did not report the primary outcome but had some form of data on diabetes and glycaemia.

The coefficients of these regressions were estimated from data sources with individual-level data which could be used to calculate prevalence using both definitions, excluding data points for which there were fewer than 25 subjects. All regressions included terms for age, sex, country's income (natural logarithm of per-capita gross domestic product in 2011 international dollars), and the year of study. When more than 400 data points were used to estimate the regression coefficients, the regressions also included regional random effects. Finally, we included interaction terms in the regressions if the interaction terms provided a better fit to the data as determined by the Bayesian Information Criterion (BIC).[2] In all cases, all sources of uncertainty in the conversion – including the sampling uncertainty of the original data, the uncertainty of the regression coefficients and random effects, and residuals

– were carried forward by using repeated draws from their respective distributions while accounting for the correlation among the uncertainties of regression coefficients and random effects. An overview of conversion regressions, in terms of the available metrics in extracted studies, is shown in Appendix Table 3. The regression coefficients and number of data points used to estimate the coefficients are shown in Appendix Table 4.

Appendix 3: Validation of statistical model

We tested how well our statistical model, which is described in the main text, predicts diabetes prevalence in countries and years without data in two different tests. In Test 1, we held out all data from 10% of countries with data (i.e., created the appearance of countries with no data where we actually had data). The countries whose data were withheld were randomly selected from the following three groups: data-rich (5 or more data sources, with at least one year of data after 2000), data-poor (1 data source), and average data availability (2 to 4 data sources, or 5 or more data sources with no data after 2000). We fitted the model to the data from the remaining 90% of countries and made estimates of the held-out observations. In Test 2, we assessed other patterns of missing data by holding out 10% of our data sources, again from a mix of data-rich, data-poor, and average-data countries, as defined above. For a given country, we either held out a random one third of the country's data or all of the country's 2000-2014 data to determine, respectively, how well we filled in the gaps for countries with intermittent data and how well we estimated in countries without recent data. We fitted the model to the remaining 90% of the dataset and made estimates of the held-out observations. We repeated each test five times, holding out a different subset of data in each repetition. In both tests, we calculated the differences between the held-out data and the estimates. We also checked the 95% credible intervals of the estimates; in a model with good external predictive validity, 95% of held-out values would be included in the 95% credible intervals.

Our statistical model performed well in the validation tests. The estimates of diabetes prevalence were unbiased as evidenced with median errors that were very close to zero globally (ranging from -0.3 percentage points to 0.1 percentage points for the two sexes in the two tests), and less than or equal to 2 percentage points in every subset of withheld data

except the 3.0 percentage points difference in Test 2 for men in Oceania and -3.3 percentage points difference in Test 1 for women in East and Southeast Asia (Appendix Table 5). The 95% credible intervals of estimated diabetes prevalence covered 94-96% of true data; among subgroups of data, coverage was rarely <90% for both sexes. Median absolute errors ranged from 2.6 to 2.9 percentage points overall, or 1.0 to 7.1 percentage points in all subsets of withheld data. For comparison, median absolute differences for diabetes prevalence between pairs of nationally representative surveys done in the same country and in the same year was 2.4 percentage points, indicating that our estimates almost perform as well as running two parallel surveys in the same country and year.

Appendix Table 1: List of analysis regions and "super-regions", and countries in each region. The hierarchical structure of the statistical model consisted of country, region, super-region, and world.

Super-region	Region
**Sub-Saharan Africa (48)**	**Central Africa (6)**: Angola, Central African Republic, Congo, DR Congo, Equatorial Guinea, Gabon
	**East Africa (17)**: Burundi, Comoros, Djibouti, Eritrea, Ethiopia, Kenya, Madagascar, Malawi, Mauritius†, Mozambique, Rwanda, Seychelles, Somalia, Sudan, Tanzania, Uganda, Zambia
	**Southern Africa (6)**: Botswana, Lesotho, Namibia, South Africa, Swaziland, Zimbabwe
	**West Africa (19)**: Benin, Burkina Faso, Cabo Verde, Cameroon, Chad, Cote d'Ivoire, Gambia, Ghana, Guinea, Guinea Bissau, Liberia, Mali, Mauritania, Niger, Nigeria, Sao Tome and Principe, Senegal, Sierra Leone, Togo
**Central Asia, Middle East and North Africa (28)**	**Central Asia (9)**: Armenia, Azerbaijan, Georgia, Kazakhstan, Kyrgyzstan, Mongolia, Tajikistan, Turkmenistan, Uzbekistan
	**Middle East and North Africa (19)**: Algeria, Bahrain, Egypt, Iran, Iraq, Jordan, Kuwait, Lebanon, Libya, Morocco, Occupied Palestinian Territory, Oman, Qatar, Saudi Arabia, Syrian Arab Republic, Tunisia, Turkey, United Arab Emirates, Yemen
**South Asia (6)**	**South Asia (6)**: Afghanistan, Bangladesh, Bhutan, India, Nepal, Pakistan
**East and Southeast Asia (16)**	**East Asia (4)**: China, China (Hong Kong SAR), North Korea, Taiwan
	**Southeast Asia (12)**: Brunei Darussalam, Cambodia, Indonesia, Lao PDR, Malaysia, Maldives, Myanmar, Philippines, Sri Lanka, Thailand, Timor-Leste, Viet Nam
**Oceania (17)**	**Polynesia and Micronesia (13)**: American Samoa, Cook Islands, French Polynesia, Kiribati, Marshall Islands, Micronesia (Federated States of), Nauru, Niue, Palau, Samoa, Tokelau, Tonga, Tuvalu
	**Melanesia (4):** Fiji, Papua New Guinea, Solomon Islands, Vanuatu
**High-income Asia Pacific (3)**	**High-income Asia Pacific (3)**: Japan, Singapore, South Korea
**Latin America and Caribbean (35)**	**Andean Latin America (3)**: Bolivia, Ecuador, Peru
	**Caribbean (18)**: Antigua and Barbuda, Bahamas, Barbados, Belize, Bermuda, Cuba, Dominica, Dominican Republic, Grenada, Guyana, Haiti, Jamaica, Puerto Rico, Saint Kitts and Nevis, Saint Lucia, Saint Vincent and the Grenadines, Suriname, Trinidad and Tobago
	**Central Latin America (9)**: Colombia, Costa Rica, El Salvador, Guatemala, Honduras, Mexico, Nicaragua, Panama, Venezuela
	**Southern Latin America (5)**: Argentina, Brazil, Chile, Paraguay, Uruguay
**High-income Western countries (27)**	**High-income English-speaking countries* (6)**: Australia, Canada, Ireland, New Zealand, United Kingdom, United States of America
	**North Western Europe (12)**: Austria, Belgium, Denmark, Finland, Germany, Greenland, Iceland, Luxembourg, Netherlands, Norway, Sweden, Switzerland
	**South Western Europe (9)**: Andorra, Cyprus, France, Greece, Israel, Italy, Malta, Portugal, Spain

**Central and Eastern Europe (20)**	**Central Europe (13)**: Albania, Bosnia and Herzegovina, Bulgaria, Croatia, Czech Republic, Hungary, Macedonia (TFYR), Montenegro, Poland, Romania, Serbia, Slovakia, Slovenia
	**Eastern Europe (7)**: Belarus, Estonia, Latvia, Lithuania, Moldova, Russian Federation, Ukraine

† Mauritius was grouped with South Asia in the hierarchical analysis, because of its population composition and epidemiological similarity.

* Although high-income English-speaking countries are geographically separated, they exhibit remarkably similar trends in cardio-metabolic risk factors and outcomes. They were therefore grouped together so that the statistical model shares information amongst them more than it does with other countries that are geographically closer but epidemiologically more distinct.

We did not have data on population by age group for American Samoa, Bermuda, French Polynesia, Greenland, and Tokelau. Country-specific estimates were made but were not used in calculation of regional and global prevalences because the latter requires weighting by age-specific population.

Appendix Table 2: Data sources used in the analysis.

	Country	Data years	Survey/Study name/Citation	Level of representativeness	Rural, urban, or both	Age range as used for global analysis		Sample size		Note
						Male	Female	Male	Female	
1	Algeria	2001	Temmar et al., J Hpertens 25:2218-26, 2007	Community	rural	35+	35+	561	655	
2	Algeria	2003	STEPS	Community	both	25-64	25-64	1,462	2,223	
3	Algeria	2005	Transition and Health Impact in North Africa	National	both	35-70	35-70	1,982	2,727	1
4	Algeria	2007-2009	The ISOR (InSulino-resistance in ORan) study	Community	urban	30-64	30-64	377	409	
5	American Samoa	1994	McGarvey et al., Pac Health Dialog 8:157-62, 2001	National	both	25+	25+	140	209	
6	American Samoa	2004	STEPS	National	both	25-64	25-64	910	1,020	1
7	Argentina	2004-2005	CArdiovascular Risk factors Multiple Evaluation in Latin America (CARMELA)	Community	urban	25-64	25-64	734	748	
8	Argentina	2011-2012	Detection and follow-up of cardiovascular disease and risk factors in the Southern Cone of Latin America. The CESCAS I Study	Community	urban	35-74	35-74	1,560	2,367	
9	Australia	1981	APCSC-Busselton	Community	urban	25+	25+	608	701	
10	Australia	1981	Glatthaar et al., Med J Aust 143:436-40, 1985	Community	both	25+	25+	1,457	1,739	
11	Australia	1988-1989	Dubbo Study of Australian Elderly	Community	urban	59+	59+	819	1,160	
12	Australia	1999-2000	The Australian Diabetes, Obesity and Lifestyle Study (AusDiab)	National	both	25+	25+	5,040	6,137	
13	Australia	1999-2003	North West Adelaide Health Study	Community	urban	18+	18+	1,894	2,094	
14	Australia	2002	Simmons et al., Diabetes Res Clin Pract 70:287-90, 2005	Community	rural	25+	25+	635	819	
15	Australia	2004-2005	The Australian Diabetes, Obesity and Lifestyle Study (AusDiab)	National	both	30+	30+	2,852	3,446	
16	Australia	2004-2006	North West Adelaide Health Study	Community	urban	20+	20+	1,501	1,666	
17	Australia	2008-2010	North West Adelaide Health Study	Community	urban	24+	24+	1,141	1,282	
18	Australia	2011-2012	National Health Measure Survey	National	both	20+	20+	3,318	4,123	
19	Australia	2012	The Australian Diabetes, Obesity and Lifestyle Study (AusDiab)	National	both	37+	37+	2,002	2,479	
20	Austria	1985	VHM&PP; Ulmer et al., J Intern Med 261:566-76, 2007	Subnational	both	20+	20+	32,600	42,176	
21	Austria	1992	Vorarlberg Health Monitoring and Promotion Programme	Subnational	rural	18+	18+	14,104	18,769	
22	Austria	1998	Vorarlberg Health Monitoring and Promotion Programme	Subnational	rural	18+	18+	16,140	20,902	
23	Austria	2004	Vorarlberg Health Monitoring and Promotion Programme	Subnational	rural	18+	18+	20,159	23,890	
24	Bahrain	1995-1996	National Population Register in Bahrain; Al-Mahroos et al., Int J Epidmiol 29:71-6, 2000	National	both	40-59	50-69	1,174	828	1
25	Bahrain	2007	STEPS	National	both	20-64	20-64	863	906	
26	Bangladesh	1999	Chandra Diabetes Study; Hussain et al., Eur J Public Health 17:291-6, 2007	Community	rural	20-59	20-59	2,037	2,720	
27	Bangladesh	2000	Abu Sayeed et al., Diabetes Care 26:1034-9, 2003	Community	rural	20-69	20-69	2,321	2,602	
28	Bangladesh	2002	Abu Sayeed et al., Diabetes Care 27:1054-9, 2004	Community	rural	20-69	20-69	467	650	
29	Bangladesh	2002	Hussain et al., Diabet Med 22:931-6, 2005	Community	rural	20+	20+	2,037	2,720	
30	Bangladesh	2002	Hussain et al., Diabet Med 22:931-6, 2005	Community	urban	20+	20+	731	824	
31	Bangladesh	2011	DHS	National	both	18+	18+	3,546	3,398	1
32	Barbados	1988-1992	Barbados Eye Study; Hennis et al., Int J Epidemiol 31:234-9, 2002	National	both	40-84	40-84	1,840	2,474	
33	Barbados	1992	Foster et al., Ethn Dis 3:404-12, 1993	Community	both	40+	40+	188	272	
34	Barbados	1997-2002	The Barbados Incidence Studies of Eye Diseases II; Nemesure et al., Ann Epidemiol 18:657-63, 2008	National	both	40-59	40-59	606	840	
35	Barbados	2011-2013	Health of the Nation (HotN)	National	both	25+	25+	396	634	
36	Belgium	1991-1994	Flemish Study on Environment, Genes and Health Outcomes (FLEMENGHO)	Community	rural	26-88	26-88	277	287	
37	Belgium	2003	The European Male Ageing Study (EMAS)	Community	both	40+		439		
38	Belgium	2005-2008	Flemish Study on Environment, Genes and Health Outcomes (FLEMENGHO)	Community	rural	18-89	18-89	295	295	
39	Belgium	2008	The European Male Ageing Study (EMAS)	Community	both	40+		356		
40	Belgium	2009-2013	Flemish Study on Environment, Genes and Health Outcomes (FLEMENGHO)	Community	rural	20-88	20-88	301	318	
41	Belgium	2010-2015	Flemish Study on Environment, Genes and Health Outcomes (FLEMENGHO)	Community	rural	18-87	18-87	360	364	
42	Belize	2004-2005	CAMDI	National	both	20+	20+	597	1,020	
43	Benin	2007	STEPS	Community	urban	25-64	25-64	588	977	1
44	Benin	2008	STEPS	National	both	25-64	25-64	1,327	1,474	1
45	Bhutan	2007	STEPS	Community	urban	25-74	25-74	1,116	1,312	
46	Bhutan	2014	STEPS	National	both	18-69	18-69	1,033	1,622	
47	Bolivia	2005-2007	Cardiovascular and metabolic syndrome risk assessment of Bolivian school children and adolescents – Relationships to obesity, diabetes, income, food intake and physical activity	National	both	18	18	110	106	
48	Bosnia and Herzegovina	2012	Non-communicable disease risk factor survey, Federation of B&H	Subnational	rural	18+	18+	831	1,005	
49	Bosnia and Herzegovina	2012	Non-communicable disease risk factor survey, Federation of B&H	Subnational	urban	18+	18+	449	597	

	Country	Data years	Survey/Study name/Citation	Level of representativeness	Rural, urban, or both	Age range as used for global analysis		Sample size		Note
						Male	Female	Male	Female	
50	Botswana	2014	STEPS	National	both	18-69	18-69	1,039	2,166	1
51	Brazil	1991	Fornes et al., Rev Saude Publica 36:12-8, 2002	Community	urban	25+	25+	386	547	
52	Brazil	1992	EPIDOSO; Ramos et al., Rev Saude Publica 32:397-407, 1998	Community	urban	65+	65+	171	293	
53	Brazil	1996-1997	The Bambui Cohort Study of Ageing	Community	urban	18+	18+	890	1,308	
54	Brazil	2000	Schaan et al., Rev Saude Publica 38:529-36, 2004	Subnational	urban	25+	25+	415	471	
55	Brazil	2003	Marquezine et al., Int J Cardiol 129:259-65, 2004	Community	urban	25-64	25-64	1,042	1,787	
56	Brazil	2003-2005	Sao Paulo Ageing & Health Study	Community	urban	65+	65+	760	1,185	
57	Brazil	2004-2006	Hearts of Brazil	National	urban	18+	18+	506	580	1
58	Brazil	2008	The Bambui Cohort Study of Ageing	Community	urban	71+	71+	248	466	
59	Brazil	2010-2013	Baependi Heart Study	Community	rural	18+	18+	504	734	
60	Burkina Faso	2013	STEPS	National	both	25-64	25-64	1,886	1,935	
61	Cabo Verde	2007	STEPS	National	both	25-64	25-64	256	434	1
62	Cambodia	2010	STEPS	National	both	25-64	25-64	1,789	3,234	1
63	Cameroon	1996	Mbanya et al., Diabetologia 40:824-9, 1997	Community	rural	24-74	24-74	292	427	
64	Cameroon	1996	Mbanya et al., Diabetologia 40:824-9, 1997	Community	urban	24-74	24-74	453	587	
65	Cameroon	1998-1999	The Cameroon Essential Non-communicable Disease Health Intervention Project (ENHIP)	Community	rural	18+	18+	467	684	1
66	Cameroon	1998-1999	The Cameroon Essential Non-communicable Disease Health Intervention Project (ENHIP)	Community	urban	18+	18+	393	508	1
67	Cameroon	2000	Defining the relationship between poverty and non-communicable disease burden in Cameroon: Preliminary report; Infobase 101051a1	Subnational	urban	15+	15+	1,641	2,028	2
68	Cameroon	2007	Cameroon Burden of Diabetes - Second Survey	Subnational	urban	18+	18+	3,106	4,101	
69	Canada	1993-1995	Kriska et al., Diabetes Care 24:1787-92, 2001	Community	rural	18-35	18-35	136	180	
70	Canada	2007-2011	Canadian Health Measures Survey	National	both	20-79	20-79	1,204	1,243	
71	Central African Republic	2010	STEPS	Community	both	25-64	25-64	1,751	1,881	1
72	Chile	1992-1993	Miquel et al., Gastroenterology 115:937-46, 1998	Community	urban	18+	18+	654	1,028	
73	Chile	1997	CARMEN; Jadue et al., Rev Med Chil 127:1004-13, 1999	Community	urban	25-64	25-64	1,020	2,100	
74	Chile	2000	Nervi et al., J Hepatol 45:299-305, 2006	Community	urban	18+	18+	335	625	
75	Chile	2003	Encuesta Nacional de Salud	National	both	18+	18+	1,485	1,774	
76	Chile	2004-2005	CArdiovascular Risk factors Multiple Evaluation in Latin America (CARMELA)	Community	urban	25-64	25-64	783	872	
77	Chile	2009-2010	Encuesta Nacional de Salud	National	both	18+	18+	1,765	2,676	
78	Chile	2011-2012	Detection and follow-up of cardiovascular disease and risk factors in the Southern Cone of Latin America. The CESCAS I Study	Community	urban	35-74	35-74	895	983	
79	China	1991-1992	Fangshan Cohort Study	Community	urban	34-86	34-86	266	555	
80	China	1992-1993	Anzhen 02 Cohort Study	Community	urban	34-65	34-65	2,030	2,112	
81	China	1997	DECODA; DECODA Study Group, Diabetes Care 26:1770-80, 2003	Community	urban	30-89	30-89	1,577	2,571	
82	China	1997	CMCS; Wang et al., Zhonghua Nei Ke Za Zhi 46:20-4, 2007	Subnational	both	35-64	35-64	16,252	14,126	
83	China	1998	Lin et al., Am J Cardiol 97:839-42, 2006	Subnational	rural	25-74	25-74	5,073	5,847	
84	China	2000-2001	The International Collaborative Study of Cardiovascular Disease in Asia (InterASIA)	National	both	35-74	35-74	7,327	7,828	
85	China	2002	Dong et al., Diabet Med 22:1427-33, 2005	Community	rural	20-74	20-74	3,350	4,740	
86	China	2002	Dong et al., Diabet Med 22:1427-33, 2005	Community	urban	20-74	20-74	1,997	2,153	
87	China	2002	China National Nutritional and Health Survey	National	both	20+	20+	23,977	27,485	
88	China	2002-2003	Li et al., Diabetes Care 35:1028-30, 2012; Study 1	Community	both	35-74	35-74	5,049	7,278	
89	China	2003	Fan et al., World J Gastroenterol 14:2418-24, 2008	Community	both	25+	25+	5,529	7,770	
90	China	2003	Wu, et al., Prev Med 51:412-5, 2010	Community	both	15+	15+	5,718	7,966	2
91	China	2004	Tian et al., Diabets Res Clin Pract 84:273-8, 2009	Community	rural	35+	35+	364,781	405,011	
92	China	2005	Wei et al., Public Health 124:593-601, 2010	Community	rural	20+	20+	374	397	
93	China	2005	Zhi et al., Chin Med Sci J 23:249-52, 2008	Community	both	18-69	18-69	9,943	10,716	
94	China	2006	Gao et al., Diabet Med 26:1220-7, 2009; Rural	Community	rural	35-74	35-74	1,007	1,443	
95	China	2006	Gao et al., Diabet Med 26:1220-7, 2009; Urban	Community	urban	35-74	35-74	707	1,259	
96	China	2006	Beijing Eye Study	Community	both	45+	45+	1,263	1,675	
97	China	2006-2007	Wang et al., Diabetes Metab 35:378-84, 2009	Community	urban	20+	20+	1,930	4,103	
98	China	2006-2007	Fu et al., BMC Public Health 11:862, 2011	Community	rural	18-64	18-64	1,865	2,641	

	Country	Data years	Survey/Study name/Citation	Level of representativeness	Rural, urban, or both	Age range as used for global analysis		Sample size		Note
						Male	Female	Male	Female	
99	China	2007	Wang et al., Ophthalmology 116:461-7, 2009	Community	rural	30+	30+	2,483	3,114	
100	China	2008	China National Diabetes and Metabolic Disorders Study; Yang et al., N Eng J Med 362:1090-101, 2010	National	both	20+	20+	18,419	27,819	
101	China	2009	Li et al., Diabetes Care 35:1028-30, 2012; Study 2	Community	both	35-74	35-74	3,461	3,962	
102	China	2009	China Health and Nutrition Study	National	both	18+	18+	4,034	4,524	
103	China	2010	Zhang et al., PLoS One 7:e37260, 2012	Community	both	18+	18+	814	982	
104	China	2010	China Noncommunicable Disease Surveillance	National	both	18+	18+	44,647	52,972	
105	China	2011	Beijing Eye Study	Community	both	50+	50+	1,018	1,389	
106	China	2012-2013	The Kailuan Study	Community	urban	18+	18+	81,041	22,148	
107	China (Hong Kong SAR)	1995-1996	Hong Kong Cardiovascular Risk Factor Prevalence Study	National	both	25-74	25-74	1,408	1,480	
108	Colombia	1989	Aschner et al., Diabetes Care 16:90-3, 1993	Community	urban	30+	30+	338	337	
109	Colombia	2001	CINDI/CARMEN-Bucaramaga; Bautista et al., Eur J Cardiovasc Prev Rehabil 13:769-75, 2006	Community	urban	25-64	25-64	622	1,217	
110	Colombia	2004-2005	CArdiovascular Risk factors Multiple Evaluation in Latin America (CARMELA)	Community	urban	25-64	25-64	738	815	
111	Colombia	2007	Encuesta Nacional de Salud	National	both	18-69	18-69	5,054	7,375	1
112	Colombia	2010	STEPS	Subnational	urban	18-64	18-64	564	789	
113	Comoros	2011	STEPS	National	both	25-64	25-64	638	1,764	1
114	Cook Islands	1980	King et al., Med J Aust 145:505-10, 1986	Community	urban	25+	25+	464	494	
115	Cook Islands	1980	King et al., Med J Aust 145:505-10, 1986	Subnational	both	25+	25+	465	494	
116	Costa Rica	1988	Campos et al., Circulation 85:648-58, 1992	Community	rural	20-65	20-65	111	123	
117	Costa Rica	1988	Campos et al., Circulation 85:648-58, 1992	Community	urban	20-65	20-65	111	120	
118	Costa Rica	2000	Ministerio de Salud, 2003	Community	urban	25-64	25-64	475	636	
119	Costa Rica	2004	CAMDI	Community	urban	20+	20+	390	756	
120	Costa Rica	2004-2006	Costa Rican Longevity and Healthy Aging Study Pre-1945 Cohort Wave 1	National	both	60+	60+	1,204	1,438	
121	Costa Rica	2006-2008	Costa Rican Longevity and Healthy Aging Study Pre-1945 Cohort Wave 2	National	both	62+	62+	1,013	1,210	
122	Costa Rica	2010-2011	Costa Rican Longevity and Healthy Aging Study 1945-1955 Cohort Wave 1	National	both	54-66	54-66	1,014	1,568	
123	Cuba	1998	Prevalencia de diabetes mellitus y tolerancia a la glucosa alterada. sus cambios en 20 años en una comunidad de Ciudad de la habana; Infobase 101063a2	Subnational	urban	25+	25+	84	166	
124	Cuba	2010	Third National Risk Factor Survey for NCD	National	both	20+	20+	1,112	1,385	
125	Cuba	2011	Noncommunicable disease risk factor in Cienfuegos	Community	urban	20-80	20-80	560	815	
126	Czech Republic	1981	Machova et al., Cas Lek Cesk 143:90-3, 2004; Site 1	Subnational	rural	25+	25+	9,189	11,004	
127	Czech Republic	1981	Machova et al., Cas Lek Cesk 143:90-3, 2004; Site 2	Subnational	rural	25+	25+	9,189	11,004	
128	Czech Republic	1997-1998	post-Czech-MONICA	National	both	25-64	25-64	1,317	1,477	
129	Czech Republic	2000-2001	post-Czech-MONICA	National	both	25-64	25-64	1,580	1,639	
130	Czech Republic	2007-2008	post-Czech-MONICA	National	both	25-64	25-64	1,671	1,846	
131	Denmark	2006-2008	The Health2006 Cohort	Community	urban	18-71	18-71	1,422	1,799	
132	Denmark	2007-2008	The Danish Health Examination Survey 2007-2008	National	both	18+	18+	6,912	10,082	
133	Denmark	2009-2010	The European Youth Heart Study	Community	both	18-28	18-28	297	322	
134	Dominica	2007	STEPS	National	both	18-64	18-64	41	79	1
135	Dominican Republic	1996-1998	Estudio factores de riesgo cardiovascular en la República Dominicana (EFRICARD); Infobase 100645a1	National	urban	25-84	25-84	1,357	2,625	
136	Ecuador	2004-2005	CArdiovascular Risk factors Multiple Evaluation in Latin America (CARMELA)	Community	urban	25-64	25-64	813	825	
137	Egypt	1991-1993	The Egyptian National Hyperetension Study	National	both	25+	25+	954	1,307	
138	Egypt	1995	Herman et al., Diabet Med 12:1126-31, 1995	Community	urban	20-79	20-79	604	425	
139	Egypt	2003-2004	Metabolic and cardiovascular risk profiles and hepatitis C virus infection in rural Egypt	Community	rural	25+	25+	316	384	
140	Egypt	2005	STEPS	National	both	18-65	18-65	967	1,000	1
141	Egypt	2007-2009	Hepatitis C infection and clearance: impact on atherosclerosis and cardiometabolic risk factors	Community	rural	35+	35+	637	662	
142	Egypt	2011	STEPS	National	both	18-65	18-65	736	1,366	
143	El Salvador	2004	CAMDI	Community	urban	20+	20+	405	822	
144	Eritrea	2010	STEPS	National	both	25-74	25-74	1,555	3,777	1
145	Estonia	1997	SWESTONIA; Johansson et al., J Intern Med 252:551-60, 2002	Community	urban	35-55	35-55	144	133	
146	Estonia	2003	The European Male Ageing Study	Community	both	40+		422		
147	Estonia	2008	The European Male Ageing Study	Community	both	40+		309		
148	Fiji	1980	Zimmet et al., Am J Epidemiol 118:673-88, 1983	Community	rural	25+	25+	382	400	

	Country	Data years	Survey/Study name/Citation	Level of representativeness	Rural, urban, or both	Age range as used for global analysis		Sample size		Note
						Male	Female	Male	Female	
149	Fiji	1980	Zimmet et al., Am J Epidemiol 118:673-88, 1983	Community	urban	25+	25+	661	768	
150	Fiji	2002	STEPS	National	both	18-64	18-64	891	1,423	
151	Fiji	2009	Fiji Eye Health Survey 2009	National	both	40+	40+	576	768	
152	Fiji	2011	STEPS	National	both	25-64	25-64	1,019	1,338	
153	Finland	1984	Tuomilehto et al., Diabetologia 29:611-5, 1986; Site 1	Subnational	both	65-84		296		
154	Finland	1984	Tuomilehto et al., Diabetologia 29:611-5, 1986; Site 2	Subnational	both	65-84		367		
155	Finland	1984	Finnish cohort of the FINE study	Community	rural	65-84		684		
156	Finland	1986	Young Finns Study	National	rural	18-24	18-24	162	192	
157	Finland	1986	Young Finns Study	National	urban	18-24	18-24	208	284	
158	Finland	1987	Tuomilehto et al., Int J Epidemiol 20:1010-7, 1991	Subnational	both	45-64	45-64	1,275	1,443	
159	Finland	1987	Kuusisto et al., Stroke 25:1157-64, 1994	Community	both	66-75	66-75	470	828	
160	Finland	1984-1989	Kuopio Ischaemic Heart Disease Risk Factor Study (KIHD)	Subnational	both	42-60		2,641		
161	Finland	1989	Finnish cohort of the FINE study	Community	rural	70-89		450		
162	Finland	1990-1992	Oulu 35 Study	Community	both	56-57	56-57	231	327	1
163	Finland	1992	Yliharsila et al., Diabet Med 22:88-91, 2005	Subnational	both	45-64	45-64	956	1,131	
164	Finland	1992	DECODE; DECODE Study Group, Diabetes Care 26:61-9, 2003	Community	both	40-69	40-69	861	1,035	
165	Finland	1991-1993	Kuopio Ischaemic Heart Disease Risk Factor Study (KIHD)	Subnational	both	46-64		1,036		
166	Finland	1996-1998	Oulu 35 Study	Community	both	60-63	60-63	244	346	1
167	Finland	1996-1998	Savitaipale Study, baseline	Community	rural	40-66	40-66	575	577	1
168	Finland	1998-2001	Kuopio Ischaemic Heart Disease Risk Factor Study (KIHD)	Subnational	both	53-73	53-73	848	915	
169	Finland	2000-2001	Health 2000 Survey	National	both	30+	30+	1,892	2,272	
170	Finland	2001	Young Finns Study	National	rural	24-39	24-39	344	395	
171	Finland	2001	Young Finns Study	National	urban	24-39	24-39	660	770	
172	Finland	2005	FIN-D2D Survey; Saaristo et al., BMC Public Health 8:423, 2008	Subnational	both	45-74	45-74	1,364	1,461	
173	Finland	2005	Mantyselka et al., Rheumatology (Oxford) 47:1235-8, 2008	Community	rural	30-65	30-65	229	250	
174	Finland	2005-2008	Kuopio Ischaemic Heart Disease Risk Factor Study (KIHD)	Subnational	both	60-81	60-81	1,239	633	
175	Finland	2007	Oulu 35 Study	Community	both	71-73	71-73	182	269	
176	Finland	2007	Young Finns Study	National	rural	30-45	30-45	377	437	
177	Finland	2007	Young Finns Study	National	urban	30-45	30-45	598	715	
178	Finland	2007-2008	Savitaipale Study, follow-up	Community	both	51-75	51-75	431	483	1
179	Finland	2008	Control group for Finnish male former elite athletes	National	both	61+		206		
180	Finland	2011	Young Finns Study	National	rural	34-49	34-49	365	422	
181	Finland	2011	Young Finns Study	National	urban	34-49	34-49	500	630	
182	France	1995-1997	MONICA Lille	Community	urban	36-67	36-67	508	536	
183	France	1995-1997	MONICA Strasbourg	Subnational	both	35-64	35-64	509	437	
184	France	1996	POLA Study; Defay et al., Int J Obes Relat Metab Disord 25:512-8, 2001	Community	both	60+	60+	1,113	1,419	
185	France	1996	Asmar et al., J Hypertens 19:1727-32, 2001	Subnational	both	15+	15+	29,692	31,416	2
186	France	1999-2001	The Three Cty Study	Community	urban	65+	65+	3,420	5,219	
187	France	2003-2005	The Three Cty Study	Community	urban	68+	68+	627	992	
188	France	2004-2006	Monitoring National du Risque Artériel; National Monitoring of Arterial Risk in Lille (MONA LISA Lille)	Community	urban	35-75	35-75	716	731	
189	France	2005-2007	National Monitoring of Arterial Risk in Bas-Rhin (MONA LISA Bas-Rhin)	Subnational	both	35-74	35-74	746	656	
190	France	2006-2007	Etude Nationale Nutrition Santé	National	both	18-74	18-74	738	1,212	
191	France	2008-2010	The Three Cty Study	Community	urban	73+	73+	250	445	
192	France	2011-2013	Enquête LIttorale Souffle Air Biologie EnvironnemenT (ELISABET) Dunkerque	Community	urban	40-64	40-64	669	562	
193	France	2011-2013	Enquête LIttorale Souffle Air Biologie EnvironnemenT (ELISABET) Lille	Community	urban	40-64	40-64	692	789	
194	French Polynesia	2010	STEPS	National	both	18-64	18-64	935	1,236	1
195	Gambia	1997	van der Sande et al., J Hum Hypertens 14:489-95, 2000	Community	both	25-89	25-89	912	1,445	
196	Georgia	2010	STEPS	National	both	18-64	18-64	1,683	4,235	1
197	Germany	1999-2001	KORA S4 Study: Kooperative Research in the Region of Augsburg Survey 4	Community	both	54-75	54-75	1,923	2,157	
198	Germany	2000	Heinz Nixdorf Recall Study; Icks et al., Diabet Med 25:1330-6, 2008	Community	urban	45-74	45-74	2,297	2,304	

	Country	Data years	Survey/Study name/Citation	Level of representativeness	Rural, urban, or both	Age range as used for global analysis		Sample size		Note
						Male	Female	Male	Female	
199	Germany	2000-2002	Epidemiological study of the chances of prevention, early recognition and optimal treatment of chronic diseases in an elderly population (ESTHER), baseline	Subnational	both	50-75	50-75	3,599	4,483	
200	Germany	2002	Echinoccoccus Multilocularis and Internal Diseases in Leutkirch	Community	urban	20-65	20-65	174	189	
201	Germany	2002-2006	Study of Health in Pomerania (SHIP-1) 5-year follow-up	Subnational	both	25-85	25-85	1,569	1,688	
202	Germany	2006-2008	KORA F4 Study: Kooperative Research in the Region of Augsburg Follow-Up of Survey 4	Community	both	31-81	31-81	1,481	1,564	
203	Germany	2008-2011	Epidemiological study of the chances of prevention, early recognition and optimal treatment of chronic diseases in an elderly population (ESTHER), follow-up	Subnational	both	58-84	58-84	2,051	2,438	
204	Germany	2008-2011	German Health Interview and Examination Survey for Adults 2008-2011	National	both	18-79	18-79	3,128	3,420	
205	Ghana	1997	Amoah et al., Diabetes Res Clin Pract 56:197-205, 2002	Subnational	both	25+	25+	1,857	2,875	
206	Ghana	2006	STEPS	Community	urban	25+	25+	689	1,368	1
207	Ghana	2012-2014	Research on Obesity and Diabetes among African Migrants (RODAM), control group in Ghana	Subnational	rural	25+	25+	386	616	
208	Ghana	2012-2014	Research on Obesity and Diabetes among African Migrants (RODAM), control group in Ghana	Subnational	urban	25+	25+	391	1,003	
209	Greece	2001	Karalis et al., BMC Public Health 25:1330-6, 2007	Community	rural	25+	25+	91	103	
210	Greenland	2005-2010	Population Health Survey in Greenland	National	both	18+	18+	1,336	1,710	
211	Guatemala	2001-2002	CAMDI	Community	urban	20+	20+	349	683	
212	Guatemala	2003-2005	The Institute of Nutrition of Central America and Panama (INCAP) Nutrition Supplementation Trial Cohort	Community	both	25-41	25-41	194	259	1
213	Guinea	2009	STEPS	Subnational	both	18-64	18-64	1,027	1,109	1
214	Honduras	2003-2004	CAMDI	Community	urban	20+	20+	435	786	
215	Hungary	1990-1998	Simay et al., Public Health 119:437-41, 2005	Community	both	18+	18+	10,651	13,647	
216	Hungary	2003	The European Male Ageing Study (EMAS)	Community	both	40+		415		
217	Hungary	2008	The European Male Ageing Study (EMAS)	Community	both	40+		344		
218	India	1988-1989	Ramachandran et al., Diabetes Res Clin Pract 58:55-60, 2002	Community	urban	20-74	20-74	438	408	1
219	India	1993	Singh et al., Int J Cardiol 66:65-72, 1998	Community	rural	25-64	25-64	894	875	
220	India	1993	Singh et al., Int J Cardiol 66:65-72, 1998	Community	urban	25-64	25-64	904	902	
221	India	1995	Ramachandran et al., Diabetes Res Clin Pract 42:181-6, 1998	Community	urban	20-74	20-74	741	709	1
222	India	1995-1997	Zargar et al., Diaebtes Res Clin Pract 47:135-46, 2000	Subnational	both	40+	40+	2,496	2,587	
223	India	1996-1999	Chennai Urban Population Study (CUPS)	Community	urban	20+	20+	541	698	
224	India	1999	DECODA; DECODA Study Group, Diabetes Care 26:1770-80, 2003	Community	urban	30-79	30-79	1,297	1,322	
225	India	2000	Ramachandran et al., Diabet Med 20:220-4, 2003	Subnational	urban	20-75	20-75	4,642	5,260	1
226	India	1999-2002	New Delhi Birth Cohort	Community	urban	26-33	26-33	865	620	
227	India	2001	JHW-2; Gupta et al., Diabetes Res Clin Pract 61:69-76, 2003	Community	urban	20+	20+	454	472	
228	India	2001-2004	Chennai Urban Rural Epidemiology Study (CURES)	Community	urban	20+	20+	1,096	1,254	
229	India	2003	Study in Chennai	Community	rural	20-79	20-79	346	575	
230	India	2003	JHW-3; Gupta et al., Indian Heart J 56:646-52, 2004	Community	urban	20+	20+	179	195	
231	India	2004	Fall et al., Diabetes Care 31:2349-56, 2008	Community	urban	26-39	26-39	1,500	1,040	
232	India	2005	Prasad et al., Diabetes Metab Syndr 6:96-101, 2012	Community	urban	20-80	20-80	591	587	
233	India	2006	Ramachandran et al., Diabetes Care 31:893-8, 2008	Community	both	20+	20+	3,321	3,745	
234	India	2006-2008	Central India Eye and Medical Study (CIEMS)	Community	rural	30+	30+	1,430	1,658	
235	India	2006-2008	Zargar et al., Diaebtes Res Clin Pract 82:276-81, 2008	Subnational	both	20-40	20-40	2,124	908	
236	India	2006-2009	New Delhi Birth Cohort	Community	urban	33-38	33-38	648	439	
237	India	2008-2010	ICMR India Diabetes Study	National	both	20+	20+	6,451	6,575	1
238	India	2011-2012	NNMB Rural Repeat Surveys: Diet and NutritionalStatus of Rural Population and Prevalnce of Hypertension	National	rural	18+	18+	14,307	18,507	
239	Indonesia	1981-1982	Waspadji et al., Tohoku J Exp Med 141 Suppl:219-28, 1983	Community	urban	15+	15+	856	906	2
240	Indonesia	2001	STEPS/SURKESNAS	Subnational	both	25+	25+	1,895	2,186	
241	Indonesia	2006	NCD RFS; Soebardi et al., Acta Med Indones 41:186-90, 2009	Community	urban	25-64	25-64	641	950	
242	Iran	1993	Amini et al., Diabetes Res Clin Pract 38:185-190, 1997	Community	urban	40+	40+	1,713	2,197	
243	Iran	1994	Sarraf-Zadegan et al., Acta Cardiol 54:257-63, 1999	Community	urban	20-69	20-69	1,000	1,200	
244	Iran	1999-2000	National Health Survey II	Subnational	both	20+	20+	498	598	
245	Iran	1999-2001	Tehran Lipid and Glucose Study	Community	urban	20+	20+	4,365	6,096	
246	Iran	2001	Report on the pilot project for community-based primary prevention of the major noncommunicable diseases in Qazvin & Abhar cities 2001; Infobase 101013a1	Community	urban	25+	25+	489	495	

	Country	Data years	Survey/Study name/Citation	Level of representativeness	Rural, urban, or both	Age range as used for global analysis		Sample size		Note
						Male	Female	Male	Female	
247	Iran	2001	Report on the pilot project for community-based primary prevention of the major noncommunicable diseases in Qazvin & Abhar cities 2001; Infobase 101013a2	Community	urban	25+	25+	471	496	
248	Iran	2001	Isfahan Healthy Heart Program, Arak	Community	rural	19+	19+	971	1,063	
249	Iran	2001	Isfahan Healthy Heart Program, Arak	Community	urban	19+	19+	2,056	2,104	
250	Iran	2001	Isfahan Healthy Heart Program, Isfahan	Community	rural	19+	19+	233	235	
251	Iran	2001	Isfahan Healthy Heart Program, Isfahan	Community	urban	19+	19+	1,779	1,920	
252	Iran	2001	Isfahan Healthy Heart Program, Najaf Abad	Community	rural	19+	19+	405	416	
253	Iran	2001	Isfahan Healthy Heart Program, Najaf Abad	Community	urban	19+	19+	577	572	
254	Iran	2002-2005	Tehran Lipid and Glucose Study	Community	urban	20+	20+	2,092	2,850	
255	Iran	2003-2004	Childhood and Adolescence Surveillance and Prevention of Adult Noncommunicable Disease R1	National	both	18	18	60	77	
256	Iran	2004	Persian Gulf Healthy Heart Study	Community	urban	25-64	25-64	1,615	1,528	
257	Iran	2004	Azimi-Nezhad et al., Singapore Med J 49:571-6, 2008	Community	both	20+	20+	1,564	1,654	
258	Iran	2005	Provincial Non-Communicable Disease Surveillance Survey 2005	National	both	25-64	25-64	23,428	24,976	
259	Iran	2005-2008	Tehran Lipid and Glucose Study	Community	urban	20+	20+	2,408	3,198	
260	Iran	2007	Isfahan Healthy Heart Program, Arak	Community	rural	19+	19+	1,019	1,020	
261	Iran	2007	Isfahan Healthy Heart Program, Arak	Community	urban	19+	19+	1,421	1,356	
262	Iran	2007	Isfahan Healthy Heart Program, Isfahan	Community	rural	19+	19+	156	151	
263	Iran	2007	Isfahan Healthy Heart Program, Isfahan	Community	urban	19+	19+	1,387	1,420	
264	Iran	2007	Isfahan Healthy Heart Program, Najaf Abad	Community	rural	19+	19+	251	250	
265	Iran	2007	Isfahan Healthy Heart Program, Najaf Abad	Community	urban	19+	19+	493	537	
266	Iran	2007	National Non-Communicable Disease Surveillance Survey 2007	National	both	25-64	25-64	1,445	1,545	
267	Iran	2007	Provincial Non-Communicable Disease Surveillance Survey 2007	National	both	25-64	25-64	9,057	9,755	
268	Iran	2008-2011	Tehran Lipid and Glucose Study	Community	urban	20+	20+	2,463	3,617	
269	Iran	2009-2010	Childhood and Adolescence Surveillance and Prevention of Adult Noncommunicable Disease R3	National	both	18	18	418	405	
270	Iran	2010-2012	Golestan Cohort Study	Subnational	both	40-85	40-85	5,398	5,970	
271	Iran	2011	Provincial Non-Communicable Disease Surveillance Survey 2011	National	both	25-69	25-69	1,979	3,269	
272	Iraq	2005	STEPS	National	both	25-65	25-65	1,817	2,379	
273	Ireland	2006-2007	Survey of Lifestye, Attitudes and Nutritional in Ireland (SLAN)	National	both	45+	45+	497	634	
274	Ireland	2008-2010	National Adult Nutrition Survey	National	both	18+	18+	445	440	
275	Israel	1990	Bar-On et al., Nutr Metab Cardiovasc Dis 2:75-8, 1992	Community	urban	25-64	25-64	1,213	1,335	
276	Israel	1990-1991	The Jerusalem Longitudinal Cohort Study	Community	urban	69-70	69-70	245	206	
277	Israel	1997-1998	The Jerusalem Longitudinal Cohort Study	Community	urban	76-77	76-77	230	222	
278	Israel	1999-2005	The Israel Glucose Intolerance, Obesity and Hypertention Study	National	urban	58+	58+	487	468	
279	Israel	2002-2007	Hadera District Study	Subnational	urban	25-78	25-78	384	374	
280	Israel	2005-2006	The Jerusalem Longitudinal Cohort Study	Community	urban	83-85	83-85	322	381	
281	Italy	1982	Verrillo et al, Diabetes Res 2:301-6, 1985	Community	rural	18+	18+	410	476	
282	Italy	1989	Ventimiglia Heart Study	Community	rural	20+	20+	471	572	
283	Italy	1990	Garancini et al., Diabete Metab 19:116-20, 1993	Community	urban	40+	40+	977	1,252	
284	Italy	1990	Bruneck Study	Community	rural	40-79	40-79	469	450	
285	Italy	1983-1996	Malattie cardiovascolari ATerosclerotiche Istituto Superiore di Sanità (MATISS)	Community	rural	18-77	18-77	3,870	4,368	
286	Italy	1991	DECODE; DECODE Study Group, Diabetes Care 26:61-9, 2003	Community	urban	40-89	40-89	804	1,013	
287	Italy	1992-1993	Italian Longitudinal Study on Aging (ILSA)	National	both	65-84	65-84	1,765	1,608	
288	Italy	1993-1994	MONICA Brianza	Subnational	urban	25-64	25-64	741	789	
289	Italy	1995	Vobarno Study; Muiesan et al, Blood Press 15:14-9, 2006	Community	both	35-64	35-64	265	309	
290	Italy	1995	Bruneck Study	Community	rural	45-84	45-84	411	408	
291	Italy	1995-1996	Italian Longitudinal Study on Aging (ILSA)	National	both	69-90	69-90	1,058	939	
292	Italy	1995-1999	PROgetto Veneto Anziani (PROVA)	Subnational	both	65+	65+	1,218	1,818	
293	Italy	1998-1999	Progetto VIP	Community	both	25-74	25-74	551	572	
294	Italy	1999	InCHIANTI Study; Ferrrucci et al., J Am Geriatr Soc 48:1618-25, 2000	Community	both	25+	25+	582	725	
295	Italy	1998-2002	Osservatorio Epidemiologico Cardiovascolare (OEC)	National	both	35-74	35-74	4,524	4,406	
296	Italy	2000	Bruneck Study	Community	rural	50-89	50-89	331	361	

	Country	Data years	Survey/Study name/Citation	Level of representativeness	Rural, urban, or both	Age range as used for global analysis		Sample size		Note
						Male	Female	Male	Female	
297	Italy	2000-2001	Italian Longitudinal Study on Aging (ILSA)	National	both	73-93	73-93	684	664	
298	Italy	2002	Vobarno Study; Muiesan et al, Blood Press 15:14-9, 2006	Community	both	25-64	25-64	169	216	
299	Italy	2000-2003	PROgetto Veneto Anziani (PROVA)	Subnational	both	67+	67+	781	1,328	
300	Italy	2001-2003	The Study of Asti	Community	both	45-64	45-64	780	878	
301	Italy	2001-2004	Scuteri et al., Nutr Metab Cardiovasc Dis 19:532-41, 2009	Community	urban	35+	35+	1,697	2,265	
302	Italy	2003	The European Male Ageing Study (EMAS)	Community	both	40+		428		
303	Italy	2002-2005	PROgetto Veneto Anziani (PROVA)	Subnational	both	68+	68+	539	986	
304	Italy	2004-2005	Italian Project on the Epidemiolofy of Alzheimer's Disease	National	both	65-84	65-84	1,350	1,199	
305	Italy	2004-2005	Vobarno study	Community	both	55-74	55-74	98	112	
306	Italy	2005	Bruneck Study	Community	rural	55-93	55-93	263	305	
307	Italy	2005-2010	Moli-sani Study	Subnational	both	35+	35+	11,496	12,366	
308	Italy	2008	The European Male Ageing Study (EMAS)	Community	both	40+		57		
309	Italy	2008-2009	Progetto VIP	Community	both	25-74	25-74	599	593	
310	Italy	2010	Bruneck Study	Community	rural	60-98	60-98	225	259	
311	Italy	2008-2012	Osservatorio Epidemiologico Cardiovascolare (OEC)/Health Examination Survey	National	both	35-80	35-80	4,324	4,288	
312	Italy	2010-2012	CArdiovascular risk MEtabolic syndrome LIver and Autoimmunity diseases (CA.ME.LI.A)	Community	both	18-75	18-75	477	515	
313	Italy	2011-2012	Vobarno Study	Community	both	49-62	49-62	107	142	
314	Jamaica	1991	Eldemire et al, West Indian Med J 45:82-4, 1996	National	both	60+	60+	649	669	
315	Jamaica	1995	MacFarlane-Anderson et al., Metabolism 47:617-21, 1998	Community	urban	25+	25+	233	329	
316	Jamaica	1996	Wilks et al., Diabet Med 16:875-83, 1999	Community	urban	25-74	25-74	520	783	
317	Jamaica	2000-2001	Jamaica Health and Lifestyle Survey	National	both	18-74	18-74	566	1,155	
318	Jamaica	2006-2007	Jamaica Youth Risk and Resiliency Behaviour Survey 2006	National	both	18-19	18-19	119	138	1
319	Jamaica	2007-2008	Jamaica Health and Lifestyle Survey	National	both	18-74	18-74	586	1,349	1
320	Japan	1985-1986	Akabane Study	Community	urban	40-69	40-69	471	593	
321	Japan	1987	Konan Town Study	Community	rural	20-79	20-79	69	87	
322	Japan	1988	Konan Town Study	Community	rural	20-79	20-79	76	85	
323	Japan	1989	Konan Town Study	Community	rural	20-79	20-79	59	63	
324	Japan	1989	National Nutrition Survey	National	both	30+	30+	1,377	1,613	
325	Japan	1990	Funagata diabetes study; Sekikawa et al., J Diabetes Complications 14:78-83, 2000	Community	rural	40-79	40-79	1,146	1,478	
326	Japan	1990	Konan Town Study	Community	rural	20-79	20-79	30	58	
327	Japan	1990	National Nutrition Survey	National	both	30+	30+	1,517	1,615	
328	Japan	1991	DECODA; DECODA Study Group, Diabetes Care 26:1770-80, 2003	Community	rural	30-89	30-89	3,896	5,182	
329	Japan	1991	Konan Town Study	Community	rural	20-79	20-79	93	117	
330	Japan	1991	National Nutrition Survey	National	both	30+	30+	1,444	1,523	
331	Japan	1992	Konan Town Study	Community	rural	20-79	20-79	55	52	
332	Japan	1992	National Nutrition Survey	National	both	30+	30+	1,332	1,445	
333	Japan	1993	Konan Town Study	Community	rural	20-79	20-79	54	65	
334	Japan	1993	National Nutrition Survey	National	both	30+	30+	1,251	1,360	
335	Japan	1994	Konan Town Study	Community	rural	20-79	20-79	42	59	
336	Japan	1994	National Nutrition Survey	National	both	20-59	20-59	970	1,112	
337	Japan	1995	Konan Town Study	Community	rural	20-79	20-79	45	60	
338	Japan	1995	National Nutrition Survey	National	both	20-59	20-59	948	1,076	
339	Japan	1996	National Nutrition Survey	National	both	30+	30+	953	1,060	
340	Japan	1997	National Nutrition Survey	National	both	20+	20+	1,196	1,293	
341	Japan	1998	Akabane Study	Community	urban	35-74	35-74	471	593	
342	Japan	1998	National Nutrition Survey	National	both	20+	20+	1,135	1,337	
343	Japan	1999	National Nutrition Survey	National	both	20+	20+	920	1,013	
344	Japan	2000	Niigata Study	Community	both	72	72	232	196	
345	Japan	2000	National Nutrition Survey	National	both	20+	20+	1,073	1,219	
346	Japan	2001	Nakagami et al., Diabetologia 46:1063-70, 2003	Community	rural	35+	35+	783	1,016	
347	Japan	2001	The Japan Association of Health Service Database	Subnational	both	20+	20+	323,553	303,615	

	Country	Data years	Survey/Study name/Citation	Level of representativeness	Rural, urban, or both	Age range as used for global analysis		Sample size		Note
						Male	Female	Male	Female	
348	Japan	2001	Niigata Study	Community	both	73	73	235	198	
349	Japan	2001	National Nutrition Survey	National	both	20+	20+	968	1,088	
350	Japan	2002	Niigata Study	Community	both	74	74	211	186	
351	Japan	2002	National Nutrition Survey	National	both	20+	20+	952	1,072	
352	Japan	2002-2003	The Hisayama Study	Community	rural	40+	40+	1,385	1,827	
353	Japan	2003	National Health and Nutrition Survey	National	both	20+	20+	900	1,059	
354	Japan	2003	Niigata Study	Community	both	75	75	215	191	
355	Japan	2004	National Health and Nutrition Survey	National	both	20+	20+	679	779	
356	Japan	2004	Niigata Study	Community	both	76	76	212	184	
357	Japan	2005	National Health and Nutrition Survey	National	both	20+	20+	660	761	
358	Japan	2005	Niigata Study	Community	both	77	77	202	189	
359	Japan	2006	National Health and Nutrition Survey	National	both	20+	20+	746	903	
360	Japan	2006	Niigata Study	Community	both	78	78	190	193	
361	Japan	2007	National Health and Nutrition Survey	National	both	20+	20+	668	787	
362	Japan	2007	Niigata Study	Community	both	79	79	181	193	
363	Japan	2008	Study on residents in Kanazawa City aged over 40	Community	urban	40+	40+	6,562	11,944	
364	Japan	2008	National Health and Nutrition Survey	National	both	20+	20+	761	851	
365	Japan	2008	Niigata Study	Community	both	80	80	162	164	
366	Japan	2009	National Health and Nutrition Survey	National	both	20+	20+	795	931	
367	Japan	2010	National Health and Nutrition Survey	National	both	20+	20+	711	806	
368	Japan	2011	National Health and Nutrition Survey	National	both	20+	20+	594	733	
369	Japan	2011	The Tokyo Health Service Association Database	Community	urban	20+	20+	6,508	5,232	
370	Japan	2012	National Health and Nutrition Survey	National	both	20+	20+	5,710	8,219	
371	Japan	2013	National Health and Nutrition Survey	National	both	20+	20+	552	579	
372	Jordan	2004	Behavioural Risk Factor Surveillence Survey (BRFSS)	National	both	18+	18+	231	462	
373	Jordan	2007	Behavioural Risk Factor Surveillence Survey (BRFSS)	National	both	18+	18+	315	413	
374	Jordan	2009	Metablic abnomalities and vitamin D study	National	both	18+	18+	786	2,388	
375	Kiribati	1981	Epidemiological survey of Kiribati	Subnational	rural	20+	20+	463	514	
376	Kiribati	1981	Epidemiological survey of Kiribati	Subnational	urban	20+	20+	880	855	
377	Kiribati	2004	STEPS	National	both	18-64	18-64	267	354	1
378	Kuwait	1996	Abdella et al., Diabetes Res Clin Pract 42:187-96, 1998	National	both	20-79	20-79	1,105	1,898	
379	Kuwait	2006	STEPS	National	both	20-65	20-65	814	1,180	
380	Kuwait	2008-2009	National Nutrition Study for the State of Kuwait (NNSSK)	National	both	18-86	18-86	431	503	
381	Kuwait	2014	STEPS	National	both	18-69	18-69	957	1,577	
382	Kyrgyzstan	2013	STEPS	National	both	25-64	25-64	910	1,564	
383	Lao PDR	2013	STEPS	National	both	18-64	18-64	950	1,433	
384	Latvia	2008-2009	Erglis et al., Medicina (Kaunas) 48:310-6, 2012	National	both	25-74	25-74	1,359	2,394	
385	Lebanon	1995	Salti et al., East Mediterr Health J 3:462-71, 1997	Subnational	both	30-89	30-89	1,138	1,380	
386	Lesotho	2012	STEPS	National	both	25-64	25-64	670	1,294	
387	Liberia	2011	STEPS	National	both	25-64	25-64	923	1,199	1
388	Libya	1999	Kadiki et al., Diabetes Metab 27:647-54, 2001	Community	both	25-84	25-84	211	388	
389	Libya	2009	STEPS	National	both	25-64	25-64	975	787	1
390	Lithuania	2001-2002	MONICA4	Community	urban	35-64	35-64	625	776	
391	Lithuania	2006-2008	MONICA4 Follow-up	Community	urban	45-69	45-69	316	425	
392	Luxembourg	2007-2009	Observation des Risques et de la Santé Cardio-Vasculaire au Luxembourg (ORISCAV-LUX)	National	both	18-69	18-69	641	680	
393	Malawi	2009	STEPS	National	both	25-64	25-64	866	1,933	1
394	Malaysia	1995	Mafauzy et al., Asia Pac J Public Health 11:16-9, 1999	Subnational	both	30+	30+	830	1,678	
395	Malaysia	2004	Rampal et al., Asia Pac J Public Health 22:194-202, 2010	National	both	20+	20+	6,261	8,575	
396	Malaysia	2008	Metabolic Syndrome Study in Malaysia	National	both	18+	18+	1,428	2,664	
397	Malaysia	2011	National Health and Morbidity Survey (NHMS)	National	both	18+	18+	3,947	3,132	
398	Maldives	2004	STEPS	Subnational	urban	25-64	25-64	716	839	

	Country	Data years	Survey/Study name/Citation	Level of representativeness	Rural, urban, or both	Age range as used for global analysis		Sample size		Note
						Male	Female	Male	Female	
399	Malta	1981	DECODE; DECODE Study Group, Diabetes Care 26:61-9, 2003	Community	both	30-89	30-89	870	1,173	
400	Malta	1987	Schranz et al., Diabetes Res Clin Pract 7:7-16, 1989	Community	both	35-79	35-79	950	1,178	
401	Marshall Islands	2002	STEPS	National	both	18-64	18-64	380	533	1
402	Mauritania	2006	STEPS	Community	urban	18-64	18-64	920	1,032	
403	Mauritius	1987	Mauritius Noncommunicable Disease Survey	National	both	25-74	25-74	2,351	2,657	
404	Mauritius	1992	Mauritius Noncommunicable Disease Survey	National	both	25-74	25-74	2,991	3,479	
405	Mauritius	1998	Mauritius Noncommunicable Disease Survey	National	both	25-74	25-74	2,483	3,166	
406	Mauritius	2004	Mauritius non communicable disease survey	National	both	25+	25+	2,219	2,279	
407	Mauritius	2009	Mauritius Noncommunicable Disease Survey	National	both	19+	19+	2,880	3,398	
408	Mexico	2004-2005	CArdiovascular Risk factors Multiple Evaluation in Latin America (CARMELA)	Community	urban	25-64	25-64	833	889	
409	Mexico	2006	Encuesta Nacional de Salud Y Nutricion (ENSANUT)	National	both	20+	20+	2,451	3,783	
410	Mexico	2006	PREVENIMSS National Coverage Surveys	Subnational	both	20-98	20-98	8,679	11,251	
411	Mexico	2012	The Mexican Health and Aging Study	National	both	50+	50+	776	1,099	
412	Micronesia (Federated States of)	2002	STEPS	Subnational	both	25-64	25-64	143	223	
413	Micronesia (Federated States of)	2006	STEPS	Subnational	both	25-64	25-64	227	478	1
414	Micronesia (Federated States of)	2008	STEPS	Subnational	both	25-64	25-64	384	592	1
415	Micronesia (Federated States of)	2009	STEPS	Subnational	both	18-64	18-64	141	290	
416	Micronesia (Federated States of)	2009	STEPS	Subnational	both	18-64	18-64	365	454	1
417	Moldova	2013	STEPS	National	both	18-69	18-69	1,320	2,314	
418	Mongolia	2005	STEPS	National	both	18-64	18-64	453	520	1
419	Mongolia	2009	STEPS	National	both	18-64	18-64	604	886	1
420	Mongolia	2013	STEPS	National	both	18-64	18-64	845	1,080	1
421	Morocco	2000	National Survey 2000; Tzai et al., J Hypertens 21:897-903, 2003	National	both	20-89	20-89	689	973	
422	Myanmar	2001	A study of glucose tolerance and associated risk factors in the Hlegu Township; Infobase 200033a1	Subnational	both	30+	30+	1,006	770	
423	Myanmar	2003-2004	STEPS	Subnational	both	25-74	25-74	1,994	2,449	
424	Myanmar	2013-2014	STEPS	Subnational	both	25-74	25-74	681	694	
425	Nauru	1982	Trends in the prevalence and incidence of non-insulin-dependent diabetes mellitus and impaired glucose tolerance	National	both	20+	20+	670	744	
426	Nauru	1987	Trends in the prevalence and incidence of non-insulin-dependent diabetes mellitus and impaired glucose tolerance	National	both	20+	20+	499	598	
427	Nauru	1994	Trends in the prevalence and incidence of non-insulin-dependent diabetes mellitus and impaired glucose tolerance	National	both	25+	25+	645	729	
428	Nauru	2006	STEPS	National	both	18-65	18-65	244	228	
429	Nepal	1990	Sasaki et al., Diabetes Res Clin Pract 67:167-74, 2005	Community	rural	20+	20+	134	127	
430	Nepal	1990	Sasaki et al., Diabetes Res Clin Pract 67:167-74, 2005	Community	urban	20+	20+	98	129	
431	Nepal	2000	Singh et al., Diabet Med 20:170-1, 2003	Subnational	rural	20+	20+	182	235	
432	Nepal	2000	Singh et al., Diabet Med 20:170-1, 2003	Subnational	urban	20+	20+	442	456	
433	Nepal	2002	Shrestha et al., Diabet Med 23:1130-5, 2006	Subnational	urban	40+	40+	423	589	
434	Nepal	2006-2011	Early detection and management of Kidney disease, Hypertension, Diabetes and Cardiovascular disease (KHDC Nepal), Tarahara	Community	rural	18+	18+	1,176	2,351	
435	Nepal	2006-2011	Early detection and management of Kidney disease, Hypertension, Diabetes and Cardiovascular disease (KHDC Nepal), Damak	Community	urban	18+	18+	1,091	1,575	
436	Nepal	2006-2011	Early detection and management of Kidney disease, Hypertension, Diabetes and Cardiovascular disease (KHDC Nepal), Dharan	Community	urban	18+	18+	4,081	6,073	
437	Nepal	2013	STEPS	National	both	18-69	18-69	1,146	2,480	
438	Netherlands	1990	DECODE; DECODE Study Group, Diabetes Care 26:61-9, 2003	Community	urban	50-79	50-79	1,131	1,322	
439	Netherlands	1990	Zutphen Elderly Study	Community	both	69-90		485		
440	Netherlands	1997-1999	the Rotterdam Study, first subcohort	Community	urban	61+	61+	1,508	2,041	
441	Netherlands	1998-2001	Regenboog Project	National	both	18-89	18-89	1,869	1,738	

	Country	Data years	Survey/Study name/Citation	Level of representativeness	Rural, urban, or both	Age range as used for global analysis		Sample size		Note
						Male	Female	Male	Female	
442	Netherlands	2000-2001	the Rotterdam Study, second subcohort	Community	urban	55+	55+	1,109	1,326	
443	Netherlands	2001	Netherlands National Health Examination	National	both	20-79	20-79	1,501	1,514	
444	Netherlands	2001-2003	Surinamese in the Netherlands: Study on Ethnicity and Health	Community	urban	35-60	35-60	243	242	
445	Netherlands	2002-2004	the Rotterdam Study, first subcohort	Community	urban	65+	65+	1,156	1,634	
446	Netherlands	2004-2005	the Rotterdam Study, second subcohort	Community	urban	58+	58+	902	1,163	
447	Netherlands	2006-2008	the Rotterdam Study, third subcohort	Community	urban	45+	45+	1,513	1,972	
448	Netherlands	2009-2010	Measuring the Netherlands (NL de Maat)	Subnational	both	30-70	30-70	1,332	1,545	
449	Netherlands	2009-2011	the Rotterdam Study, first subcohort	Community	urban	72+	72+	640	922	
450	Netherlands	2011-2012	the Rotterdam Study, second subcohort	Community	urban	65+	65+	698	877	
451	Netherlands	2011-2013	Healthy Life in an Urban Setting	Community	urban	18-71	18-71	852	985	
452	Netherlands	2012-2014	the Rotterdam Study, third subcohort	Community	urban	51+	51+	1,190	1,555	
453	New Zealand	2008-2009	The Adult Nutrition Survey	National	both	20+	20+	1,264	1,623	
454	Nicaragua	2003-2004	CAMDI	Community	urban	20+	20+	781	919	
455	Nigeria	1998	Okesina et al., East Afr Med J 76:212-6, 1999	Community	rural	21+	21+	222	120	
456	Nigeria	1999-2009	Prostate Cancer Dietary Risk Factors; Ukoli et al., West Afr J Med 26:7-13, 2007	Subnational	both	35+	35+	446		
457	Niue	1980	King et al., Med J Aust 145:505-10, 1986	National	both	25+	25+	431	519	
458	Norway	2001-2002	The Tromsø Study: Tromsø 5, Tromsø Study Panel	Community	both	30-89	30-89	2,460	3,405	
459	Norway	2007-2008	The Tromsø Study: Tromsø 6	Community	both	30-87	30-87	5,863	6,618	
460	Occupied Palestinian Territory	1996	Husseini et al., Diabet Med 17:746-8, 2000	Community	rural	30-65	30-65	209	291	
461	Occupied Palestinian Territory	1996-1998	Ramallah study	Community	rural	18-64	18-64	203	404	1
462	Occupied Palestinian Territory	1996-1998	Ramallah study	Community	urban	18-64	18-64	182	455	1
463	Occupied Palestinian Territory	2010	STEPS	National	both	18-64	18-64	1,704	3,223	
464	Oman	1991	National Health Survey; Asfour et al., Diabet Med 12:1122-5, 1995	National	both	20+	20+	2,133	2,963	
465	Oman	2000	Oman National Health Survey; Al-Lawati et al., Diab Med 19:954-7, 2002	National	both	20+	20+	2,905	2,933	
466	Pakistan	1994	Basit et al., J Pak Med Assoc 52:357-60, 2002	Subnational	rural	25+	25+	761	1,362	
467	Pakistan	1995	Pakistan National Diabetes Survey	National	rural	25+	25+	1,208	2,243	
468	Pakistan	1995	Pakistan National Diabetes Survey	National	urban	25+	25+	685	1,297	
469	Pakistan	1998	Shera et al., Prim Care Diabetes 4:79-83, 2010	Community	both	25+	25+	815	1,037	
470	Pakistan	2002	Basit et al., Diabetes Res Clin Pract 94:456-62, 2011; Study 1	Subnational	rural	25+	25+	670	1,362	
471	Pakistan	2005	COBRA-1	Community	urban	40+	40+	1,376	1,506	
472	Pakistan	2009-2010	Basit et al., Diabetes Res Clin Pract 94:456-62, 2011; Study 2	Subnational	rural	25+	25+	424	840	
473	Palau	2013	STEPS	National	both	25-64	25-64	832	930	1
474	Panama	2010-2011	Prevalencia de factores de riesgo asociados a enfermedad cardiovascular 2010-2011	Subnational	both	18+	18+	1,074	2,478	
475	Papua New Guinea	1991	Dowse et al., Med J Aust 160:767-74, 1994; Site 1	Community	urban	25-74	25-74	352	312	
476	Papua New Guinea	1991	Dowse et al., Med J Aust 160:767-74, 1994; Site 2	Community	rural	25-74	25-74	91	108	
477	Papua New Guinea	1991	Dowse et al., Med J Aust 160:767-74, 1994; Site 3	Community	rural	25-74	25-74	162	379	
478	Papua New Guinea	1991	Dowse et al., Med J Aust 160:767-74, 1994; Site 4	Community	rural	25-74	25-74	253	487	
479	Papua New Guinea	2007	STEPS	National	both	18-64	18-64	1,252	1,295	1
480	Paraguay	1992	Jimenez et al., Diabet Med 15:334-8, 1998	Community	urban	20-74	20-74	512	1,094	
481	Peru	2004-2005	CArdiovascular Risk factors Multiple Evaluation in Latin America (CARMELA)	Community	urban	25-64	25-64	769	883	
482	Peru	2004-2005	Encuesta Nacional de Indicadores Nutricionales, Bioquímicos, Socioeconómicos y Culturales Relacionados con las Enfermedades Crónicas Degenerativas (ENIN)	National	both	20+	20+	1,984	1,985	
483	Peru	2005	FRENT	Subnational	urban	25+	25+	1,052	2,147	
484	Peru	2005	PREVENCION Study; Medina-Lezama et al., J Am Soc Hypertens 1:216-25, 2007	Community	urban	20-80	20-80	867	1,011	
485	Peru	2007-2008	PERU MIGRANT Study	Community	both	35+	35+	407	441	
486	Peru	2009-2012	CRONICAS Cohort Study	Subnational	both	35+	35+	1,518	1,597	
487	Philippines	1998	National Nutrition Survey; Tanchoco et al., Asia Pac J Clin Nutr 12:271-6, 2003	National	both	20+	20+	1,030	927	

	Country	Data years	Survey/Study name/Citation	Level of representativeness	Rural, urban, or both	Age range as used for global analysis		Sample size		Note
						Male	Female	Male	Female	
488	Philippines	2002	Baltazar et al., Diabetes Res Clin Pract 64:107-15, 2004	Subnational	both	25-65	25-65	2,548	4,445	
489	Philippines	2003-2004	National Nutrition and Health Survey (NNHeS); Dans et al., Phil J Intern Med 43:103-15, 2005	National	both	20+	20+	2,561	2,879	
490	Philippines	2005	Cebu Longitudinal Health and Nutrition Survey 2005 Child Followup	Community	both	20-22	20-22	927	764	
491	Philippines	2005	Cebu Longitudinal Health and Nutrition Survey 2005 Mother Followup	Community	both		35-69		1,872	
492	Philippines	2008	Philippines LIFECARE Cohort	National	both	20-50	20-50	1,329	1,743	
493	Philippines	2009	Life Course Study in Cardiovascular Disease Epidemiology	Subnational	both	20-50	20-50	1,327	1,741	
494	Philippines	2013	8th National Nutrition Survey (NNS)	National	both	18+	18+	69,401	67,644	
495	Poland	1993	DECODE; DECODE Study Group, Diabetes Care 26:61-9, 2003	Community	urban	40-79	40-79	172	192	
496	Poland	2003	The European Male Ageing Study (EMAS)	Community	both	40+		394		
497	Poland	2003-2005	National Multicenter Health Survey in Poland. Project WOBASZ	National	both	20-74	20-74	6,105	6,787	
498	Poland	2008	The European Male Ageing Study (EMAS)	Community	both	40+		309		
499	Poland	2003-2013	Mogielica Human Ecology Study	Community	rural	45+	45+	64	223	
500	Poland	2007-2011	The PolSenior Project	National	both	55+	55+	2,376	2,261	
501	Poland	2010	Zatonska et al., Ann Agric Environ Med 18:265-9, 2011	Community	both	45-64	45-64	1,289	2,570	
502	Poland	2011	The NATPOL 2011 Study	National	both	20-79	20-79	1,115	1,197	
503	Portugal	2001	EPIPorto; Alves et al., Eur J Endocrinol 159:755-60, 2008	Community	urban	25+	25+	847	1,354	
504	Puerto Rico	2006	Pérez et al., Ethn Dis 18:434-41, 2008	Community	urban	25-84	25-84	274	532	
505	Qatar	2007-2008	Qatar National Population Based Study; Abdulbari et al., Diabetes Res Clin Pract 84:99-106, 2009	National	both	20-60	20-60	571	546	
506	Qatar	2012	STEPS	National	both	18-64	18-64	550	876	1
507	Romania	1997	Valorile medii si limitele normalitatii unor constante biologice; Infobase 101221a1	National	both	30-85	30-85	3,964	5,050	
508	Rwanda	2012	STEPS	National	both	18-64	18-64	2,228	3,675	
509	Samoa	1991	Collins et al., Diabetes Care 17:288-96, 1994; Site 1	Community	urban	25+	25+	330	455	
510	Samoa	1991	Collins et al., Diabetes Care 17:288-96, 1994; Site 2	Community	rural	25+	25+	229	234	
511	Samoa	1991	Collins et al., Diabetes Care 17:288-96, 1994; Site 3	Community	rural	25+	25+	233	291	
512	Samoa	1995	McGarvey et al., Pac Health Dialog 8:157-62, 2001	National	both	25+	25+	136	141	
513	Samoa	2002	STEPS	National	both	25-64	25-64	1,149	1,295	1
514	Samoa	2010	Samoan Genome-Wide Association Study	National	both	24-65	24-65	1,166	1,770	
515	Saudi Arabia	1992-1996	Saudi Health Information Survey; Warsy and El-Hazmi, East Mediterr Health J, 5:1236-42, 1999	National	both	15-60	15-60	6,237	8,490	1,2
516	Saudi Arabia	1998	National Epidemiological Health Survey; Al-Nozha et al., Saudi Med J 25:1603-10, 2004	National	both	30-70	30-70	8,002	8,804	
517	Saudi Arabia	2004-2005	STEPS	National	both	15-64	15-64	2,155	2,231	2
518	Saudi Arabia	2009	RIYADH Cohort 2; Al-Daghri et al., BMC Med 9:76, 2011	Community	urban	46-80	46-80	1,409	1,267	
519	Saudi Arabia	2013	Saudi Health Interview Survey	National	both	20+	20+	1,807	2,126	1
520	Seychelles	1989	Seychelles Heart Survey I	National	both	25-64	25-64	513	568	
521	Seychelles	2004	Seychelles Heart Survey III	National	both	25-64	25-64	566	687	
522	Seychelles	2013-2014	Seychelles Heart Survey IV	National	both	25-64	25-64	531	699	
523	Singapore	1982-1985	Thyroid Heart Study	National	both	18+	18+	1,018	978	
524	Singapore	1992	National Health Survey 1992	National	both	18-64	18-64	1,744	1,703	
525	Singapore	1993-1995	NUH Heart Study	National	both	26+	26+	495	484	
526	Singapore	1998	National Health Survey 1998	National	both	18-69	18-69	2,279	2,263	
527	Singapore	2004	National Health Survey 2004	National	both	18-74	18-74	1,796	1,842	
528	Singapore	2004-2007	Combined follow up of Singapore Cardiovascular Cohort study and Singapore Prospective study	National	both	24+	24+	2,465	2,674	
529	Singapore	2009-2011	The Singapore Chinese Eye Study	Community	both	40+	40+	1,581	1,593	
530	Singapore	2010	National Health Survey 2010	National	both	18-69	18-69	1,955	2,083	
531	Singapore	2012-2013	Singapore Health Study 2012	National	both	18-79	18-79	930	1,002	
532	Slovakia	1993	Countrywide Integrated Noncommunicable Diseases Intervention Programme (CINDI)	National	both	18-64	18-64	718	1,177	
533	Slovakia	1998	Countrywide Integrated Noncommunicable Diseases Intervention Programme (CINDI)	National	both	18-64	18-64	855	1,044	
534	Slovakia	2003	Countrywide Integrated Noncommunicable Diseases Intervention Programme (CINDI)	National	both	18-64	18-64	617	866	
535	Slovakia	2008	Countrywide Integrated Noncommunicable Diseases Intervention Programme (CINDI)	National	both	18-64	18-64	390	561	
536	Slovakia	2011-2012	Europan Health Examination Survey (EHES)	National	both	18-64	18-64	878	1,069	
537	Solomon Islands	2006	STEPS	Subnational	both	18-64	18-64	411	531	1
538	South Africa	1990	Mollentze et al., S Afr Med J 85:90-6, 1995	Community	rural	25+	25+	279	576	

	Country	Data years	Survey/Study name/Citation	Level of representativeness	Rural, urban, or both	Age range as used for global analysis		Sample size		Note
						Male	Female	Male	Female	
539	South Africa	1990	Omar et al., S Afr Med J 83:641-3, 1993	Community	urban	25+	25+	88	203	
540	South Africa	1990	Levitt et al., Diabetes Care 16:601-7, 1993	Community	urban	30+	30+	210	514	
541	South Africa	1990	Mollentze et al., S Afr Med J 85:90-6, 1995	Community	urban	25+	25+	290	468	
542	South Africa	2005	Motala et al., Diabetes Care 31:1783-8, 2008	Community	rural	25+	25+	154	689	
543	South Africa	2008-2009	Cape Town Bellville South Cohort Study - Baseline evaluation I	Community	urban	18+	18+	217	706	
544	South Korea	1988	Park et al., Diabetes Care 18:545-8, 1995	Community	both	30+	30+	1,090	1,407	
545	South Korea	2001	Kim et al., Br J Psychiatry 185:102-7, 2004	Community	both	65+	65+	300	432	
546	South Korea	2001	Korea National Health and Nutrition Examination Survey (KNHANES)	National	both	18+	18+	2,227	2,869	
547	South Korea	2005	Korea National Health and Nutrition Examination Survey (KNHANES)	National	both	18+	18+	2,256	3,024	
548	South Korea	2007	Korea National Health and Nutrition Examination Survey (KNHANES)	National	both	18+	18+	1,164	1,610	
549	South Korea	2008	Korea National Health and Nutrition Examination Survey (KNHANES)	National	both	18+	18+	2,691	3,683	
550	South Korea	2009	Korea National Health and Nutrition Examination Survey (KNHANES)	National	both	18+	18+	3,088	3,929	
551	South Korea	2010	Korea National Health and Nutrition Examination Survey (KNHANES)	National	both	18+	18+	2,504	3,266	
552	South Korea	2011	Korea National Health and Nutrition Examination Survey (KNHANES)	National	both	18+	18+	2,456	3,266	
553	South Korea	2012	Korea National Health and Nutrition Examination Survey (KNHANES)	National	both	18+	18+	2,206	3,037	
554	South Korea	2013	Korea National Health and Nutrition Examination Survey (KNHANES)	National	both	18+	18+	2,139	2,770	
555	Spain	1989	Cardiovascular Risk Study in Catalonia	Subnational	both	20-85	20-85	132	134	
556	Spain	1994	de Pablos-Velasco et al., Diabet Med 18:235-41, 2001	Community	urban	30+	30+	305	386	
557	Spain	1994	DECODE; DECODE Study Group, Diabetes Care 26:61-9, 2003	Community	urban	30-89	30-89	2,108	2,699	
558	Spain	1996	REGICOR Study; Masia et al., Rev Esp Cardiol 57:261-4, 2004	Subnational	both	25-74	25-74	874	874	
559	Spain	1999	The Asturias Study; Botas, et al., Diabet Med 20:904-8, 2003	Subnational	both	30-79	30-79	445	542	
560	Spain	1999-2000	Factores de riesgo en las islas Baleares: Estudio CORSAIB	Subnational	both	35-75	35-75	813	775	
561	Spain	2001-2002	Catalan Health Interview Survey	Subnational	both	18-74	18-74	574	728	1
562	Spain	2000-2005	CDC of the Canary Islands	Community	both	18-75	18-75	2,886	3,765	
563	Spain	2003	The European Male Ageing Study (EMAS)	Community	both	40+		402		
564	Spain	2003-2005	Registre Gironi del Cor	Subnational	both	35-79	35-79	2,950	3,278	
565	Spain	2004	Vioque J et al. Obesity 2008; 16: 664-670	Community	urban	24+	24+	66	97	1
566	Spain	2004	Cardiovascular Risk Study in Castilla y León	Subnational	both	18+	18+	1,828	2,000	
567	Spain	2004-2006	PREVICTUS	National	both	60+	60+	3,334	3,825	
568	Spain	2006	Lopez Suarez et al., Rev Esp Cardiol 61:1150-8, 2008	Community	urban	50-75	50-75	398	460	
569	Spain	2007-2009	Harmonizing Equation of Risk in Mediterraneon countries Extremadura (HERMEX)	Subnational	both	25-79	25-79	1,298	1,498	
570	Spain	2008	The European Male Ageing Study (EMAS)	Community	both	40+		255		
571	Spain	2008-2010	Study on Nutrition and Cardiovascular Risk in Spain (ENRICA)	National	both	18+	18+	6,102	6,768	
572	Spain	2009-2010	The Di@bet.es Study; Soriguer et al., Diabetologia 55:88-93, 2012	National	both	18+	18+	2,174	2,898	
573	Sri Lanka	2000	Malavige et al., Diabetes Res Clin Pract 57:143-5, 2002	Community	urban	30-64	30-64	421	621	
574	Sri Lanka	2005-2006	Sri Lanka Diabetes, Cardiovascular Study	National	both	20-69	20-69	1,720	2,668	
575	Sri Lanka	2007	Pinidiyapathirage et al., Diabet Med 30:326-32, 2013	Community	urban	35-64	35-64	1,349	1,636	
576	Sri Lanka	2010	Pubudu De Silva et al., Int J Equity Health 11:76, 2012	Subnational	both	35-64	35-64	628	606	
577	Sudan	2005-2006	STEPS	National	both	25-64	25-64	145	321	
578	Swaziland	2014	STEPS	National	both	18-69	18-69	825	1,520	
579	Sweden	1980-1984	Uppsala Longitudinal Study of Adult Men (ULSAM)	Community	both	60		1,843		
580	Sweden	1990	MONICA Northern Sweden	Subnational	both	25-74	25-74	389	437	
581	Sweden	1991	Asplund-Carlson et al., J Intern Me 236:57-64, 1994	Subnational	both	40-50		1,564		
582	Sweden	1991-1995	Uppsala Longitudinal Study of Adult Men (ULSAM)	Community	both	70		1,219		
583	Sweden	1992-1994	Malmö Diet and Cancer	Community	urban	46-68	46-68	2,283	3,225	
584	Sweden	1994	DECODE; DECODE Study Group, Diabetes Care 26:61-9, 2003	Community	urban	30-79	30-79	1,058	1,120	
585	Sweden	1994	Helicobacter Pylori	Community	urban	56-65	56-65	170	217	
586	Sweden	1994	MONICA Northern Sweden	Subnational	both	25-74	25-74	500	522	
587	Sweden	1995	MONICA Gothenburg	Community	urban	25-64	25-64	452	402	1
588	Sweden	1997	SWESTONIA; Johansson et al., J Intern Med 252:551-60, 2002	Community	urban	35-55	35-55	137	135	
589	Sweden	2001-2003	Uppsala Longitudinal Study of Adult Men (ULSAM)	Community	both	82		511		

	Country	Data years	Survey/Study name/Citation	Level of representativeness	Rural, urban, or both	Age range as used for global analysis		Sample size		Note
						Male	Female	Male	Female	
590	Sweden	2001-2004	Swedish INTERGENE Cohort Study	Subnational	both	24-76	24-76	1,515	1,699	
591	Sweden	2001-2004	Prospective Investigation of the Vasculature in Uppsala Seniors (PIVUS)	Community	both	70	70	505	508	
592	Sweden	2003	The European Male Ageing Study (EMAS)	Community	both	40+		402		
593	Sweden	2002-2006	Malmö Preventive Project Re-examination Study Cohort; Leosdottir et al., Cardiovasc Diabetol 10:118, 2011	Community	both	57-86	57-86	11,546	6,680	
594	Sweden	2004	Welin et al., BMC Public Health 8:403, 2008	Community	urban	50-61	50-61	1,250	667	
595	Sweden	2004	MONICA Northern Sweden	Subnational	both	25-74	25-74	535	544	
596	Sweden	2004-2005	Population Study of Women in Gothenburg	Community	urban		38-50		449	
597	Sweden	2008	The European Male Ageing Study (EMAS)	Community	both	40+		361		
598	Sweden	2006-2009	Prospective Investigation of the Vasculature in Uppsala Seniors (PIVUS)	Community	both	75	75	407	419	
599	Sweden	2009	MONICA Northern Sweden	Subnational	both	25-74	25-74	379	414	
600	Sweden	2014	MONICA Northern Sweden	Subnational	both	25-74	25-74	425	429	
601	Switzerland	2003-2006	Cohorte Lausannoise	Community	urban	35-75	35-75	3,175	3,530	
602	Switzerland	2007-2012	Bus Santé Study	Subnational	both	20-80	20-80	1,884	1,919	
603	Switzerland	2009-2012	Cohorte Lausannoise	Community	urban	40-81	40-81	2,190	2,505	
604	Syrian Arab Republic	2002	National Survey on non-communicable diseases and factors affecting their development; Infobase 102921a1	National	both	15-65	15-65	1,581	2,604	2
605	Taiwan	1988	Chou et al., Diabetes Care 15:81-9, 1992	Community	both	20+	20+	396	458	
606	Taiwan	1991	Chou et al., Diabetes Care 17:1055-8, 1994	Community	both	30+	30+	1,207	1,353	
607	Taiwan	1993-1996	Nutrition and Health Survey in Taiwan (NAHSIT)	National	both	18+	18+	1,349	1,501	1
608	Taiwan	1995	Lu et al., Diabet Med 15:564-72, 1998	Community	urban	20+	20+	781	858	
609	Taiwan	1996	Chen et al., Diabetes Res Clin Pract 44:59-69, 1999	Community	both	40-79	40-79	779	822	
610	Taiwan	1997	Chen et al., Diabetes Res Clin Pract 51:59-66, 2001	Community	both	50-79	50-79	540	1,053	
611	Taiwan	1998	Lai et al., J Gerontol A Biol Sci Med Sci 55:M257-9, 2000	Community	both	65-80	65-80	387	198	
612	Taiwan	1999-2000	Nutrition and Health Survey in Taiwan (NAHSIT)	National	both	65+	65+	735	710	
613	Taiwan	2002	Taiwanese Survey on Hypertension, Hyperglycemia and Hyperlipidemia (TSHHH)	National	both	20+	20+	2,818	3,111	
614	Taiwan	2003	Fuh et al., Diabet Med 24:788-91, 2007	Community	both		40-54		720	
615	Taiwan	2005	TCHS; Lin et al., Eur J Clin Invest 37:783-90, 2007	Community	urban	40+	40+	1,147	1,212	
616	Taiwan	2005-2008	Nutrition and Health Survey in Taiwan (NAHSIT)	National	both	19+	19+	1,276	1,294	
617	Taiwan	2007	Taiwanese Survey on Hypertension, Hyperglycemia and Hyperlipidemia (TSHHH)	National	both	20+	20+	2,024	2,328	
618	Tanzania	1987	Swai et al., BMJ 305:1057-62, 1992	Community	rural	15+	15+	3,301	4,283	1,2
619	Tanzania	1987	McLarty et al., Lancet 1:871-5, 1989	Subnational	rural	25+	25+	1,909	2,396	
620	Tanzania	1989	Swai et al., Diabetes Care 15:1378-85, 1992; Site 1	Community	rural	15+	15+	482	585	2
621	Tanzania	1989	Swai et al., Diabetes Care 15:1378-85, 1992; Site 2	Community	rural	15+	15+	582	802	2
622	Tanzania	1997	Aspray et al., Trans R Soc Trop Med Hyg 94:637-44, 2000	Community	rural	15-64	15-64	401	527	2
623	Tanzania	1997	Aspray et al., Trans R Soc Trop Med Hyg 94:637-44, 2000	Community	urban	15-64	15-64	332	438	2
624	Tanzania	2011	STEPS	Subnational	both	25-64	25-64	897	1,413	1
625	Tanzania	2012	STEPS	National	both	25-64	25-64	2,218	2,420	1
626	Thailand	1983	Vannasaeng et al., J Med Assoc Thai 70 Suppl 2:126-30, 1987	Subnational	urban	20-79	20-79	442	681	
627	Thailand	1991	Thailand National Health Examination Survey I	National	both	18+	18+	5,101	6,893	
628	Thailand	1997	Thailand National Health Examination Survey II	National	both	18-59	18-59	1,017	1,676	
629	Thailand	2000	The International Collaborative Study of Cardiovascular Disease in Asia (InterASIA)	National	both	35+	35+	2,017	3,085	
630	Thailand	2004	Thailand National Health Examination Survey III	National	both	18+	18+	17,319	18,955	
631	Thailand	2009	Thailand National Health Examination Survey IV	National	both	20+	20+	7,975	8,879	
632	Timor-Leste	2009-2010	Timor-Leste Eye Health Survey	Subnational	both	40+	40+	246	248	
633	Timor-Leste	2014	STEPS	National	both	18-69	18-69	970	1,336	
634	Togo	2010	STEPS	National	both	18-64	18-64	1,512	1,594	1
635	Tokelau	2005	STEPS	National	both	18-64	18-64	237	263	1
636	Tonga	1998-2000	Colaguiri et al., Diabetes Care 25:1378-83, 2002	National	both	30+	30+	351	447	
637	Tonga	2004	STEPS	National	both	18-64	18-64	237	301	1
638	Tonga	2011	STEPS	National	both	18-64	18-64	854	1,369	
639	Tunisia	1981	Papoz et al., Int J Epidemiol 17:419-22, 1988	Community	rural	20+	20+	618	893	
640	Tunisia	1990	Gharbi et al., Rev Epidemiol Sante Publique 50:349-55, 2002	Community	both	35-50	35-50	345	345	

	Country	Data years	Survey/Study name/Citation	Level of representativeness	Rural, urban, or both	Age range as used for global analysis		Sample size		Note
						Male	Female	Male	Female	
641	Tunisia	1996-1997	Arian Healthy Project	Community	both	35-66	35-66	647	768	
642	Tunisia	1996-1997	Tunisian National Nutrition Survey	National	both	18+	18+	1,315	2,554	
643	Tunisia	2001	Romdhane et al., Tunis Med 83 Suppl 5:41-6, 2005	Community	both	40-69	40-69	744	1,092	
644	Tunisia	2005	Tunisian National Survey (TAHINA)	National	both	35-71	35-71	3,329	4,590	
645	Tunisia	2009-2010	ObeMaghreb Study, Tunisia	Subnational	urban	18-49	18-49	979	696	
646	Turkey	1990	Turkish Adult Risk Factor Study (TARF)	National	both	20+	20+	1,027	1,128	
647	Turkey	1994	Kelestimur et al., Acta Diabetol 36:85-91, 1999	Community	urban	30-79	30-79	607	845	
648	Turkey	1995	Turkish Adult Risk Factor Study (TARF)	National	both	25+	25+	582	606	
649	Turkey	1998	Turkish Adult Risk Factor Study (TARF)	National	both	28+	28+	445	515	
650	Turkey	1999	Erem et al., Diabetes Res Clin Pract 54:203-8, 2001	Community	urban	20-69	20-69	1,324	1,322	
651	Turkey	2000	Turkish Adult Risk Factor Study (TARF)	National	both	30+	30+	522	592	
652	Turkey	2000-2002	The Healthy Nutrition for Healthy Heart Study; Sanisoglu et al., BMC Public Health 6:92, 2006	National	both	30+	30+	4,778	10,657	
653	Turkey	2001	Yumuk et al., Diabetes Res Clin Pract 70:151-8, 2005	Community	urban	20+	20+	1,042	1,789	
654	Turkey	2001	Sekuri et al., Jpn Heart J 45:119-31, 2004	Community	rural		45+		205	
655	Turkey	2001-2002	Turkish Adult Risk Factor Study (TARF)	National	both	32+	32+	968	1,114	
656	Turkey	2002	Gokcel et al., Diabetes Care 26:3031-4, 2003	Subnational	both	20-79	20-79	607	1,030	
657	Turkey	2002	Soysal et al., Anadolu Kardiyol Derg 5:196-201, 2005	Subnational	urban	25-39	25-39	289	469	
658	Turkey	2005-2006	Turkish Adult Risk Factor Study (TARF)	National	both	33+	33+	645	730	
659	Turkey	2007-2008	Turkish Adult Risk Factor Study (TARF)	National	both	35+	35+	612	721	
660	Turkey	2009-2010	Turkish Adult Risk Factor Study (TARF)	National	both	37+	37+	249	307	
661	Turkey	2010	TURDEP-II; Satman et al., Eur J Epidemiol 28:169-80, 2013	National	urban	20+	20+	5,840	9,943	
662	Turkey	2010	TURDEP-II; Satman et al., Eur J Epidemiol 28:169-80, 2013	National	rural	20+	20+	3,863	6,578	
663	Turkey	2009-2012	Prevalence of diabetes and associated risk factors among adult population in Trabzon city	Subnational	both	20+	20+	1,583	2,138	
664	Turkey	2011	Chronic Disease Risk Factor Survey	National	both	20+	20+	6,214	7,168	
665	Turkey	2012-2013	Turkish Adult Risk Factor Study (TARF)	National	both	37+	37+	645	738	
666	Turkey	2014	Turkish Adult Risk Factor Study (TARF)	National	both	44+	44+	204	232	
667	Turkmenistan	2013	STEPS	National	both	18-64	18-64	1,072	1,857	
668	Uganda	2011-2012	The Prevalence and Distribution of Non-communicable Diseases and Their Risk Factors in Kasese District, Uganda	Subnational	both	25-79	25-79	74	63	1
669	Uganda	2014	STEPS	National	both	18-69	18-69	1,361	1,927	
670	United Arab Emirates	1999-2000	Emirates National Diabetes and Coronary Artery Disease Risk Factor Study (ENDCAD); Malik et al., Diabetes Res Clin Pract 69:188-95	National	both	20-80	20-80	2,839	3,773	
671	United Arab Emirates	2006	Saadi et al., Diabetes Res Clin Pract 78:369-77, 2007	Community	urban	18+	18+	1,176	1,220	
672	United Kingdom	1984-1986	Scottish Heart Health Survey (SHHS)	Subnational	both	40-59	40-59	4,023	3,860	
673	United Kingdom	1987	Croxson et al., Diabet Med 8:28-31, 1991	Community	urban	65-85	65-85	360	497	
674	United Kingdom	1987-1988	Edinburgh Artery Study	Community	urban	54-75	54-75	797	769	
675	United Kingdom	1993	DECODE; DECODE Study Group, Diabetes Care 26:61-9, 2003	Community	urban	30-79	30-79	415	384	
676	United Kingdom	1993	Whickham Survey; Vanderpump et al., Diabet Med 13:741-7, 1996	Community	urban	35+	35+	761	938	
677	United Kingdom	1998-2000	The British Regional Heart Study	National	urban	60-79	60-79	3,879		
678	United Kingdom	1999-2001	British Women's Heart and Health Study	National	both		60-79		3,501	
679	United Kingdom	1999-2004	Hertfordshire Cohort Study	Subnational	both	59-73	59-73	1,452	1,318	
680	United Kingdom	2003	The European Male Ageing Study (EMAS)	Community	both	40+		386		
681	United Kingdom	2003	Health Survey for England (HSE)	National	both	18+	18+	1,452	1,593	
682	United Kingdom	2003	Scottish Healthy Survey (SHeS)	Subnational	both	18+	18+	1,839	2,155	
683	United Kingdom	2004-2005	English Longitudinal Study of Ageing (ELSA)	National	both	52-80	52-80	1,884	2,115	
684	United Kingdom	2005	Health Survey for England (HSE)	National	both	65+	65+	989	1,174	
685	United Kingdom	2006	Health Survey for England (HSE)	National	both	18+	18+	2,916	3,478	
686	United Kingdom	2008	The European Male Ageing Study (EMAS)	Community	both	40+		306		
687	United Kingdom	2008	Health Survey for England (HSE)	National	both	18+	18+	1,505	1,804	
688	United Kingdom	2008	Scottish Healthy Survey (SHeS)	Subnational	both	18+	18+	395	462	
689	United Kingdom	2006-2010	MRC National Survey of Health and Development (NSHD)	National	both	60-64	60-64	879	961	
690	United Kingdom	2008-2009	English Longitudinal Study of Ageing (ELSA)	National	both	50+	50+	2,201	2,595	

	Country	Data years	Survey/Study name/Citation	Level of representativeness	Rural, urban, or both	Age range as used for global analysis		Sample size		Note
						Male	Female	Male	Female	
691	United Kingdom	2009	Health Survey for England (HSE)	National	both	18+	18+	1,050	1,210	
692	United Kingdom	2009	Scottish Healthy Survey (SHeS)	Subnational	both	18+	18+	360	468	
693	United Kingdom	2010	Health Survey for England (HSE)	National	both	18+	18+	1,678	2,112	
694	United Kingdom	2010	Scottish Healthy Survey (SHeS)	Subnational	both	18+	18+	337	431	
695	United Kingdom	2011	Health Survey for England (HSE)	National	both	18+	18+	1,697	2,151	
696	United Kingdom	2011	Scottish Healthy Survey (SHeS)	Subnational	both	18+	18+	321	371	
697	United Kingdom	2012	Health Survey for England (HSE)	National	both	18+	18+	1,706	2,132	
698	United Kingdom	2012-2013	English Longitudinal Study of Ageing (ELSA)	National	both	50+	50+	2,014	2,409	
699	United Kingdom	2013	Health Survey for England (HSE)	National	both	18+	18+	2,003	2,375	
700	United States of America	1976-1980	US NHANES II	National	both	20-74	20-74	1,834	2,075	3
701	United States of America	1983-1987	Offspring Cohort of Framingham Heart Study, Exam 3	Community	urban	22-77	18-76	1,483	1,516	
702	United States of America	1985-1986	The Coronary Artery Risk Development in (Young) Adults (CARDIA) Study, Y0	Subnational	urban	18-30	18-30	2,204	2,666	
703	United States of America	1987	The Atherosclerosis Risk in Communities Study (ARIC), Exam 1	Subnational	both	44-66	44-66	6,421	7,731	
704	United States of America	1987-1991	Offspring Cohort of Framingham Heart Study, Exam 4	Community	urban	26-81	22-79	1,576	1,590	
705	United States of America	1989-1990	The Cardiovascular Health Study (CHS), Baseline	Subnational	both	65+	65+	2,156	2,836	
706	United States of America	1988-1994	US NHANES III	National	both	20+	20+	3,332	3,658	
707	United States of America	1990-1992	The Atherosclerosis Risk in Communities Study (ARIC), Exam 2	Subnational	both	47-70	46-70	5,853	7,113	
708	United States of America	1991-1995	Offspring Cohort of Framingham Heart Study, Exam 5	Community	urban	30-82	26-84	1,627	1,759	
709	United States of America	1992-1993	The Coronary Artery Risk Development in (Young) Adults (CARDIA) Study, Y7	Subnational	urban	25-37	25-37	1,667	1,981	
710	United States of America	1992-1993	The Cardiovascular Health Study (CHS), Year 5	Subnational	both	67+	67+	1,701	2,279	
711	United States of America	1995-1996	The Bogalusa Heart Study (BHS), Cycle 8	Community	rural	20-37	20-37	33	38	
712	United States of America	1995-1996	The Coronary Artery Risk Development in (Young) Adults (CARDIA) Study, Y10	Subnational	urban	28-40	28-40	1,712	2,119	
713	United States of America	1995-1998	Offspring Cohort of Framingham Heart Study, Exam 6	Community	urban	35-86	29-85	1,480	1,698	
714	United States of America	1996-1997	The Cardiovascular Health Study (CHS), Year 9	Subnational	both	72+	72+	1,176	1,714	
715	United States of America	1996-1997	Study of Women's Health Across the Nation (SWAN)	National	both		40-55		3,066	
716	United States of America	1996-1998	The Atherosclerosis Risk in Communities Study (ARIC), Exam 4	Subnational	both	52-75	53-75	4,570	5,637	
717	United States of America	1997-1999	Study of Women's Health Across the Nation (SWAN)	National	both		40-55		2,562	
718	United States of America	1998-2001	Offspring Cohort of Framingham Heart Study, Exam 7	Community	urban	37-89	33-90	1,357	1,585	
719	United States of America	1999-2000	US NHANES 1999-2000	National	both	18+	18+	972	973	
720	United States of America	1999-2001	Study of Women's Health Across the Nation (SWAN)	National	both		40-56		2,223	
721	United States of America	2000-2001	The Coronary Artery Risk Development in (Young) Adults (CARDIA) Study, Y15	Subnational	urban	32-49	32-47	697	973	
722	United States of America	2000-2002	Study of Women's Health Across the Nation (SWAN)	National	both		40-57		2,212	
723	United States of America	2001-2002	US NHANES 2001-2002	National	both	18+	18+	1,181	1,126	
724	United States of America	2002-2005	Third Generation Cohort (Gen III) of Framingham Heart Study, Exam 1	Community	urban	19-72	19-70	1,661	1,880	
725	United States of America	2003-2004	US NHANES 2003-2004	National	both	18+	18+	1,069	1,044	
726	United States of America	2005-2006	The Coronary Artery Risk Development in (Young) Adults (CARDIA) Study, Y20	Subnational	urban	38-50	38-50	1,498	1,963	
727	United States of America	2005-2006	US NHANES 2005-2006	National	both	18+	18+	1,084	974	
728	United States of America	2007-2008	US NHANES 2007-2008	National	both	18+	18+	1,218	1,233	
729	United States of America	2008-2009	National Longitudinal Study of Adolescent Health (AddHealth) Wave IV	National	both	24-34	24-34	2,093	2,580	
730	United States of America	2009-2010	US NHANES 2009-2010	National	both	18+	18+	1,296	1,437	
731	United States of America	2011-2012	US NHANES 2011-2012	National	both	18+	18+	1,214	1,195	
732	Uruguay	1992	Enfermedades Cardiovasculares	National	both	19+	19+	449	574	
733	Uruguay	2011-2012	Detection and follow-up of cardiovascular disease and risk factors in the Southern Cone of Latin America. The CESCAS I Study	Community	urban	35-74	35-74	635	915	
734	Uzbekistan	2002	DHS	Subnational	urban	18-59	18-49	551	625	
735	Uzbekistan	2014	STEPS	National	both	18-64	18-64	1,463	2,088	1
736	Vanuatu	1998	1998 Vanuatu non-comunicable disease survey report; Infobase 101417a1	National	both	20-59	20-59	185	179	
737	Vanuatu	2011	STEPS	National	both	25-64	25-64	2,202	2,151	1
738	Venezuela	2000	Zulia Coronary Heart Disease Risk Factor Study; Florez et al., Diabetes Res Clin Pract 69:63-77, 2005	Subnational	both	25+	25+	1,120	2,552	
739	Venezuela	2004-2005	CArdiovascular Risk factors Multiple Evaluation in Latin America (CARMELA)	Community	urban	25-64	25-64	713	1,135	
740	Venezuela	2005-2006	Brajkovich et al., Rev Ven Endoc Metab 4:31-2, 2006	Community	urban	20-65	20-65	204	439	

	Country	Data years	Survey/Study name/Citation	Level of representativeness	Rural, urban, or both	Age range as used for global analysis		Sample size		Note
						Male	Female	Male	Female	
741	Venezuela	2007-2008	Venezuelan Study of Metabolic Syndrome, Obesity and Lifestyle (VEMSOLS)	Community	urban	20+	20+	106	229	
742	Venezuela	2008-2009	Venezuelan Study of Metabolic Syndrome, Obesity and Lifestyle (VEMSOLS)	Community	rural	20+	20+	51	89	
743	Venezuela	2010-2011	Venezuelan Study of Metabolic Syndrome, Obesity and Lifestyle (VEMSOLS)	Community	urban	20+	20+	51	154	
744	Viet Nam	1990	Quoc et al., Am J Epidemiol 139:713-22, 1994	Community	both	25-69	25-69	1,712	2,061	
745	Viet Nam	2001	Duc Son et al., Diabet Med 21:371-6, 2004	Community	both	25+	25+	654	2,001	
746	Viet Nam	2005	Non-communicable disease risk factors in Ho Chi Minh City	Community	both	25-64	25-64	889	827	1
747	Viet Nam	2008-2009	The survey on diabetes and its risk factors in 2 northern provinces of Vietnam (DM-S)	Subnational	both	25+	25+	824	1,435	
748	Viet Nam	2009	STEPS	National	both	25-64	25-64	6,437	7,486	1
749	Viet Nam	2011	Quang Binh et al., BMC Public Health 12:939, 2012	Subnational	rural	40-64	40-64	1,761	949	
750	Zambia	2008	STEPS	Community	urban	25+	25+	597	1,174	1
751	Zimbabwe	2005	Zimbabwe Non-Communicable Disease Risk Factors (ZiNCoDs/STEPS) Preliminary Report; Infobase 102537a1	National	both	25+	25+	444	1,393	

1. Fasting glucose was measured in capillary whole blood, and was converted to plasma-equivalent by multiplying by 1.11.[3]
2. The first age group started from 15 years old, but had a mean age >18 years.
3. National study conducted between 1977 and 1979, included in the analysis as a 1980 study.

Appendix Table 3: Availability of diabetes and glycaemia metrics in sources that did not report the primary outcome (prevalence defined by FPG ≥7.0 mmol/L or history of diabetes diagnosis or use of diabetes medication), and choices of predictors for use in conversion regressions described in Appendix 2.

**Metrics available from a previous pooling or extracted from a published study**	**Number of data points (per cent of total data) reported using these metrics and needing conversion to primary outcome**	**Independent variable used in the conversion regression** [a]
Mean FPG	767 (9.9%)	Mean FPG [b]
Mean 2hOGTT	37 (0.5%)	Mean 2hOGTT [b]
Mean HbA1c	39 (0.5%)	Mean HbA1c [b]
Prevalence (FPG ≥6.0 mmol/L)	15 (0.2%)	Prevalence (FPG ≥6.0 mmol/L)
Prevalence (FPG ≥6.1 mmol/L)	14 (0.2%)	Prevalence (FPG ≥6.1 mmol/L)
Prevalence (FPG ≥6.7 mmol/L)	20 (0.3%)	Prevalence (FPG ≥6.7 mmol/L)
Prevalence (FPG ≥6.8 mmol/L)	24 (0.3%)	Prevalence (FPG ≥6.8 mmol/L)
Prevalence (FPG ≥7.0 mmol/L)	38 (0.5%)	Prevalence (FPG ≥7.0 mmol/L)
Prevalence (FPG ≥7.8 mmol/L)	8 (0.1%)	Prevalence (FPG ≥7.8 mmol/L)
Prevalence (FPG ≥5.5 mmol/L or history of diabetes diagnosis or use of diabetes medication)	6 (0.1%)	Prevalence (FPG ≥5.5 mmol/L or history of diabetes diagnosis or use of diabetes medication)
Prevalence (FPG ≥5.6 mmol/L or history of diabetes diagnosis or use of diabetes medication)	8 (0.1%)	Prevalence (FPG ≥5.6 mmol/L or history of diabetes diagnosis or use of diabetes medication)
Prevalence (FPG ≥6.1 mmol/L or history of diabetes diagnosis or use of diabetes medication)	11 (0.1%)	Prevalence (FPG ≥6.1 mmol/L or history of diabetes diagnosis or use of diabetes medication)
Prevalence (FPG ≥6.8 mmol/L or history of diabetes diagnosis or use of diabetes medication)	12 (0.2%)	Prevalence (FPG ≥6.8 mmol/L or history of diabetes diagnosis or use of diabetes medication)
Prevalence (FPG ≥7.8 mmol/L or history of diabetes diagnosis or use of diabetes medication)	8 (0.1%)	Prevalence (FPG ≥7.8 mmol/L or history of diabetes diagnosis or use of diabetes medication)
Prevalence (2hOGTT ≥11.1 mmol/L)	20 (0.3%)	Prevalence (2hOGTT ≥11.1 mmol/L)
Prevalence (2hOGTT ≥11.1 mmol/L or history of diabetes diagnosis or use of diabetes medication)	229 (3.0%)	Prevalence (2hOGTT ≥11.1 mmol/L or history of diabetes diagnosis or use of diabetes medication)

Prevalence (FPG ≥7.0 mmol/L or 2hOGTT ≥11.1 mmol/L)	44 (0.6%)	Prevalence (FPG ≥7.0 mmol/L or 2hOGTT ≥11.1 mmol/L)
Prevalence (FPG ≥7.8 mmol/L or 2hOGTT ≥11.1 mmol/L)	52 (0.7%)	Prevalence (FPG ≥7.8 mmol/L or 2hOGTT ≥11.1 mmol/L)
Prevalence (FPG ≥7.0 mmol/L or 2hOGTT ≥11.1 mmol/L or history of diabetes diagnosis or use of diabetes medication)	146 (1.9%)	Prevalence (FPG ≥7.0 mmol/L or 2hOGTT ≥11.1 mmol/L or history of diabetes diagnosis or use of diabetes medication)
Prevalence (FPG ≥7.8 mmol/L or 2hOGTT ≥11.1 mmol/L or history of diabetes diagnosis or use of diabetes medication)	151 (2.0%)	Prevalence (FPG ≥7.8 mmol/L or 2hOGTT ≥11.1 mmol/L or history of diabetes diagnosis or use of diabetes medication)
Prevalence (HbA1c ≥10% or history of diabetes diagnosis or use of diabetes medication)	14 (0.2%)	Prevalence (HbA1c ≥10% or history of diabetes diagnosis or use of diabetes medication)
Prevalence (FPG ≥7.0 mmol/L or HbA1c ≥6.5% or history of diabetes diagnosis or use of diabetes medication)	6 (0.1%)	Prevalence (FPG ≥7.0 mmol/L or HbA1c ≥6.5% or history of diabetes diagnosis or use of diabetes medication)
Prevalence (FPG ≥7.0 mmol/L or 2hOGTT ≥11.1 mmol/L or HbA1c ≥6.5% or history of diabetes diagnosis or use of diabetes medication)	2 (0.0%)	Prevalence (FPG ≥7.0 mmol/L or 2hOGTT ≥11.1 mmol/L or HbA1c ≥6.5% or history of diabetes diagnosis or use of diabetes medication)
Mean FPG<br>Mean 2hOGTT	11 (0.1%)	Mean FPG [b]
Mean FPG<br>Prevalence (FPG ≥7.0 mmol/L or 2hOGTT ≥11.1 mmol/L or history of diabetes diagnosis or use of diabetes medication)	10 (0.1%)	Mean FPG [b]
Mean FPG<br>Prevalence (FPG ≥7.8 mmol/L or 2hOGTT ≥11.1 mmol/L or history of diabetes diagnosis or use of diabetes medication)	2 (0.0%)	Mean FPG [b]
Mean FPG<br>Prevalence (FPG ≥6.7 mmol/L)	8 (0.1%)	Mean FPG [b]
Mean FPG<br>Prevalence (FPG ≥7.0 mmol/L or 2hOGTT ≥11.1 mmol/L or history of diabetes diagnosis or use of diabetes medication)<br>Prevalence (FPG in 6.1-7.0 mmol/L)	2 (0.0%)	Mean FPG [b]
Mean FPG<br>Mean HbA1c<br>Prevalence (HbA1c ≥6.5% or history of diabetes diagnosis or use of diabetes medication)<br>Prevalence of impaired fasting glucose (FPG ≥5.6 mmol/L and FPG <7.0 mmol/L and no diabetes diagnosis and no use of diabetes medication)	2 (0.0%)	Mean FPG [b]
Prevalence (FPG ≥7.0 mmol/L)<br>Prevalence (FPG in 6.1-7.0 mmol/L)	30 (0.4%)	Prevalence (FPG ≥7.0 mmol/L)
Prevalence (FPG ≥7.0 mmol/L)<br>Prevalence of impaired fasting glucose (FPG in 5.6-7.0 mmol/L and no	8 (0.1%)	Prevalence (FPG ≥7.0 mmol/L)

diabetes diagnosis and no use of diabetes medication)		
Prevalence (FPG ≥7.0 mmol/L or 2hOGTT ≥11.1 mmol/L or history of diabetes diagnosis or use of diabetes medication)<br>Prevalence (FPG in 6.1-7.0 mmol/L)	12 (0.2%)	Prevalence (FPG ≥7.0 mmol/L or 2hOGTT ≥11.1 mmol/L or history of diabetes diagnosis or use of diabetes medication)
Prevalence (FPG ≥7.0 mmol/L or 2hOGTT ≥11.1 mmol/L or history of diabetes diagnosis or use of diabetes medication)<br>Prevalence of impaired glucose tolerance (FPG <7.0 mmol/L and 2hOGTT in 7.8-11.1 mmol/L and no diabetes diagnosis and no use of diabetes medication)	12 (0.2%)	Prevalence (FPG ≥7.0 mmol/L or 2hOGTT ≥11.1 mmol/L or history of diabetes diagnosis or use of diabetes medication)
Prevalence (FPG ≥7.0 mmol/L or 2hOGTT ≥11.1 mmol/L or history of diabetes diagnosis or use of diabetes medication)<br>Prevalence of impaired fasting glucose (FPG in 5.6-7.0 mmol/L and 2hOGTT <7.8 mmol/L and no diabetes diagnosis and no use of diabetes medication)<br>Prevalence of impaired glucose tolerance (FPG <5.6 mmol/L and 2hOGTT in 7.8-11.1 mmol/L and no diabetes diagnosis and no use of diabetes medication)	10 (0.1%)	Prevalence (FPG ≥7.0 mmol/L or 2hOGTT ≥11.1 mmol/L or history of diabetes diagnosis or use of diabetes medication)
Prevalence (FPG ≥7.0 mmol/L or 2hOGTT ≥11.1 mmol/L or history of diabetes diagnosis or use of diabetes medication)<br>Prevalence of impaired glucose tolerance (2hOGTT in 7.8-11.1 mmol/L and no diabetes diagnosis and no use of diabetes medication)	18 (0.2%)	Prevalence (FPG ≥7.0 mmol/L or 2hOGTT ≥11.1 mmol/L or history of diabetes diagnosis or use of diabetes medication)
Mean 2hOGTT<br>Prevalence (FPG ≥7.0 mmol/L or 2hOGTT ≥11.1 mmol/L or history of diabetes diagnosis or use of diabetes medication)<br>Prevalence (2hOGTT in 7.8-11.1 mmol/L)	6 (0.1%)	Prevalence (FPG ≥7.0 mmol/L or 2hOGTT ≥11.1 mmol/L or history of diabetes diagnosis or use of diabetes medication)
Prevalence (FPG ≥7.8 mmol/L or 2hOGTT ≥11.1 mmol/L or history of diabetes diagnosis or use of diabetes medication)<br>Prevalence of impaired glucose tolerance (FPG <7.8 mmol/L and 2hOGTT in 7.8-11.1 mmol/L and no diabetes diagnosis and no use of diabetes medication)	30 (0.4%)	Prevalence (FPG ≥7.8 mmol/L or 2hOGTT ≥11.1 mmol/L or history of diabetes diagnosis or use of diabetes medication)
Prevalence (FPG ≥7.8 mmol/L or 2hOGTT ≥11.1 mmol/L or history of diabetes diagnosis or use of diabetes medication)<br>Prevalence of impaired glucose tolerance (2hOGTT in 7.8-11.1 mmol/L and no diabetes diagnosis and no use of diabetes medication)	16 (0.2%)	Prevalence (FPG ≥7.8 mmol/L or 2hOGTT ≥11.1 mmol/L or history of diabetes diagnosis or use of diabetes medication)
Prevalence (FPG ≥7.0 mmol/L or 2hOGTT ≥11.1 mmol/L or HbA1c ≥6.5% or history of diabetes diagnosis or use of diabetes medication)<br>Prevalence of impaired glucose tolerance (2hOGTT in 7.8-11.1 mmol/L and no diabetes diagnosis and no use of diabetes medication)	10 (0.1%)	Prevalence (FPG ≥7.0 mmol/L or 2hOGTT ≥11.1 mmol/L or HbA1c ≥6.5% or history of diabetes diagnosis or use of diabetes medication)

Mean FPG<br>Prevalence (FPG ≥7.0 mmol/L)	257 (3.3%)	Mean FPG [b]<br>Prevalence (FPG ≥7.0 mmol/L)
Mean FPG<br>Mean HbA1c<br>Prevalence (FPG ≥7.0 mmol/L)	54 (0.7%)	Mean FPG [b]<br>Prevalence (FPG ≥7.0 mmol/L)
Mean FPG<br>Mean 2hOGTT<br>Prevalence (FPG ≥7.0 mmol/L)	14 (0.2%)	Mean FPG [b]<br>Prevalence (FPG ≥7.0 mmol/L)
Mean FPG<br>Mean HbA1c<br>Prevalence (FPG ≥7.0 mmol/L)Prevalence (FPG in 6.1-7.0 mmol/L)	15 (0.2%)	Mean FPG [b]<br>Prevalence (FPG ≥7.0 mmol/L)
Mean FPG<br>Prevalence (FPG ≥7.0 mmol/L)<br>Prevalence (FPG in 6.1-7.0 mmol/L)	334 (4.3%)	Mean FPG [b]<br>Prevalence (FPG ≥7.0 mmol/L)
Mean FPG<br>Prevalence (FPG ≥7.0 mmol/L)<br>Prevalence (FPG ≥7.8 mmol/L or history of diabetes diagnosis or use of diabetes medication)	2 (0.0%)	Mean FPG [b]<br>Prevalence (FPG ≥7.0 mmol/L)
Mean FPG<br>Prevalence (FPG ≥7.0 mmol/L)<br>Prevalence (FPG ≥7.8 mmol/L or 2hOGTT ≥11.1 mmol/L)	10 (0.1%)	Mean FPG [b]<br>Prevalence (FPG ≥7.0 mmol/L)
Mean HbA1c<br>Prevalence (HbA1c ≥6.5% or history of diabetes diagnosis or use of diabetes medication)	392 (5.1%)	Mean HbA1c [b]<br>Prevalence (HbA1c ≥6.5% or history of diabetes diagnosis or use of diabetes medication)

[a] All prevalences were probit-transformed to avoid negative prevalences and because the fit to the data was better.
[b] We used 1/mean because it provided better fit according to Bayesian information criterion (BIC).[2]

Appendix Table 4: Model specifications and regression coefficients to estimate the primary outcome (prevalence defined by FPG ≥7.0 mmol/L or history of diabetes diagnosis or use of diabetes medication) from other metrics. The dependent variable in all regressions was prevalence, fitted using a generalised linear (mixed) model with a probit link function. Random effects for super-regions are presented when they were included in the models.

* denotes statistical interaction. CI: confidence interval.

Independent variable: mean FPG	
**Variables**	**Coefficients (95% CI)**
Intercept	1.01 (0.86, 1.17)
Inverse mean FPG	-17.30 (-18.00, -16.60)
Mean age of age group	0.0098 (0.0075, 0.012)
Male sex	0.0020 (-0.0047, 0.0087)
Natural logarithm of per-capita gross domestic product	0.020 (0.014, 0.026)
Study mid-year (per one more recent year since 1980)	0.0014 (0.00096, 0.0019)
Inverse mean FPG * mean age of age group	0.016 (0.0036, 0.029)
Random effects for super-regions	Yes
**Number of data points used to fit the model = 3,722**	

Traditional R^2 is not clearly defined for mixed-effect models. The pseudo-R^2 for the model, which describes the proportion of variance explained by both fixed and random factors,[4] was 0.719.

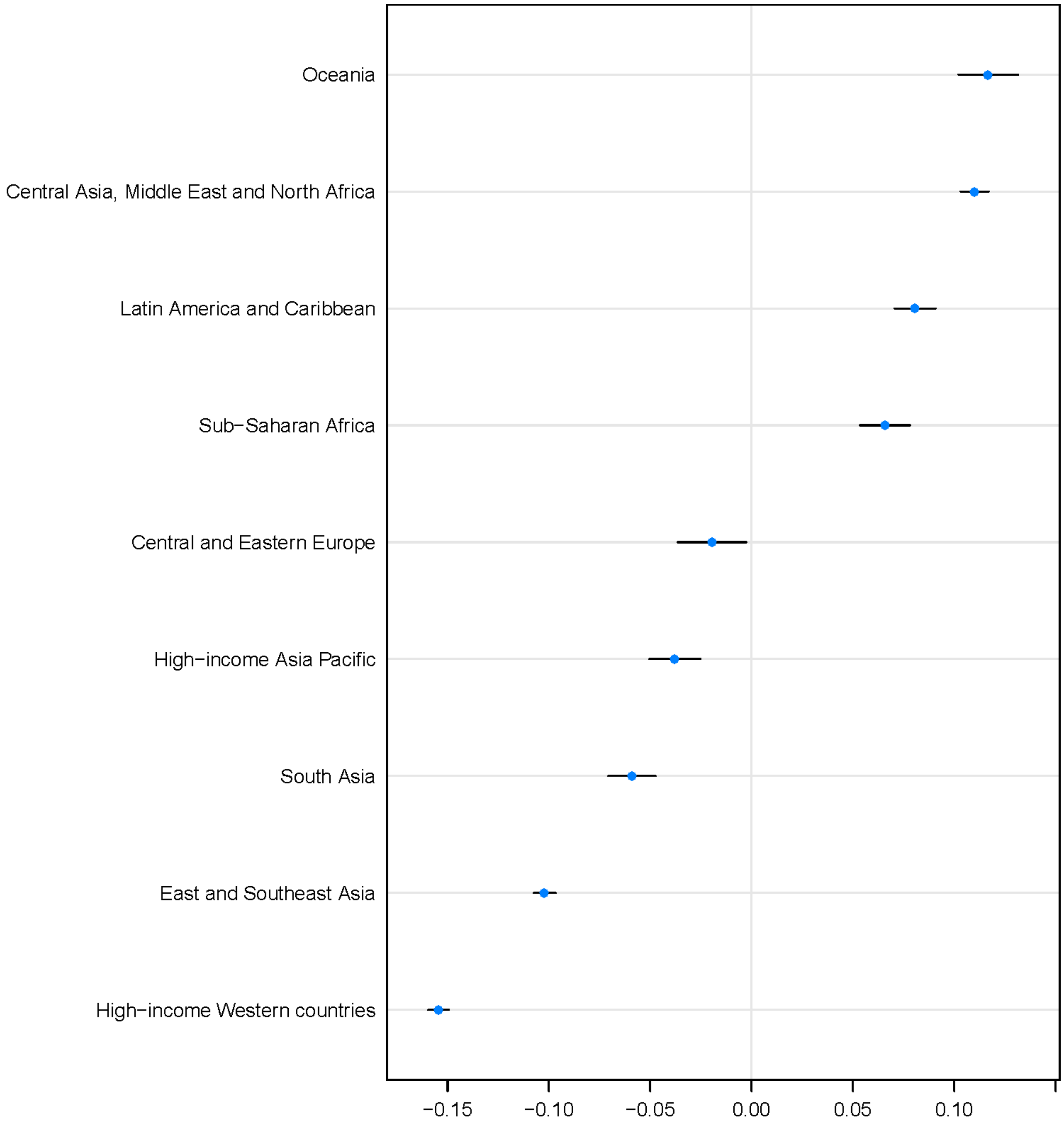

Oceania
Central Asia, Middle East and North Africa
Latin America and Caribbean
Sub−Saharan Africa
Central and Eastern Europe
High−income Asia Pacific
South Asia
East and Southeast Asia
High−income Western countries
−0.15
−0.10
−0.05
0.00
0.05
0.10

Independent variable: diabetes prevalence (FPG ≥6.1 mmol/L)	
**Variables**	**Coefficients (95% CI)**
Intercept	-2.11 (-2.21, -2.00)
Probit-transformed prevalence (FPG ≥6.1 mmol/L)	0.54 (0.51, 0.57)
Mean age of age group	0.013 (0.012, 0.014)
Male sex	-0.025 (-0.040, -0.0098)
Natural logarithm of per-capita gross domestic product	0.082 (0.075, 0.088)
Study mid-year (per one more recent year since 1980)	0.0029 (0.0022, 0.0035)
Probit-transformed prevalence (FPG ≥6.1 mmol/L) * mean age of age group	0.0016 (0.00099, 0.0021)
Probit-transformed prevalence (FPG ≥6.1 mmol/L) * male sex	0.021 (0.0052, 0.036)
Random effects for super-regions	Yes
**Number of data points used to fit the model = 2,483**	

Traditional R^2 is not clearly defined for mixed-effect models. The pseudo-R^2 for the model, which describes the proportion of variance explained by both fixed and random factors,[4] was 0.736.

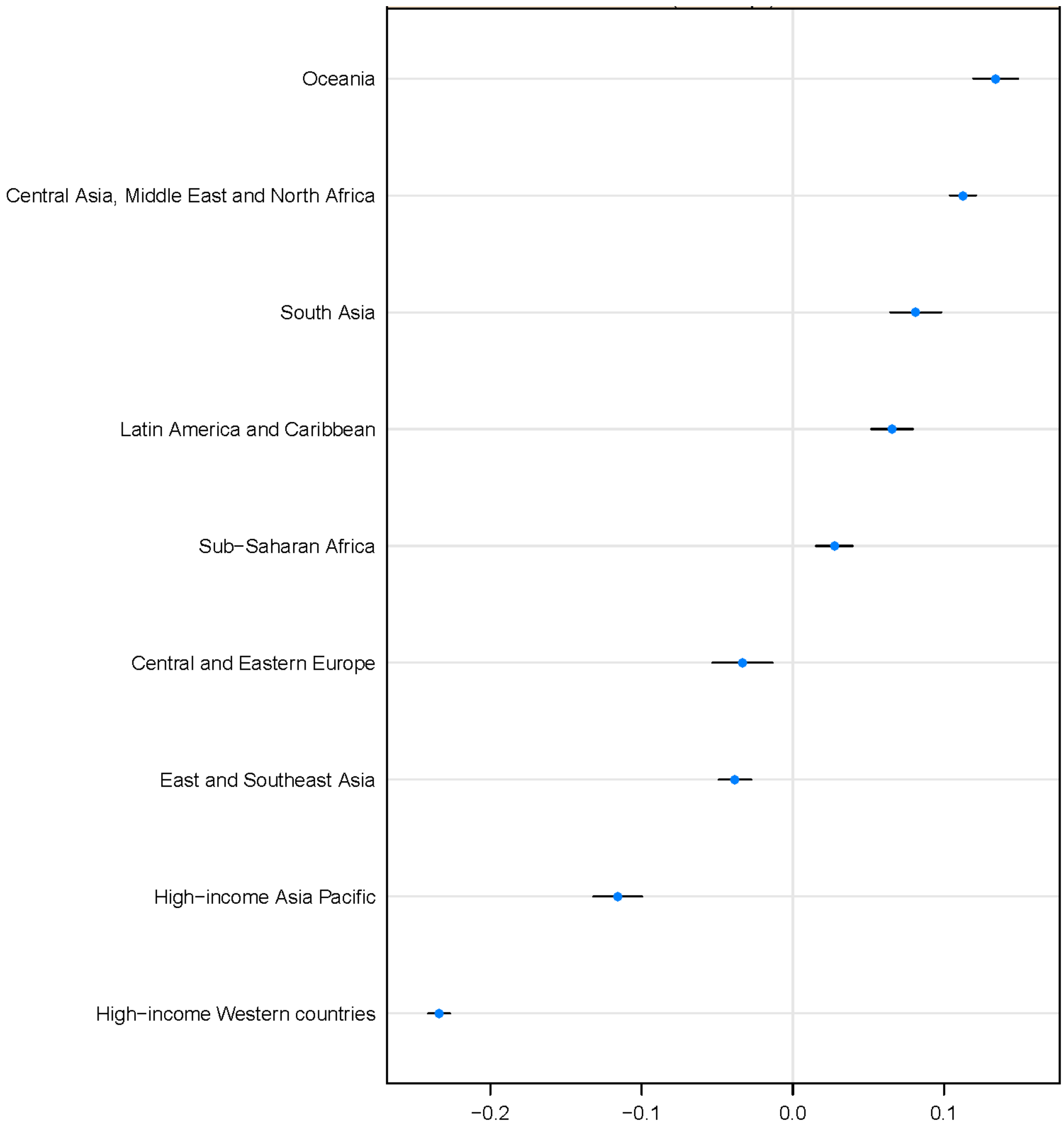
Oceania
Central Asia, Middle East and North Africa
South Asia
Latin America and Caribbean
Sub−Saharan Africa
Central and Eastern Europe
East and Southeast Asia
High−income Asia Pacific
High−income Western countries
−0.2
−0.1
0.0
0.1

Independent variable: diabetes prevalence (FPG ≥6.7 mmol/L)	
**Variables**	**Coefficients (95% CI)**
Intercept	-1.23 (-1.32, -1.13)
Probit-transformed prevalence (FPG ≥6.7 mmol/L)	0.68 (0.65, 0.71)
Mean age of age group	0.010 (0.0094, 0.011)
Male sex	0.021 (-0.0011, 0.043)
Natural logarithm of per-capita gross domestic product	0.049 (0.042, 0.056)
Study mid-year (per one more recent year since 1980)	0.0044 (0.0038, 0.0051)
Probit-transformed prevalence (FPG ≥6.7 mmol/L) * mean age of age group	0.0029 (0.0023, 0.0035)
Probit-transformed prevalence (FPG ≥6.7 mmol/L) * male sex	0.044 (0.027, 0.062)
Random effects for super-regions	Yes
**Number of data points used to fit the model = 2,452**	

Traditional R^2 is not clearly defined for mixed-effect models. The pseudo-R^2 for the model, which describes the proportion of variance explained by both fixed and random factors,[4] was 0.798.

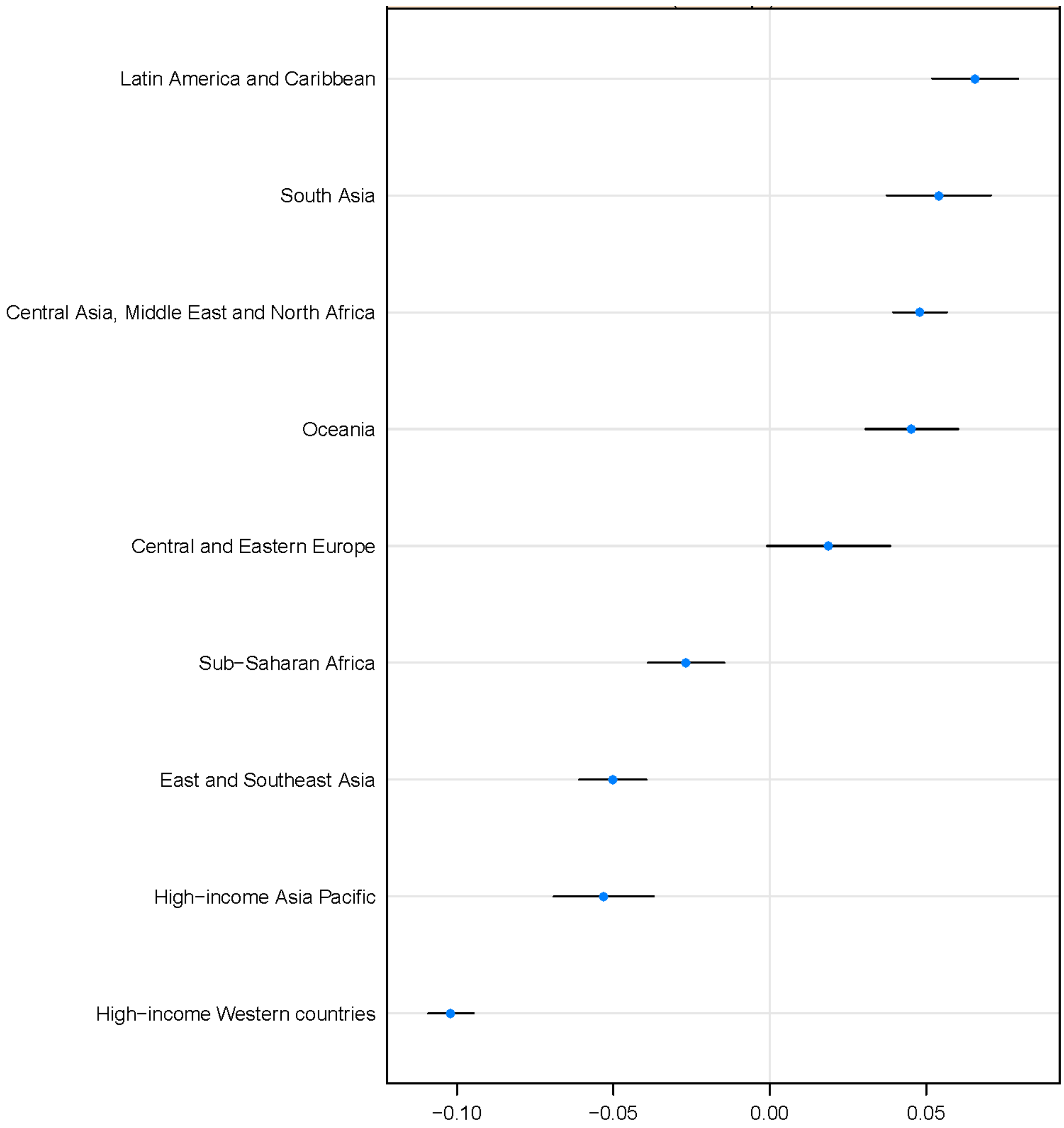

Latin America and Caribbean
South Asia
Central Asia, Middle East and North Africa
Oceania
Central and Eastern Europe
Sub−Saharan Africa
East and Southeast Asia
High−income Asia Pacific
High−income Western countries
−0.10
−0.05
0.00
0.05

Independent variable: diabetes prevalence (FPG ≥6.8 mmol/L)	
**Variables**	**Coefficients (95% CI)**
Intercept	-1.07 (-1.16, -0.97)
Probit-transformed prevalence (FPG ≥6.8 mmol/L)	0.72 (0.69, 0.76)
Mean age of age group	0.0095 (0.0086, 0.010)
Male sex	0.023 (-0.00010, 0.046)
Natural logarithm of per-capita gross domestic product	0.043 (0.036, 0.050)
Study mid-year (per one more recent year since 1980)	0.0045 (0.0039, 0.0052)
Probit-transformed prevalence (FPG ≥6.8 mmol/L) * mean age of age group	0.0026 (0.0020, 0.0033)
Probit-transformed prevalence (FPG ≥6.8 mmol/L) * male sex	0.042 (0.025, 0.060)
Random effects for super-regions	Yes
**Number of data points used to fit the model = 2,449**	

Traditional R^2 is not clearly defined for mixed-effect models. The pseudo-R^2 for the model, which describes the proportion of variance explained by both fixed and random factors,[4] was 0.803.

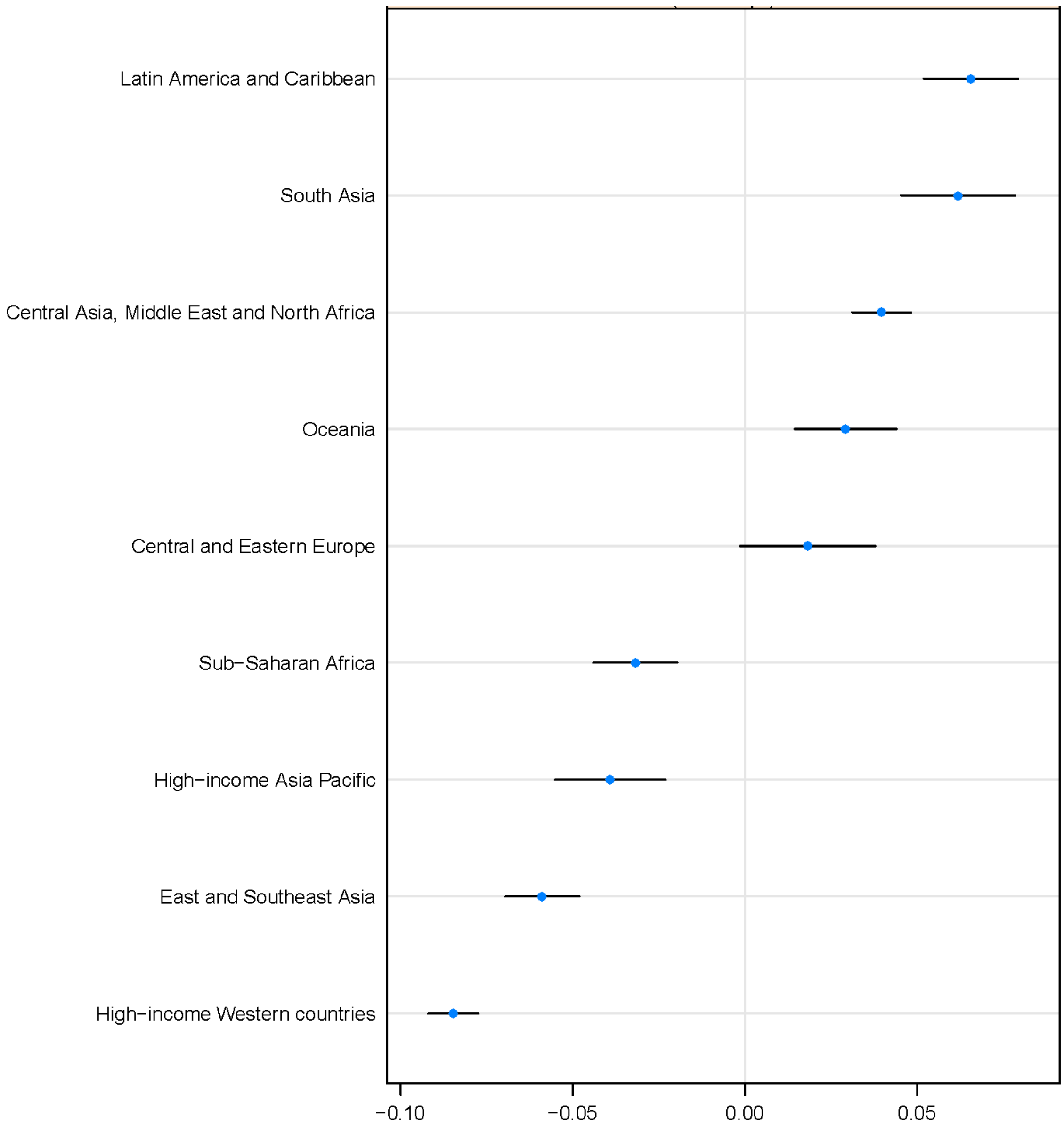
Latin America and Caribbean
South Asia
Central Asia, Middle East and North Africa
Oceania
Central and Eastern Europe
Sub−Saharan Africa
High−income Asia Pacific
East and Southeast Asia
High−income Western countries
−0.10
−0.05
0.00
0.05

Independent variable: diabetes prevalence (FPG ≥7.0 mmol/L)	
**Variables**	**Coefficients (95% CI)**
Intercept	-0.55 (-0.63, -0.47)
Probit-transformed prevalence (FPG ≥7.0 mmol/L)	0.85 (0.83, 0.88)
Mean age of age group	0.0057 (0.0049, 0.0064)
Male sex	0.031 (0.0087, 0.053)
Natural logarithm of per-capita gross domestic product	0.034 (0.028, 0.040)
Study mid-year (per one more recent year since 1980)	-0.000048 (-0.00051, 0.00042)
Probit-transformed prevalence (FPG ≥7.0 mmol/L) * mean age of age group	0.00093 (0.00043, 0.0014)
Probit-transformed prevalence (FPG ≥7.0 mmol/L) * male sex	0.047 (0.030, 0.063)
Random effects for super-regions	Yes
**Number of data points used to fit the model = 3,569**	

Traditional R^2 is not clearly defined for mixed-effect models. The pseudo-R^2 for the model, which describes the proportion of variance explained by both fixed and random factors,[4] was 0.803.

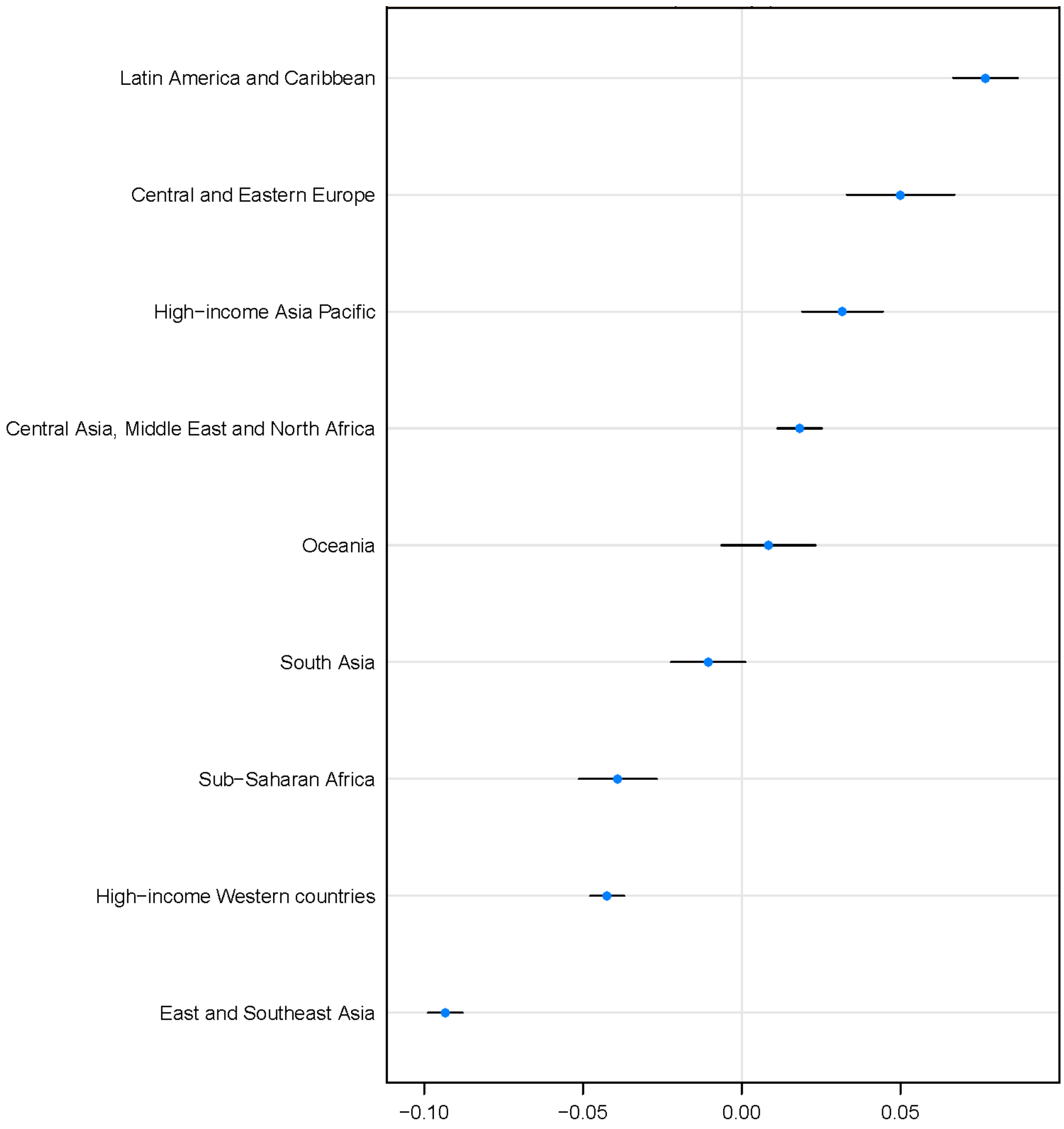

Latin America and Caribbean
Central and Eastern Europe
High−income Asia Pacific
Central Asia, Middle East and North Africa
Oceania
South Asia
Sub−Saharan Africa
High−income Western countries
East and Southeast Asia
−0.10
−0.05
0.00
0.05

Independent variable: diabetes prevalence (FPG ≥7.8 mmol/L)	
**Variables**	**Coefficients (95% CI)**
Intercept	-0.87 (-0.98, -0.76)
Probit-transformed prevalence (FPG ≥7.8 mmol/L)	0.72 (0.68, 0.76)
Mean age of age group	0.011 (0.0098, 0.012)
Male sex	0.041 (0.012, 0.070)
Natural logarithm of per-capita gross domestic product	0.025 (0.018, 0.032)
Study mid-year (per one more recent year since 1980)	0.0085 (0.0078, 0.0091)
Probit-transformed prevalence (FPG ≥7.8 mmol/L) * mean age of age group	0.0021 (0.0015, 0.0028)
Probit-transformed prevalence (FPG ≥7.8 mmol/L) * male sex	0.031 (0.013, 0.050)
Random effects for super-regions	Yes
**Number of data points used to fit the model = 2,324**	

Traditional R^2 is not clearly defined for mixed-effect models. The pseudo-R^2 for the model, which describes the proportion of variance explained by both fixed and random factors, [4] was 0.781.

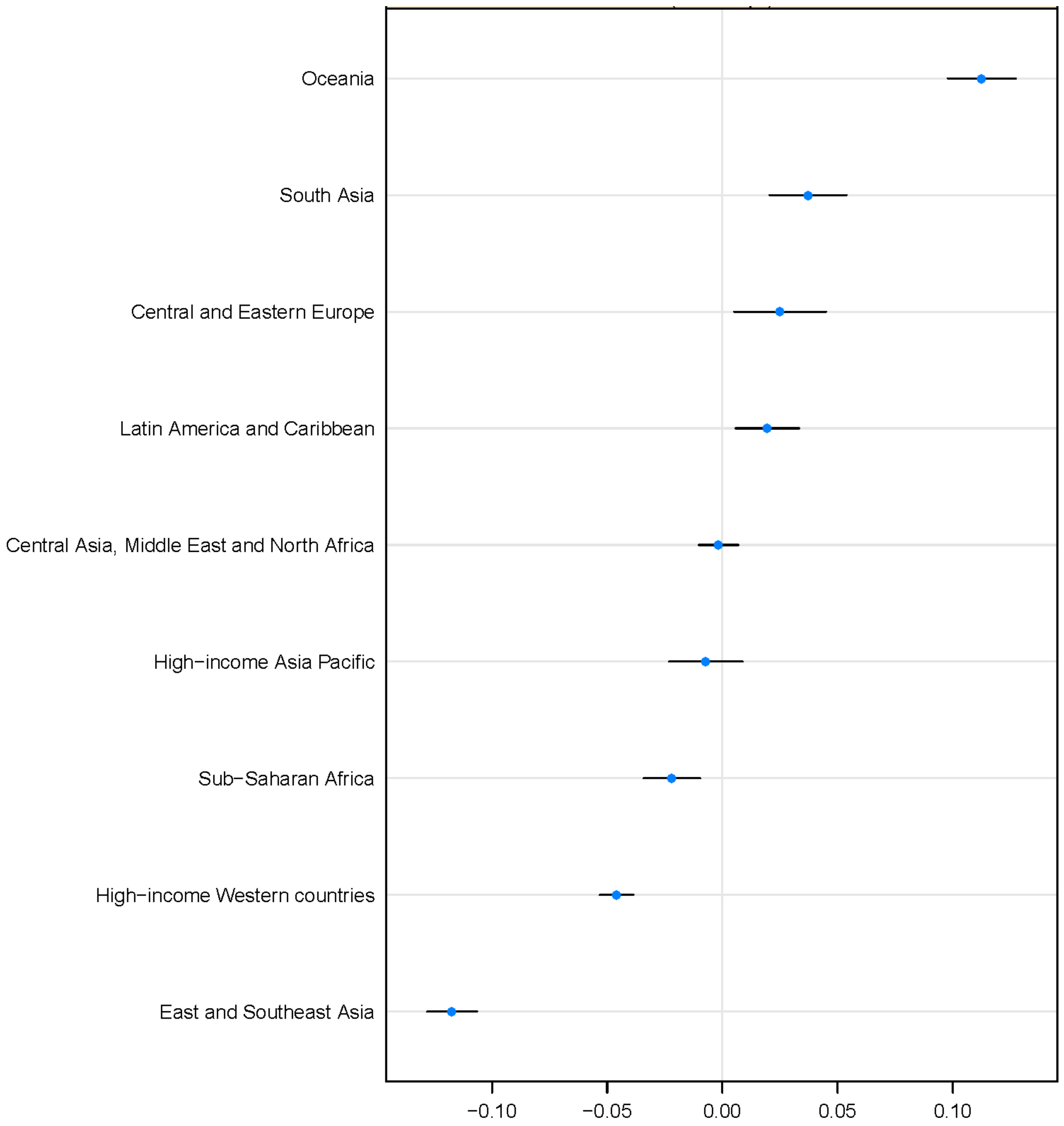
Oceania
South Asia
Central and Eastern Europe
Latin America and Caribbean
Central Asia, Middle East and North Africa
High−income Asia Pacific
Sub−Saharan Africa
High−income Western countries
East and Southeast Asia
−0.10
−0.05
0.00
0.05
0.10

Independent variable: diabetes prevalence (FPG ≥6.0 mmol/L)	
**Variables**	**Coefficients (95% CI)**
Intercept	-2.11 (-2.22, -2.00)
Probit-transformed prevalence (FPG ≥6.0 mmol/L)	0.55 (0.52, 0.58)
Mean age of age group	0.013 (0.012, 0.014)
Male sex	-0.018 (-0.032, -0.0036)
Natural logarithm of per-capita gross domestic product	0.075 (0.068, 0.082)
Study mid-year (per one more recent year since 1980)	0.0029 (0.0022, 0.0035)
Probit-transformed prevalence (FPG ≥6.0 mmol/L) * mean age of age group	0.00080 (0.00025, 0.0014)
Probit-transformed prevalence (FPG ≥6.0 mmol/L) * male sex	0.033 (0.017, 0.048)
Random effects for super-regions	Yes
**Number of data points used to fit the model = 2,484**	

Traditional R^2 is not clearly defined for mixed-effect models. The pseudo-R^2 for the model, which describes the proportion of variance explained by both fixed and random factors, [4] was 0.727.

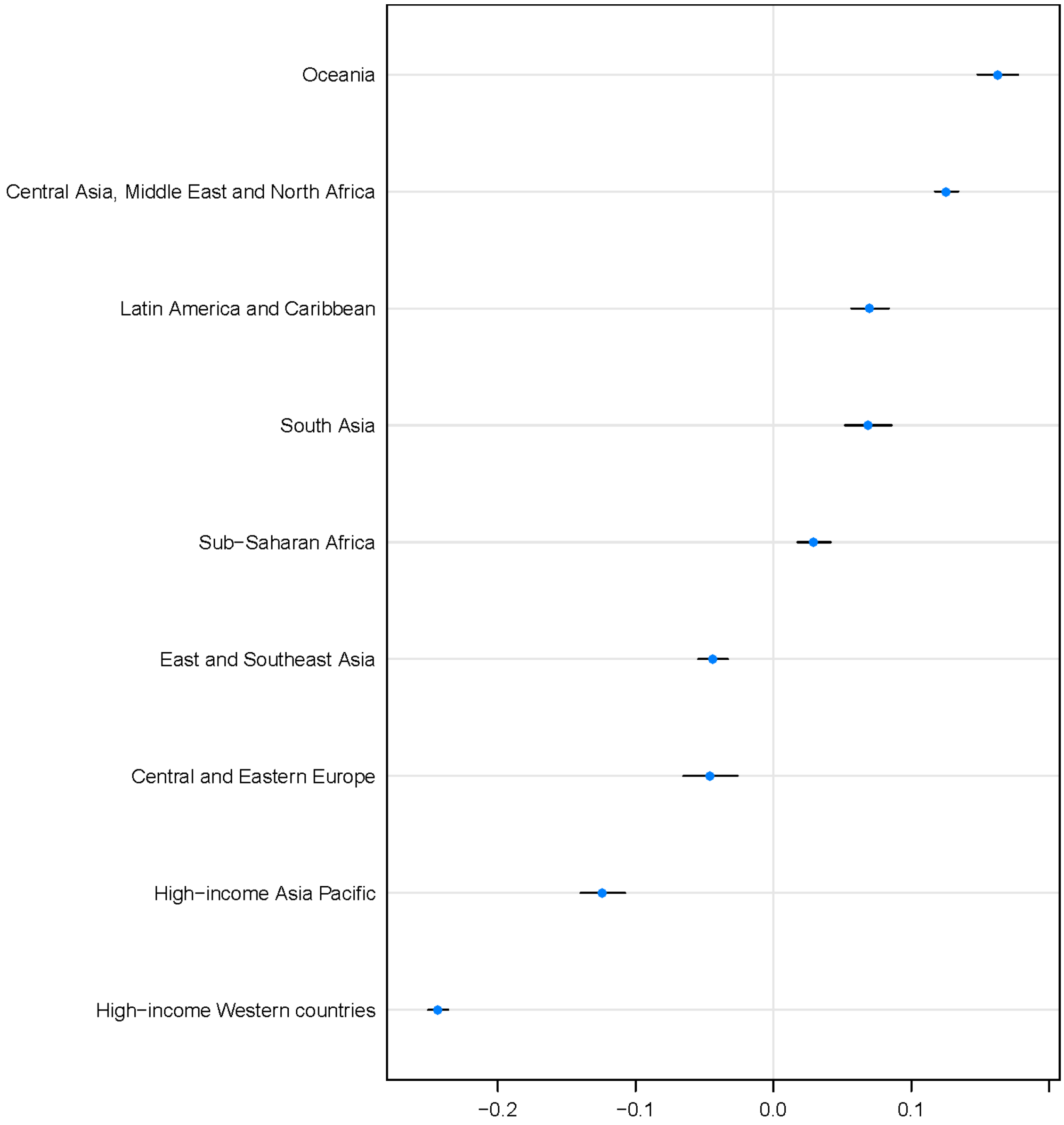
Oceania
Central Asia, Middle East and North Africa
Latin America and Caribbean
South Asia
Sub−Saharan Africa
East and Southeast Asia
Central and Eastern Europe
High−income Asia Pacific
High−income Western countries
−0.2
−0.1
0.0
0.1

Independent variable: diabetes prevalence (FPG ≥5.5 mmol/L or history of diabetes diagnosis or use of diabetes medication)	
**Variables**	**Coefficients (95% CI)**
Intercept	-2.60 (-2.72, -2.47)
Probit-transformed prevalence (FPG ≥5.5 mmol/L or history of diabetes diagnosis or use of diabetes medication)	0.47 (0.44, 0.49)
Mean age of age group	0.016 (0.016, 0.016)
Male sex	-0.028 (-0.036, -0.021)
Natural logarithm of per-capita gross domestic product	0.070 (0.063, 0.076)
Study mid-year (per one more recent year since 1980)	0.0029 (0.0022, 0.0035)
Probit-transformed prevalence (FPG ≥5.5 mmol/L or history of diabetes diagnosis or use of diabetes medication) * mean age of age group	-0.00083 (-0.0013, -0.00037)
Random effects for super-regions	Yes
**Number of data points used to fit the model = 2,529**	

Traditional R^2 is not clearly defined for mixed-effect models. The pseudo-R^2 for the model, which describes the proportion of variance explained by both fixed and random factors,[4] was 0.673.

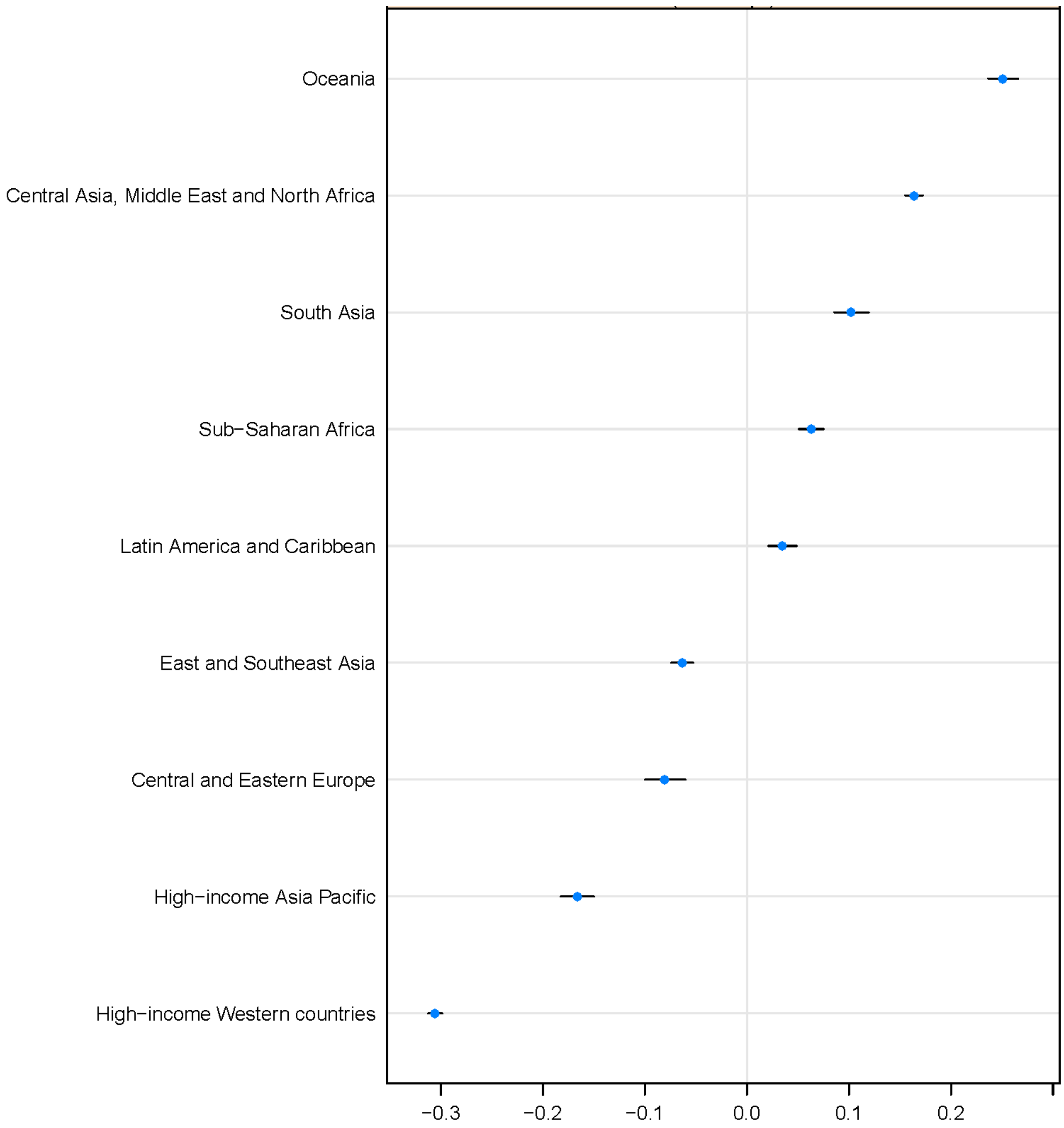
Oceania
Central Asia, Middle East and North Africa
South Asia
Sub−Saharan Africa
Latin America and Caribbean
East and Southeast Asia
Central and Eastern Europe
High−income Asia Pacific
High−income Western countries
−0.3
−0.2
−0.1
0.0
0.1
0.2

Independent variable: diabetes prevalence (FPG ≥5.6 mmol/L or history of diabetes diagnosis or use of diabetes medication)	
**Variables**	**Coefficients (95% CI)**
Intercept	-2.54 (-2.66, -2.42)
Probit-transformed prevalence (FPG ≥5.6 mmol/L or history of diabetes diagnosis or use of diabetes medication)	0.46 (0.45, 0.47)
Mean age of age group	0.015 (0.015, 0.016)
Male sex	-0.033 (-0.040, -0.025)
Natural logarithm of per-capita gross domestic product	0.074 (0.068, 0.081)
Study mid-year (per one more recent year since 1980)	0.0030 (0.0024, 0.0036)
Random effects for super-regions	Yes
**Number of data points used to fit the model = 2,532**	

Traditional R^2 is not clearly defined for mixed-effect models. The pseudo-R^2 for the model, which describes the proportion of variance explained by both fixed and random factors,[4] was 0.688.

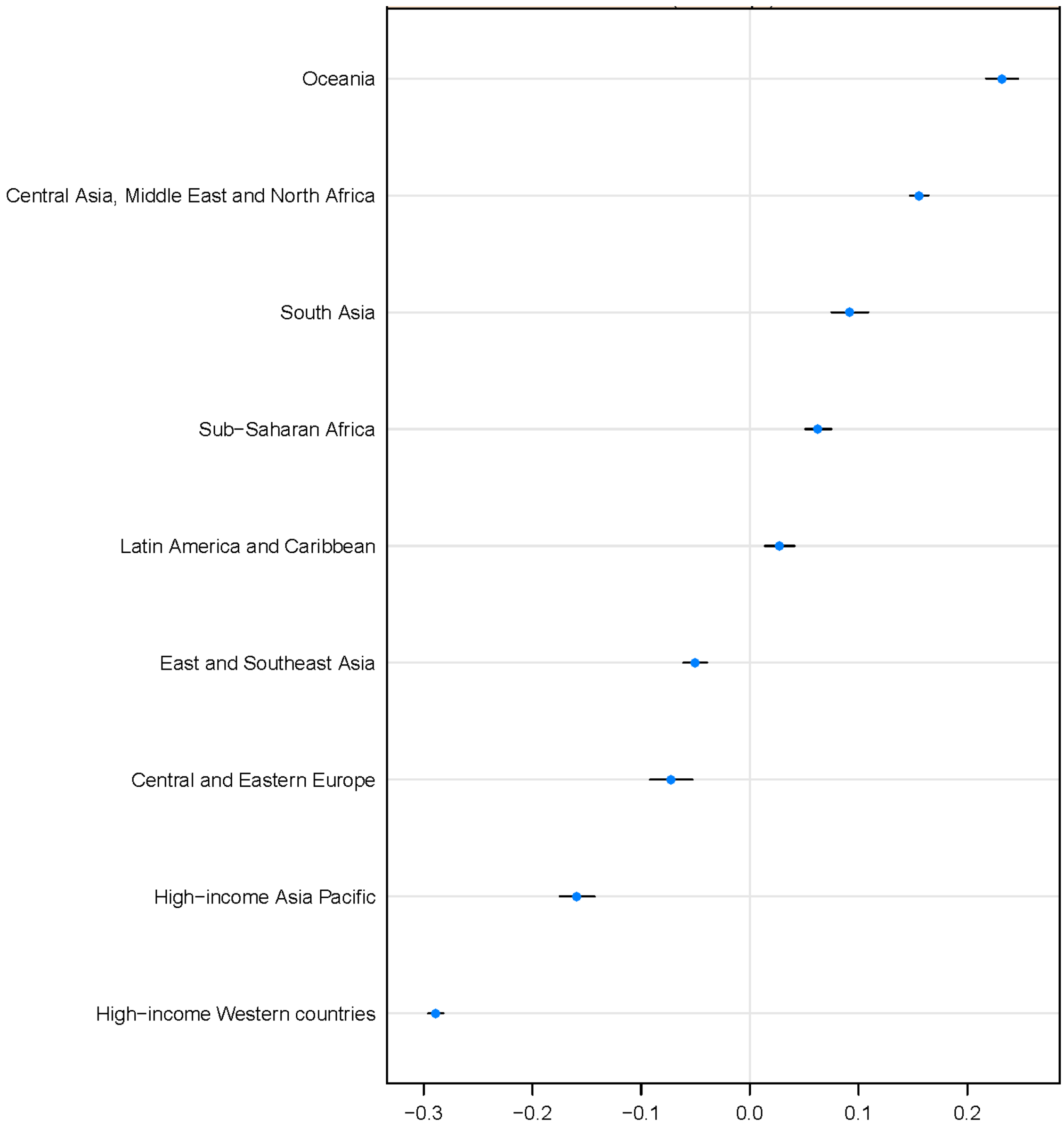
Oceania
Central Asia, Middle East and North Africa
South Asia
Sub−Saharan Africa
Latin America and Caribbean
East and Southeast Asia
Central and Eastern Europe
High−income Asia Pacific
High−income Western countries
−0.3
−0.2
−0.1
0.0
0.1
0.2

Independent variable: diabetes prevalence (FPG ≥6.1 mmol/L or history of diabetes diagnosis or use of diabetes medication)	
**Variables**	**Coefficients (95% CI)**
Intercept	-1.73 (-1.83, -1.64)
Probit-transformed prevalence (FPG ≥6.1 mmol/L or history of diabetes diagnosis or use of diabetes medication)	0.64 (0.61, 0.66)
Mean age of age group	0.0099 (0.0093, 0.010)
Male sex	-0.034 (-0.042, -0.027)
Natural logarithm of per-capita gross domestic product	0.065 (0.058, 0.072)
Study mid-year (per one more recent year since 1980)	0.00084 (0.00021, 0.0015)
Probit-transformed prevalence (FPG ≥6.1 mmol/L or history of diabetes diagnosis or use of diabetes medication) * mean age of age group	0.0014 (0.00083, 0.0019)
Random effects for super-regions	Yes
**Number of data points used to fit the model = 2,533**	

Traditional R^2 is not clearly defined for mixed-effect models. The pseudo-R^2 for the model, which describes the proportion of variance explained by both fixed and random factors,[4] was 0.765.

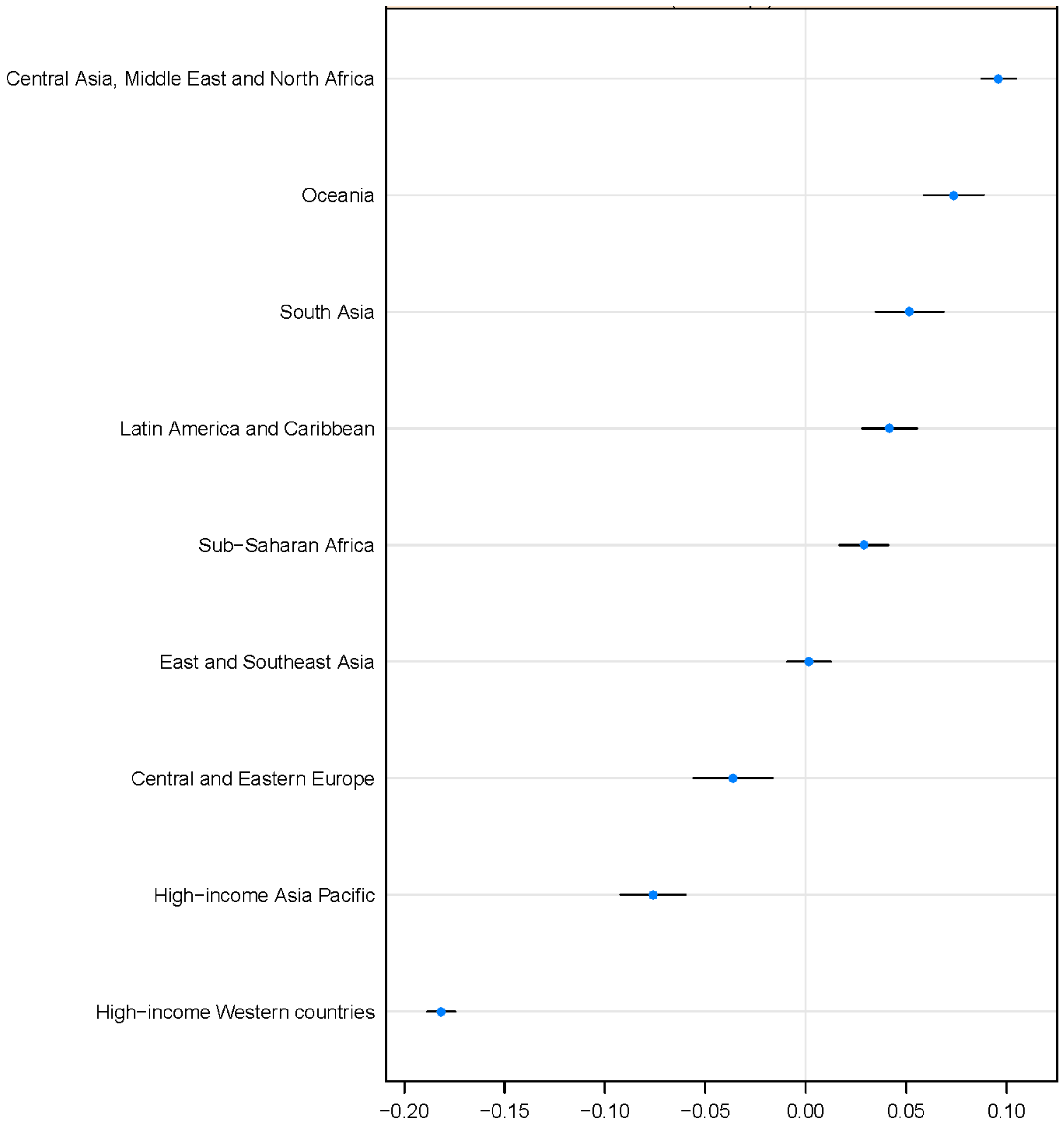

Central Asia, Middle East and North Africa
Oceania
South Asia
Latin America and Caribbean
Sub−Saharan Africa
East and Southeast Asia
Central and Eastern Europe
High−income Asia Pacific
High−income Western countries
−0.20
−0.15
−0.10
−0.05
0.00
0.05
0.10

Independent variable: diabetes prevalence (FPG ≥6.8 mmol/L or history of diabetes diagnosis or use of diabetes medication)	
**Variables**	**Coefficients (95% CI)**
Intercept	-0.22 (-0.30, -0.15)
Probit-transformed prevalence (FPG ≥6.8 mmol/L or history of diabetes diagnosis or use of diabetes medication)	0.95 (0.92, 0.98)
Mean age of age group	0.0015 (0.00068, 0.0022)
Male sex	-0.0071 (-0.015, 0.00063)
Natural logarithm of per-capita gross domestic product	0.0089 (0.0031, 0.015)
Study mid-year (per one more recent year since 1980)	-0.00023 (-0.00087, 0.00040)
Probit-transformed prevalence (FPG ≥6.8 mmol/L or history of diabetes diagnosis or use of diabetes medication) * mean age of age group	0.00060 (0.0000062, 0.0012)
Random effects for super-regions	Yes
**Number of data points used to fit the model = 2,533**	

Traditional R^2 is not clearly defined for mixed-effect models. The pseudo-R^2 for the model, which describes the proportion of variance explained by both fixed and random factors,[4] was 0.848.

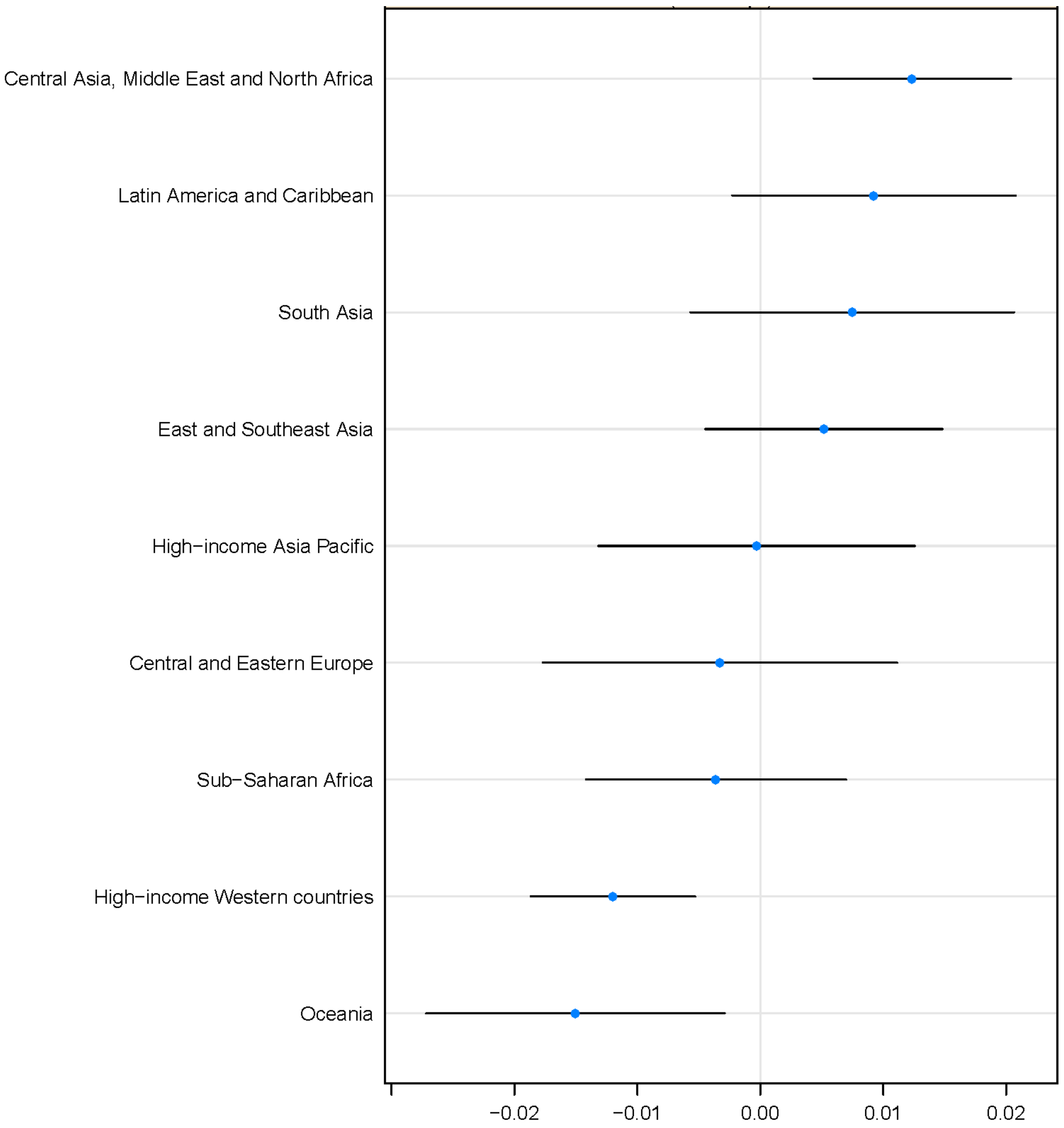
Central Asia, Middle East and North Africa
Latin America and Caribbean
South Asia
East and Southeast Asia
High−income Asia Pacific
Central and Eastern Europe
Sub−Saharan Africa
High−income Western countries
Oceania
−0.02
−0.01
0.00
0.01
0.02

Independent variable: mean 2hOGTT	
**Variables**	**Coefficients (95% CI)**
Intercept	0.47 (0.10, 0.83)
Inverse mean 2hOGTT	-24.30 (-25.90, -22.70)
Mean age of age group	-0.020 (-0.024, -0.016)
Male sex	0.15 (0.13, 0.17)
Natural logarithm of per-capita gross domestic product	0.092 (0.073, 0.11)
Study mid-year (per one more recent year since 1980)	0.017 (0.016, 0.018)
Inverse mean 2hOGTT * mean age of age group	0.24 (0.21, 0.27)
Random effects for super-regions	Yes
**Number of data points used to fit the model = 614**	

Traditional R^2 is not clearly defined for mixed-effect models. The pseudo-R^2 for the model, which describes the proportion of variance explained by both fixed and random factors,[4] was 0.710.

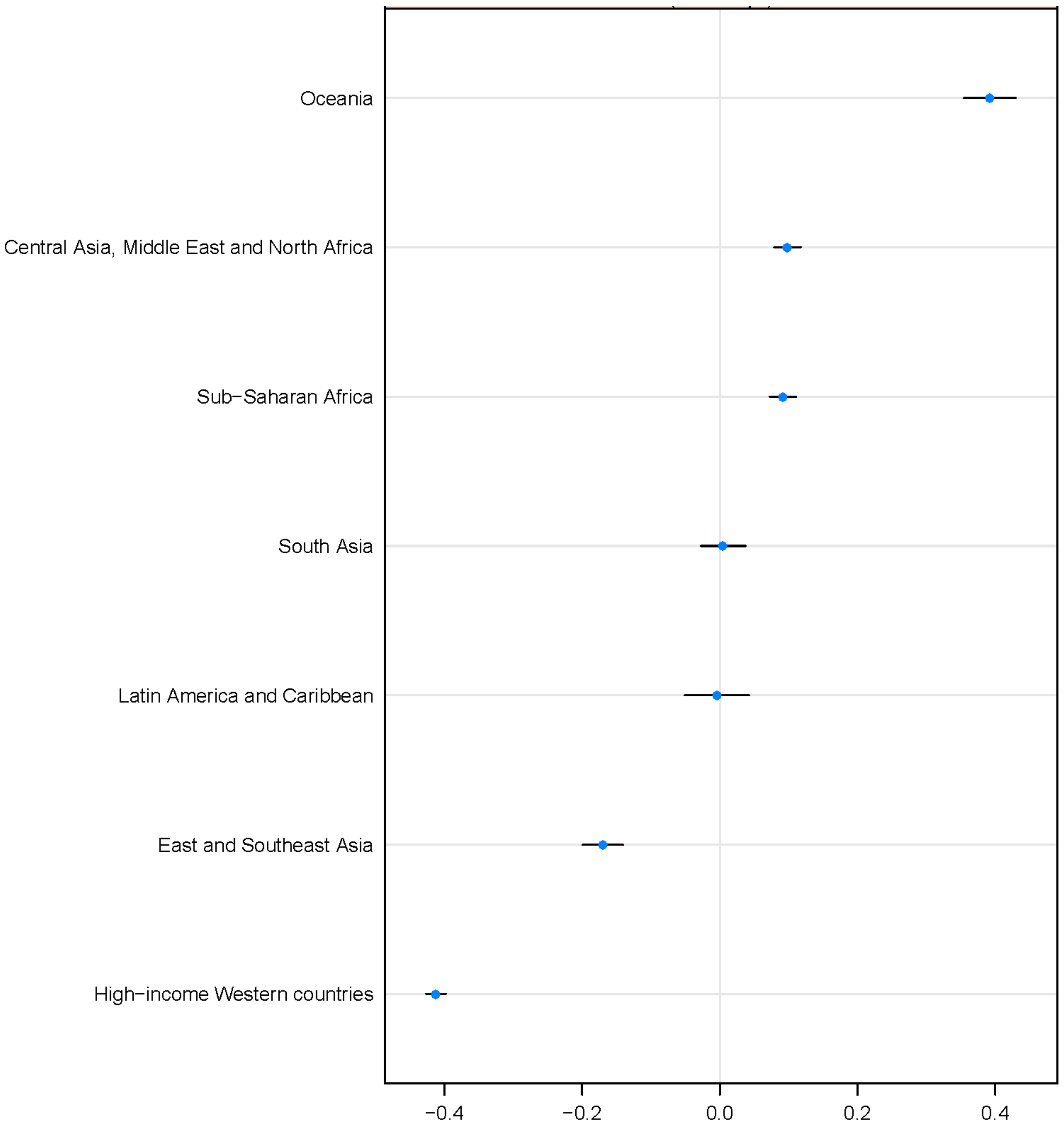
Oceania
Central Asia, Middle East and North Africa
Sub−Saharan Africa
South Asia
Latin America and Caribbean
East and Southeast Asia
High−income Western countries
−0.4
−0.2
0.0
0.2
0.4

Independent variable: diabetes prevalence (2hOGTT ≥11.1 mmol/L)	
**Variables**	**Coefficients (95% CI)**
Intercept	-1.50 (-2.41, -0.59)
Probit-transformed prevalence (2hOGTT ≥11.1 mmol/L)	0.81 (0.68, 0.95)
Mean age of age group	0.0068 (0.00094, 0.013)
Male sex	0.056 (-0.0054, 0.12)
Natural logarithm of per-capita gross domestic product	0.11 (0.048, 0.18)
Study mid-year (per one more recent year since 1980)	0.0073 (0.0044, 0.010)
Random effects for super-regions	No
**Number of data points used to fit the model = 57**	

Traditional R^2 is not clearly defined for generalised linear models. The pseudo-R^2 for the model, which describes the proportion of variance explained by the model,[4] was 0.817.

Independent variable: diabetes prevalence (FPG ≥7.0 mmol/L or 2hOGTT ≥11.1 mmol/L)	
**Variables**	**Coefficients (95% CI)**
Intercept	-0.89 (-1.78, -0.0027)
Probit-transformed prevalence (FPG ≥7.0 mmol/L or 2hOGTT ≥11.1 mmol/L)	1.00 (0.85, 1.14)
Mean age of age group	0.0024 (-0.0035, 0.0083)
Male sex	0.0050 (-0.058, 0.068)
Natural logarithm of per-capita gross domestic product	0.074 (0.010, 0.14)
Study mid-year (per one more recent year since 1980)	0.010 (0.0070, 0.013)
Random effects for super-regions	No
**Number of data points used to fit the model = 57**	

Traditional R^2 is not clearly defined for generalised linear models. The pseudo-R^2 for the model, which describes the proportion of variance explained by the model,[4] was 0.849.

Independent variable: diabetes prevalence (FPG ≥7.8 mmol/L or 2hOGTT ≥11.1 mmol/L)	
**Variables**	**Coefficients (95% CI)**
Intercept	-1.12 (-2.03, -0.21)
Probit-transformed prevalence (FPG ≥7.8 mmol/L or 2hOGTT ≥11.1 mmol/L)	0.90 (0.76, 1.04)
Mean age of age group	0.0036 (-0.0025, 0.0096)
Male sex	0.034 (-0.028, 0.096)
Natural logarithm of per-capita gross domestic product	0.091 (0.026, 0.16)
Study mid-year (per one more recent year since 1980)	0.0083 (0.0053, 0.011)
Random effects for super-regions	No
**Number of data points used to fit the model = 57**	

Traditional R^2 is not clearly defined for generalised linear models. The pseudo-R^2 for the model, which describes the proportion of variance explained by the model,[4] was 0.831.

Independent variable: diabetes prevalence (2hOGTT ≥11.1 mmol/L or history of diabetes diagnosis or use of diabetes medication)	
**Variables**	**Coefficients (95% CI)**
Intercept	-0.14 (-0.35, 0.076)
Probit-transformed prevalence (2hOGTT ≥11.1 mmol/L or history of diabetes diagnosis or use of diabetes medication)	1.00 (0.95, 1.06)
Mean age of age group	-0.0036 (-0.0051, -0.0020)
Male sex	0.10 (0.063, 0.14)
Natural logarithm of per-capita gross domestic product	0.0057 (-0.013, 0.024)
Study mid-year (per one more recent year since 1980)	0.0056 (0.0043, 0.0069)
Probit-transformed prevalence (2hOGTT ≥11.1 mmol/L or history of diabetes diagnosis or use of diabetes medication) * mean age of age group	-0.0024 (-0.0034, -0.0014)
Probit-transformed prevalence (2hOGTT ≥11.1 mmol/L or history of diabetes diagnosis or use of diabetes medication) * male sex	0.068 (0.033, 0.10)
Random effects for super-regions	Yes
**Number of data points used to fit the model = 694**	

Traditional R^2 is not clearly defined for mixed-effect models. The pseudo-R^2 for the model, which describes the proportion of variance explained by both fixed and random factors,[4] was 0.821.

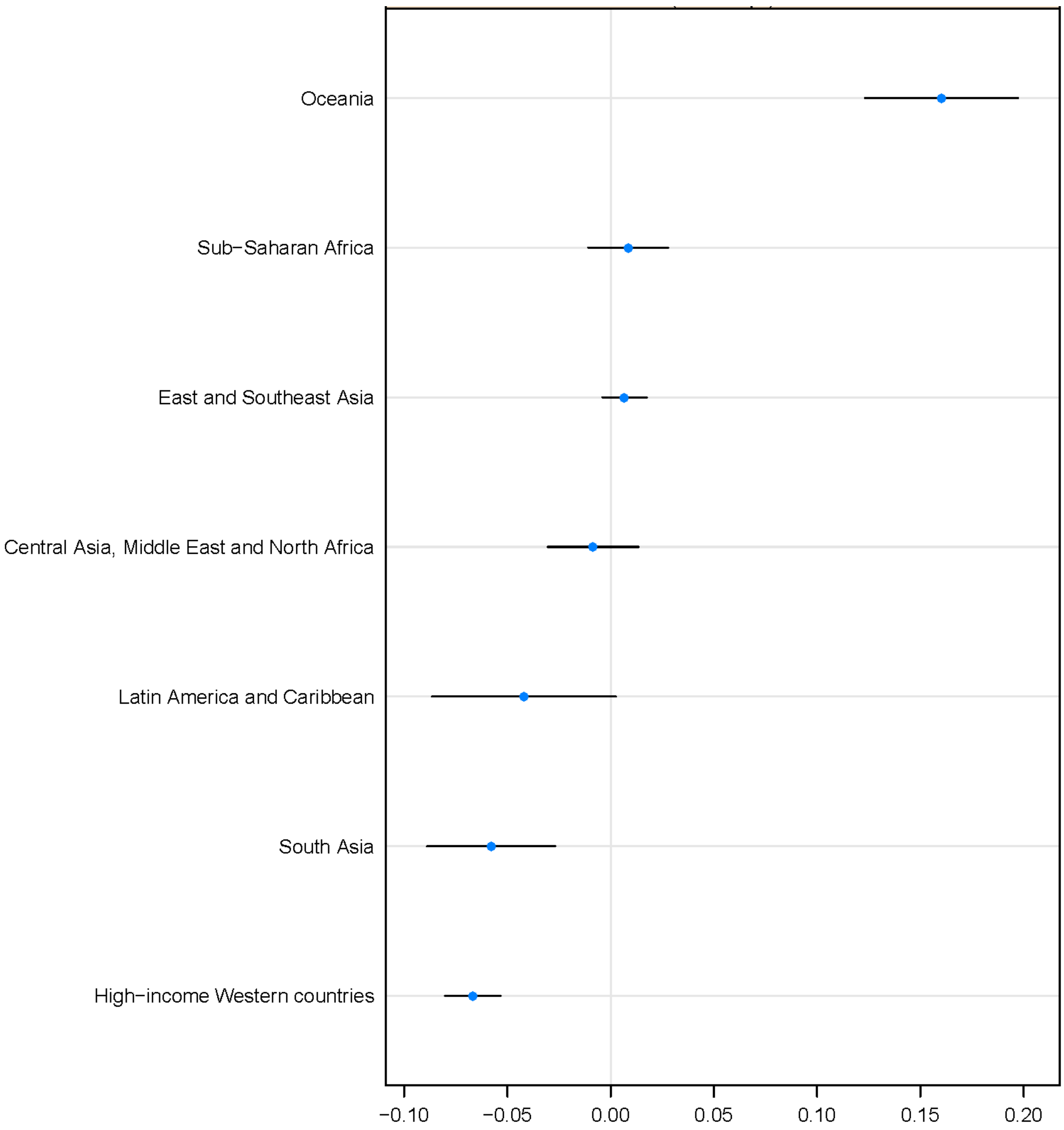

Oceania
Sub−Saharan Africa
East and Southeast Asia
Central Asia, Middle East and North Africa
Latin America and Caribbean
South Asia
High−income Western countries
−0.10
−0.05
0.00
0.05
0.10
0.15
0.20

Independent variable: diabetes prevalence (FPG ≥7.0 mmol/L or 2hOGTT ≥11.1 mmol/L or history of diabetes diagnosis or use of diabetes medication)	
**Variables**	**Coefficients (95% CI)**
Intercept	0.12 (-0.057, 0.29)
Probit-transformed prevalence (FPG ≥7.0 mmol/L or 2hOGTT ≥11.1 mmol/L or history of diabetes diagnosis or use of diabetes medication)	1.12 (1.06, 1.18)
Mean age of age group	-0.0050 (-0.0064, -0.0035)
Male sex	0.017 (-0.0014, 0.035)
Natural logarithm of per-capita gross domestic product	-0.0048 (-0.020, 0.011)
Study mid-year (per one more recent year since 1980)	0.0042 (0.0030, 0.0054)
Probit-transformed prevalence (FPG ≥7.0 mmol/L or 2hOGTT ≥11.1 mmol/L or history of diabetes diagnosis or use of diabetes medication) * mean age of age group	-0.0021 (-0.0031, -0.00099)
Random effects for super-regions	Yes
**Number of data points used to fit the model = 688**	

Traditional R^2 is not clearly defined for generalised linear models. The pseudo-R^2 for the model, which describes the proportion of variance explained by the model,[4] was 0.844.

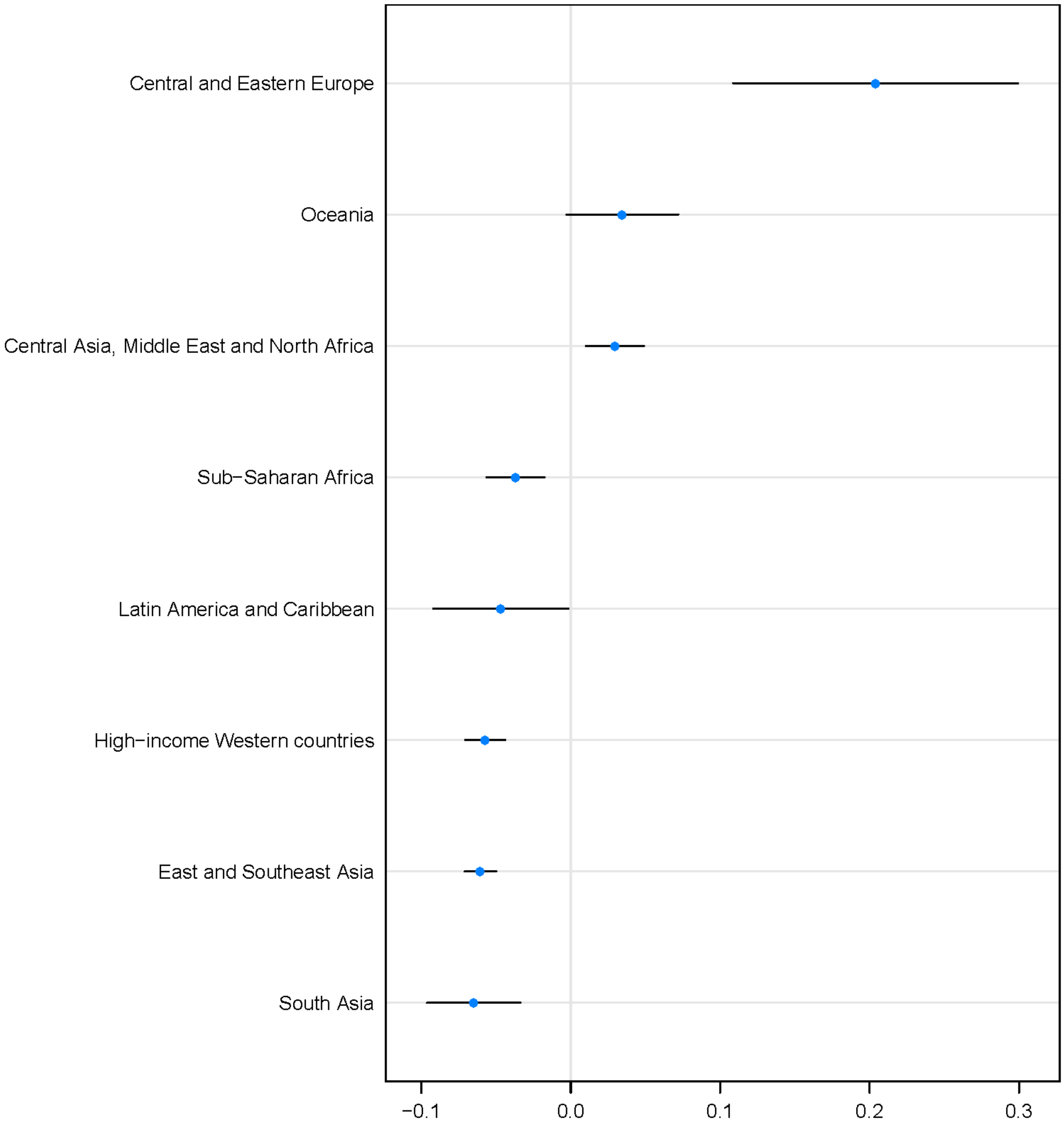

Central and Eastern Europe
Oceania
Central Asia, Middle East and North Africa
Sub−Saharan Africa
Latin America and Caribbean
High−income Western countries
East and Southeast Asia
South Asia
−0.1
0.0
0.1
0.2
0.3

Independent variable: diabetes prevalence (FPG ≥7.8 mmol/L or 2hOGTT ≥11.1 mmol/L or history of diabetes diagnosis or use of diabetes medication)	
**Variables**	**Coefficients (95% CI)**
Intercept	0.080 (-0.15, 0.31)
Probit-transformed prevalence (FPG ≥7.8 mmol/L or 2hOGTT ≥11.1 mmol/L or history of diabetes diagnosis or use of diabetes medication)	1.11 (1.04, 1.19)
Mean age of age group	-0.0052 (-0.0071, -0.0033)
Male sex	0.056 (0.037, 0.076)
Natural logarithm of per-capita gross domestic product	0.0014 (-0.018, 0.020)
Study mid-year (per one more recent year since 1980)	0.0046 (0.0031, 0.0061)
Probit-transformed prevalence (FPG ≥7.8 mmol/L or 2hOGTT ≥11.1 mmol/L or history of diabetes diagnosis or use of diabetes medication) * mean age of age group	-0.0020 (-0.0034, -0.00060)
Random effects for super-regions	Yes
**Number of data points used to fit the model = 476**	

Traditional R^2 is not clearly defined for mixed-effect models. The pseudo-R^2 for the model, which describes the proportion of variance explained by both fixed and random factors,[4] was 0.825.

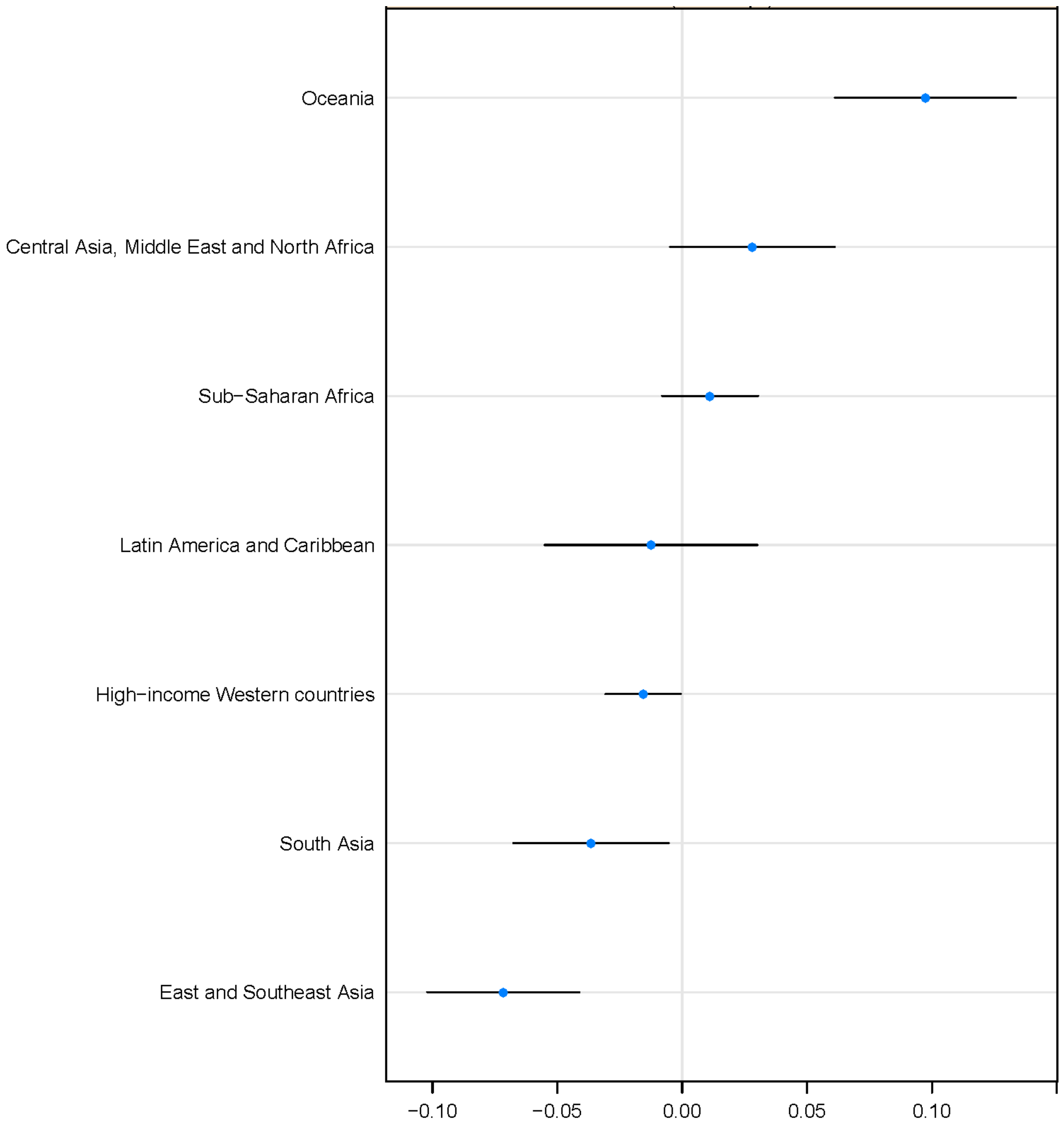
Oceania
Central Asia, Middle East and North Africa
Sub−Saharan Africa
Latin America and Caribbean
High−income Western countries
South Asia
East and Southeast Asia
−0.10
−0.05
0.00
0.05
0.10

Independent variable: mean HbA1c	
**Variables**	**Coefficients (95% CI)**
Intercept	-4.93 (-5.62, -4.23)
Inverse mean HbA1c	-6.02 (-8.45, -3.60)
Mean age of age group	0.0079 (9.0e-05, 0.016)
Male sex	0.12 (0.10, 0.14)
Natural logarithm of per-capita gross domestic product	0.38 (0.34, 0.42)
Study mid-year (per one more recent year since 2000)	-0.00093 (-0.0036, 0.0017)
Inverse mean HbA1c * mean age of age group	0.085 (0.041, 0.13)
Random effects for super-regions	Yes
**Number of data points used to fit the model = 497**	

Traditional R^2 is not clearly defined for mixed-effect models. The pseudo-R^2 for the model, which describes the proportion of variance explained by both fixed and random factors,[4] was 0.554.

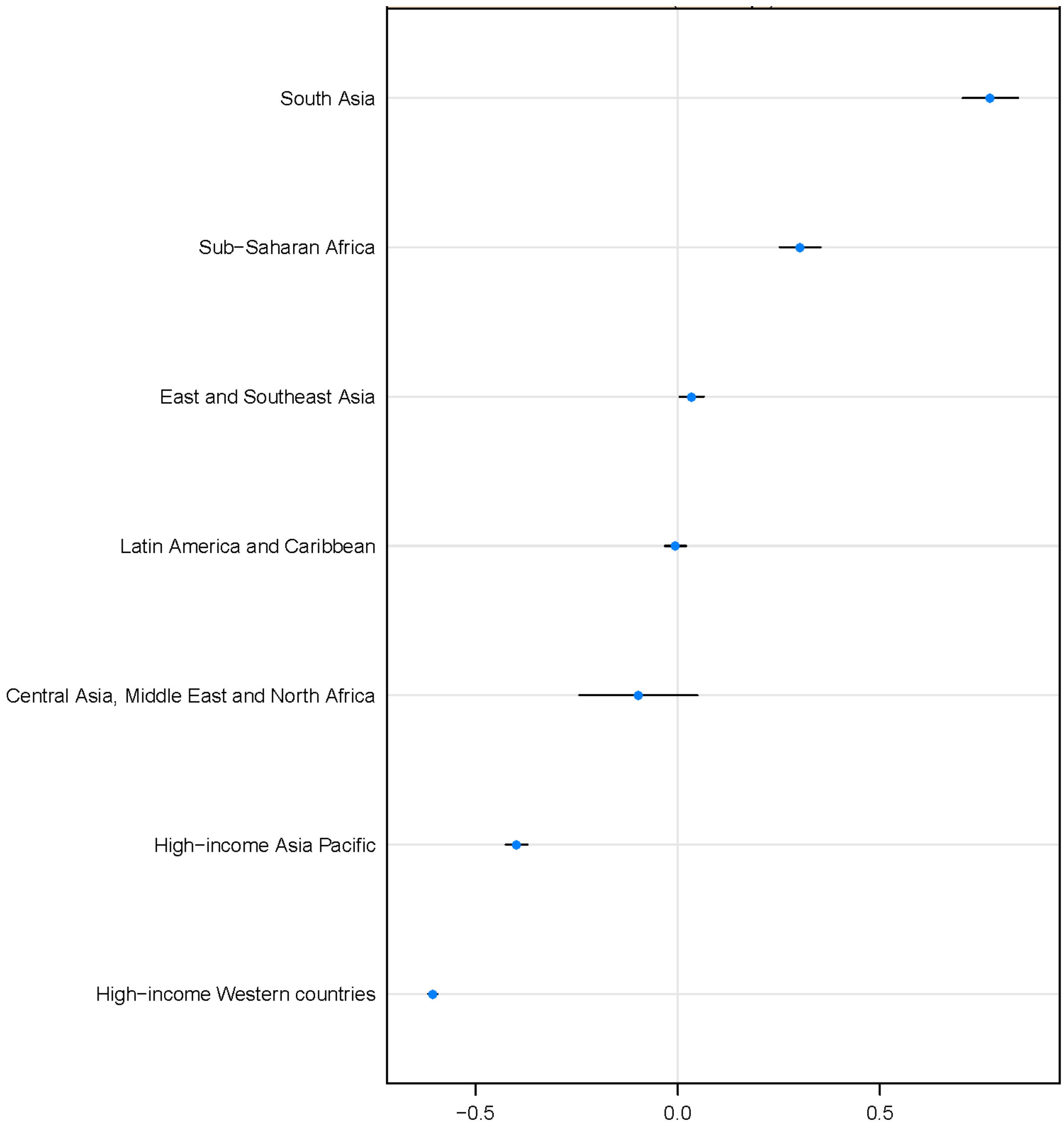

South Asia
Sub−Saharan Africa
East and Southeast Asia
Latin America and Caribbean
Central Asia, Middle East and North Africa
High−income Asia Pacific
High−income Western countries
−0.5
0.0
0.5

Independent variable: diabetes prevalence (HbA1c ≥10% or history of diabetes diagnosis or use of diabetes medication)	
**Variables**	**Coefficients (95% CI)**
Intercept	-1.18 (-1.65, -0.70)
Probit-transformed prevalence (HbA1c ≥10% or history of diabetes diagnosis or use of diabetes medication)	0.90 (0.81, 0.98)
Mean age of age group	0.0023 (-0.000023, 0.0047)
Male sex	0.062 (0.043, 0.080)
Natural logarithm of per-capita gross domestic product	0.11 (0.070, 0.15)
Study mid-year (per one more recent year since 2000)	-0.0033 (-0.0059, -0.00062)
Probit-transformed prevalence (HbA1c ≥10% or history of diabetes diagnosis or use of diabetes medication) * mean age of age group	-0.0017 (-0.0031, -0.00020)
Random effects for super-regions	Yes
**Number of data points used to fit the model = 481**	

Traditional R^2 is not clearly defined for mixed-effect models. The pseudo-R^2 for the model, which describes the proportion of variance explained by both fixed and random factors,[4] was 0.768.

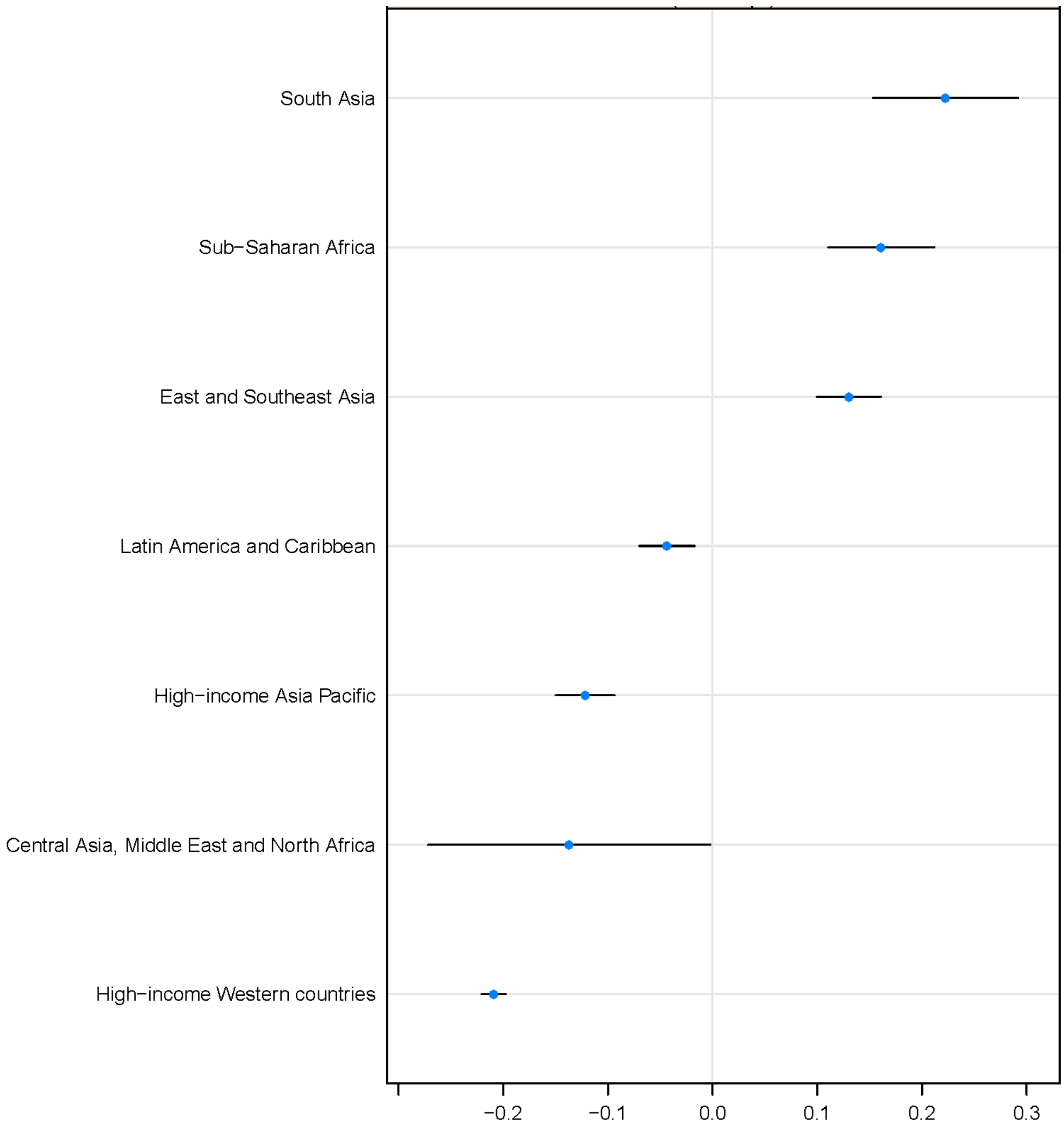
South Asia
Sub−Saharan Africa
East and Southeast Asia
Latin America and Caribbean
High−income Asia Pacific
Central Asia, Middle East and North Africa
High−income Western countries
−0.2
−0.1
0.0
0.1
0.2
0.3

Independent variable: diabetes prevalence (FPG ≥7.0 mmol/L or 2hOGTT ≥11.1 mmol/L or HbA1c ≥10% or history of diabetes diagnosis or use of diabetes medication)	
**Variables**	**Coefficients (95% CI)**
Intercept	-0.70 (-0.98, -0.42)
Probit-transformed prevalence (FPG ≥7.0 mmol/L or 2hOGTT ≥11.1 mmol/L or HbA1c ≥10% or history of diabetes diagnosis or use of diabetes medication)	1.08 (0.92, 1.23)
Mean age of age group	-0.0037 (-0.0069, -0.00049)
Male sex	0.13 (0.056, 0.21)
Natural logarithm of per-capita gross domestic product	0.043 (0.010, 0.077)
Study mid-year (per one more recent year since 2000)	0.0036 (-0.0013, 0.0085)
Probit-transformed prevalence (FPG ≥7.0 mmol/L or 2hOGTT ≥11.1 mmol/L or HbA1c ≥10% or history of diabetes diagnosis or use of diabetes medication) * mean age of age group	-0.0046 (-0.0071, -0.0021)
Probit-transformed prevalence (FPG ≥7.0 mmol/L or 2hOGTT ≥11.1 mmol/L or HbA1c ≥10% or history of diabetes diagnosis or use of diabetes medication) * male sex	0.074 (0.0012, 0.15)
Random effects for super-regions	No
**Number of data points used to fit the model = 146**	

Traditional R^2 is not clearly defined for generalised linear models. The pseudo-R^2 for the model, which describes the proportion of variance explained by the model,[4] was 0.796.

Independent variable: mean FPG and diabetes prevalence (FPG ≥7.0 mmol/L)	
**Variables**	**Coefficients (95% CI)**
Intercept	0.055 (-0.13, 0.24)
Inverse mean FPG	-4.53 (-5.74, -3.31)
Probit-transformed prevalence (FPG ≥7.0 mmol/L)	0.70 (0.66, 0.75)
Mean age of age group	-0.0026 (-0.0058, 0.00070)
Male sex	-0.44 (-0.54, -0.34)
Natural logarithm of per-capita gross domestic product	0.034 (0.028, 0.040)
Study mid-year (per one more recent year since 1980)	-0.000093 (-0.00056, 0.00037)
Inverse mean FPG * mean age of age group	0.061 (0.038, 0.085)
Inverse mean FPG * male sex	3.73 (2.96, 4.49)
Probit-transformed prevalence (FPG ≥7.0 mmol/L) * mean age of age group	0.0029 (0.0020, 0.0039)
Probit-transformed prevalence (FPG ≥7.0 mmol/L) * male sex	0.19 (0.15, 0.22)
Random effects for super-regions	Yes
**Number of data points used to fit the model = 3,569**	

Traditional R^2 is not clearly defined for mixed-effect models. The pseudo-R^2 for the model, which describes the proportion of variance explained by both fixed and random factors,[4] was 0.804.

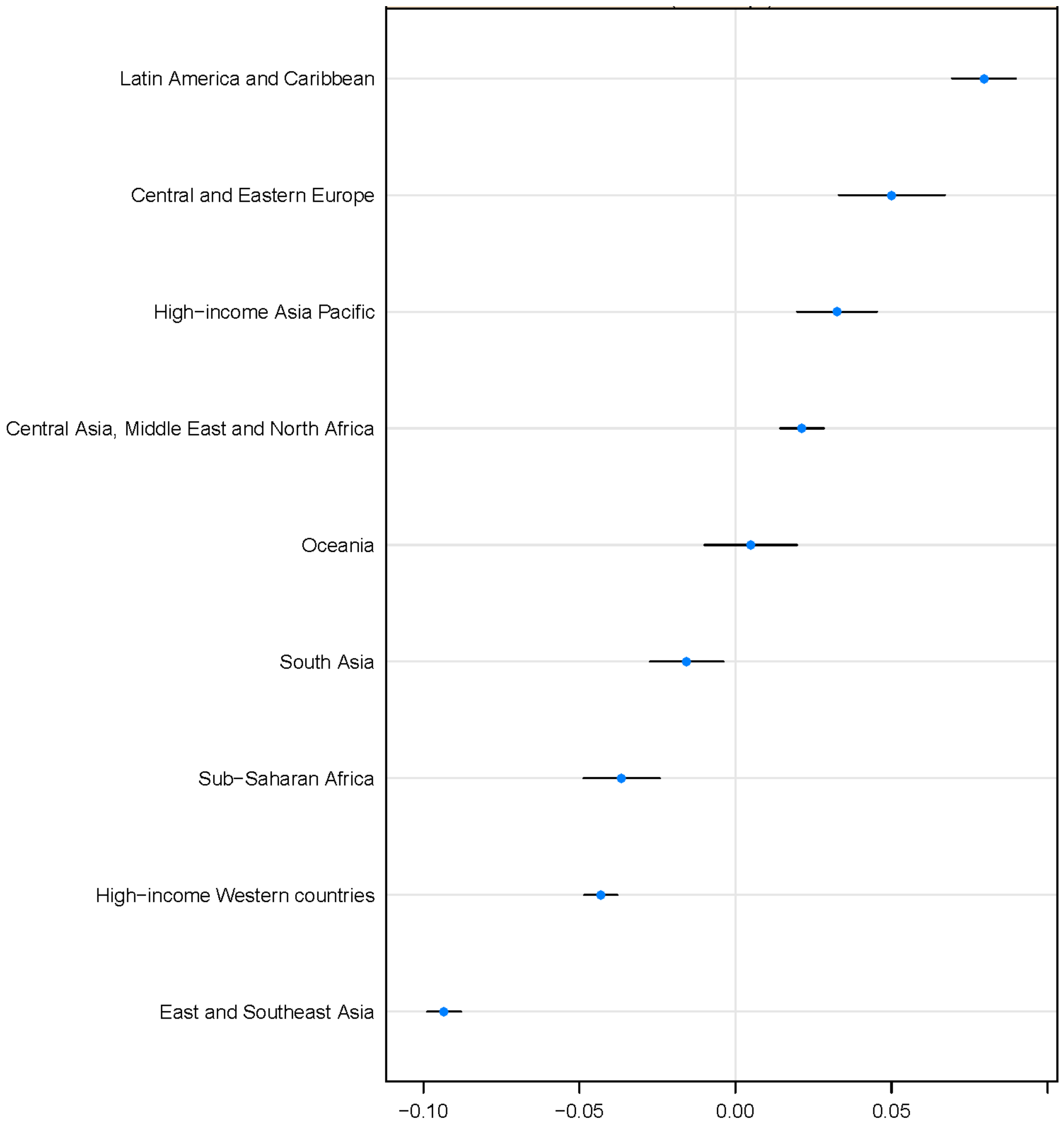
Latin America and Caribbean
Central and Eastern Europe
High−income Asia Pacific
Central Asia, Middle East and North Africa
Oceania
South Asia
Sub−Saharan Africa
High−income Western countries
East and Southeast Asia
−0.10
−0.05
0.00
0.05

Independent variable: mean HbA1c and diabetes prevalence (HbA1c ≥6.5% or history of diabetes diagnosis or use of diabetes medication)	
**Variables**	**Coefficients (95% CI)**
Intercept	1.11 (0.63, 1.59)
Inverse mean HbA1c	-5.46 (-7.96, -2.96)
Probit-transformed prevalence (HbA1c ≥6.5% or history of diabetes diagnosis or use of diabetes medication)	0.89 (0.82, 0.97)
Mean age of age group	-0.029 (-0.037, -0.022)
Male sex	0.029 (0.012, 0.045)
Natural logarithm of per-capita gross domestic product	-0.028 (-0.052, -0.0030)
Study mid-year (per one more recent year since 2000)	-0.0053 (-0.0075, -0.0032)
Inverse mean HbA1c * mean age of age group	0.18 (0.14, 0.23)
Probit-transformed prevalence (HbA1c ≥6.5% or history of diabetes diagnosis or use of diabetes medication) * mean age of age group	0.0013 (-0.0000042, 0.0026)
Random effects for super-regions	Yes
**Number of data points used to fit the model = 743**	

Traditional R^2 is not clearly defined for mixed-effect models. The pseudo-R^2 for the model, which describes the proportion of variance explained by both fixed and random factors,[4] was 0.780.

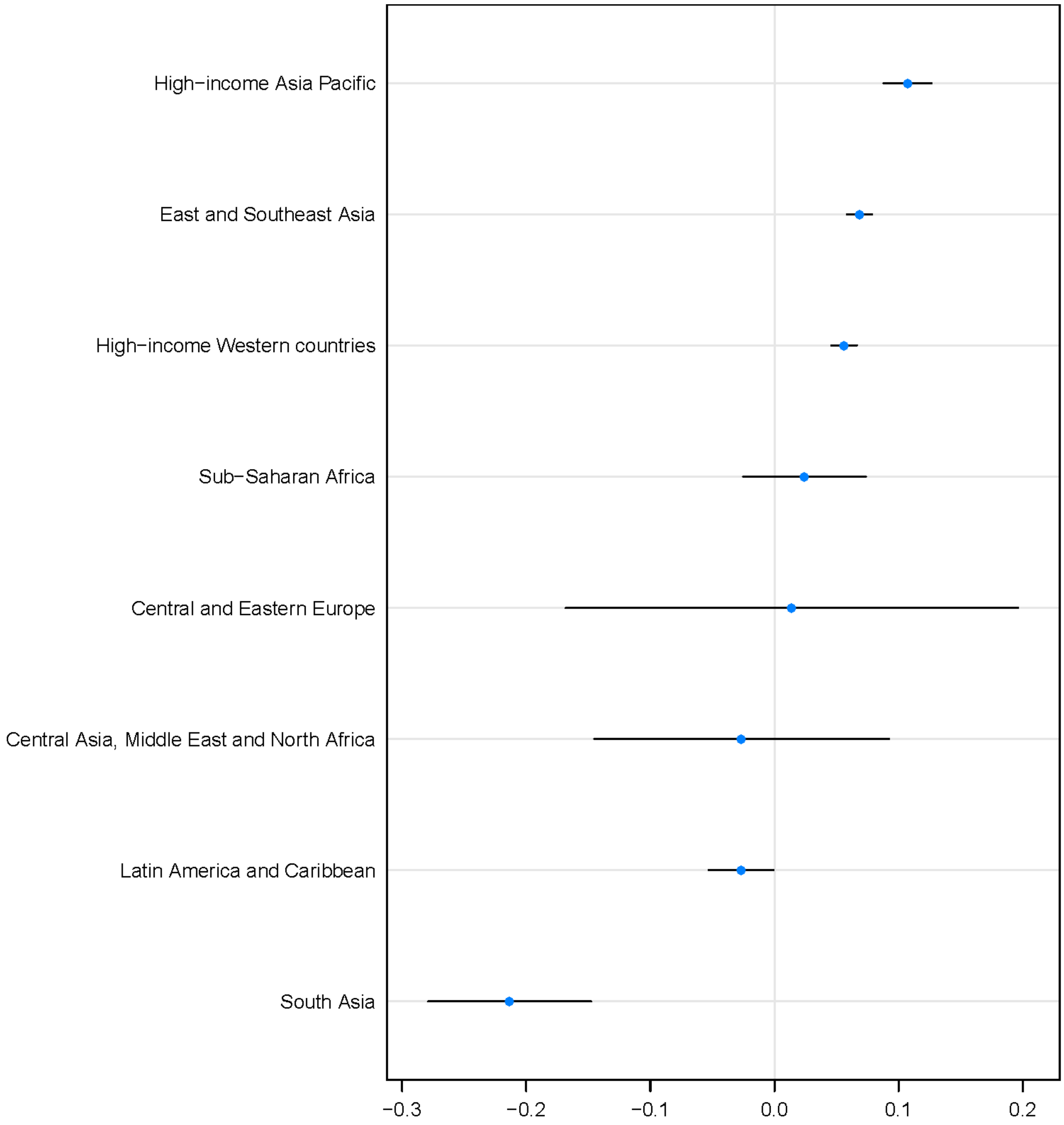
High−income Asia Pacific
East and Southeast Asia
High−income Western countries
Sub−Saharan Africa
Central and Eastern Europe
Central Asia, Middle East and North Africa
Latin America and Caribbean
South Asia
−0.3
−0.2
−0.1
0.0
0.1
0.2

Independent variable: diabetes prevalence (FPG ≥7.8 mmol/L or history of diabetes diagnosis or use of diabetes medication)	
**Variables**	**Coefficients (95% CI)**
Intercept	0.17 (0.097, 0.24)
Probit-transformed prevalence (FPG ≥7.8 mmol/L or history of diabetes diagnosis or use of diabetes medication)	0.96 (0.94, 0.96)
Mean age of age group	0.00031 (0.0000036, 0.00062)
Male sex	-0.022 (-0.041, -0.0028)
Natural logarithm of per-capita gross domestic product	-0.014 (-0.021, -0.0084)
Study mid-year (per one more recent year since 1980)	0.00099 (0.00053, 0.0014)
Probit-transformed prevalence (FPG ≥7.8 mmol/L or history of diabetes diagnosis or use of diabetes medication) * male sex	-0.023 (-0.037, -0.0084)
Random effects for super-regions	Yes
**Number of data points used to fit the model = 3,697**	

Traditional R^2 is not clearly defined for mixed-effect models. The pseudo-R^2 for the model, which describes the proportion of variance explained by both fixed and random factors,[4] was 0.842.

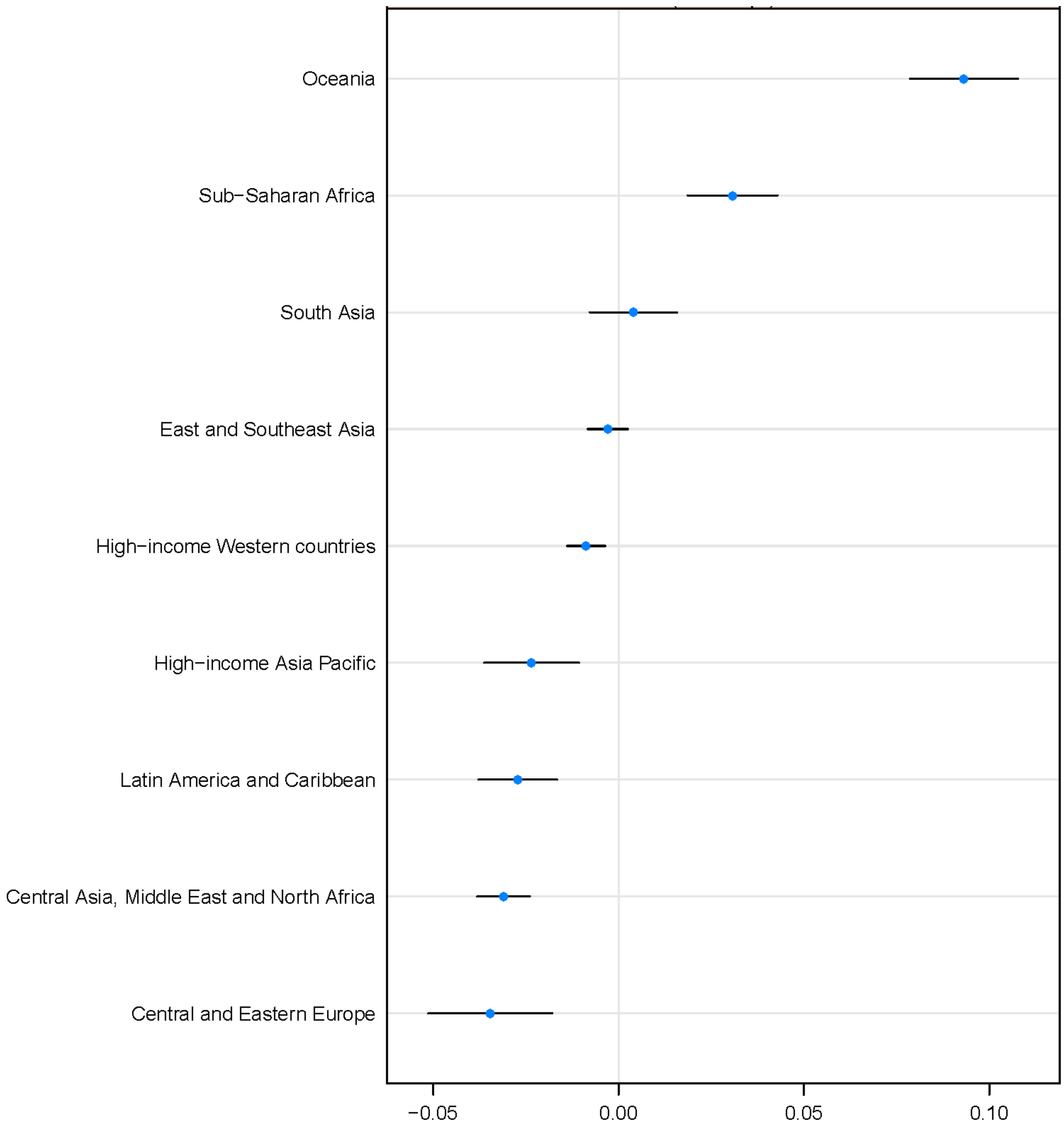
Oceania
Sub−Saharan Africa
South Asia
East and Southeast Asia
High−income Western countries
High−income Asia Pacific
Latin America and Caribbean
Central Asia, Middle East and North Africa
Central and Eastern Europe
−0.05
0.00
0.05
0.10

Independent variable: diabetes prevalence (FPG ≥7.0 mmol/L or HbA1c ≥6.5% or history of diabetes diagnosis or use of diabetes medication)	
**Variables**	**Coefficients (95% CI)**
Intercept	-0.61 (-0.79, -0.43)
Probit-transformed prevalence (FPG ≥7.0 mmol/L or HbA1c ≥6.5% or history of diabetes diagnosis or use of diabetes medication	0.94 (0.91, 0.98)
Mean age of age group	-0.000040 (-0.0010, 0.00095)
Male sex	0.025 (0.0062, 0.044)
Natural logarithm of per-capita gross domestic product	0.054 (0.038, 0.070)
Study mid-year (per one more recent year since 2000)	-0.0039 (-0.0062, -0.0016)
Random effects for super-regions	No[†]
**Number of data points used to fit the model = 497**	

[†] The model did not include regional random effects because if included, they were all estimated as zero. The coefficients of other variables were identical in models with and without regional random effects.
Traditional R^2 is not clearly defined for mixed-effect models. The pseudo-R^2 for the model, which describes the proportion of variance explained by both fixed and random factors,[4] was 0.779.

Appendix Table 5: Results of model validation.

Test 1, Men											
**Data**		**No. of held out observations**	**Percent covered**	**Error (percentage point) †**				**Absolute error (percentage point)**			
				**Median**	**Q1**	**Q3**	**(p*)**	**Median**	**Q1**	**Q3**	**(p*)**
All		1782	96	-0.16	-3.34	2.20	0.7443	2.77	0.98	6.09	0.7443
Super-region	Central and Eastern Europe	70	96	0.22	-2.14	1.67	0.6662	1.87	0.80	5.84	0.6662
	Central Asia, Middle East and North Africa	344	97	-0.46	-5.39	1.55	0.6949	3.07	0.91	8.35	0.6949
	East and Southeast Asia	195	96	-0.50	-2.55	1.46	0.8942	2.02	0.75	4.99	0.8942
	High-income Asia Pacific	80	90	1.67	0.27	4.01	0.0617	2.33	1.14	4.57	0.0617
	High-income Western countries	562	96	0.18	-2.74	2.36	0.8613	2.49	0.94	4.60	0.8613
	Latin America and Caribbean	215	95	-1.01	-5.90	1.65	0.2222	3.84	1.35	7.35	0.2222
	Oceania	92	95	1.31	-2.61	7.03	0.6369	5.42	2.11	12.38	0.6369
	South Asia	134	97	-1.60	-5.15	0.70	0.0261	3.34	1.27	7.33	0.0261
	Sub-Saharan Africa	90	93	0.05	-3.44	2.15	0.9532	3.02	0.81	7.62	0.9532
Urban or rural studies	Rural	229	95	-0.75	-3.15	1.59	0.5372	2.73	0.93	5.73	0.5372
	Urban	518	95	-0.30	-4.12	1.60	0.3636	2.83	1.03	6.55	0.3636
	Both rural and urban	1035	96	0.07	-3.07	2.51	0.6513	2.76	0.98	6.02	0.6513
Study representative-ness	Community	803	95	-0.49	-4.02	1.59	0.1359	2.82	0.99	6.14	0.1359
	Subnational	293	95	-0.42	-2.97	2.18	0.6060	2.57	0.96	5.17	0.6060
	National	686	97	0.30	-2.68	2.84	0.4356	2.79	0.99	6.44	0.4356
Age band	18-40	503	89	-0.34	-1.69	0.65	0.0029	1.06	0.49	2.22	0.0029
	40-60	585	99	-0.05	-3.35	2.54	0.4854	2.94	1.18	5.59	0.4854
	60 and above	694	98	0.25	-5.57	4.52	0.9571	4.99	2.43	8.83	0.9571
Years	1980-1989	111	100	-0.86	-3.30	1.99	0.2145	2.49	1.23	5.18	0.2145
	1990-1999	376	95	-0.41	-4.24	2.38	0.6899	3.21	1.00	6.70	0.6899
	2000-2009	948	96	-0.28	-3.39	1.95	0.9269	2.72	0.96	5.83	0.9269
	2010-2014	347	94	0.39	-2.25	2.88	0.6638	2.57	0.97	6.15	0.6638
Data density	Data poor	149	95	0.40	-3.05	3.98	0.7449	3.62	1.46	7.71	0.7449
	Average data density	301	98	-1.38	-7.28	1.33	0.0241	3.58	1.38	8.92	0.0241
	Data rich	1332	95	-0.03	-2.74	2.32	0.6173	2.55	0.92	5.47	0.6173

**Test 1, Women**											
**Data**		**No. of held out observations**	**Percent covered**	**Error (percentage point) †**				**Absolute error (percentage point)**			
				**Median**	**Q1**	**Q3**	**(p*)**	**Median**	**Q1**	**Q3**	**(p*)**
All		1705	95	0.13	-3.13	2.73	0.1932	2.89	1.10	6.71	0.1932
Super-region	Central and Eastern Europe	75	96	0.80	-1.48	2.68	0.4104	2.33	0.89	4.41	0.4104
	Central Asia, Middle East and North Africa	323	95	0.08	-4.71	2.33	0.7531	3.20	1.19	8.28	0.7531
	East and Southeast Asia	134	96	-3.35	-6.32	0.36	0.0088	4.42	1.78	7.16	0.0088
	High-income Asia Pacific	80	90	1.13	-0.13	4.15	0.2370	2.52	0.68	4.98	0.2370
	High-income Western countries	435	91	0.36	-1.48	2.52	0.1009	2.02	0.83	4.49	0.1009
	Latin America and Caribbean	239	95	0.93	-2.59	4.44	0.0305	3.61	1.41	7.52	0.0305
	Oceania	104	100	1.92	-3.80	8.66	0.3168	6.37	2.54	12.60	0.3168
	South Asia	176	98	-2.01	-6.27	0.05	0.0052	3.21	1.21	7.99	0.0052
	Sub-Saharan Africa	139	96	0.83	-1.45	4.05	0.1165	2.29	1.15	6.85	0.1165
Urban or rural studies	Rural	209	87	0.35	-1.63	2.60	0.1167	2.09	0.89	5.93	0.1167
	Urban	498	94	-0.08	-3.97	2.59	0.8312	3.12	1.33	7.15	0.8312
	Both rural and urban	998	96	0.12	-3.17	2.81	0.3980	2.98	1.03	6.72	0.3980
Study representative-ness	Community	712	94	-0.21	-3.67	2.63	0.6201	3.16	1.33	7.07	0.6201
	Subnational	256	96	0.32	-1.72	3.31	0.1460	2.50	0.89	6.78	0.1460
	National	737	95	0.19	-3.06	2.66	0.5283	2.79	1.00	6.35	0.5283
Age band	18-40	534	86	-0.05	-1.45	0.93	0.6207	1.15	0.58	2.25	0.6207
	40-60	572	99	0.29	-2.87	3.12	0.4178	2.99	1.46	6.11	0.4178
	60 and above	599	97	0.86	-6.35	5.91	0.1197	6.17	3.15	11.15	0.1197
Years	1980-1989	114	94	0.18	-3.66	3.23	0.9432	3.49	0.84	5.84	0.9432
	1990-1999	307	98	-0.15	-3.29	2.50	0.8056	2.72	1.22	7.15	0.8056
	2000-2009	928	94	0.14	-3.25	2.61	0.3633	2.90	1.08	6.42	0.3633
	2010-2014	356	94	0.28	-2.58	3.42	0.3142	2.93	1.13	7.18	0.3142
Data density	Data poor	163	95	0.74	-1.91	4.11	0.2261	3.17	1.15	8.01	0.2261
	Average data density	305	94	-0.52	-4.76	2.83	0.7318	3.78	1.91	8.27	0.7318
	Data rich	1237	95	0.12	-2.66	2.56	0.2171	2.61	0.98	6.17	0.2171

Test 2, Men											
**Data**		**No. of held out observations**	**Percent covered**	**Error (percentage point) †**				**Absolute error (percentage point)**			
				**Median**	**Q1**	**Q3**	**(p*)**	**Median**	**Q1**	**Q3**	**(p*)**
All		1878	95	-0.31	-3.87	2.00	0.6528	2.84	1.06	6.69	0.6528
Super-region	Central and Eastern Europe	74	97	0.47	-2.17	2.10	0.6330	2.17	0.94	5.48	0.6330
	Central Asia, Middle East and North Africa	330	96	0.06	-4.52	2.92	0.6406	3.49	1.52	8.92	0.6406
	East and Southeast Asia	250	97	-0.33	-2.09	2.47	0.7050	2.21	0.90	5.35	0.7050
	High-income Asia Pacific	190	97	-1.55	-7.04	0.65	0.0052	2.89	1.02	7.18	0.0052
	High-income Western countries	589	94	-0.46	-4.11	1.36	0.1138	2.41	0.94	5.24	0.1138
	Latin America and Caribbean	181	94	-0.25	-4.37	3.06	0.9880	3.57	1.55	6.91	0.9880
	Oceania	84	93	2.97	-1.59	13.98	0.0185	7.06	2.49	15.28	0.0185
	South Asia	109	97	-0.64	-5.90	2.42	0.5534	3.19	1.41	7.77	0.5534
	Sub-Saharan Africa	71	94	-0.01	-2.58	1.54	0.5930	1.91	0.84	6.94	0.5930
Urban or rural studies	Rural	267	91	0.00	-2.63	2.03	0.3715	2.42	0.90	5.72	0.3715
	Urban	458	94	0.23	-3.53	2.37	0.6495	2.84	1.09	6.50	0.6495
	Both rural and urban	1153	97	-0.52	-4.32	1.78	0.2153	2.97	1.10	6.92	0.2153
Study representative-ness	Community	775	94	-0.10	-3.72	2.23	0.9566	2.94	1.13	6.19	0.9566
	Subnational	302	95	-0.05	-2.60	2.07	0.3126	2.42	0.93	6.04	0.3126
	National	801	97	-0.51	-4.55	1.69	0.2372	2.92	1.06	7.08	0.2372
Age band	18-40	518	87	-0.21	-1.46	0.80	0.1579	1.10	0.50	2.07	0.1579
	40-60	616	100	0.15	-3.50	2.83	0.9262	3.12	1.37	5.77	0.9262
	60 and above	744	98	-1.26	-7.72	3.88	0.0199	5.57	2.40	9.93	0.0199
Years	1980-1989	91	95	0.39	-2.80	3.60	0.2115	3.12	1.39	7.91	0.2115
	1990-1999	233	96	-0.53	-3.21	1.66	0.6311	2.51	1.06	5.15	0.6311
	2000-2009	1177	96	-0.34	-4.18	1.96	0.4765	2.85	1.04	6.77	0.4765
	2010-2014	377	94	0.15	-3.78	2.04	0.7252	2.89	1.06	7.02	0.7252
Data density	Data poor	*Not applicable for source-based test*									
	Average data density	348	96	-0.59	-5.30	2.17	0.8209	3.64	1.38	9.00	0.8209
	Data rich	1530	95	-0.26	-3.61	1.96	0.4946	2.75	1.02	6.16	0.4946
Hold out pattern	Post-2000 data removed	680	96	-0.40	-4.70	2.01	0.1575	3.12	1.16	7.15	0.1575
	Random set of data removed	1198	95	-0.28	-3.41	1.97	0.6528	2.72	1.03	6.20	0.6528

**Test 2, Women**												
**Data**		**No. of held out observations**	**Percent covered**	**Error (percentage point) †**				**Absolute error (percentage point)**				
				**Median**	**Q1**	**Q3**	**(p*)**	**Median**	**Q1**	**Q3**	**(p*)**	
All		1739	94	-0.12	-3.05	1.97	0.9142	2.57	0.95	5.92	0.9142	
Super-region	Central and Eastern Europe	56	98	-0.42	-3.70	1.37	0.5431	2.00	0.85	4.34	0.5431	
	Central Asia, Middle East and North Africa	279	96	-0.03	-5.12	2.46	0.8740	3.15	1.23	8.65	0.8740	
	East and Southeast Asia	194	97	-0.48	-3.30	1.61	0.4554	2.71	0.86	4.42	0.4554	
	High-income Asia Pacific	225	86	0.51	-2.10	2.57	0.2911	2.31	0.93	4.48	0.2911	
	High-income Western countries	518	93	-0.67	-2.76	1.21	0.0510	1.73	0.82	4.17	0.0510	
	Latin America and Caribbean	199	94	-0.28	-5.15	3.27	0.6751	4.03	1.44	8.93	0.6751	
	Oceania	100	97	1.16	-4.19	5.07	0.7684	5.05	2.20	11.30	0.7684	
	South Asia	109	97	0.39	-1.42	2.37	0.3274	2.23	0.91	5.82	0.3274	
	Sub-Saharan Africa	59	97	0.29	-1.97	2.25	0.4257	2.16	1.09	6.65	0.4257	
Urban or rural studies	Rural	259	86	0.02	-2.83	2.72	0.6099	2.78	1.02	5.86	0.6099	
	Urban	467	93	0.05	-2.96	1.82	0.8078	2.27	0.91	5.52	0.8078	
	Both rural and urban	1013	96	-0.23	-3.25	2.04	0.6734	2.57	0.95	6.02	0.6734	
Study representative-ness	Community	708	91	-0.07	-3.51	1.85	0.7801	2.70	1.11	5.82	0.7801	
	Subnational	304	98	-0.19	-2.43	2.07	0.9138	2.25	0.89	5.65	0.9138	
	National	727	95	-0.06	-3.04	2.11	0.9740	2.55	0.94	6.07	0.9740	
Age band	18-40	514	84	-0.07	-1.35	0.76	0.4465	1.03	0.48	2.11	0.4465	
	40-60	579	98	-0.01	-2.92	2.29	0.7739	2.68	1.15	5.41	0.7739	
	60 and above	646	98	-0.42	-7.08	3.96	0.4847	5.16	2.34	9.78	0.4847	
Years	1980-1989	105	90	0.11	-2.21	1.89	0.9042	2.13	0.80	5.08	0.9042	
	1990-1999	329	93	-0.04	-3.22	2.22	0.5032	2.75	1.18	6.56	0.5032	
	2000-2009	939	95	-0.21	-3.31	1.85	0.8813	2.57	1.01	5.81	0.8813	
	2010-2014	366	94	-0.08	-2.68	1.92	0.5341	2.35	0.87	5.70	0.5341	
Data density	Data poor	*Not applicable for source-based test*										
	Average data density	326	94	-0.66	-4.20	1.83	0.6838	2.91	1.17	7.61	0.6838	
	Data rich	1413	94	-0.03	-2.92	1.98	0.9719	2.45	0.91	5.60	0.9719	
Hold out pattern	Post-2000 data removed	306	92	0.51	-2.15	2.71	0.2590	2.49	0.97	5.33	0.2590	
	Random set of data removed	1433	94	-0.25	-3.28	1.79	0.4610	2.59	0.94	6.14	0.4610	

† Estimated values minus held out values.

* p-values for model error comparisons were calculated using the non-parametric Wilcoxon signed-rank test for paired data. The p-values are calculated assuming independence of the held-out observations. They should therefore be interpreted as an approximation because there is some dependence among the held-out observations, within each of the five repetitions for example.

Appendix Table 6: Age-standardised diabetes prevalence by sex and country in people aged 18 years and older in 1980, 1990, 2000, 2010 and 2014. Numbers in brackets show 95% credible intervals.

Country (Men)	Year				
	1980	1990	2000	2010	2014
Afghanistan	4.5% (1.5-9.5)	6.2% (3.4-9.9)	8.1% (5.3-11.5)	10.5% (6.8-15.1)	11.6% (6.4-18.2)
Albania	4.2% (1.2-10.0)	5.0% (2.3-8.9)	6.0% (3.4-9.4)	7.2% (4.4-10.9)	7.7% (3.9-13.1)
Algeria	4.7% (1.7-10.0)	6.3% (3.8-9.6)	8.6% (6.0-11.8)	11.2% (7.8-15.5)	12.3% (7.4-18.8)
American Samoa	17.4% (9.1-27.4)	25.4% (18.0-33.3)	30.5% (22.5-38.9)	31.0% (21.9-41.1)	30.8% (19.1-43.7)
Andorra	6.4% (2.7-12.2)	7.2% (4.4-10.9)	7.7% (5.2-10.9)	8.1% (5.2-11.8)	8.1% (4.3-13.5)
Angola	2.6% (0.7-6.5)	3.9% (1.8-7.1)	5.3% (2.9-8.8)	7.6% (4.1-13.1)	8.5% (3.8-16.2)
Antigua and Barbuda	3.9% (1.3-9.0)	5.0% (2.6-8.5)	6.5% (3.8-10.2)	8.7% (4.7-14.2)	9.9% (4.4-17.9)
Argentina	7.4% (2.7-15.1)	8.0% (4.7-12.5)	8.7% (5.6-12.6)	9.4% (5.9-13.8)	9.9% (5.1-16.3)
Armenia	5.9% (2.0-12.5)	6.8% (3.6-11.4)	7.8% (4.5-12.0)	10.0% (6.3-14.8)	11.0% (5.9-17.6)
Australia	5.6% (2.5-10.3)	6.0% (3.8-8.8)	6.7% (4.7-9.1)	6.8% (4.6-9.2)	6.8% (3.8-10.5)
Austria	4.1% (1.6-8.5)	4.5% (2.7-7.0)	5.1% (3.4-7.3)	5.4% (3.4-7.9)	5.4% (2.8-9.1)
Azerbaijan	5.5% (1.8-11.7)	6.4% (3.4-10.8)	7.4% (4.2-11.5)	10.2% (6.3-15.1)	11.6% (6.2-18.6)
Bahamas	6.3% (2.3-12.9)	7.8% (4.6-12.5)	9.4% (6.2-13.8)	10.7% (6.5-16.4)	11.2% (5.5-18.7)
Bahrain	8.4% (3.4-16.5)	9.3% (5.9-13.4)	10.6% (7.6-14.2)	11.6% (8.0-16.0)	12.0% (7.0-18.5)
Bangladesh	3.8% (1.3-8.5)	5.5% (3.1-8.8)	7.5% (5.1-10.4)	9.5% (6.3-13.5)	10.3% (5.8-15.9)
Barbados	5.0% (1.7-11.2)	6.2% (3.4-10.2)	7.8% (4.9-11.7)	9.7% (5.7-15.1)	10.5% (5.1-17.9)
Belarus	4.7% (1.5-10.6)	5.6% (2.8-9.4)	6.4% (3.6-9.9)	7.5% (4.2-11.9)	8.0% (3.7-14.3)
Belgium	6.4% (2.6-12.6)	6.1% (3.6-9.2)	5.9% (3.9-8.2)	5.6% (3.7-8.1)	5.7% (3.0-9.2)
Belize	5.4% (1.9-11.4)	6.3% (3.5-10.1)	7.8% (5.0-11.5)	9.3% (5.5-14.0)	10.0% (4.8-17.1)
Benin	3.1% (0.9-7.6)	4.2% (2.0-7.4)	5.7% (3.4-8.7)	7.0% (4.3-10.4)	7.4% (3.7-12.4)
Bermuda	10.6% (3.7-21.3)	11.3% (6.1-18.5)	11.5% (7.1-17.4)	13.3% (8.0-20.4)	14.2% (7.1-23.9)
Bhutan	3.9% (1.3-8.7)	6.1% (3.4-9.7)	9.0% (6.1-12.4)	11.3% (7.5-15.5)	11.9% (6.7-17.9)
Bolivia	4.1% (1.2-10.2)	4.8% (2.2-8.4)	5.6% (3.0-8.5)	6.4% (3.4-10.2)	7.0% (2.9-12.4)
Bosnia and Herzegovina	4.2% (1.2-10.4)	5.0% (2.2-9.3)	6.7% (3.5-11.0)	7.7% (4.6-11.8)	8.0% (4.1-13.3)
Botswana	2.0% (0.5-5.3)	3.6% (1.7-6.4)	5.5% (3.2-8.5)	6.8% (4.1-10.5)	7.6% (4.0-12.6)
Brazil	4.8% (1.6-10.6)	5.8% (3.2-9.2)	6.6% (4.2-9.5)	7.5% (4.6-11.1)	7.8% (4.1-13.3)
Brunei Darussalam	6.3% (2.4-12.8)	7.3% (4.3-11.6)	8.5% (5.4-12.5)	9.2% (5.6-13.8)	9.2% (4.7-15.4)
Bulgaria	4.6% (1.7-9.9)	5.6% (3.2-8.9)	6.4% (4.1-9.4)	7.8% (4.8-11.7)	8.4% (4.3-14.3)
Burkina Faso	2.3% (0.6-6.3)	3.3% (1.5-6.2)	4.8% (2.7-7.8)	6.7% (3.9-10.4)	7.4% (3.6-12.5)
Burundi	1.0% (0.2-2.9)	1.6% (0.5-3.6)	2.5% (1.1-4.9)	3.7% (1.8-6.4)	4.2% (1.8-7.9)
Cabo Verde	3.1% (0.8-7.6)	4.5% (2.3-7.8)	6.7% (4.1-10.0)	8.7% (5.4-12.9)	9.1% (4.6-15.2)
Cambodia	2.7% (0.7-6.7)	3.7% (1.8-6.5)	5.0% (3.0-7.8)	6.7% (4.2-9.8)	7.4% (3.8-12.1)
Cameroon	2.9% (0.8-7.6)	3.9% (2.0-6.7)	5.0% (3.1-7.5)	6.2% (3.7-9.2)	6.5% (3.2-11.2)
Canada	5.0% (2.1-9.7)	5.5% (3.4-8.2)	6.1% (4.1-8.5)	6.2% (4.2-9.0)	6.2% (3.3-10.3)
Central African Republic	2.9% (0.8-7.1)	3.8% (1.7-7.1)	5.1% (2.6-8.8)	7.1% (3.7-12.5)	8.0% (3.5-15.4)
Chad	2.8% (0.7-7.0)	3.7% (1.7-6.8)	5.3% (2.9-8.5)	7.2% (4.1-11.3)	7.9% (3.9-13.8)
Chile	7.2% (2.7-14.6)	7.5% (4.4-11.6)	8.4% (5.7-11.7)	9.5% (6.2-13.6)	10.2% (5.5-16.2)

Country (Men)	Year				
	1980	1990	2000	2010	2014
China	3.5% (1.0-8.4)	4.4% (2.3-7.4)	6.2% (4.3-8.4)	8.8% (6.1-12.2)	9.9% (5.7-15.7)
China (Hong Kong SAR)	7.8% (2.8-16.7)	8.1% (4.4-13.2)	8.9% (5.3-13.6)	8.9% (4.5-14.9)	9.3% (3.7-17.7)
Colombia	4.4% (1.6-9.8)	5.4% (3.0-8.8)	6.7% (4.3-9.7)	7.8% (5.0-11.3)	8.3% (4.3-13.8)
Comoros	3.2% (0.9-7.6)	4.2% (2.0-7.5)	5.8% (3.3-9.3)	7.2% (4.4-10.8)	7.9% (4.1-12.9)
Congo	3.3% (0.9-7.9)	4.7% (2.0-8.8)	5.8% (2.9-10.2)	7.0% (3.6-12.8)	7.7% (3.4-15.5)
Cook Islands	11.0% (5.6-17.7)	17.9% (12.4-23.9)	23.8% (17.4-30.5)	27.4% (19.6-36.4)	28.3% (17.6-40.4)
Costa Rica	4.2% (1.5-9.7)	5.5% (3.1-8.9)	7.0% (4.7-10.0)	8.4% (5.4-12.0)	8.8% (4.6-14.7)
Cote d'Ivoire	3.3% (0.9-8.4)	4.3% (2.1-7.7)	5.6% (3.3-8.6)	6.8% (4.0-10.5)	7.3% (3.6-12.6)
Croatia	4.3% (1.5-9.6)	5.3% (2.9-8.7)	6.1% (3.8-9.0)	7.6% (4.6-11.3)	8.4% (4.2-14.2)
Cuba	5.0% (1.8-10.8)	6.2% (3.6-10.0)	6.7% (4.2-10.2)	6.9% (4.0-11.0)	7.3% (3.5-12.7)
Cyprus	3.4% (1.2-7.5)	5.0% (2.9-8.0)	6.9% (4.6-9.9)	8.0% (5.1-11.8)	8.3% (4.3-13.7)
Czech Republic	6.2% (2.6-12.1)	6.7% (3.9-10.2)	7.1% (4.6-10.2)	8.1% (4.9-11.9)	8.4% (4.3-14.3)
Denmark	4.9% (1.9-9.8)	5.2% (3.2-8.1)	5.6% (3.6-8.1)	5.4% (3.3-7.9)	5.4% (2.7-8.9)
Djibouti	5.7% (2.0-11.9)	6.9% (3.6-11.4)	8.1% (4.7-12.9)	8.6% (4.8-13.8)	8.6% (4.0-15.2)
Dominica	4.0% (1.3-8.9)	5.4% (2.9-8.7)	6.6% (4.1-10.1)	7.9% (4.6-12.4)	8.5% (4.1-14.9)
Dominican Republic	3.5% (1.1-8.0)	4.7% (2.6-7.8)	6.7% (4.2-9.9)	8.2% (4.9-12.9)	8.6% (4.1-15.1)
DR Congo	2.3% (0.6-5.8)	3.2% (1.4-6.1)	4.2% (2.1-7.3)	5.5% (2.7-10.2)	6.2% (2.6-12.6)
Ecuador	3.7% (1.1-9.3)	4.6% (2.1-8.0)	5.7% (3.2-8.7)	6.8% (3.6-10.6)	7.5% (3.1-13.2)
Egypt	6.5% (2.4-13.3)	8.4% (5.1-12.5)	10.8% (7.5-14.7)	14.2% (10.0-19.1)	16.0% (10.0-23.6)
El Salvador	4.9% (1.6-11.1)	6.2% (3.3-10.2)	7.9% (5.1-11.5)	8.9% (5.7-13.1)	9.3% (4.8-15.6)
Equatorial Guinea	2.6% (0.7-6.2)	3.5% (1.6-6.7)	5.5% (3.0-9.3)	8.5% (4.6-14.5)	9.5% (4.4-17.7)
Eritrea	2.1% (0.6-5.3)	3.0% (1.3-5.6)	4.3% (2.4-7.1)	5.4% (3.1-8.6)	6.0% (2.9-10.5)
Estonia	5.9% (2.0-13.1)	6.2% (3.2-10.4)	6.5% (3.8-9.9)	7.4% (4.1-11.5)	7.7% (3.5-13.8)
Ethiopia	2.2% (0.5-5.7)	3.0% (1.3-5.8)	4.2% (2.3-7.0)	5.3% (2.9-8.6)	5.8% (2.7-10.4)
Fiji	8.3% (3.5-16.0)	9.0% (5.1-14.3)	11.0% (6.9-17.0)	14.5% (8.7-21.7)	15.9% (8.4-25.8)
Finland	5.7% (2.5-11.0)	6.4% (4.1-9.3)	6.7% (4.8-8.8)	6.5% (4.4-9.0)	6.4% (3.6-10.3)
France	5.2% (2.1-10.2)	5.8% (3.5-8.9)	6.6% (4.5-9.3)	7.3% (4.7-10.6)	7.5% (4.1-12.2)
French Polynesia	10.2% (4.6-18.1)	15.0% (9.6-21.5)	19.1% (13.3-25.7)	21.6% (14.7-29.6)	22.3% (13.2-33.3)
Gabon	3.6% (1.1-8.5)	5.6% (2.6-10.2)	7.7% (4.0-13.1)	9.4% (4.8-16.5)	10.0% (4.4-19.2)
Gambia	3.3% (0.9-8.2)	4.3% (2.1-7.5)	6.1% (3.6-9.3)	8.4% (5.1-12.8)	9.4% (4.7-16.1)
Georgia	5.5% (1.8-12.1)	6.7% (3.5-11.3)	7.9% (4.6-12.2)	11.4% (7.1-16.7)	13.3% (7.3-20.8)
Germany	5.0% (2.1-10.0)	5.3% (3.2-8.2)	5.7% (3.9-7.8)	5.9% (4.0-8.3)	6.0% (3.3-9.8)
Ghana	3.0% (0.9-7.6)	3.9% (1.9-7.0)	5.2% (2.9-8.2)	6.1% (3.5-9.6)	6.4% (3.1-11.0)
Greece	4.9% (1.8-9.8)	5.7% (3.4-8.7)	6.4% (4.2-9.3)	7.0% (4.4-10.6)	7.2% (3.7-12.1)
Greenland	4.7% (1.8-9.7)	5.2% (3.1-8.0)	5.5% (3.7-7.7)	5.5% (3.6-7.9)	5.6% (2.9-9.2)
Grenada	3.4% (1.0-7.9)	4.4% (2.3-7.5)	6.1% (3.7-9.2)	8.0% (4.5-12.7)	8.8% (4.0-15.6)
Guatemala	4.4% (1.4-10.3)	5.4% (2.7-9.4)	6.9% (4.1-10.4)	8.4% (5.0-12.8)	8.9% (4.3-15.3)
Guinea	2.4% (0.6-6.2)	3.3% (1.5-6.1)	4.6% (2.7-7.3)	6.1% (3.6-9.4)	6.8% (3.3-11.6)
Guinea Bissau	2.6% (0.7-6.6)	3.6% (1.7-6.5)	5.1% (3.0-8.0)	6.8% (4.1-10.5)	7.4% (3.7-12.6)
Guyana	3.6% (1.1-8.3)	4.6% (2.3-7.9)	6.2% (3.6-9.8)	8.1% (4.5-13.2)	9.1% (4.1-16.3)
Haiti	2.8% (0.8-7.2)	4.0% (1.8-7.2)	5.7% (3.2-9.3)	7.8% (4.4-12.5)	8.6% (4.0-15.5)
Honduras	3.7% (1.1-8.8)	4.8% (2.4-8.3)	6.1% (3.8-9.1)	7.8% (4.8-11.7)	8.5% (4.2-14.4)

Country (Men)	Year				
	1980	1990	2000	2010	2014
Hungary	5.5% (2.1-11.6)	6.5% (3.8-10.1)	7.2% (4.7-10.3)	8.4% (5.2-12.3)	8.9% (4.6-14.6)
Iceland	7.4% (3.0-14.5)	8.4% (4.7-13.2)	8.0% (5.1-11.6)	7.7% (4.7-11.4)	7.6% (3.8-12.8)
India	3.7% (1.3-7.8)	5.4% (3.2-8.4)	7.3% (5.2-9.9)	8.7% (6.0-12.2)	9.1% (5.2-14.2)
Indonesia	3.2% (1.0-7.3)	4.3% (2.3-7.2)	5.8% (3.7-8.5)	7.0% (4.5-10.1)	7.4% (3.8-12.1)
Iran	5.0% (1.9-10.6)	5.8% (3.6-8.5)	7.4% (5.5-9.4)	10.2% (7.5-13.4)	11.4% (7.2-17.2)
Iraq	7.5% (3.2-14.6)	9.4% (6.0-13.4)	11.5% (8.2-15.4)	15.2% (10.7-20.7)	17.2% (10.7-25.3)
Ireland	4.3% (1.6-8.7)	5.2% (2.9-8.2)	6.3% (4.0-9.2)	7.1% (4.6-10.2)	7.3% (4.0-11.6)
Israel	5.5% (2.2-10.7)	6.3% (3.9-9.5)	6.8% (4.5-9.9)	6.9% (4.3-10.5)	7.1% (3.6-12.2)
Italy	4.5% (1.9-9.1)	5.2% (3.3-7.6)	6.0% (4.3-8.0)	6.7% (4.6-9.5)	7.1% (3.9-11.2)
Jamaica	3.9% (1.3-8.6)	4.7% (2.7-7.7)	6.4% (4.2-9.5)	8.3% (5.0-12.9)	9.3% (4.5-16.0)
Japan	5.7% (2.7-9.9)	6.4% (4.5-8.6)	7.3% (5.7-9.3)	8.2% (6.0-10.8)	8.4% (5.2-12.4)
Jordan	7.0% (2.8-13.8)	9.4% (6.0-13.5)	12.0% (8.8-15.9)	15.1% (11.0-20.1)	16.5% (10.5-24.0)
Kazakhstan	6.1% (2.1-12.8)	7.5% (3.9-12.2)	8.9% (5.3-13.6)	11.3% (7.0-16.5)	12.4% (6.6-19.8)
Kenya	2.1% (0.6-5.2)	3.0% (1.4-5.4)	4.1% (2.4-6.6)	5.3% (3.1-8.2)	5.8% (2.9-10.0)
Kiribati	6.8% (2.9-12.7)	11.9% (7.4-17.3)	17.3% (12.2-23.1)	21.0% (14.3-29.2)	22.0% (12.7-33.6)
Kuwait	11.7% (4.9-21.1)	13.3% (8.3-19.3)	15.3% (11.2-20.3)	18.4% (13.4-24.4)	19.7% (12.8-28.1)
Kyrgyzstan	4.4% (1.4-9.7)	5.4% (2.7-9.2)	6.7% (3.8-10.5)	8.8% (5.3-13.2)	9.9% (5.3-16.1)
Lao PDR	3.1% (0.8-7.4)	3.9% (1.9-7.0)	5.4% (3.2-8.4)	7.1% (4.5-10.4)	7.7% (4.1-12.4)
Latvia	6.1% (2.0-13.4)	6.5% (3.3-11.0)	6.6% (3.8-10.2)	7.3% (4.1-11.3)	7.8% (3.6-13.7)
Lebanon	7.1% (2.9-13.9)	8.0% (4.9-11.9)	9.0% (5.7-13.1)	12.5% (8.3-17.5)	14.5% (8.7-21.8)
Lesotho	2.3% (0.6-5.7)	3.1% (1.3-5.8)	4.7% (2.4-7.6)	6.5% (3.7-10.3)	7.3% (3.5-12.8)
Liberia	4.0% (1.2-9.2)	5.6% (3.0-9.4)	5.7% (3.5-8.5)	7.2% (4.6-10.6)	7.8% (4.1-12.9)
Libya	7.5% (3.0-14.8)	9.0% (5.8-13.1)	10.7% (7.9-14.1)	14.0% (10.3-18.7)	15.2% (9.5-22.5)
Lithuania	6.5% (2.2-14.6)	7.0% (3.6-11.8)	7.0% (4.1-10.7)	8.1% (4.7-12.5)	8.9% (4.3-15.7)
Luxembourg	5.9% (2.3-12.2)	6.4% (3.5-10.5)	6.8% (4.2-10.2)	6.8% (4.3-9.9)	7.0% (3.6-11.5)
Macedonia (TFYR)	4.7% (1.6-10.3)	5.6% (3.0-9.3)	6.4% (3.9-9.6)	7.1% (4.3-10.8)	7.6% (3.8-12.9)
Madagascar	2.4% (0.7-6.0)	3.5% (1.7-6.3)	4.7% (2.7-7.4)	5.8% (3.5-9.0)	6.3% (3.1-10.8)
Malawi	2.3% (0.6-5.8)	3.2% (1.4-6.0)	4.6% (2.6-7.4)	5.9% (3.5-9.1)	6.6% (3.3-11.4)
Malaysia	5.0% (1.8-10.3)	6.5% (3.9-10.0)	8.8% (6.1-12.2)	10.8% (7.3-15.2)	11.4% (6.5-17.7)
Maldives	3.6% (1.1-8.2)	5.4% (2.8-9.1)	7.8% (4.3-12.5)	10.5% (5.8-16.6)	11.1% (5.3-18.9)
Mali	3.0% (0.8-7.5)	4.0% (1.9-7.4)	5.4% (3.0-8.7)	7.5% (4.3-11.9)	8.4% (4.0-14.5)
Malta	7.1% (3.1-13.1)	7.6% (4.8-11.2)	8.3% (5.7-11.7)	8.9% (5.7-13.2)	9.0% (4.7-15.1)
Marshall Islands	9.2% (4.2-16.0)	13.7% (9.1-19.0)	17.7% (12.8-23.6)	20.0% (13.5-27.9)	20.8% (11.8-31.6)
Mauritania	3.3% (0.9-8.0)	4.9% (2.4-8.4)	6.5% (3.8-10.0)	8.0% (4.7-12.4)	8.5% (4.1-14.6)
Mauritius	7.1% (3.0-13.1)	9.0% (5.8-13.2)	11.1% (7.6-15.2)	12.3% (8.0-17.6)	12.9% (7.2-20.1)
Mexico	6.5% (2.5-13.2)	7.7% (4.6-11.9)	9.3% (6.2-13.1)	10.4% (6.9-14.8)	10.9% (5.8-17.7)
Micronesia (Federated States of)	6.4% (2.5-12.3)	9.9% (6.1-14.8)	14.2% (9.9-19.2)	18.6% (12.9-25.6)	20.5% (12.2-30.7)
Moldova	3.8% (1.1-9.2)	4.5% (2.0-8.0)	5.1% (2.7-8.3)	6.9% (3.7-11.0)	7.8% (3.6-13.3)
Mongolia	5.4% (1.7-11.4)	6.8% (3.6-11.4)	8.2% (4.8-12.7)	10.8% (6.8-15.8)	12.2% (6.8-19.2)
Montenegro	3.6% (1.0-8.9)	4.9% (2.5-8.5)	6.1% (3.8-9.2)	7.2% (4.4-10.9)	7.6% (3.7-12.9)
Morocco	4.9% (1.7-10.3)	6.5% (3.8-9.9)	9.0% (6.2-12.4)	12.5% (8.5-17.2)	14.0% (8.4-21.5)
Mozambique	2.3% (0.6-5.7)	3.3% (1.6-6.0)	4.9% (2.7-7.7)	6.1% (3.6-9.4)	6.6% (3.3-11.4)

Country (Men)	Year				
	1980	1990	2000	2010	2014
Myanmar	3.1% (0.9-7.1)	3.7% (1.9-6.3)	4.8% (2.9-7.3)	6.3% (4.0-9.2)	6.9% (3.7-11.1)
Namibia	2.6% (0.7-6.1)	3.4% (1.6-5.9)	4.9% (2.8-7.6)	6.7% (3.9-10.3)	7.3% (3.5-12.6)
Nauru	24.7% (13.7-37.0)	29.8% (21.2-39.3)	31.3% (23.0-40.3)	30.2% (20.8-40.8)	30.1% (17.9-43.9)
Nepal	4.0% (1.3-8.9)	5.7% (3.1-9.3)	8.3% (5.5-11.5)	10.8% (7.4-14.8)	11.7% (6.8-17.6)
Netherlands	3.9% (1.5-8.1)	4.3% (2.6-6.5)	5.0% (3.4-6.8)	5.2% (3.5-7.3)	5.2% (2.8-8.4)
New Zealand	6.1% (2.6-11.5)	6.6% (4.2-9.7)	7.3% (5.1-10.1)	7.7% (5.2-10.9)	7.9% (4.3-12.7)
Nicaragua	5.1% (1.7-11.1)	6.0% (3.2-9.8)	7.3% (4.6-10.8)	8.7% (5.3-13.1)	9.2% (4.6-15.7)
Niger	2.5% (0.6-6.4)	3.2% (1.3-6.0)	4.5% (2.4-7.5)	5.5% (3.1-9.0)	5.9% (2.7-10.9)
Nigeria	2.6% (0.7-6.6)	3.6% (1.7-6.4)	4.6% (2.8-7.0)	5.8% (3.6-8.7)	6.3% (3.2-10.6)
Niue	8.1% (3.5-14.9)	14.1% (9.2-20.2)	19.9% (14.5-26.4)	25.0% (17.8-33.4)	26.8% (16.7-38.3)
North Korea	5.0% (1.8-10.6)	5.1% (2.8-8.2)	5.1% (2.8-7.9)	5.4% (2.7-9.2)	5.8% (2.3-11.1)
Norway	4.6% (1.8-9.6)	5.3% (3.1-8.1)	6.0% (4.0-8.5)	6.2% (4.1-8.9)	6.2% (3.3-10.1)
Occupied Palestinian Territory	6.6% (2.6-12.9)	8.0% (5.1-11.4)	10.6% (7.8-14.0)	14.7% (10.6-19.6)	16.5% (10.3-24.3)
Oman	4.8% (1.7-9.9)	7.5% (4.8-10.8)	10.2% (7.6-13.6)	13.1% (9.4-17.6)	14.3% (8.6-21.7)
Pakistan	4.9% (1.8-10.1)	6.7% (4.1-10.1)	9.1% (6.3-12.2)	11.6% (7.6-16.1)	12.6% (7.0-19.5)
Palau	12.1% (6.1-19.9)	17.4% (12.0-23.3)	21.6% (15.7-28.0)	24.2% (16.9-32.7)	24.8% (15.0-36.1)
Panama	4.5% (1.5-9.7)	5.5% (3.0-8.7)	6.9% (4.5-9.9)	8.2% (5.2-11.9)	8.9% (4.6-14.7)
Papua New Guinea	5.9% (2.0-12.9)	7.9% (4.4-12.9)	10.7% (6.8-15.5)	13.8% (7.9-21.2)	15.4% (7.4-25.9)
Paraguay	3.4% (1.0-8.1)	4.4% (2.2-7.6)	6.1% (3.6-9.1)	7.4% (4.2-11.3)	8.0% (3.8-14.0)
Peru	4.9% (1.5-11.5)	5.7% (2.8-9.5)	6.2% (3.6-9.2)	6.8% (3.8-10.3)	7.2% (3.2-12.6)
Philippines	3.9% (1.4-8.6)	4.8% (2.8-7.7)	5.7% (3.9-8.2)	6.7% (4.4-9.4)	7.1% (3.9-11.2)
Poland	5.2% (2.0-10.8)	5.9% (3.4-9.1)	6.5% (4.3-9.2)	7.7% (5.1-11.1)	8.4% (4.5-13.5)
Portugal	3.8% (1.2-8.4)	5.0% (2.7-8.3)	6.5% (4.1-9.5)	7.9% (4.9-11.8)	8.4% (4.3-14.0)
Puerto Rico	6.8% (2.6-13.7)	9.9% (5.9-15.4)	11.6% (7.7-16.8)	12.6% (7.8-19.1)	13.0% (6.6-21.6)
Qatar	10.8% (4.5-20.5)	11.7% (7.6-16.7)	13.7% (9.9-18.1)	17.3% (12.6-22.9)	18.9% (12.0-27.0)
Romania	4.0% (1.3-8.9)	4.6% (2.5-7.4)	5.4% (3.3-7.9)	6.6% (3.9-10.0)	7.3% (3.6-12.3)
Russian Federation	5.6% (1.9-12.4)	6.0% (3.0-9.9)	6.2% (3.4-9.7)	7.0% (3.9-11.1)	7.4% (3.4-13.1)
Rwanda	1.1% (0.2-3.1)	1.6% (0.5-3.6)	2.7% (1.2-5.0)	3.8% (1.9-6.6)	4.3% (1.8-8.0)
Saint Kitts and Nevis	5.4% (1.8-11.6)	6.7% (3.6-10.9)	8.5% (5.2-12.8)	11.4% (6.6-17.8)	12.7% (6.0-21.6)
Saint Lucia	3.4% (1.0-8.0)	4.6% (2.4-7.9)	7.2% (4.2-10.9)	11.3% (6.0-18.4)	13.7% (6.1-24.1)
Saint Vincent and the Grenadines	4.1% (1.3-9.3)	5.4% (2.9-8.9)	7.3% (4.6-10.9)	8.8% (5.3-13.6)	9.3% (4.5-16.3)
Samoa	6.1% (2.3-12.4)	10.0% (6.0-14.8)	15.5% (10.7-20.7)	20.6% (13.7-28.5)	22.7% (13.1-33.9)
Sao Tome and Principe	4.3% (1.3-10.1)	5.7% (2.8-10.0)	7.3% (4.2-11.2)	8.0% (4.7-12.3)	8.3% (4.1-14.2)
Saudi Arabia	7.7% (3.1-14.7)	10.3% (6.8-14.7)	13.1% (9.8-17.1)	16.1% (12.0-21.3)	17.6% (11.5-25.4)
Senegal	3.6% (1.0-8.4)	4.6% (2.3-8.2)	5.8% (3.4-9.2)	7.1% (4.2-10.9)	7.5% (3.7-12.9)
Serbia	4.0% (1.3-9.3)	5.1% (2.7-8.5)	6.0% (3.7-9.0)	6.9% (4.2-10.4)	7.3% (3.5-12.5)
Seychelles	5.0% (1.7-10.7)	6.3% (3.5-10.1)	8.0% (4.9-12.1)	9.6% (6.0-14.5)	10.3% (5.5-16.7)
Sierra Leone	3.2% (0.9-7.8)	4.1% (2.0-7.3)	5.2% (3.1-8.2)	6.5% (3.9-9.9)	7.1% (3.5-12.1)
Singapore	7.8% (3.6-14.0)	9.0% (5.9-12.9)	9.5% (6.9-12.9)	9.2% (6.5-12.5)	9.4% (5.5-14.1)
Slovakia	4.8% (1.7-10.3)	5.7% (3.3-9.0)	6.3% (4.2-9.0)	7.5% (4.7-10.9)	8.2% (4.3-13.4)
Slovenia	4.2% (1.5-9.1)	5.1% (2.8-8.3)	6.0% (3.7-8.7)	6.8% (4.0-10.3)	7.3% (3.6-12.6)
Solomon Islands	5.5% (1.7-12.5)	7.4% (4.0-12.4)	9.8% (6.0-14.5)	11.7% (6.7-18.1)	12.6% (6.0-21.6)

Country (Men)	Year				
	1980	1990	2000	2010	2014
Somalia	3.0% (0.9-7.2)	4.1% (2.0-7.4)	5.6% (3.1-9.1)	6.9% (3.9-11.0)	7.4% (3.6-12.8)
South Africa	4.8% (1.6-10.2)	5.6% (3.0-9.0)	7.2% (4.4-10.7)	9.0% (5.5-13.6)	9.7% (4.9-16.3)
South Korea	4.0% (1.5-8.1)	6.6% (3.9-10.0)	8.2% (5.4-11.6)	8.6% (5.9-11.9)	9.3% (5.5-14.3)
Spain	5.4% (2.1-10.5)	6.5% (4.0-9.7)	7.9% (5.7-10.7)	8.5% (5.8-12.1)	8.5% (4.6-13.8)
Sri Lanka	2.9% (0.9-6.8)	3.5% (1.8-6.2)	4.8% (2.9-7.1)	6.3% (3.9-9.5)	7.0% (3.6-12.0)
Sudan	3.1% (0.9-7.3)	4.5% (2.2-7.8)	6.5% (3.9-10.1)	7.9% (4.8-11.8)	8.3% (4.3-13.7)
Suriname	6.1% (2.2-13.2)	7.5% (4.2-12.1)	8.5% (5.6-12.4)	10.2% (6.3-15.2)	10.9% (5.5-18.3)
Swaziland	2.7% (0.7-6.5)	3.9% (1.9-6.9)	5.4% (3.1-8.4)	7.1% (4.2-10.8)	7.9% (4.0-13.4)
Sweden	5.1% (2.1-10.1)	5.3% (3.2-7.9)	5.7% (4.1-7.7)	5.9% (3.9-8.2)	5.8% (3.2-9.3)
Switzerland	3.4% (1.3-7.3)	4.2% (2.4-6.6)	4.7% (3.1-6.8)	5.1% (3.3-7.4)	5.3% (2.8-8.9)
Syrian Arab Republic	5.6% (2.1-11.3)	7.1% (4.3-10.7)	9.8% (7.0-13.2)	12.7% (9.1-17.1)	14.0% (8.5-21.0)
Taiwan	4.8% (1.7-10.3)	6.0% (3.7-9.1)	6.6% (4.4-9.3)	7.5% (4.4-11.3)	8.0% (3.8-13.9)
Tajikistan	4.4% (1.4-9.8)	5.1% (2.5-8.9)	6.1% (3.4-9.8)	8.8% (5.1-13.7)	10.3% (5.1-17.4)
Tanzania	2.3% (0.6-5.7)	3.3% (1.6-5.8)	4.6% (2.7-7.2)	5.6% (3.4-8.4)	6.0% (3.1-9.9)
Thailand	3.5% (1.2-7.8)	4.6% (2.6-7.4)	6.1% (4.2-8.5)	7.7% (5.1-10.8)	8.3% (4.5-13.3)
Timor-Leste	2.4% (0.7-5.8)	3.5% (1.6-6.2)	4.6% (2.6-7.3)	5.2% (3.0-8.0)	5.4% (2.6-9.1)
Togo	3.1% (0.9-7.8)	4.0% (1.9-7.2)	5.4% (3.2-8.3)	6.8% (4.2-10.1)	7.3% (3.8-12.3)
Tokelau	6.9% (2.2-14.6)	12.0% (6.5-19.1)	18.0% (11.9-25.2)	23.9% (15.7-33.4)	26.2% (15.0-39.2)
Tonga	7.0% (2.7-13.8)	11.3% (6.8-16.8)	16.1% (11.2-21.7)	20.3% (13.7-27.7)	21.9% (12.9-32.6)
Trinidad and Tobago	3.2% (0.8-8.5)	4.1% (1.8-7.7)	6.0% (3.3-9.7)	9.0% (4.6-15.1)	10.4% (4.5-19.1)
Tunisia	4.8% (1.8-9.6)	6.2% (3.8-9.1)	8.3% (6.0-11.1)	11.0% (7.9-14.9)	12.1% (7.4-18.3)
Turkey	4.5% (1.5-9.5)	6.3% (3.9-9.1)	8.5% (6.2-11.0)	11.8% (8.9-15.1)	13.0% (8.7-18.5)
Turkmenistan	5.1% (1.7-11.1)	6.0% (3.1-10.0)	7.6% (4.5-11.5)	10.6% (6.7-15.4)	12.4% (6.9-19.6)
Tuvalu	7.6% (3.1-14.3)	12.9% (8.5-18.2)	18.4% (13.3-23.9)	22.0% (15.6-29.8)	23.2% (14.3-34.4)
Uganda	1.4% (0.3-3.9)	1.9% (0.6-3.9)	2.7% (1.2-5.0)	3.7% (1.9-6.4)	4.4% (2.1-7.8)
Ukraine	4.9% (1.5-11.1)	5.5% (2.7-9.3)	6.0% (3.3-9.5)	7.0% (3.8-11.2)	7.4% (3.3-13.3)
United Arab Emirates	9.6% (3.8-18.0)	9.6% (5.8-14.4)	11.8% (8.6-16.0)	14.7% (10.7-19.6)	15.0% (9.2-22.5)
United Kingdom	4.8% (2.0-9.0)	5.3% (3.4-7.7)	5.9% (4.2-7.9)	6.5% (4.8-8.4)	6.6% (4.1-9.7)
United States of America	4.7% (2.2-8.5)	5.7% (3.8-8.0)	7.0% (5.2-9.3)	7.9% (5.7-10.9)	8.2% (4.9-12.7)
Uruguay	7.5% (2.8-15.0)	7.7% (4.4-11.9)	8.3% (5.4-11.8)	8.8% (5.4-12.9)	9.1% (4.8-14.9)
Uzbekistan	4.5% (1.4-10.3)	5.4% (2.6-9.2)	6.6% (3.8-10.3)	9.1% (5.4-13.5)	10.5% (5.6-16.9)
Vanuatu	6.9% (2.5-14.9)	8.4% (4.7-13.7)	11.1% (7.0-16.2)	14.2% (8.5-21.4)	15.7% (8.2-25.7)
Venezuela	7.0% (2.8-14.3)	8.1% (4.8-12.6)	9.2% (6.3-12.8)	9.7% (6.4-13.6)	9.9% (5.3-15.7)
Viet Nam	2.6% (0.7-6.0)	3.2% (1.6-5.5)	4.1% (2.5-6.1)	5.2% (3.3-7.5)	5.5% (2.9-9.2)
Yemen	3.3% (1.0-7.8)	4.9% (2.4-8.3)	7.4% (4.5-11.3)	11.0% (6.7-16.6)	12.6% (6.7-20.6)
Zambia	3.2% (1.0-7.3)	4.1% (2.1-7.1)	4.9% (2.8-7.6)	5.9% (3.6-9.0)	6.5% (3.3-11.1)
Zimbabwe	2.9% (0.8-7.1)	3.9% (1.8-6.7)	5.2% (3.0-8.2)	6.1% (3.5-9.6)	6.5% (3.1-11.6)

Country (Women)	Year				
	1980	1990	2000	2010	2014
Afghanistan	5.4% (2.0-11.3)	6.9% (4.0-10.9)	8.5% (5.7-11.9)	11.1% (7.2-15.8)	12.2% (6.8-18.8)
Albania	5.7% (1.9-12.5)	5.9% (2.9-10.2)	6.2% (3.7-9.5)	6.9% (4.2-10.4)	7.1% (3.5-12.2)
Algeria	5.6% (2.1-11.5)	7.1% (4.3-10.6)	9.2% (6.5-12.3)	11.6% (8.3-15.8)	12.6% (7.7-18.9)
American Samoa	20.1% (11.0-31.0)	24.9% (17.7-32.6)	29.4% (22.2-37.4)	32.0% (22.8-42.5)	32.9% (21.3-46.6)
Andorra	7.2% (3.0-13.5)	6.9% (4.4-10.4)	6.4% (4.3-9.1)	6.0% (3.7-8.8)	5.8% (2.9-9.9)
Angola	2.9% (0.8-7.3)	3.9% (1.8-7.5)	5.2% (2.7-9.1)	7.1% (3.8-12.6)	7.8% (3.6-15.1)
Antigua and Barbuda	5.6% (1.9-12.3)	7.0% (3.7-11.4)	8.8% (5.3-13.3)	11.6% (6.8-18.3)	13.0% (6.3-22.5)
Argentina	6.1% (2.2-12.9)	6.8% (3.8-10.9)	7.7% (4.9-11.1)	8.8% (5.6-12.9)	9.5% (5.1-15.5)
Armenia	6.8% (2.3-14.4)	7.9% (4.2-13.0)	9.0% (5.5-13.8)	11.1% (7.1-16.2)	12.0% (6.6-19.1)
Australia	4.2% (1.9-7.9)	4.5% (2.9-6.7)	5.0% (3.5-6.9)	5.0% (3.5-6.9)	5.0% (2.9-7.9)
Austria	3.7% (1.5-7.6)	3.5% (2.1-5.6)	3.5% (2.3-5.1)	3.3% (2.1-5.0)	3.2% (1.6-5.7)
Azerbaijan	6.2% (2.1-13.3)	7.3% (3.8-12.1)	8.5% (5.1-13.1)	11.6% (7.4-16.6)	13.0% (7.2-20.3)
Bahamas	8.0% (3.1-16.0)	9.5% (5.6-15.0)	11.3% (7.5-16.4)	12.9% (8.2-19.5)	13.7% (7.2-22.6)
Bahrain	9.6% (3.6-18.1)	9.5% (5.9-13.8)	9.9% (7.1-13.5)	10.4% (7.1-14.4)	10.6% (6.1-16.7)
Bangladesh	3.8% (1.3-8.5)	5.0% (2.8-8.2)	6.6% (4.4-9.4)	8.5% (5.6-12.2)	9.3% (5.2-14.6)
Barbados	7.2% (2.7-14.8)	8.4% (4.8-13.5)	10.2% (6.5-15.0)	12.7% (7.7-19.0)	13.7% (7.2-22.8)
Belarus	5.8% (2.0-12.6)	6.4% (3.3-10.6)	6.9% (3.9-10.8)	7.4% (4.1-11.7)	7.5% (3.5-13.3)
Belgium	5.4% (2.2-10.8)	4.9% (2.9-7.5)	4.3% (2.8-6.1)	3.7% (2.3-5.3)	3.5% (1.8-5.8)
Belize	7.6% (2.8-15.3)	9.0% (5.3-14.1)	11.3% (7.5-16.1)	14.0% (8.9-20.5)	15.2% (8.2-24.5)
Benin	2.7% (0.7-6.6)	3.6% (1.8-6.5)	5.0% (2.9-7.7)	6.4% (3.8-9.6)	7.0% (3.6-11.6)
Bermuda	11.2% (4.0-23.0)	11.7% (6.2-19.3)	12.3% (7.6-18.4)	14.5% (8.8-21.9)	15.4% (7.9-25.7)
Bhutan	4.6% (1.6-10.0)	6.5% (3.7-10.3)	8.9% (6.0-12.4)	10.9% (7.4-14.9)	11.5% (6.9-17.1)
Bolivia	4.9% (1.5-11.3)	5.8% (2.9-9.8)	7.0% (4.0-10.5)	8.3% (4.6-12.5)	8.9% (4.1-15.1)
Bosnia and Herzegovina	5.2% (1.6-12.2)	5.4% (2.5-9.5)	6.5% (3.5-10.3)	6.8% (4.1-10.4)	6.9% (3.5-11.6)
Botswana	3.8% (1.1-9.1)	5.7% (2.9-9.6)	7.5% (4.6-11.3)	8.8% (5.5-13.0)	9.5% (5.2-15.2)
Brazil	5.6% (2.1-11.5)	6.3% (3.7-9.8)	7.0% (4.7-9.8)	8.1% (5.1-11.9)	8.7% (4.6-14.4)
Brunei Darussalam	8.8% (3.7-16.8)	9.3% (5.7-14.3)	10.1% (6.5-14.6)	9.9% (6.1-14.6)	9.7% (5.0-15.9)
Bulgaria	5.7% (2.1-11.6)	5.7% (3.2-9.0)	5.9% (3.7-8.7)	6.6% (4.0-9.9)	6.9% (3.3-11.8)
Burkina Faso	2.3% (0.6-5.9)	2.9% (1.3-5.5)	3.9% (2.1-6.3)	5.0% (2.8-8.0)	5.5% (2.6-9.6)
Burundi	1.5% (0.3-4.4)	2.1% (0.7-4.5)	2.8% (1.3-5.3)	3.6% (1.8-6.3)	4.1% (1.8-7.7)
Cabo Verde	3.2% (0.9-7.8)	4.4% (2.2-7.7)	6.1% (3.8-9.0)	7.6% (4.7-11.4)	8.0% (4.1-13.5)
Cambodia	3.3% (1.0-8.0)	4.4% (2.2-7.4)	5.4% (3.3-8.0)	6.5% (4.1-9.5)	6.9% (3.6-11.4)
Cameroon	3.1% (0.9-7.6)	3.8% (1.9-6.5)	4.8% (3.0-7.1)	6.2% (3.8-9.3)	6.9% (3.5-11.8)
Canada	4.0% (1.7-7.7)	4.4% (2.7-6.6)	4.7% (3.1-6.8)	4.8% (3.1-6.8)	4.8% (2.5-7.9)
Central African Republic	2.9% (0.8-7.4)	3.7% (1.6-7.2)	4.9% (2.5-8.7)	6.7% (3.6-12.1)	7.6% (3.5-14.8)
Chad	2.5% (0.7-6.3)	3.0% (1.3-5.7)	4.1% (2.2-6.7)	5.5% (3.1-8.9)	6.2% (2.9-11.0)
Chile	7.8% (3.0-15.6)	8.1% (4.9-12.3)	8.9% (6.2-12.1)	10.1% (6.7-14.2)	10.8% (6.1-17.0)
China	5.0% (1.6-11.3)	5.4% (3.0-8.6)	6.2% (4.4-8.2)	7.3% (4.9-10.1)	7.6% (4.3-12.2)
China (Hong Kong SAR)	7.6% (2.8-15.7)	7.3% (4.0-11.8)	6.8% (4.0-10.5)	6.0% (3.1-10.3)	5.8% (2.3-11.5)
Colombia	5.6% (2.2-11.6)	6.6% (3.8-10.4)	7.6% (5.2-10.6)	8.4% (5.5-11.9)	8.7% (4.6-14.1)
Comoros	3.2% (0.9-7.8)	4.2% (2.1-7.3)	5.6% (3.3-8.7)	7.2% (4.5-10.7)	8.0% (4.3-13.0)
Congo	3.5% (1.0-8.5)	4.6% (2.1-9.1)	5.7% (3.0-10.3)	7.0% (3.6-12.7)	7.6% (3.4-15.0)

Country (Women)	Year				
	1980	1990	2000	2010	2014
Cook Islands	12.1% (6.4-19.3)	17.3% (12.1-23.0)	22.1% (16.6-28.3)	25.7% (18.3-34.0)	26.7% (16.8-38.2)
Costa Rica	4.4% (1.6-9.8)	5.5% (3.2-8.9)	7.0% (4.7-10.0)	8.4% (5.5-11.9)	8.9% (4.8-14.4)
Cote d'Ivoire	3.3% (1.0-7.9)	4.0% (1.9-7.0)	4.9% (2.9-7.6)	5.9% (3.4-9.1)	6.3% (3.1-10.9)
Croatia	4.9% (1.8-10.5)	5.3% (2.9-8.5)	5.5% (3.4-8.2)	6.0% (3.7-9.1)	6.3% (3.2-10.8)
Cuba	6.2% (2.3-12.9)	7.2% (4.1-11.7)	7.9% (5.1-11.8)	9.0% (5.4-14.0)	9.6% (4.8-16.6)
Cyprus	3.9% (1.4-8.2)	4.8% (2.8-7.6)	5.5% (3.6-7.9)	5.6% (3.5-8.5)	5.7% (2.8-9.8)
Czech Republic	7.3% (3.1-13.7)	6.8% (4.0-10.6)	6.5% (4.2-9.4)	6.5% (3.9-10.0)	6.6% (3.3-11.4)
Denmark	4.5% (1.8-9.1)	4.2% (2.5-6.5)	3.8% (2.5-5.6)	3.4% (2.1-5.1)	3.3% (1.7-5.6)
Djibouti	5.7% (2.1-11.8)	6.4% (3.4-10.8)	7.3% (4.2-11.5)	7.5% (4.2-12.3)	7.6% (3.6-13.5)
Dominica	6.3% (2.3-13.3)	8.3% (4.9-13.0)	10.2% (6.6-14.9)	12.5% (7.8-19.1)	13.6% (7.4-22.8)
Dominican Republic	4.6% (1.6-10.2)	5.9% (3.3-9.7)	8.2% (5.3-11.9)	10.6% (6.6-16.1)	11.4% (6.1-19.6)
DR Congo	2.6% (0.7-6.6)	3.2% (1.4-6.4)	4.1% (2.1-7.5)	5.4% (2.8-9.8)	6.1% (2.7-12.1)
Ecuador	4.5% (1.4-10.6)	5.5% (2.7-9.4)	6.8% (3.9-10.1)	7.8% (4.3-12.0)	8.5% (3.7-14.9)
Egypt	8.0% (3.1-15.6)	10.2% (6.4-14.8)	13.3% (9.6-17.5)	17.6% (13.0-23.2)	19.8% (12.9-28.2)
El Salvador	5.5% (1.9-11.5)	6.7% (3.8-10.8)	8.7% (5.7-12.4)	10.0% (6.5-14.2)	10.7% (5.7-17.1)
Equatorial Guinea	2.9% (0.8-7.2)	3.8% (1.7-7.4)	5.8% (3.1-10.1)	8.3% (4.6-14.4)	9.2% (4.3-17.3)
Eritrea	2.4% (0.6-6.0)	2.9% (1.3-5.4)	3.8% (2.1-6.2)	4.6% (2.6-7.3)	5.0% (2.5-9.0)
Estonia	6.5% (2.3-13.8)	6.4% (3.3-10.6)	6.3% (3.6-9.8)	6.4% (3.5-10.5)	6.5% (2.9-12.0)
Ethiopia	2.5% (0.7-5.9)	3.0% (1.3-5.6)	3.8% (2.0-6.3)	4.6% (2.6-7.4)	5.0% (2.4-8.9)
Fiji	10.5% (4.8-19.0)	11.5% (7.0-17.7)	14.0% (9.1-20.8)	17.4% (11.1-25.1)	18.9% (10.5-29.1)
Finland	4.3% (1.8-8.7)	4.6% (2.8-7.0)	4.6% (3.2-6.2)	4.4% (2.9-6.2)	4.3% (2.3-7.1)
France	4.7% (1.9-9.0)	4.5% (2.6-7.0)	4.5% (2.9-6.4)	4.4% (2.8-6.6)	4.4% (2.3-7.5)
French Polynesia	11.4% (5.5-19.8)	14.1% (9.0-20.5)	16.6% (11.4-22.9)	18.4% (12.4-25.8)	19.2% (11.3-29.1)
Gabon	3.9% (1.2-9.3)	5.4% (2.5-10.2)	7.4% (3.9-13.0)	9.2% (4.8-16.3)	10.0% (4.5-19.2)
Gambia	3.2% (0.9-7.6)	4.1% (2.0-7.2)	5.6% (3.4-8.6)	7.2% (4.3-11.0)	7.9% (4.0-13.5)
Georgia	5.6% (1.8-12.2)	6.7% (3.4-11.6)	7.8% (4.5-12.1)	10.6% (6.7-15.4)	12.1% (6.8-18.8)
Germany	4.2% (1.7-8.7)	4.1% (2.5-6.2)	4.1% (2.8-5.6)	3.9% (2.6-5.7)	3.9% (2.1-6.5)
Ghana	3.0% (0.9-7.4)	3.8% (1.9-6.8)	5.0% (2.9-8.0)	6.1% (3.6-9.7)	6.6% (3.4-11.2)
Greece	6.0% (2.4-11.8)	5.9% (3.5-9.1)	5.9% (3.8-8.7)	6.0% (3.6-9.0)	6.0% (3.0-10.4)
Greenland	5.0% (2.1-9.9)	5.0% (3.0-7.7)	4.9% (3.3-6.8)	4.7% (3.0-6.6)	4.6% (2.4-7.6)
Grenada	5.1% (1.8-11.2)	6.5% (3.6-10.6)	8.8% (5.6-12.9)	11.9% (7.2-18.2)	13.3% (6.7-22.1)
Guatemala	5.0% (1.7-11.0)	6.1% (3.2-10.2)	7.8% (4.9-11.5)	9.6% (6.0-14.1)	10.4% (5.4-17.2)
Guinea	2.5% (0.7-6.4)	3.3% (1.5-6.0)	4.3% (2.5-6.8)	5.5% (3.3-8.4)	6.1% (3.1-10.3)
Guinea Bissau	2.8% (0.8-6.9)	3.6% (1.7-6.5)	4.9% (2.9-7.6)	6.3% (3.8-9.6)	6.8% (3.4-11.8)
Guyana	5.0% (1.6-11.3)	6.2% (3.2-10.5)	8.3% (5.0-12.8)	11.2% (6.6-17.4)	12.6% (6.3-21.5)
Haiti	3.0% (0.8-7.3)	4.0% (1.9-7.1)	5.7% (3.2-9.0)	7.8% (4.4-12.7)	8.8% (4.3-15.9)
Honduras	3.9% (1.2-8.9)	5.1% (2.7-8.7)	6.8% (4.3-9.9)	9.0% (5.6-13.0)	10.0% (5.2-16.6)
Hungary	6.0% (2.4-12.2)	5.9% (3.3-9.4)	5.9% (3.7-8.8)	6.4% (3.9-9.7)	6.7% (3.4-11.5)
Iceland	5.2% (2.0-10.9)	5.2% (2.7-8.8)	4.8% (3.0-7.2)	4.3% (2.5-6.7)	4.2% (2.0-7.3)
India	4.6% (1.7-9.5)	6.0% (3.6-9.1)	7.3% (5.2-10.0)	8.1% (5.5-11.3)	8.3% (4.8-12.8)
Indonesia	4.1% (1.5-9.0)	5.4% (3.1-8.6)	6.9% (4.6-9.7)	7.7% (5.1-10.8)	8.0% (4.3-12.6)
Iran	6.0% (2.3-12.3)	6.8% (4.3-9.7)	8.5% (6.4-10.7)	11.5% (8.8-14.9)	12.9% (8.4-18.8)

Country (Women)	Year				
	**1980**	**1990**	**2000**	**2010**	**2014**
Iraq	8.7% (3.6-16.6)	10.5% (6.8-15.0)	12.4% (9.1-16.2)	15.7% (11.3-21.0)	17.5% (11.1-25.4)
Ireland	3.3% (1.3-7.0)	3.9% (2.2-6.4)	4.6% (2.9-6.8)	5.0% (3.2-7.2)	5.1% (2.7-8.5)
Israel	5.8% (2.4-11.0)	6.1% (3.8-9.1)	6.1% (4.0-8.8)	5.8% (3.5-8.8)	5.8% (2.8-10.0)
Italy	4.9% (2.1-9.4)	4.8% (3.1-7.1)	4.7% (3.4-6.4)	4.6% (3.1-6.6)	4.6% (2.5-7.6)
Jamaica	5.7% (2.0-12.1)	7.1% (4.1-11.2)	9.7% (6.5-13.8)	12.9% (8.3-18.9)	14.4% (7.8-23.3)
Japan	5.3% (2.5-9.3)	5.4% (3.9-7.3)	5.6% (4.3-7.1)	5.3% (3.7-7.1)	5.0% (3.0-7.7)
Jordan	9.2% (3.7-17.3)	11.6% (7.6-16.2)	14.0% (10.5-18.0)	16.2% (12.3-20.9)	17.2% (11.3-24.6)
Kazakhstan	6.2% (2.1-13.3)	7.5% (3.9-12.3)	8.8% (5.4-13.5)	10.6% (6.7-15.4)	11.4% (6.2-18.2)
Kenya	2.7% (0.8-6.6)	3.5% (1.7-6.2)	4.5% (2.7-7.0)	5.6% (3.4-8.5)	6.2% (3.1-10.4)
Kiribati	6.8% (3.0-12.2)	11.1% (6.9-16.1)	16.4% (11.4-22.2)	21.0% (14.4-28.8)	22.6% (13.5-33.5)
Kuwait	13.2% (5.3-24.1)	13.7% (8.4-20.0)	15.6% (11.4-20.4)	18.3% (13.4-24.2)	19.6% (12.9-27.7)
Kyrgyzstan	4.2% (1.3-9.5)	5.4% (2.6-9.5)	7.0% (4.0-10.9)	9.5% (5.9-13.9)	10.8% (5.9-17.3)
Lao PDR	3.9% (1.2-8.9)	4.7% (2.4-7.9)	6.0% (3.7-9.0)	7.2% (4.7-10.3)	7.6% (4.2-12.0)
Latvia	6.3% (2.2-13.6)	6.5% (3.3-10.8)	6.4% (3.7-9.8)	6.5% (3.6-10.4)	6.6% (2.9-12.1)
Lebanon	6.7% (2.7-13.1)	7.5% (4.6-11.2)	8.4% (5.3-12.2)	10.8% (7.3-15.2)	12.2% (7.4-18.5)
Lesotho	4.8% (1.4-11.6)	5.9% (2.8-10.4)	7.7% (4.4-11.9)	9.2% (5.4-14.0)	9.9% (5.0-16.3)
Liberia	3.5% (1.1-8.2)	4.7% (2.4-7.9)	4.9% (3.0-7.5)	6.7% (4.2-9.9)	7.6% (4.1-12.7)
Libya	9.1% (3.7-17.1)	10.4% (6.6-14.8)	12.2% (9.1-15.8)	15.4% (11.6-20.1)	16.6% (10.7-23.8)
Lithuania	7.4% (2.5-15.3)	7.4% (3.9-12.2)	7.0% (4.0-10.6)	6.9% (3.9-11.0)	7.1% (3.3-12.6)
Luxembourg	4.2% (1.6-9.0)	4.1% (2.1-6.9)	4.0% (2.4-6.1)	3.9% (2.4-5.8)	3.9% (2.0-6.6)
Macedonia (TFYR)	6.2% (2.3-12.5)	6.3% (3.4-10.0)	6.5% (3.9-9.5)	6.6% (4.0-10.0)	6.8% (3.4-11.8)
Madagascar	2.6% (0.8-6.2)	3.2% (1.5-5.6)	3.9% (2.2-6.2)	4.5% (2.6-7.0)	4.8% (2.4-8.3)
Malawi	2.7% (0.8-6.8)	3.4% (1.6-6.2)	4.5% (2.6-7.2)	5.5% (3.4-8.3)	6.0% (3.1-9.9)
Malaysia	5.6% (2.2-11.5)	7.1% (4.4-10.7)	9.1% (6.3-12.7)	10.4% (7.1-14.5)	10.7% (6.2-16.7)
Maldives	5.0% (1.7-10.8)	7.0% (3.8-11.5)	8.8% (5.0-14.0)	10.5% (5.8-16.6)	10.7% (5.3-18.4)
Mali	2.6% (0.7-6.4)	3.3% (1.5-6.1)	4.4% (2.4-7.1)	5.8% (3.3-9.3)	6.4% (3.0-11.5)
Malta	7.6% (3.4-13.7)	7.2% (4.6-10.7)	6.8% (4.7-9.8)	6.5% (4.1-9.7)	6.4% (3.3-10.8)
Marshall Islands	10.7% (5.2-18.2)	14.4% (9.8-19.8)	18.2% (13.4-23.9)	20.7% (14.2-28.2)	21.5% (12.6-31.9)
Mauritania	4.0% (1.2-9.3)	5.6% (2.8-9.4)	7.3% (4.4-11.2)	8.8% (5.2-13.4)	9.4% (4.7-15.8)
Mauritius	8.9% (4.0-15.9)	10.3% (6.7-14.6)	11.7% (8.3-15.8)	12.6% (8.3-17.8)	13.1% (7.5-20.1)
Mexico	6.5% (2.5-13.2)	7.8% (4.7-12.0)	9.6% (6.7-13.2)	10.9% (7.4-15.3)	11.5% (6.4-18.3)
Micronesia (Federated States of)	9.2% (4.1-17.0)	12.5% (8.1-17.8)	16.8% (12.1-22.1)	21.4% (15.3-28.7)	23.4% (14.6-34.0)
Moldova	5.7% (1.8-12.4)	6.3% (3.1-10.7)	6.9% (3.7-11.0)	8.1% (4.4-12.7)	8.4% (4.0-14.4)
Mongolia	5.0% (1.6-11.0)	6.5% (3.3-11.1)	7.8% (4.5-12.1)	10.0% (6.4-14.5)	11.2% (6.4-17.4)
Montenegro	5.3% (1.7-11.5)	6.0% (3.1-9.8)	6.3% (3.9-9.4)	6.6% (3.9-9.9)	6.6% (3.2-11.3)
Morocco	5.4% (2.0-11.5)	7.0% (4.2-10.5)	9.2% (6.4-12.6)	12.1% (8.3-16.8)	13.4% (8.1-20.5)
Mozambique	2.6% (0.7-6.6)	3.6% (1.7-6.3)	4.8% (2.8-7.5)	5.8% (3.5-8.8)	6.2% (3.2-10.7)
Myanmar	4.0% (1.4-8.9)	4.8% (2.6-7.9)	6.0% (3.9-8.6)	7.4% (4.9-10.5)	7.9% (4.4-12.5)
Namibia	3.8% (1.2-8.9)	4.5% (2.3-7.7)	5.8% (3.4-8.9)	7.1% (4.3-10.9)	7.5% (3.9-12.7)
Nauru	25.4% (14.2-38.5)	27.7% (19.4-36.9)	28.4% (20.9-36.7)	28.2% (19.3-38.7)	28.4% (17.0-41.9)
Nepal	3.6% (1.2-8.4)	4.8% (2.6-8.1)	6.7% (4.5-9.4)	8.7% (5.9-12.2)	9.5% (5.5-14.8)
Netherlands	3.7% (1.4-7.5)	3.7% (2.2-5.7)	3.8% (2.6-5.3)	3.6% (2.4-5.2)	3.5% (1.9-5.8)

Country (Women)	Year				
	1980	1990	2000	2010	2014
New Zealand	4.7% (2.0-8.9)	5.1% (3.1-7.7)	5.7% (3.9-7.9)	5.9% (4.0-8.3)	6.0% (3.4-9.6)
Nicaragua	5.8% (2.1-12.1)	6.9% (3.9-11.0)	8.5% (5.5-12.2)	10.2% (6.5-14.8)	11.0% (5.8-17.8)
Niger	2.5% (0.7-6.4)	3.0% (1.3-5.7)	4.0% (2.1-6.5)	4.9% (2.6-7.9)	5.3% (2.4-9.5)
Nigeria	3.2% (0.9-7.7)	4.0% (2.0-6.9)	4.8% (2.9-7.4)	5.7% (3.5-8.6)	6.0% (3.1-10.3)
Niue	10.5% (5.0-17.9)	15.5% (10.4-21.4)	20.5% (15.0-26.6)	25.5% (18.3-33.2)	27.3% (17.6-38.3)
North Korea	6.5% (2.5-13.2)	6.1% (3.5-9.5)	5.8% (3.4-9.0)	5.8% (3.0-9.8)	5.9% (2.6-11.3)
Norway	3.8% (1.5-7.7)	4.0% (2.3-6.2)	4.2% (2.8-5.9)	4.0% (2.6-5.8)	3.9% (2.0-6.5)
Occupied Palestinian Territory	7.4% (3.0-13.9)	9.1% (6.0-12.9)	11.8% (8.9-15.2)	15.6% (11.6-20.3)	17.5% (11.4-24.9)
Oman	5.8% (2.2-11.6)	8.0% (5.0-11.5)	9.9% (7.3-13.1)	11.6% (8.3-15.6)	12.3% (7.4-18.4)
Pakistan	5.9% (2.4-11.9)	7.2% (4.4-10.7)	9.0% (6.3-12.2)	11.2% (7.5-15.6)	12.1% (7.0-18.6)
Palau	12.9% (6.6-21.3)	15.8% (10.8-21.4)	18.5% (13.4-24.6)	20.8% (14.2-28.8)	21.6% (12.8-32.4)
Panama	5.1% (1.8-10.9)	6.2% (3.6-9.9)	7.7% (5.1-11.0)	9.1% (6.0-12.9)	9.8% (5.3-15.6)
Papua New Guinea	5.5% (1.9-12.1)	7.3% (4.0-11.7)	9.8% (6.2-14.5)	12.8% (7.5-19.7)	14.3% (7.1-24.3)
Paraguay	3.6% (1.2-8.0)	4.6% (2.5-7.7)	6.0% (3.7-8.9)	7.5% (4.5-11.2)	8.3% (4.2-14.1)
Peru	5.9% (2.0-13.2)	6.6% (3.4-10.9)	7.1% (4.2-10.4)	7.6% (4.3-11.4)	8.1% (3.8-13.7)
Philippines	4.3% (1.6-9.0)	5.4% (3.2-8.5)	6.4% (4.3-9.0)	7.0% (4.8-9.8)	7.3% (4.2-11.3)
Poland	6.2% (2.5-12.5)	6.2% (3.6-9.6)	6.3% (4.2-9.0)	6.7% (4.3-9.7)	7.0% (3.7-11.5)
Portugal	4.6% (1.7-9.9)	5.0% (2.8-8.0)	5.2% (3.3-7.5)	5.2% (3.2-8.0)	5.3% (2.7-9.2)
Puerto Rico	7.3% (2.8-14.8)	9.9% (5.7-15.7)	11.5% (7.7-16.9)	12.9% (8.0-19.6)	13.4% (7.1-22.4)
Qatar	11.6% (4.6-21.3)	12.3% (7.9-17.4)	14.2% (10.4-18.5)	17.3% (12.9-22.8)	18.8% (12.2-26.8)
Romania	5.3% (1.9-11.0)	5.3% (3.0-8.3)	5.6% (3.5-8.1)	6.1% (3.6-9.2)	6.4% (3.1-10.9)
Russian Federation	7.4% (2.7-15.5)	7.4% (3.9-12.0)	7.5% (4.4-11.4)	7.8% (4.4-12.3)	8.0% (3.7-13.8)
Rwanda	1.6% (0.4-4.7)	2.2% (0.8-4.8)	3.2% (1.5-5.9)	4.2% (2.2-7.0)	4.5% (2.1-8.1)
Saint Kitts and Nevis	7.2% (2.6-15.2)	8.9% (5.0-14.2)	11.2% (7.1-16.4)	14.7% (8.8-22.1)	16.3% (8.4-26.6)
Saint Lucia	4.8% (1.6-10.9)	6.5% (3.5-10.8)	9.1% (5.6-13.7)	13.0% (7.3-20.0)	15.1% (7.2-25.9)
Saint Vincent and the Grenadines	5.3% (1.8-11.7)	6.8% (3.8-11.2)	9.1% (5.9-13.3)	11.1% (6.9-16.6)	11.8% (6.1-19.9)
Samoa	9.0% (3.8-16.7)	12.8% (8.0-18.5)	18.7% (13.5-24.7)	24.3% (16.6-32.8)	26.6% (16.2-38.6)
Sao Tome and Principe	4.1% (1.3-9.6)	5.3% (2.7-9.3)	6.6% (4.0-10.2)	7.4% (4.4-11.2)	7.8% (3.9-13.2)
Saudi Arabia	8.7% (3.6-16.2)	10.6% (6.9-15.0)	13.1% (9.8-17.0)	15.7% (11.7-20.6)	17.0% (11.1-24.4)
Senegal	4.0% (1.2-9.2)	4.8% (2.4-8.5)	5.8% (3.4-9.0)	6.9% (4.1-10.5)	7.3% (3.7-12.6)
Serbia	5.4% (1.9-11.4)	5.7% (3.0-9.1)	5.8% (3.6-8.6)	6.2% (3.7-9.5)	6.5% (3.2-11.1)
Seychelles	6.4% (2.4-12.8)	7.5% (4.2-11.7)	8.8% (5.5-13.0)	10.0% (6.3-14.7)	10.4% (5.6-16.7)
Sierra Leone	3.5% (1.1-8.2)	4.1% (2.0-7.2)	5.0% (2.9-7.7)	6.1% (3.6-9.4)	6.6% (3.3-11.6)
Singapore	7.9% (3.7-13.8)	8.2% (5.4-11.6)	7.8% (5.5-10.5)	6.7% (4.6-9.4)	6.5% (3.7-10.3)
Slovakia	5.1% (1.8-10.8)	5.5% (3.1-8.5)	5.6% (3.6-8.0)	6.1% (3.8-8.9)	6.3% (3.2-10.8)
Slovenia	5.5% (2.0-11.3)	5.9% (3.3-9.3)	6.2% (3.9-9.1)	6.7% (4.0-10.1)	7.0% (3.4-12.1)
Solomon Islands	6.5% (2.3-14.1)	8.4% (4.5-13.7)	11.0% (6.9-16.0)	13.7% (8.4-20.5)	15.1% (7.9-25.0)
Somalia	3.2% (1.0-7.5)	4.0% (2.0-7.0)	4.9% (2.7-7.9)	5.8% (3.3-9.4)	6.2% (3.1-11.1)
South Africa	7.7% (2.9-15.5)	8.4% (4.9-12.9)	9.9% (6.4-14.2)	11.8% (7.5-17.3)	12.6% (6.8-20.2)
South Korea	4.4% (1.8-8.8)	6.2% (3.8-9.3)	6.8% (4.5-9.8)	6.5% (4.4-8.9)	6.7% (3.9-10.4)
Spain	6.1% (2.5-11.5)	6.0% (3.8-8.9)	6.0% (4.2-8.1)	5.8% (3.8-8.3)	5.7% (3.1-9.3)
Sri Lanka	4.0% (1.3-9.0)	4.7% (2.6-7.8)	6.0% (3.9-8.6)	7.1% (4.6-10.5)	7.7% (4.0-12.8)

Country (Women)	Year				
	1980	1990	2000	2010	2014
Sudan	4.3% (1.4-9.8)	5.7% (3.0-9.4)	7.7% (4.8-11.5)	9.0% (5.5-13.4)	9.5% (5.0-15.6)
Suriname	7.8% (3.0-16.1)	9.2% (5.4-14.5)	10.5% (7.0-15.1)	12.6% (8.0-18.9)	13.5% (7.3-22.6)
Swaziland	5.1% (1.6-11.7)	6.7% (3.4-11.1)	8.6% (5.1-12.8)	10.4% (6.3-15.2)	11.3% (6.1-18.1)
Sweden	4.4% (1.8-9.0)	4.1% (2.6-6.2)	4.0% (2.8-5.6)	4.0% (2.6-5.6)	4.0% (2.1-6.6)
Switzerland	3.0% (1.1-6.4)	3.2% (1.8-5.1)	3.2% (2.0-4.7)	2.9% (1.8-4.3)	2.8% (1.4-4.8)
Syrian Arab Republic	6.8% (2.7-13.3)	8.3% (5.2-12.1)	10.9% (8.1-14.2)	13.9% (10.2-18.5)	15.3% (9.6-22.4)
Taiwan	5.7% (2.2-11.5)	6.3% (3.9-9.4)	6.0% (4.1-8.5)	5.9% (3.5-9.2)	6.0% (2.8-10.5)
Tajikistan	4.3% (1.3-9.8)	5.0% (2.4-8.8)	6.0% (3.3-9.5)	8.6% (5.1-13.1)	9.9% (5.1-16.4)
Tanzania	2.7% (0.8-6.3)	3.5% (1.8-6.1)	4.7% (2.8-7.2)	5.7% (3.6-8.4)	6.1% (3.3-9.9)
Thailand	5.3% (2.0-11.2)	6.2% (3.7-9.5)	7.3% (5.1-10.0)	8.4% (5.7-11.6)	8.8% (4.9-13.5)
Timor-Leste	3.0% (0.9-7.1)	4.0% (2.0-6.8)	5.0% (2.9-7.7)	5.3% (3.3-7.9)	5.5% (2.9-9.0)
Togo	2.8% (0.8-7.1)	3.6% (1.7-6.4)	4.9% (2.9-7.5)	6.3% (3.9-9.4)	7.0% (3.6-11.6)
Tokelau	9.1% (3.4-18.2)	13.4% (7.9-20.7)	19.0% (13.0-25.7)	24.8% (16.7-34.2)	27.3% (16.3-40.2)
Tonga	10.4% (4.5-19.3)	14.3% (9.1-20.6)	19.2% (13.7-25.0)	24.2% (17.1-32.3)	26.4% (16.4-37.6)
Trinidad and Tobago	4.9% (1.4-11.9)	5.8% (2.8-10.3)	8.1% (4.8-12.6)	11.4% (6.3-18.3)	13.0% (6.0-22.7)
Tunisia	6.0% (2.3-11.8)	7.7% (4.9-11.1)	9.7% (7.2-12.7)	11.9% (8.7-15.6)	12.9% (7.9-19.0)
Turkey	6.0% (2.3-12.2)	7.9% (5.0-11.2)	10.0% (7.5-12.7)	13.0% (9.9-16.3)	14.2% (9.5-19.6)
Turkmenistan	4.8% (1.5-10.8)	5.5% (2.8-9.6)	7.0% (4.0-11.0)	10.2% (6.6-14.7)	12.0% (6.9-18.8)
Tuvalu	9.9% (4.4-17.6)	14.2% (9.6-19.5)	19.1% (14.3-24.7)	22.9% (16.5-30.4)	24.3% (15.4-35.1)
Uganda	2.1% (0.5-5.5)	2.5% (1.0-5.2)	3.3% (1.6-6.0)	4.2% (2.3-7.0)	4.7% (2.3-8.2)
Ukraine	6.3% (2.2-13.4)	6.6% (3.4-11.0)	6.7% (3.8-10.4)	7.1% (3.9-11.3)	7.2% (3.3-13.0)
United Arab Emirates	10.4% (4.2-19.3)	10.3% (6.3-15.2)	12.4% (9.1-16.5)	14.9% (11.1-19.7)	15.4% (9.7-22.6)
United Kingdom	4.0% (1.8-7.6)	4.4% (2.8-6.5)	4.8% (3.4-6.6)	4.9% (3.6-6.5)	4.9% (3.1-7.4)
United States of America	4.3% (2.1-8.1)	4.9% (3.3-6.9)	5.7% (4.3-7.5)	6.2% (4.4-8.4)	6.4% (3.8-9.9)
Uruguay	7.3% (2.8-14.9)	7.6% (4.4-11.6)	8.1% (5.4-11.5)	9.1% (5.9-13.2)	9.8% (5.3-15.9)
Uzbekistan	4.4% (1.4-9.8)	5.3% (2.6-9.3)	6.7% (3.9-10.4)	9.2% (5.7-13.7)	10.7% (5.9-16.8)
Vanuatu	7.2% (2.7-15.1)	8.6% (4.9-13.7)	11.1% (7.1-16.1)	14.4% (8.9-21.4)	16.0% (8.4-25.9)
Venezuela	6.9% (2.7-13.8)	7.6% (4.5-11.8)	8.5% (5.8-11.7)	8.8% (5.8-12.4)	9.0% (4.8-14.4)
Viet Nam	3.3% (1.0-7.4)	4.0% (2.1-6.6)	4.8% (3.0-7.0)	5.1% (3.3-7.2)	5.1% (2.7-8.2)
Yemen	3.1% (1.0-7.5)	4.2% (2.1-7.3)	6.2% (3.6-9.4)	8.9% (5.2-13.7)	10.1% (5.3-17.0)
Zambia	4.0% (1.3-8.9)	4.7% (2.5-7.9)	5.4% (3.2-8.4)	6.2% (3.9-9.4)	6.7% (3.5-11.1)
Zimbabwe	5.1% (1.6-11.6)	5.7% (2.9-9.6)	6.7% (4.1-10.1)	7.2% (4.4-10.9)	7.6% (3.9-12.7)

Appendix Figure 1: Flowchart of secondary search for data sources.

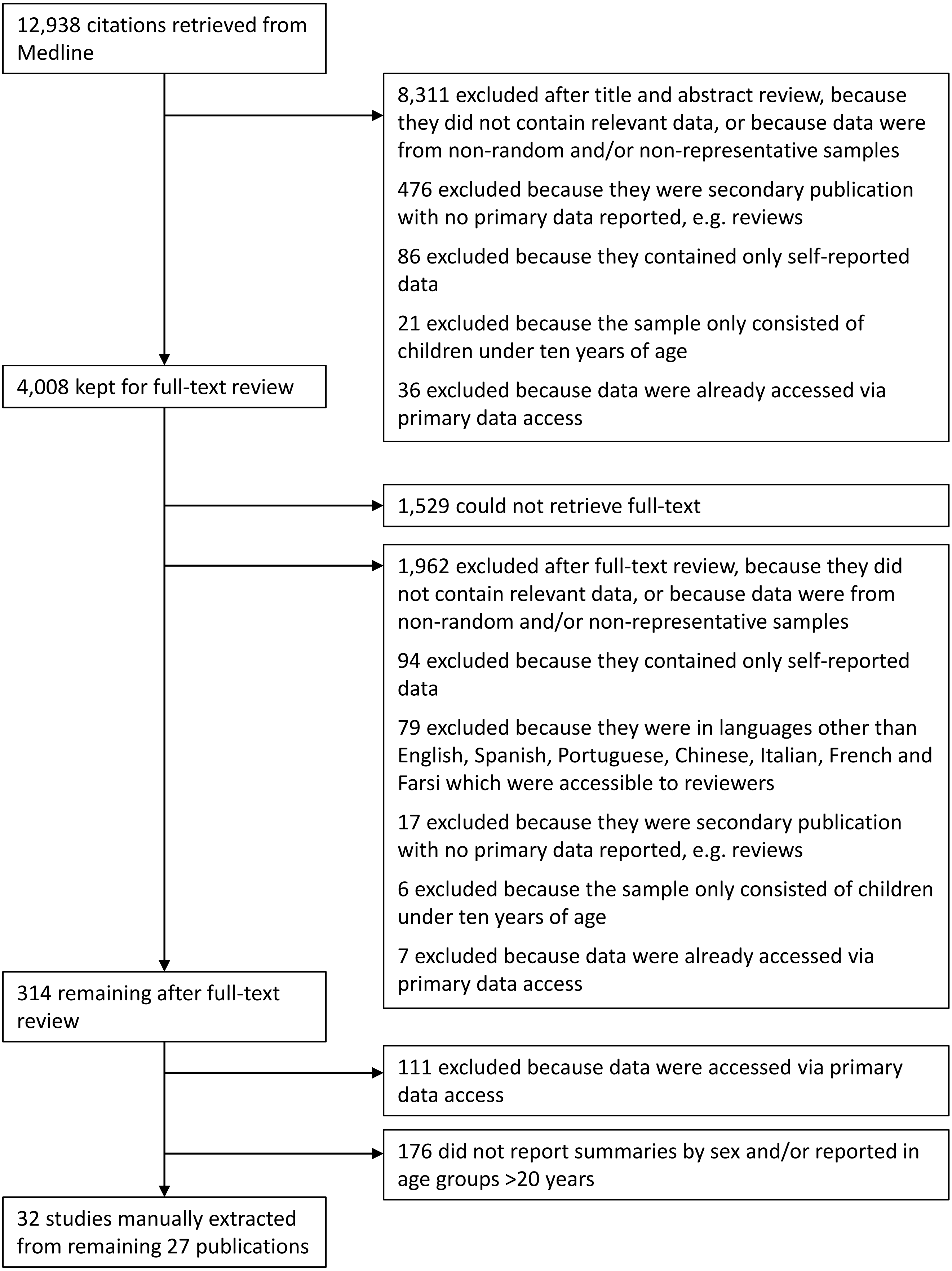
12,938 citations retrieved from Medline
8,311 excluded after title and abstract review, because they did not contain relevant data, or because data were from non-random and/or non-representative samples
476 excluded because they were secondary publication with no primary data reported, e.g. reviews
86 excluded because they contained only self-reported data
21 excluded because the sample only consisted of children under ten years of age
36 excluded because data were already accessed via primary data access
4,008 kept for full-text review
1,529 could not retrieve full-text
1,962 excluded after full-text review, because they did not contain relevant data, or because data were from non-random and/or non-representative samples
94 excluded because they contained only self-reported data
79 excluded because they were in languages other than English, Spanish, Portuguese, Chinese, Italian, French and Farsi which were accessible to reviewers
17 excluded because they were secondary publication with no primary data reported, e.g. reviews
6 excluded because the sample only consisted of children under ten years of age
7 excluded because data were already accessed via primary data access
314 remaining after full-text review
111 excluded because data were accessed via primary data access
176 did not report summaries by sex and/or reported in age groups >20 years
32 studies manually extracted from remaining 27 publications

Appendix Figure 2: Number of data sources by country.

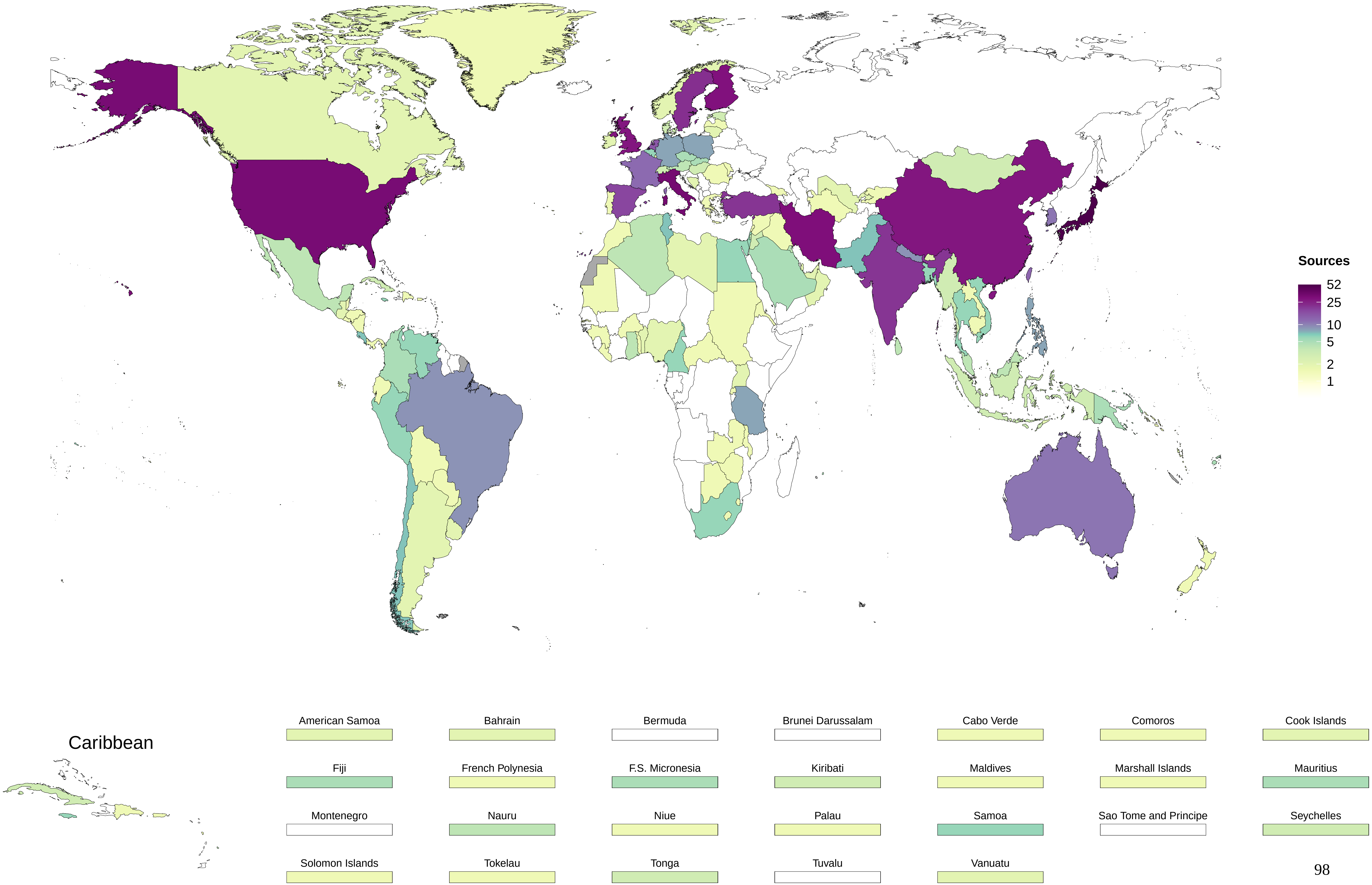
Sources
52
25
10
5
2
1
Caribbean
American Samoa
Bahrain
Bermuda
Brunei Darussalam
Cabo Verde
Comoros
Cook Islands
Fiji
French Polynesia
F.S. Micronesia
Kiribati
Maldives
Marshall Islands
Mauritius
Montenegro
Nauru
Niue
Palau
Samoa
Sao Tome and Principe
Seychelles
Solomon Islands
Tokelau
Tonga
Tuvalu
Vanuatu

Appendix Figure 3: Number of data sources by region and year.

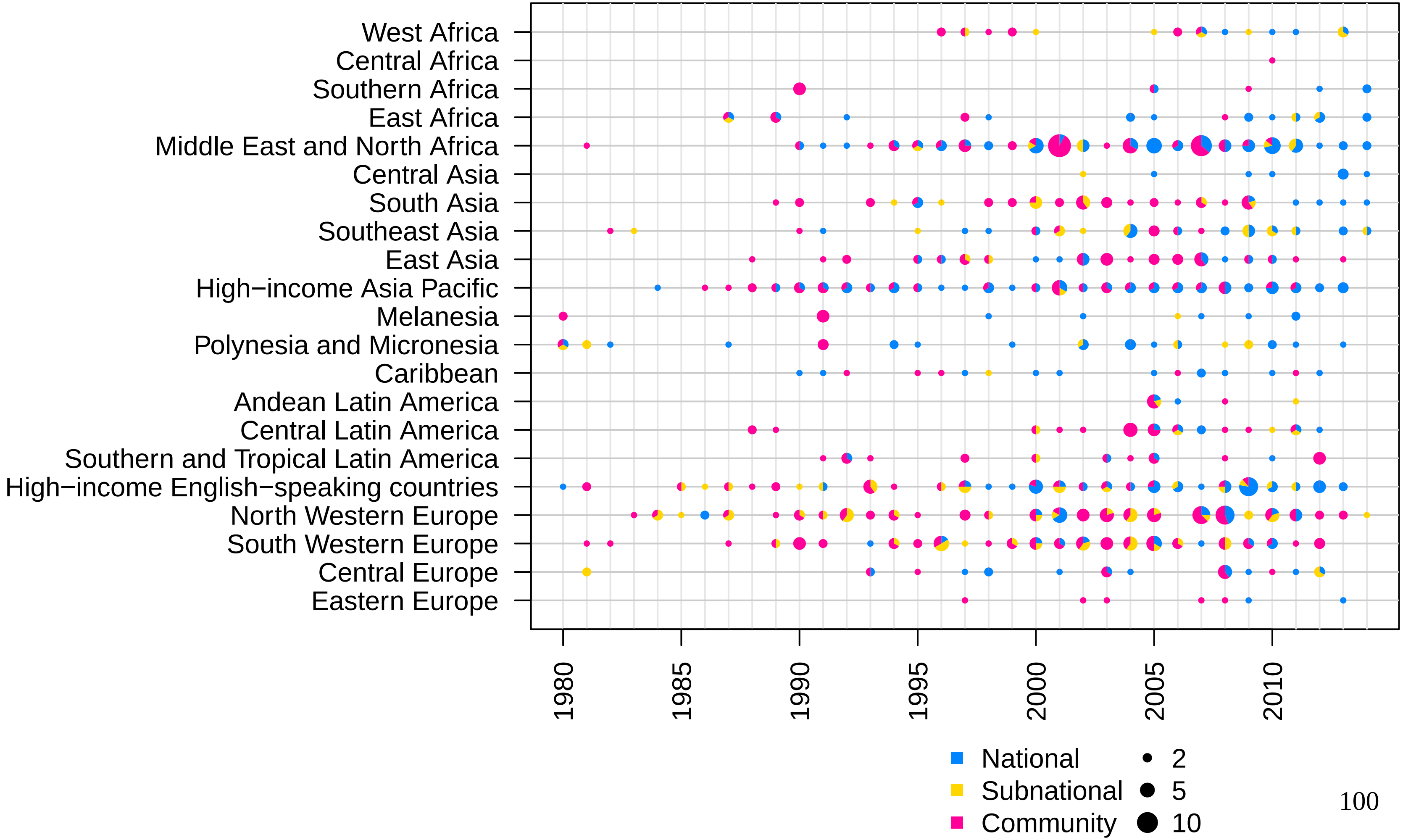
West Africa
Central Africa
Southern Africa
East Africa
Middle East and North Africa
Central Asia
South Asia
Southeast Asia
East Asia
High−income Asia Pacific
Melanesia
Polynesia and Micronesia
Caribbean
Andean Latin America
Central Latin America
Southern and Tropical Latin America
High−income English−speaking countries
North Western Europe
South Western Europe
Central Europe
Eastern Europe
1980
1985
1990
1995
2000
2005
2010
National
Subnational
Community
2
5
10

Appendix Figure 4: Regional trends in the number of adults with diabetes, decomposed into the contributions of population growth and ageing, rise in prevalence, and interaction of the two.

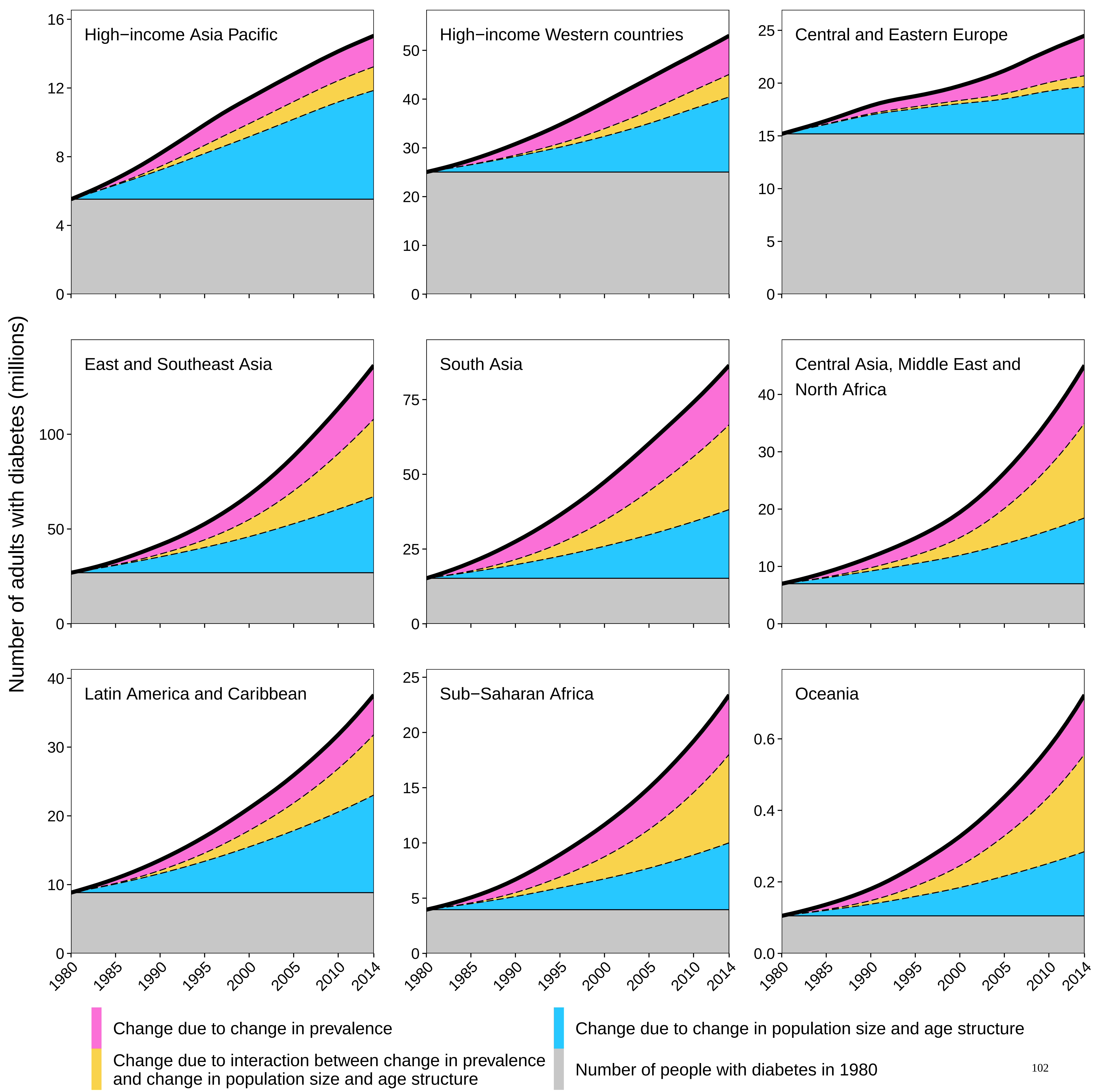
High−income Asia Pacific
High−income Western countries
Central and Eastern Europe
East and Southeast Asia
South Asia
Central Asia, Middle East and North Africa
Latin America and Caribbean
Sub−Saharan Africa
Oceania
Number of adults with diabetes (millions)
Change due to change in prevalence
Change due to interaction between change in prevalence and change in population size and age structure
Change due to change in population size and age structure
Number of people with diabetes in 1980

Appendix Figure 5: Trends in age-standardised and crude prevalence of diabetes by sex and country in people aged 18 years and older. The lines show the posterior mean and the shaded area shows the 95% credible interval for age-standardised prevalence.

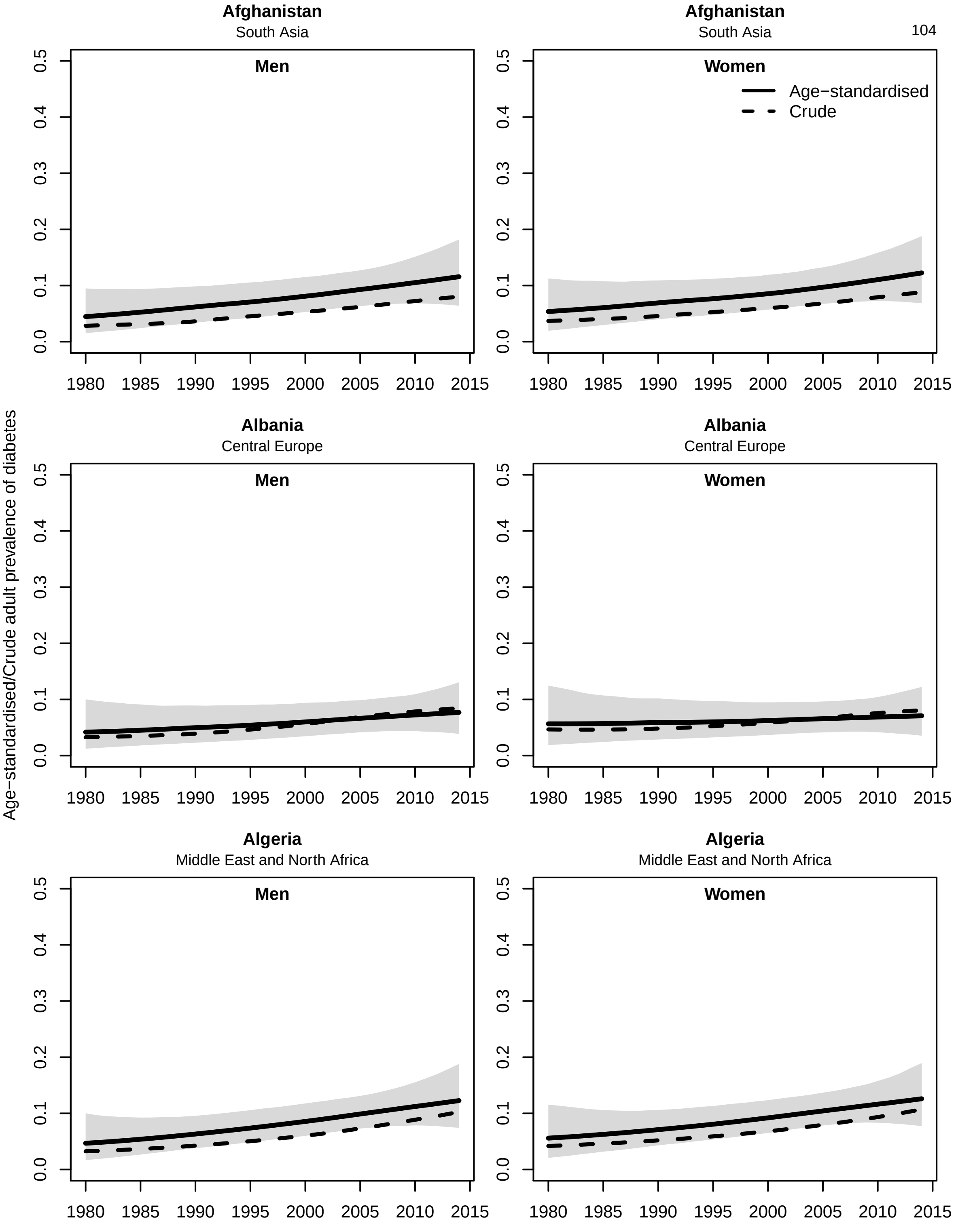
Afghanistan
South Asia
Men
Afghanistan
South Asia
Women
Age−standardised
Crude
Albania
Central Europe
Men
Albania
Central Europe
Women
Algeria
Middle East and North Africa
Men
Algeria
Middle East and North Africa
Women
Age−standardised/Crude adult prevalence of diabetes
0.0
0.1
0.2
0.3
0.4
0.5
1980
1985
1990
1995
2000
2005
2010
2015

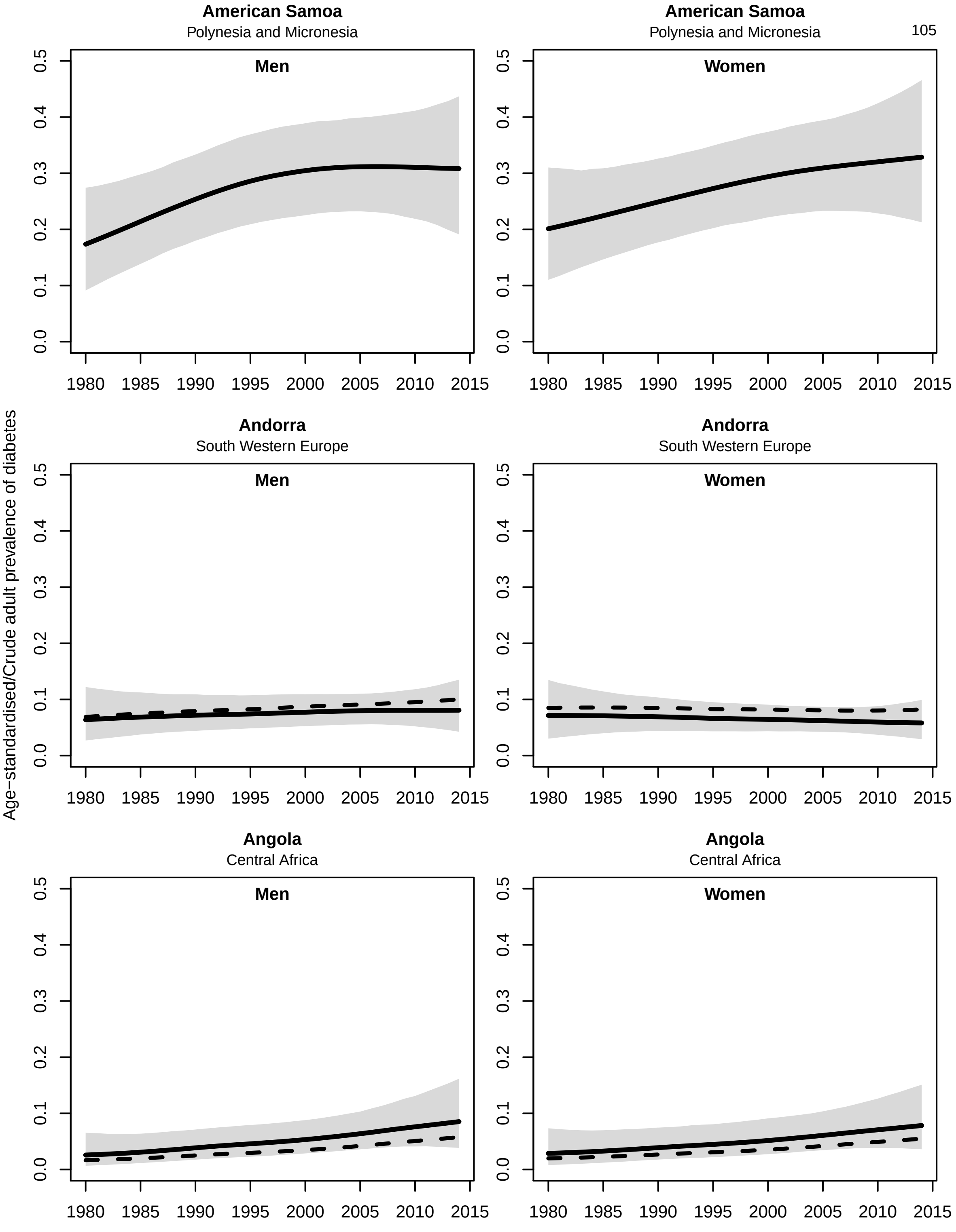
American Samoa
Polynesia and Micronesia
Men
Women
Andorra
South Western Europe
Men
Women
Angola
Central Africa
Men
Women
Age−standardised/Crude adult prevalence of diabetes
0.0
0.1
0.2
0.3
0.4
0.5
1980
1985
1990
1995
2000
2005
2010
2015

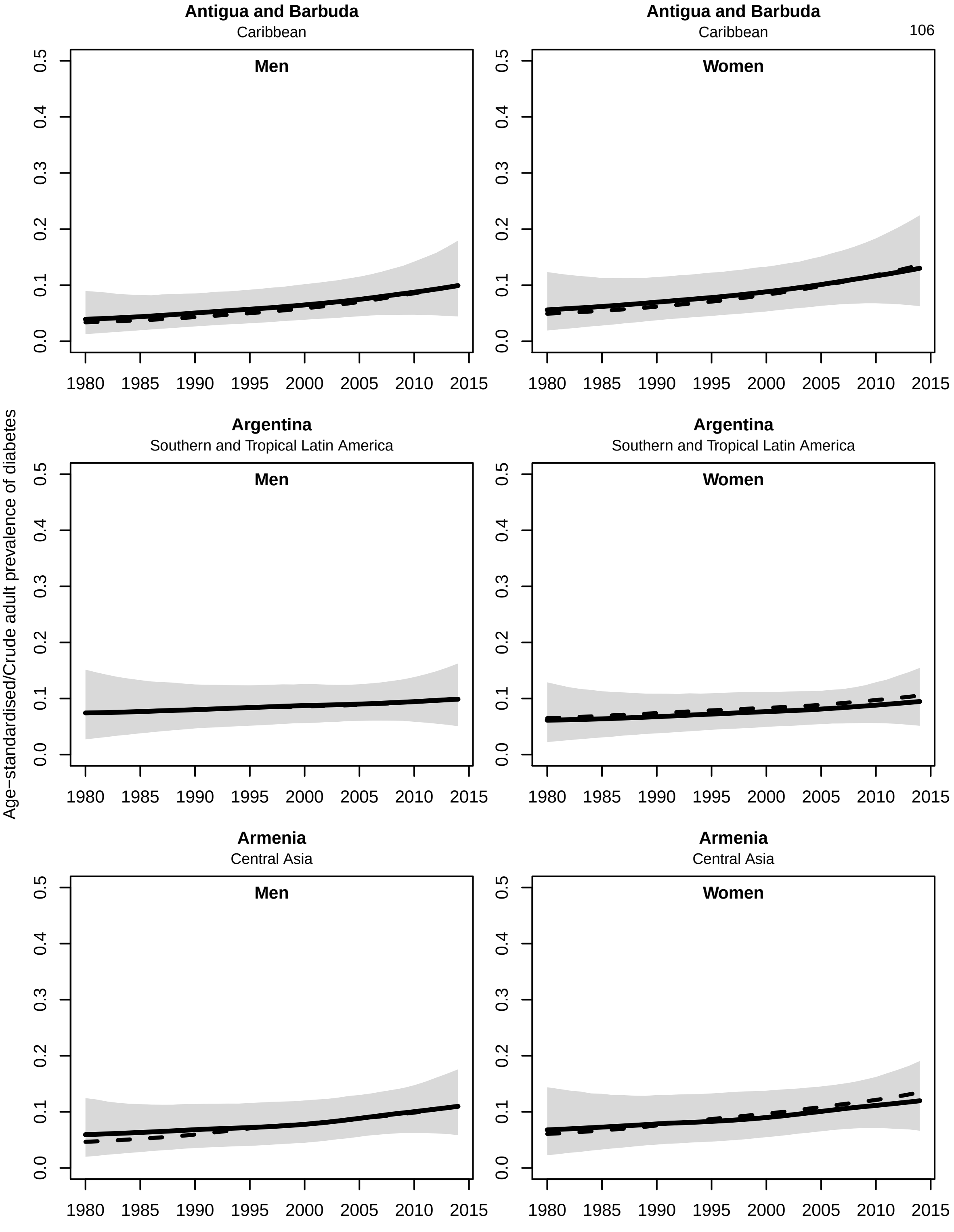
Antigua and Barbuda
Caribbean
Men
Antigua and Barbuda
Caribbean
Women
Argentina
Southern and Tropical Latin America
Men
Argentina
Southern and Tropical Latin America
Women
Armenia
Central Asia
Men
Armenia
Central Asia
Women
Age−standardised/Crude adult prevalence of diabetes
0.0
0.1
0.2
0.3
0.4
0.5
1980
1985
1990
1995
2000
2005
2010
2015

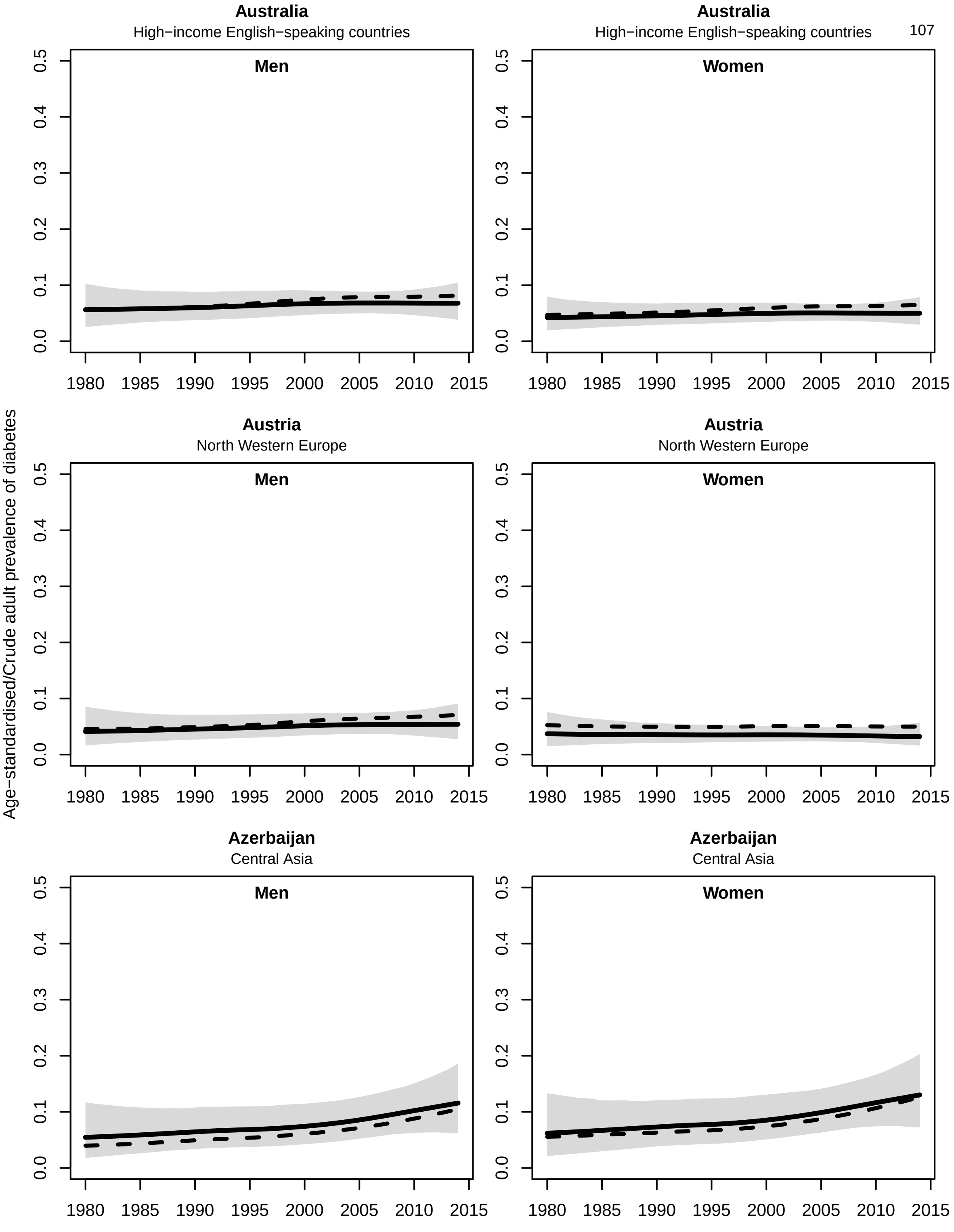
Australia
High−income English−speaking countries
Men
Australia
High−income English−speaking countries
Women
Austria
North Western Europe
Men
Austria
North Western Europe
Women
Azerbaijan
Central Asia
Men
Azerbaijan
Central Asia
Women
Age−standardised/Crude adult prevalence of diabetes
0.0
0.1
0.2
0.3
0.4
0.5
1980
1985
1990
1995
2000
2005
2010
2015

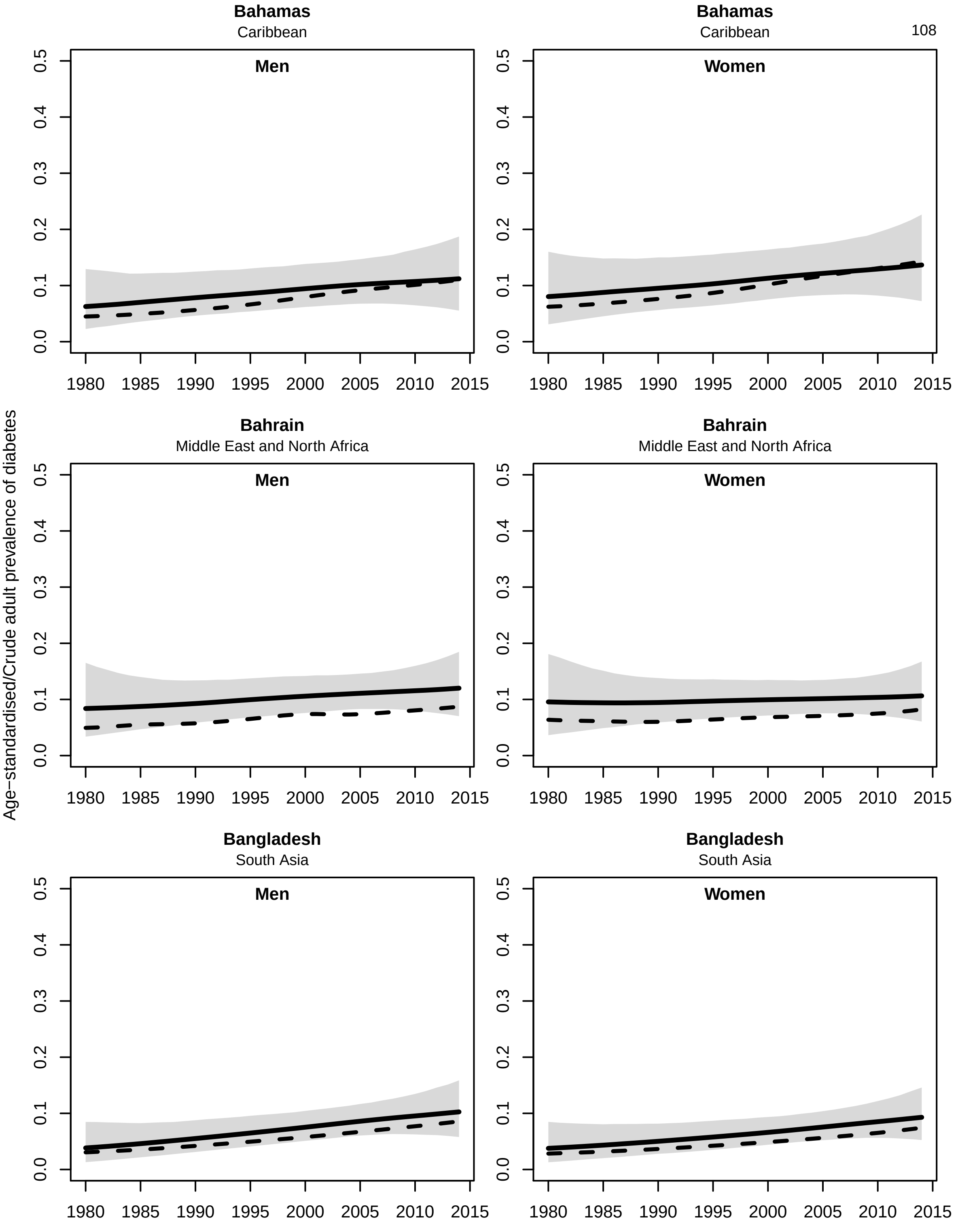
Bahamas
Caribbean
Men
Bahamas
Caribbean
Women
Bahrain
Middle East and North Africa
Men
Bahrain
Middle East and North Africa
Women
Bangladesh
South Asia
Men
Bangladesh
South Asia
Women
Age−standardised/Crude adult prevalence of diabetes
0.0
0.1
0.2
0.3
0.4
0.5
1980
1985
1990
1995
2000
2005
2010
2015

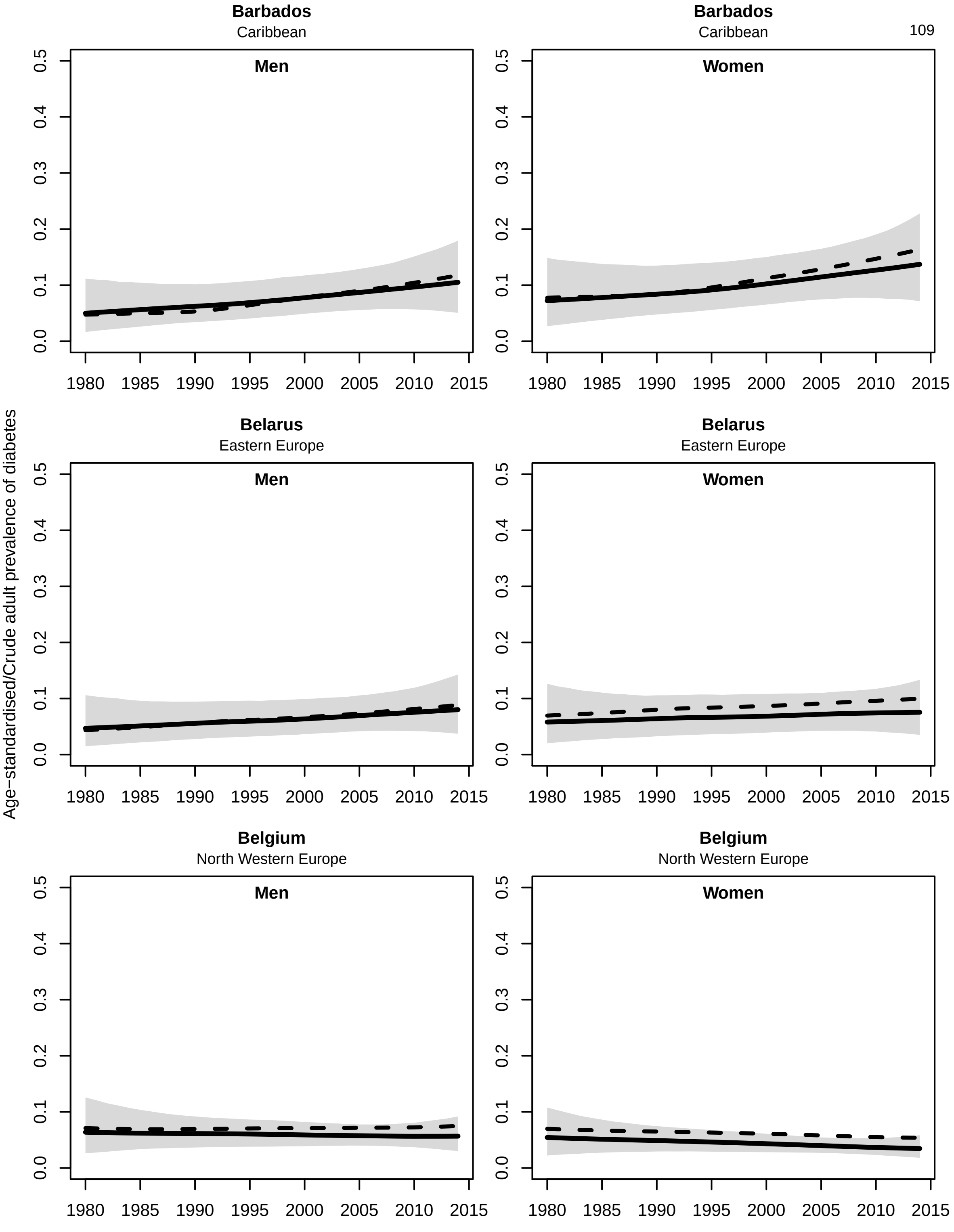
Barbados
Caribbean
Men
Barbados
Caribbean
Women
Belarus
Eastern Europe
Men
Belarus
Eastern Europe
Women
Belgium
North Western Europe
Men
Belgium
North Western Europe
Women
Age−standardised/Crude adult prevalence of diabetes
0.0
0.1
0.2
0.3
0.4
0.5
1980
1985
1990
1995
2000
2005
2010
2015

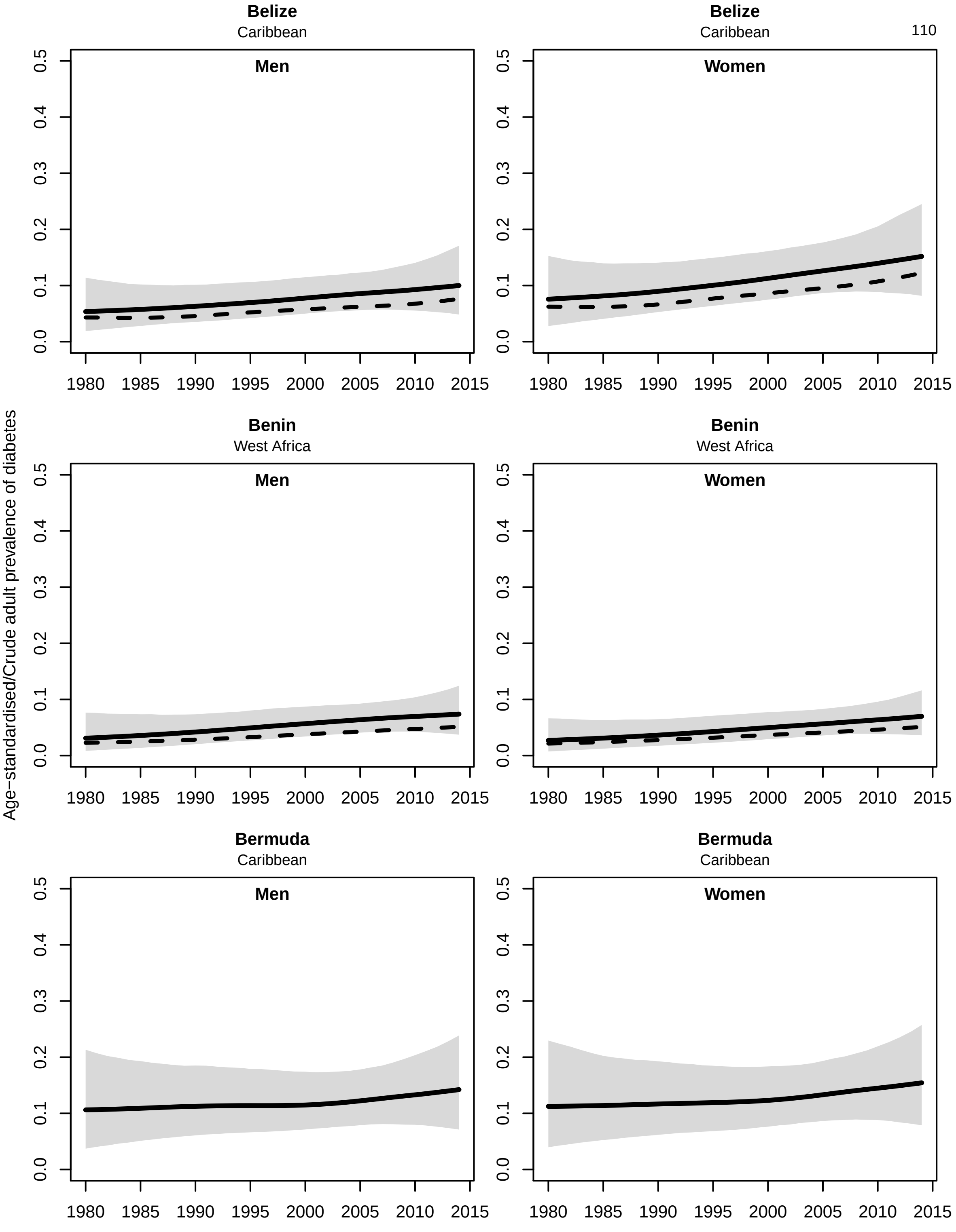
Belize
Caribbean
Men
Women
Benin
West Africa
Men
Women
Bermuda
Caribbean
Men
Women
Age−standardised/Crude adult prevalence of diabetes
0.0
0.1
0.2
0.3
0.4
0.5
1980
1985
1990
1995
2000
2005
2010
2015

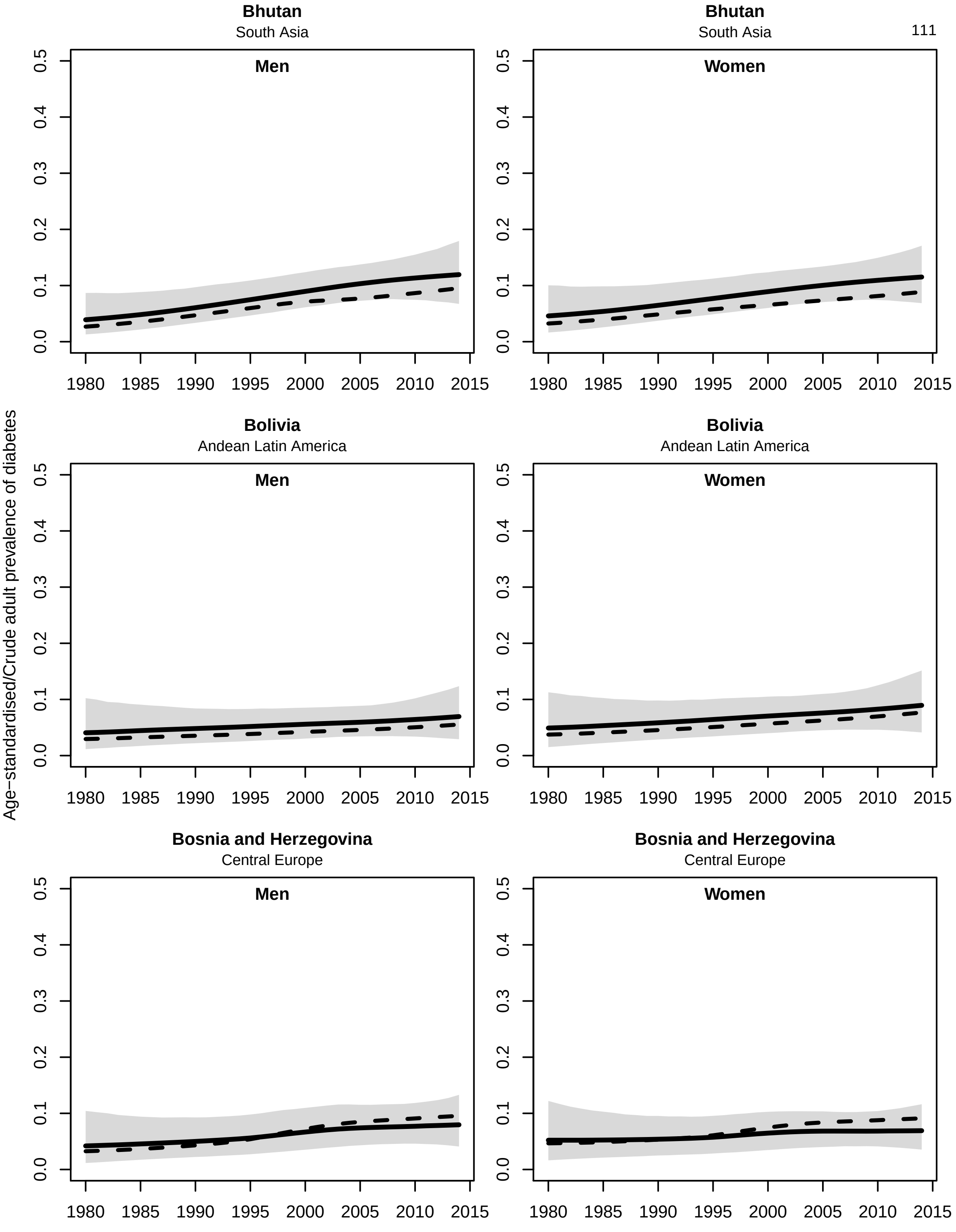
Bhutan
South Asia
Men
Bhutan
South Asia
Women
Bolivia
Andean Latin America
Men
Bolivia
Andean Latin America
Women
Bosnia and Herzegovina
Central Europe
Men
Bosnia and Herzegovina
Central Europe
Women
Age−standardised/Crude adult prevalence of diabetes
0.0
0.1
0.2
0.3
0.4
0.5
1980
1985
1990
1995
2000
2005
2010
2015

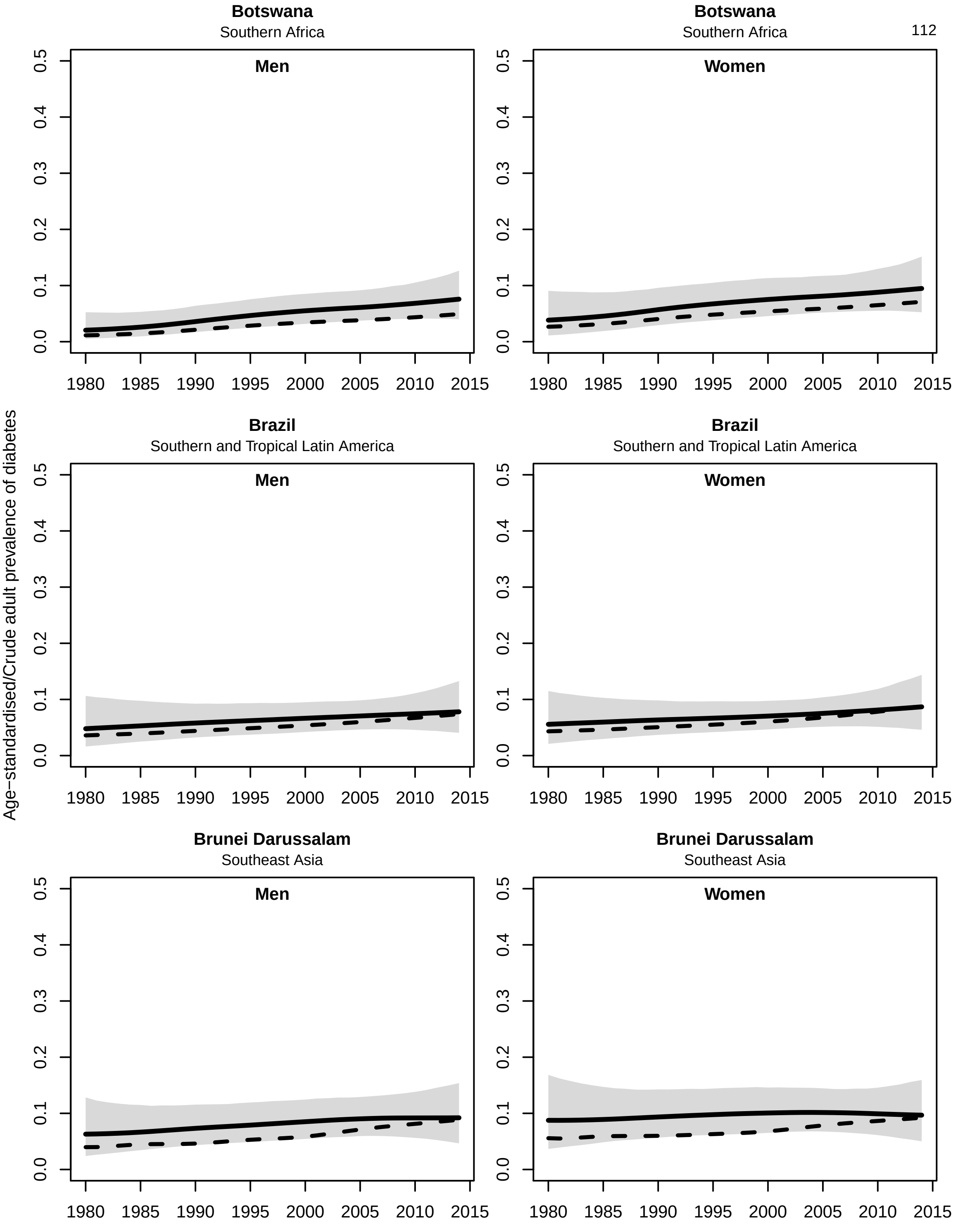
Botswana
Southern Africa
Men
Botswana
Southern Africa
Women
Brazil
Southern and Tropical Latin America
Men
Brazil
Southern and Tropical Latin America
Women
Brunei Darussalam
Southeast Asia
Men
Brunei Darussalam
Southeast Asia
Women
Age−standardised/Crude adult prevalence of diabetes
0.0
0.1
0.2
0.3
0.4
0.5
1980
1985
1990
1995
2000
2005
2010
2015

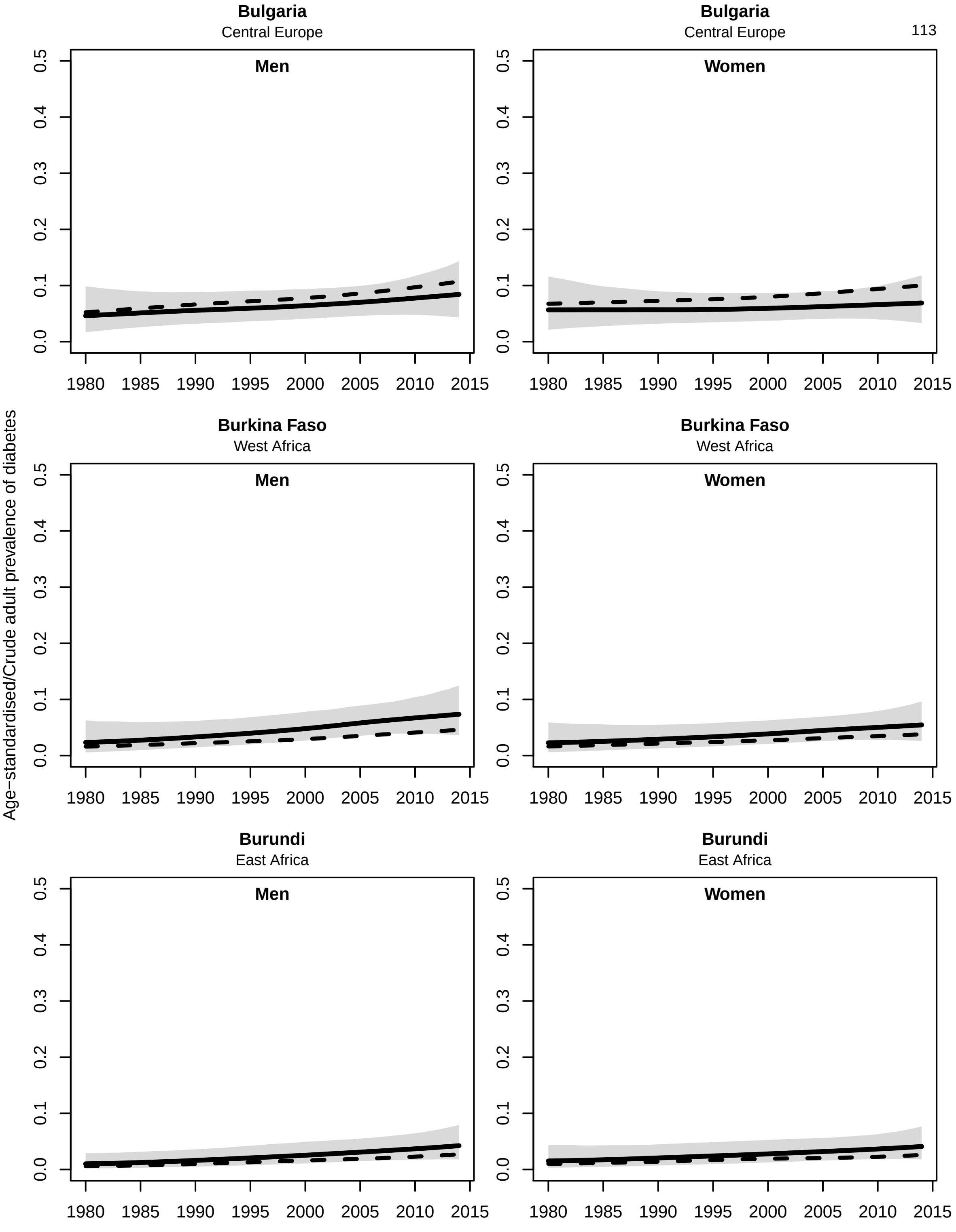
Bulgaria
Central Europe
Men
Bulgaria
Central Europe
Women
Burkina Faso
West Africa
Men
Burkina Faso
West Africa
Women
Burundi
East Africa
Men
Burundi
East Africa
Women
Age−standardised/Crude adult prevalence of diabetes
0.0
0.1
0.2
0.3
0.4
0.5
1980
1985
1990
1995
2000
2005
2010
2015

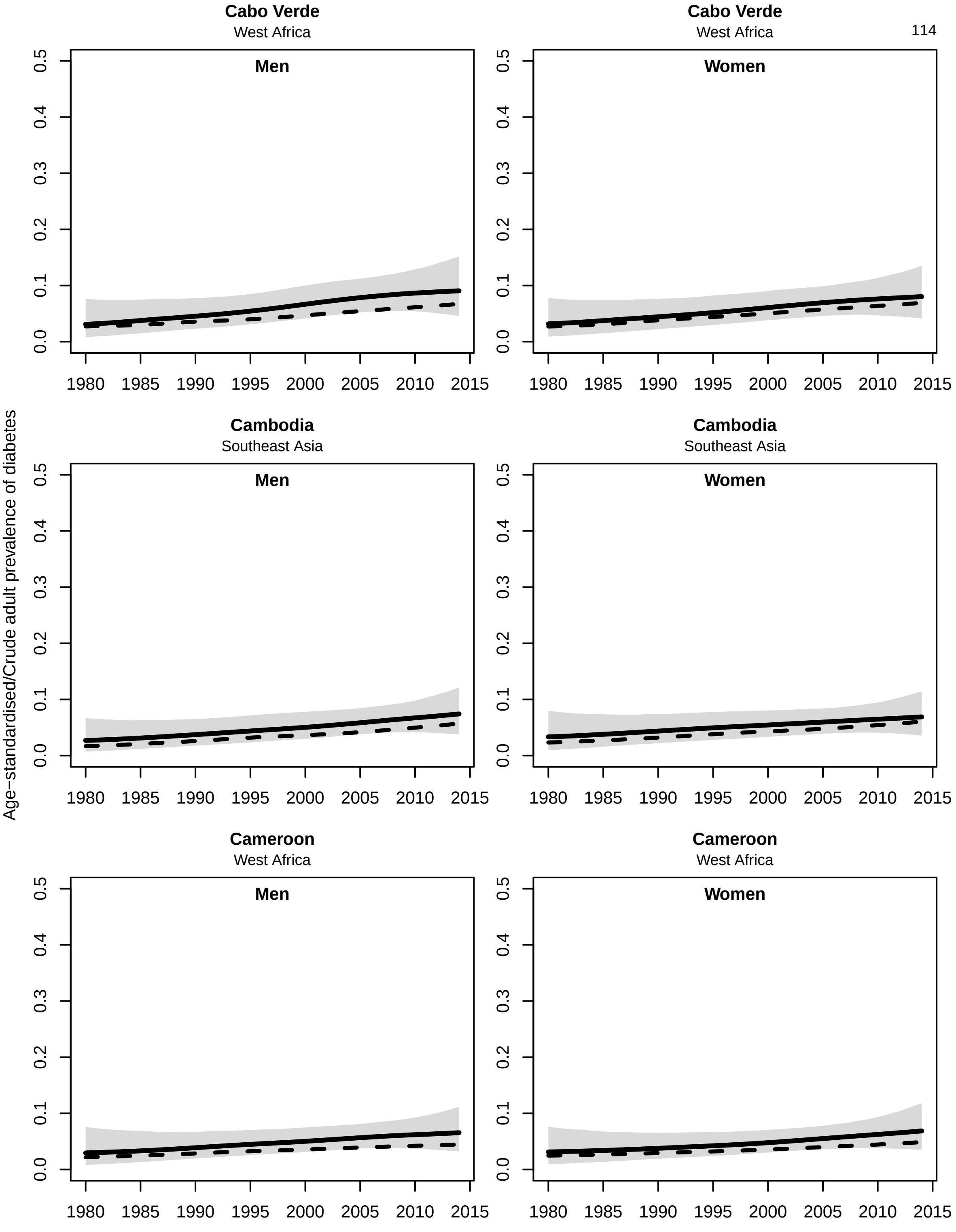
Cabo Verde
West Africa
Men
Cabo Verde
West Africa
Women
Cambodia
Southeast Asia
Men
Cambodia
Southeast Asia
Women
Cameroon
West Africa
Men
Cameroon
West Africa
Women
Age−standardised/Crude adult prevalence of diabetes
0.0
0.1
0.2
0.3
0.4
0.5
1980
1985
1990
1995
2000
2005
2010
2015

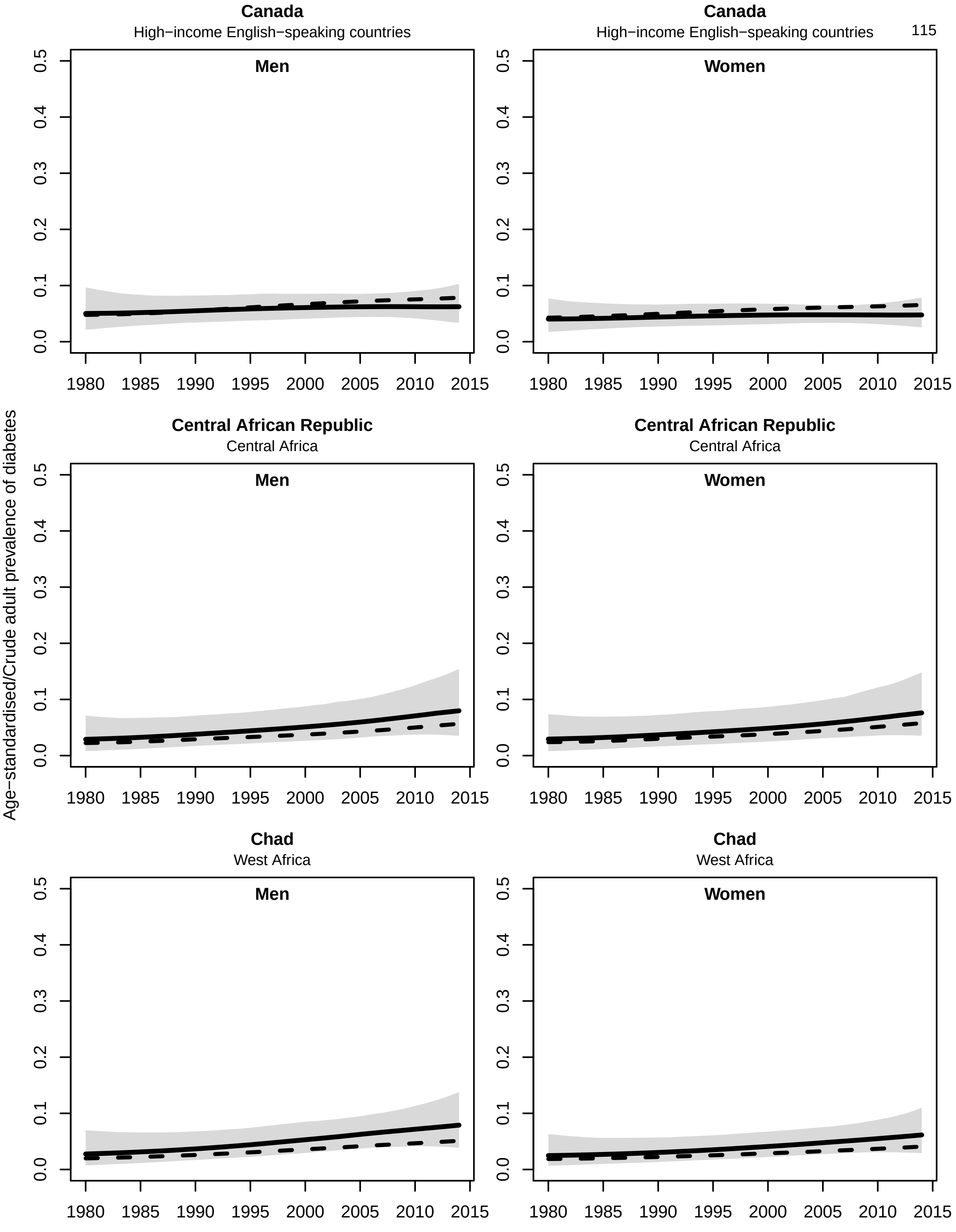
Canada
High−income English−speaking countries
Men
Canada
High−income English−speaking countries
Women
Central African Republic
Central Africa
Men
Central African Republic
Central Africa
Women
Chad
West Africa
Men
Chad
West Africa
Women
Age−standardised/Crude adult prevalence of diabetes
0.0
0.1
0.2
0.3
0.4
0.5
1980
1985
1990
1995
2000
2005
2010
2015

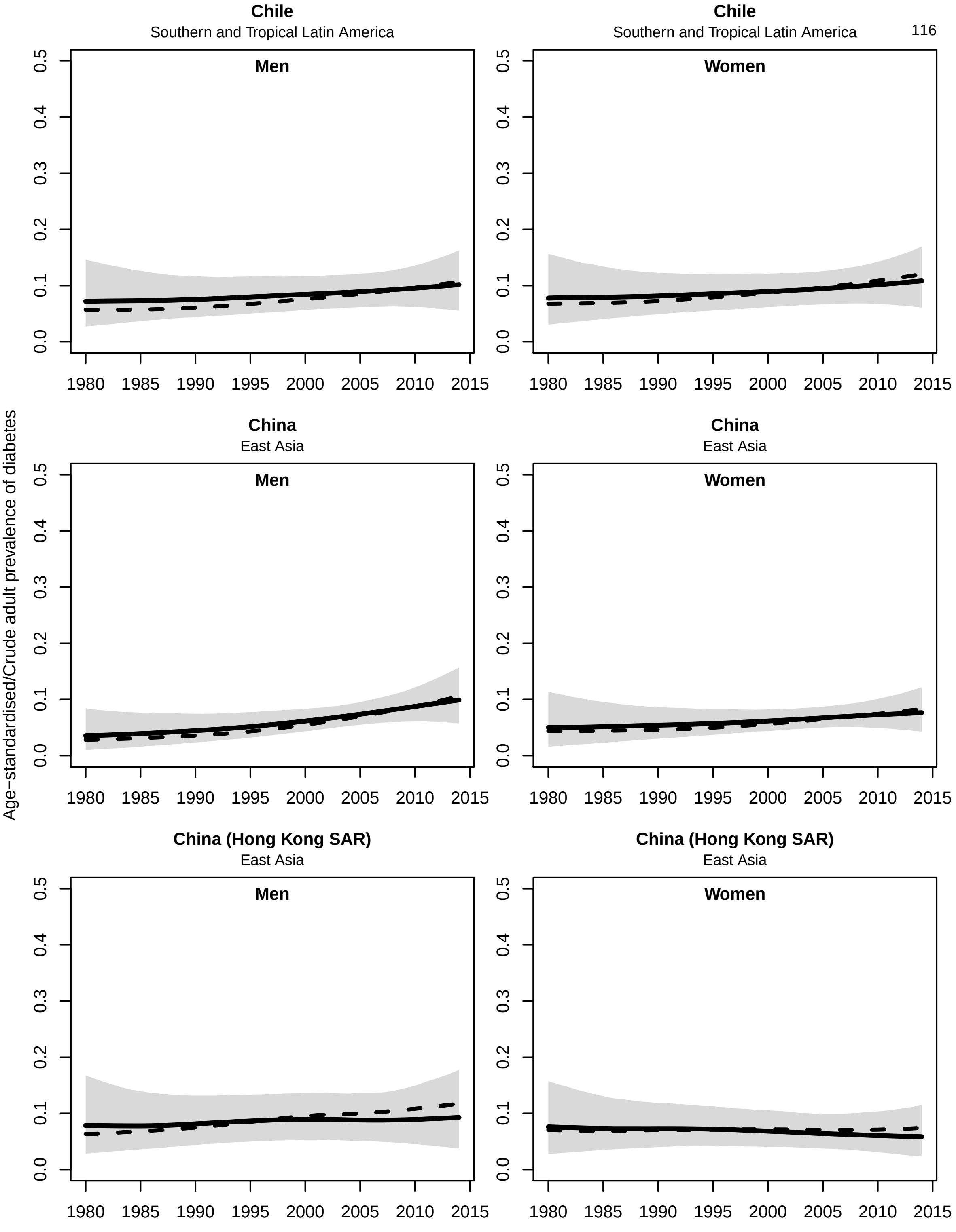
Chile
Southern and Tropical Latin America
Men
Women
China
East Asia
China (Hong Kong SAR)
Age−standardised/Crude adult prevalence of diabetes
0.0
0.1
0.2
0.3
0.4
0.5
1980
1985
1990
1995
2000
2005
2010
2015

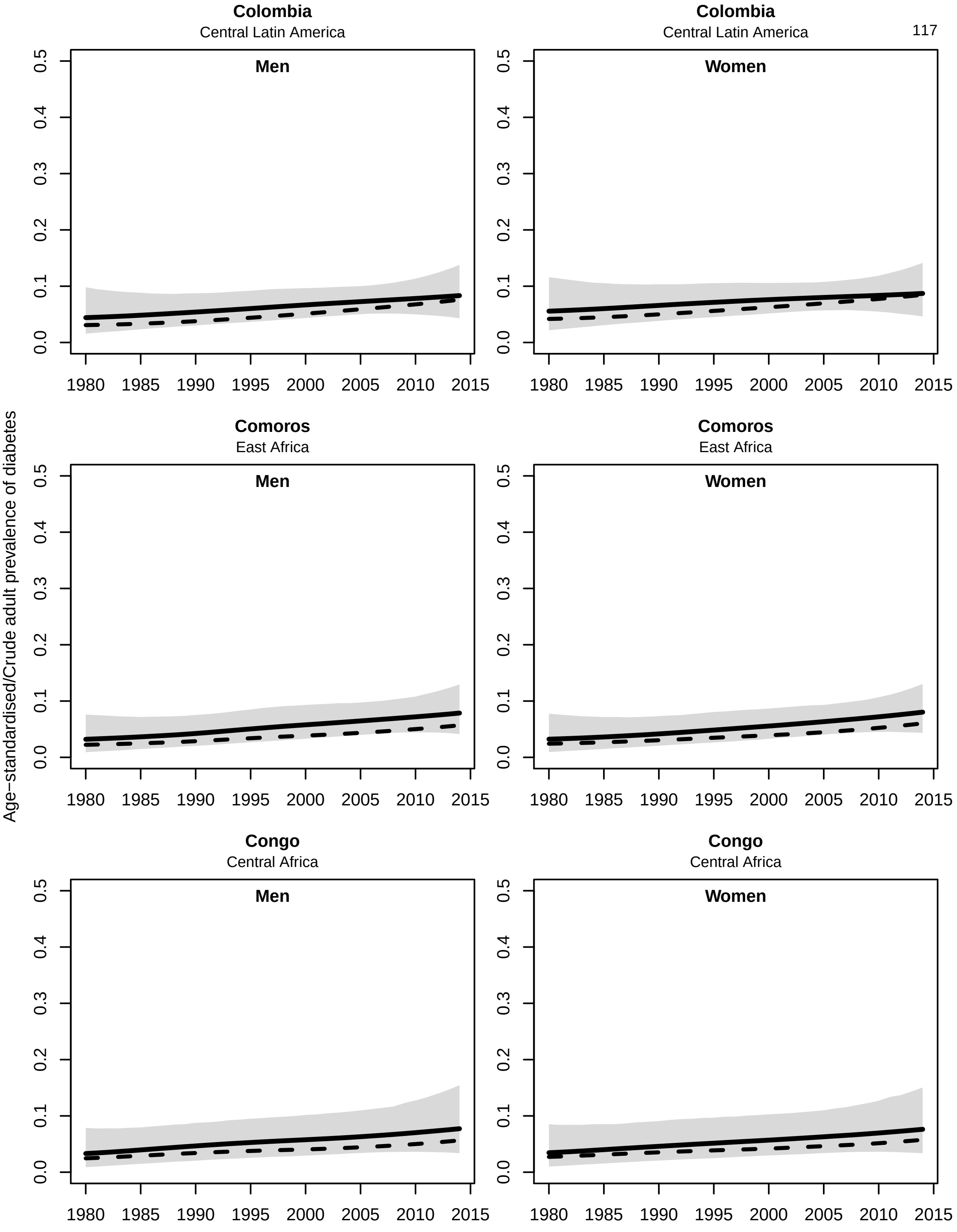
Colombia
Central Latin America
Men
Colombia
Central Latin America
Women
Comoros
East Africa
Men
Comoros
East Africa
Women
Congo
Central Africa
Men
Congo
Central Africa
Women
Age−standardised/Crude adult prevalence of diabetes
0.0
0.1
0.2
0.3
0.4
0.5
1980
1985
1990
1995
2000
2005
2010
2015

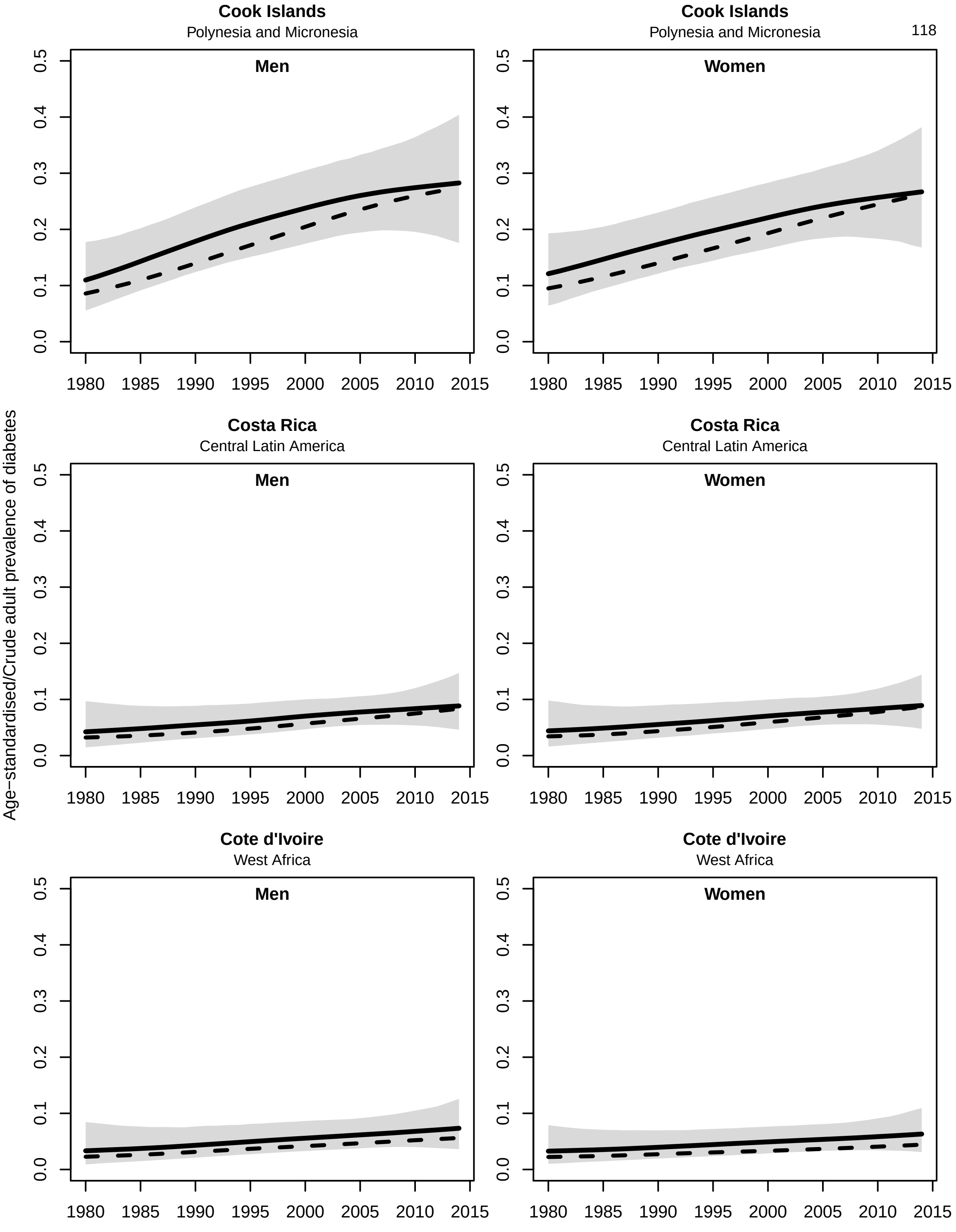
Cook Islands
Polynesia and Micronesia
Men
Women
Costa Rica
Central Latin America
Cote d'Ivoire
West Africa
Age−standardised/Crude adult prevalence of diabetes
0.0
0.1
0.2
0.3
0.4
0.5
1980
1985
1990
1995
2000
2005
2010
2015

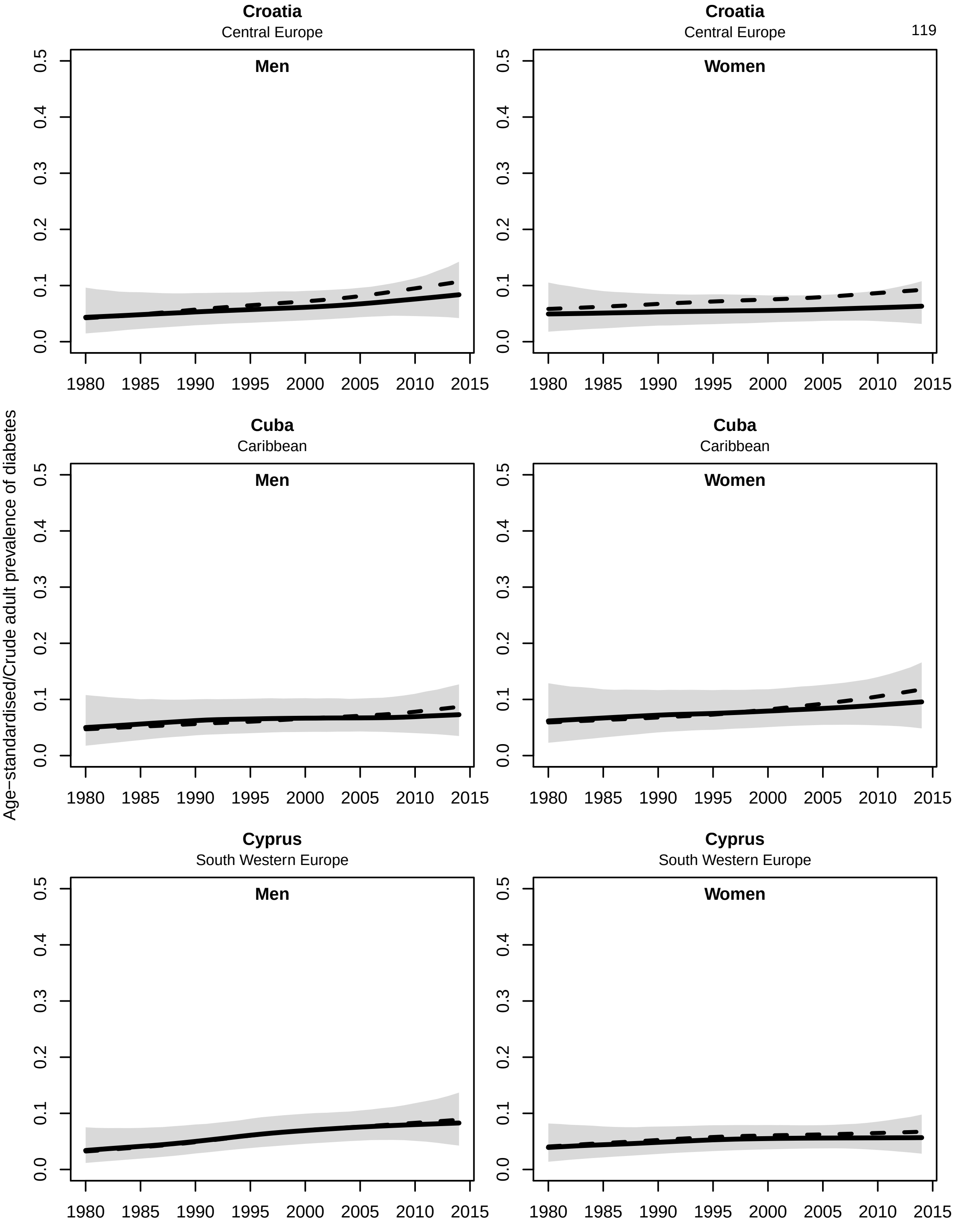
Croatia
Central Europe
Men
Croatia
Central Europe
Women
Cuba
Caribbean
Men
Cuba
Caribbean
Women
Cyprus
South Western Europe
Men
Cyprus
South Western Europe
Women
Age−standardised/Crude adult prevalence of diabetes
0.0
0.1
0.2
0.3
0.4
0.5
1980
1985
1990
1995
2000
2005
2010
2015

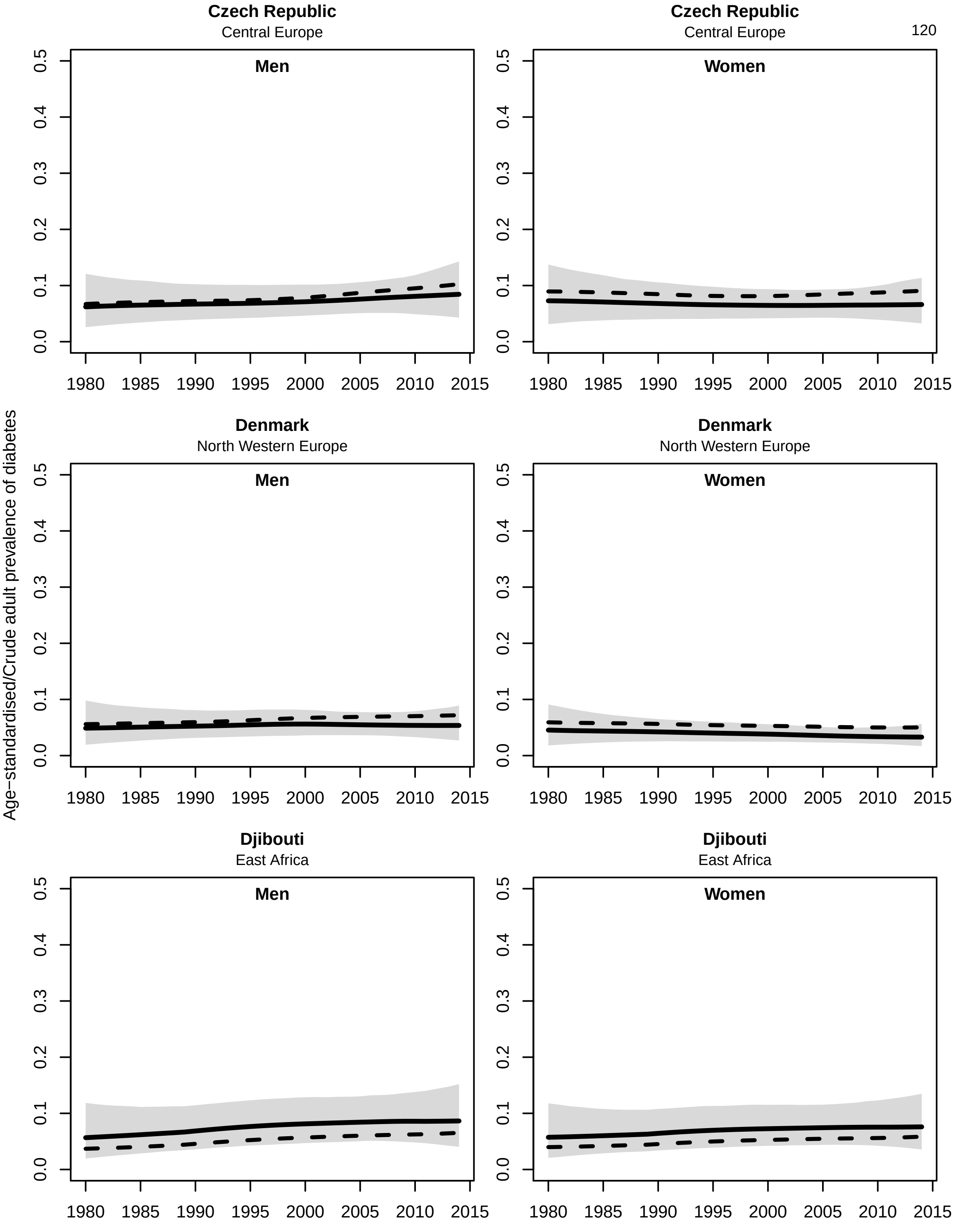
Czech Republic
Central Europe
Men
Czech Republic
Central Europe
Women
Denmark
North Western Europe
Men
Denmark
North Western Europe
Women
Djibouti
East Africa
Men
Djibouti
East Africa
Women
Age−standardised/Crude adult prevalence of diabetes
0.0
0.1
0.2
0.3
0.4
0.5
1980
1985
1990
1995
2000
2005
2010
2015

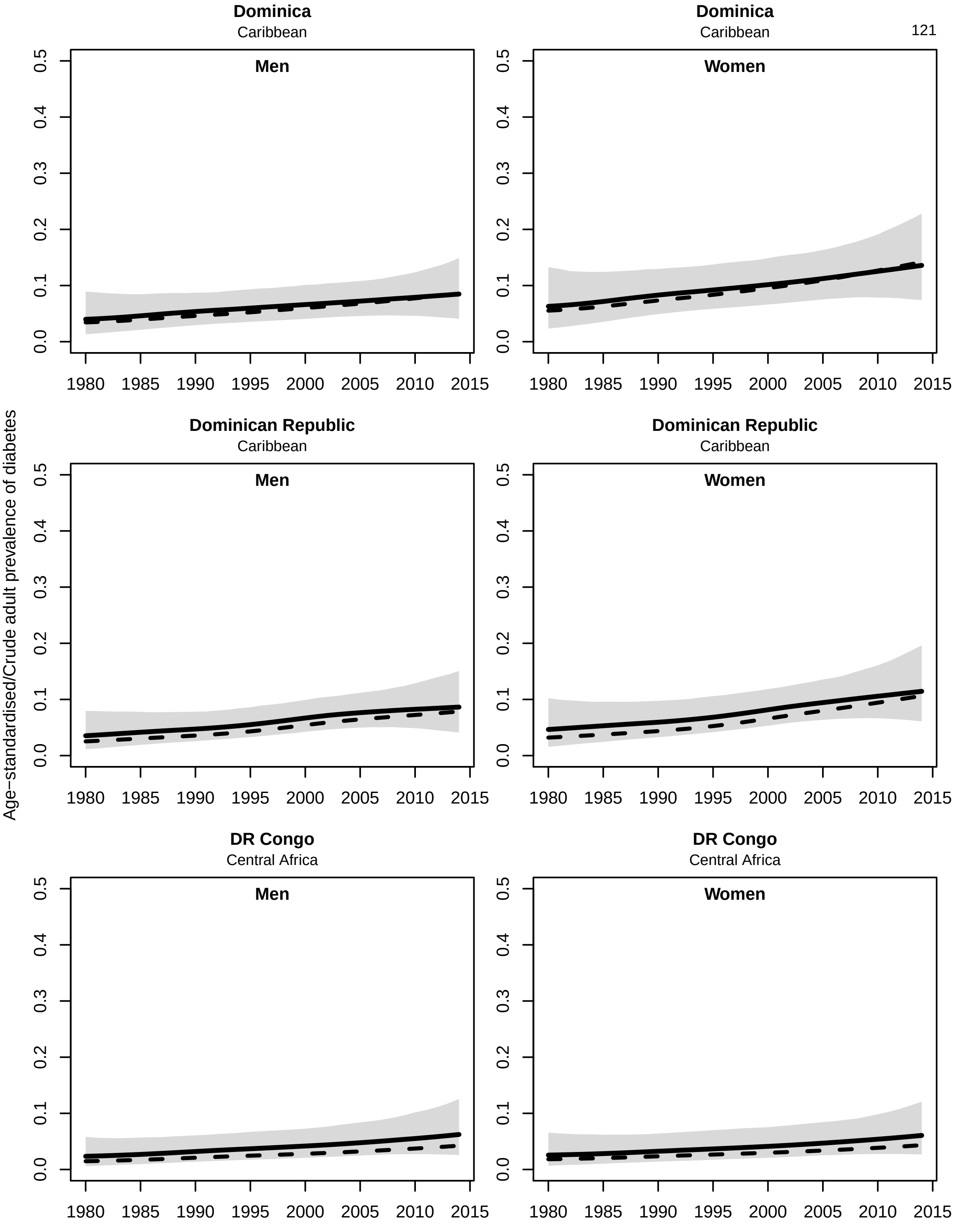
Dominica
Caribbean
Men
Dominica
Caribbean
Women
Dominican Republic
Caribbean
Men
Dominican Republic
Caribbean
Women
DR Congo
Central Africa
Men
DR Congo
Central Africa
Women
Age−standardised/Crude adult prevalence of diabetes
0.0
0.1
0.2
0.3
0.4
0.5
1980
1985
1990
1995
2000
2005
2010
2015

Age−standardised/Crude adult prevalence of diabetes

Ecuador
Andean Latin America

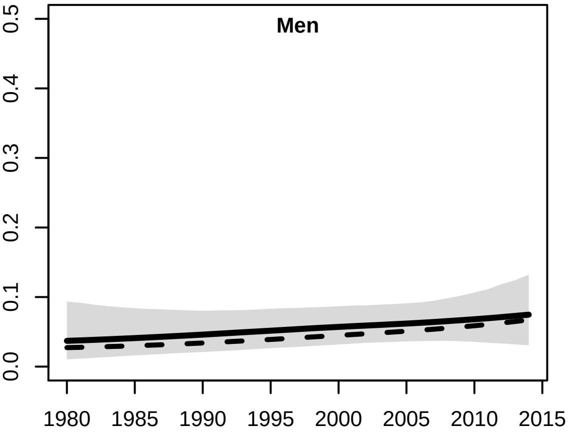

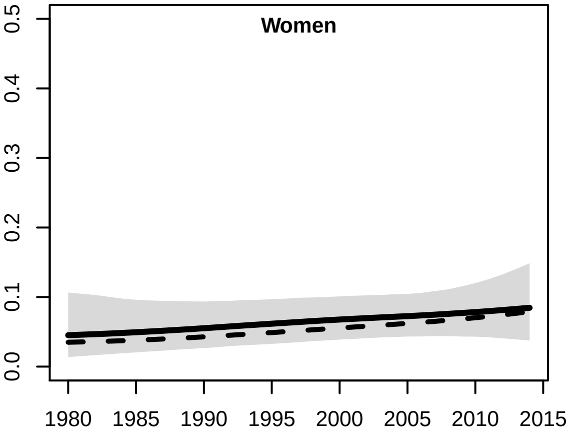

Egypt
Middle East and North Africa

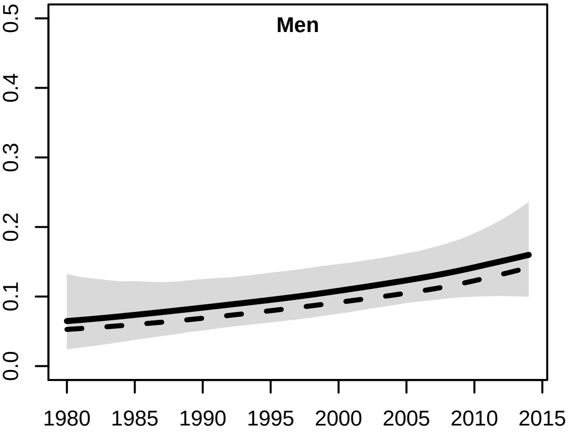

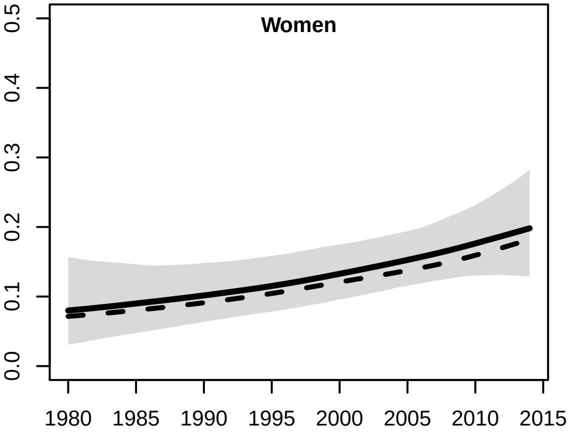

El Salvador
Central Latin America

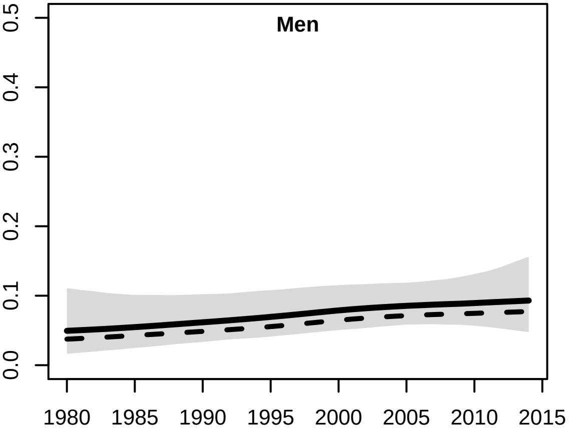

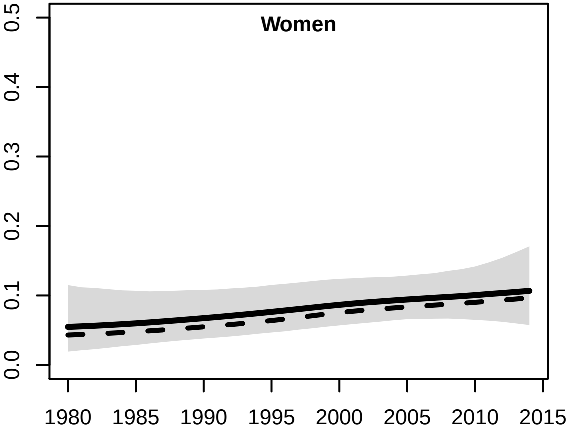

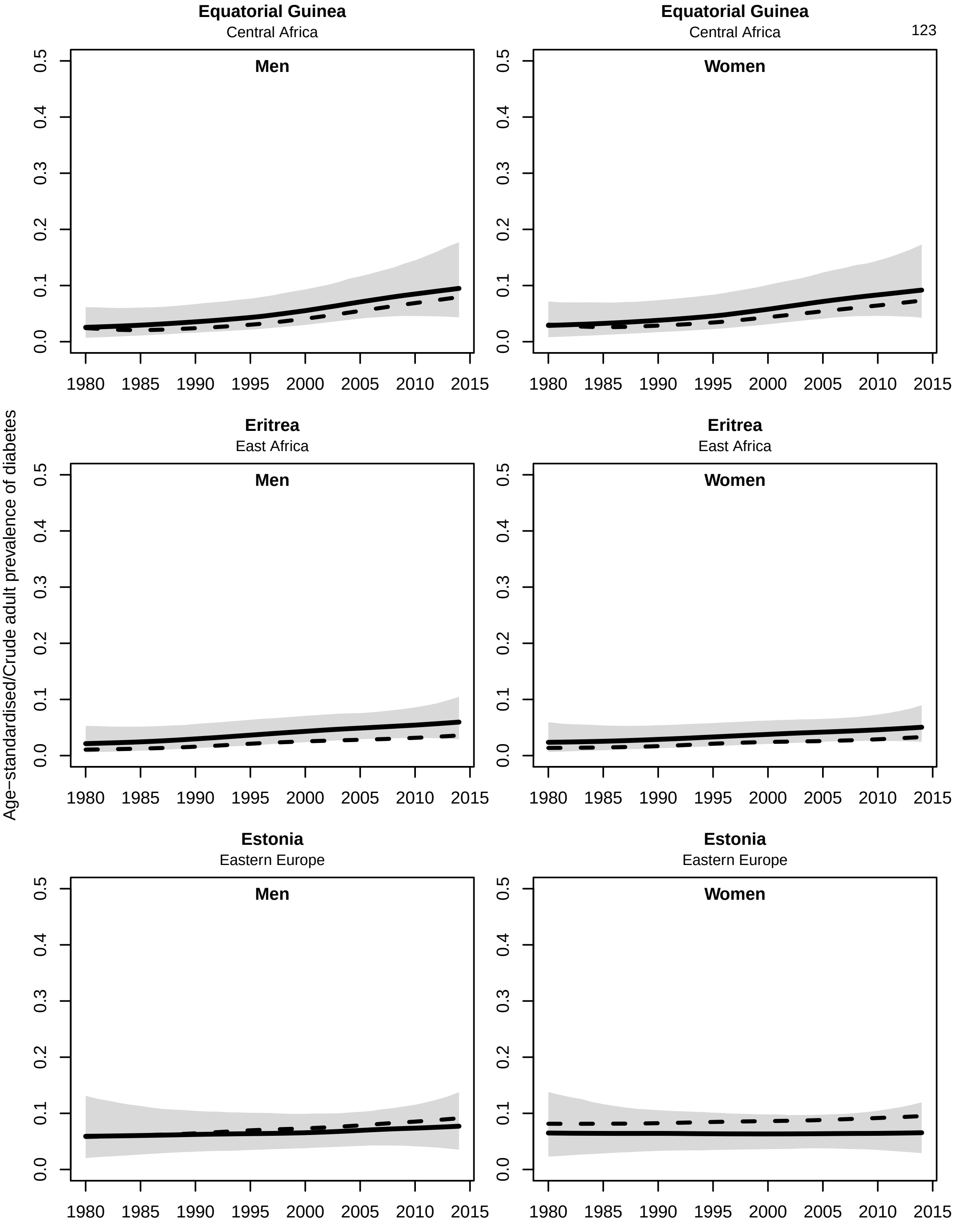
Equatorial Guinea
Central Africa
Men
Equatorial Guinea
Central Africa
Women
Eritrea
East Africa
Men
Eritrea
East Africa
Women
Estonia
Eastern Europe
Men
Estonia
Eastern Europe
Women
Age−standardised/Crude adult prevalence of diabetes
0.0
0.1
0.2
0.3
0.4
0.5
1980
1985
1990
1995
2000
2005
2010
2015

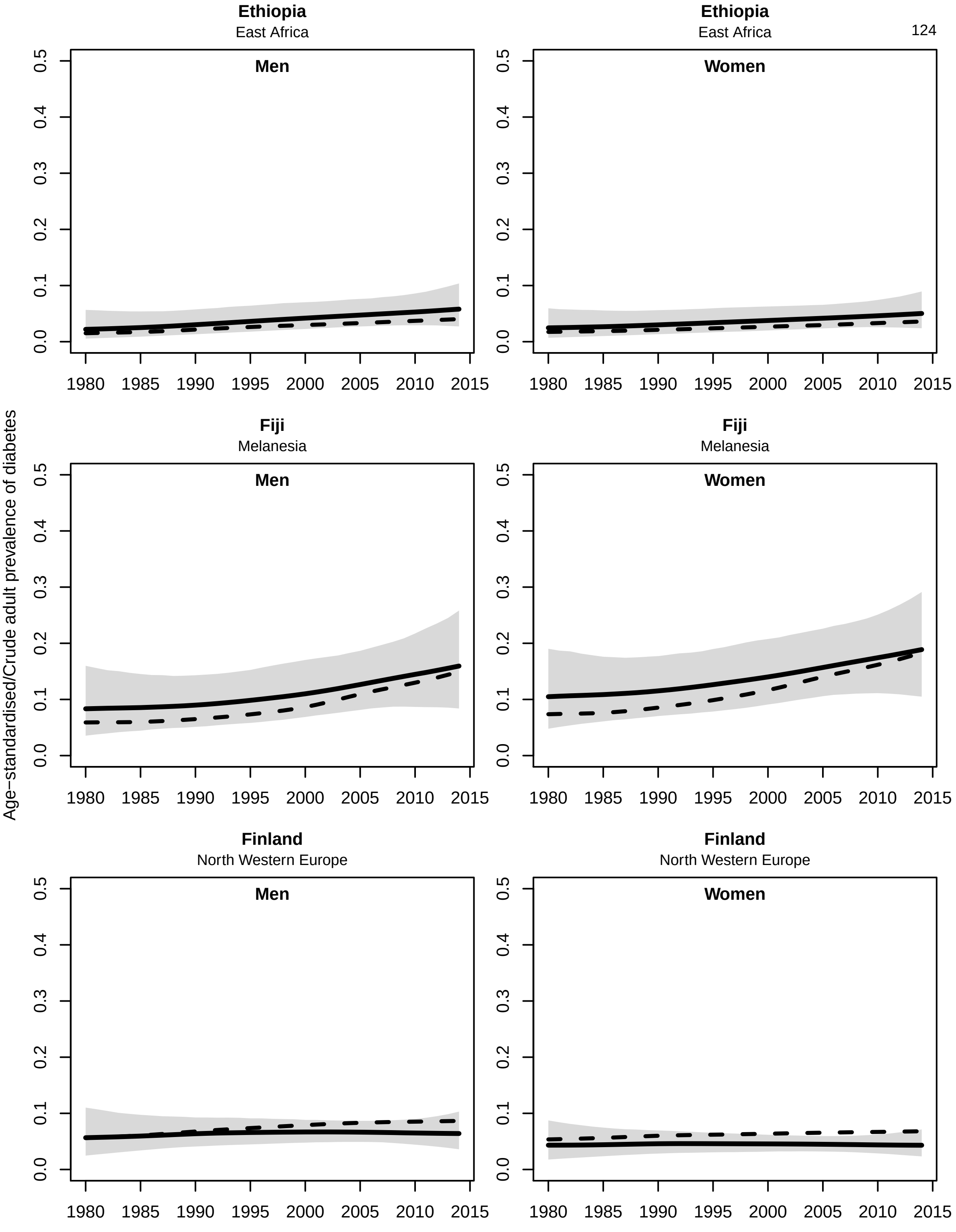
Ethiopia
East Africa
Men
Ethiopia
East Africa
Women
Fiji
Melanesia
Men
Fiji
Melanesia
Women
Finland
North Western Europe
Men
Finland
North Western Europe
Women
Age−standardised/Crude adult prevalence of diabetes
0.0
0.1
0.2
0.3
0.4
0.5
1980
1985
1990
1995
2000
2005
2010
2015

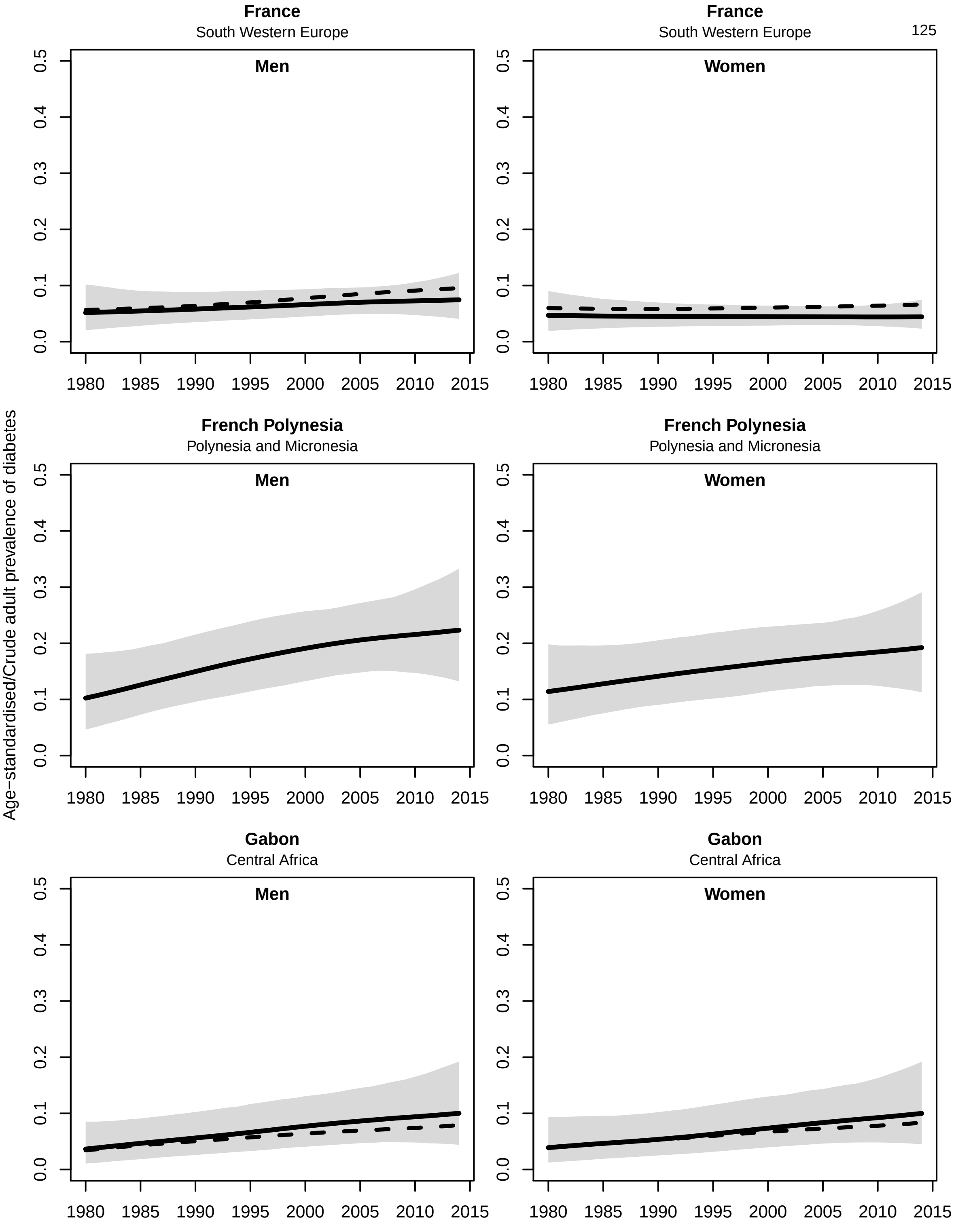
France
South Western Europe
Men
France
South Western Europe
Women
French Polynesia
Polynesia and Micronesia
Men
French Polynesia
Polynesia and Micronesia
Women
Gabon
Central Africa
Men
Gabon
Central Africa
Women
Age−standardised/Crude adult prevalence of diabetes
0.0
0.1
0.2
0.3
0.4
0.5
1980
1985
1990
1995
2000
2005
2010
2015

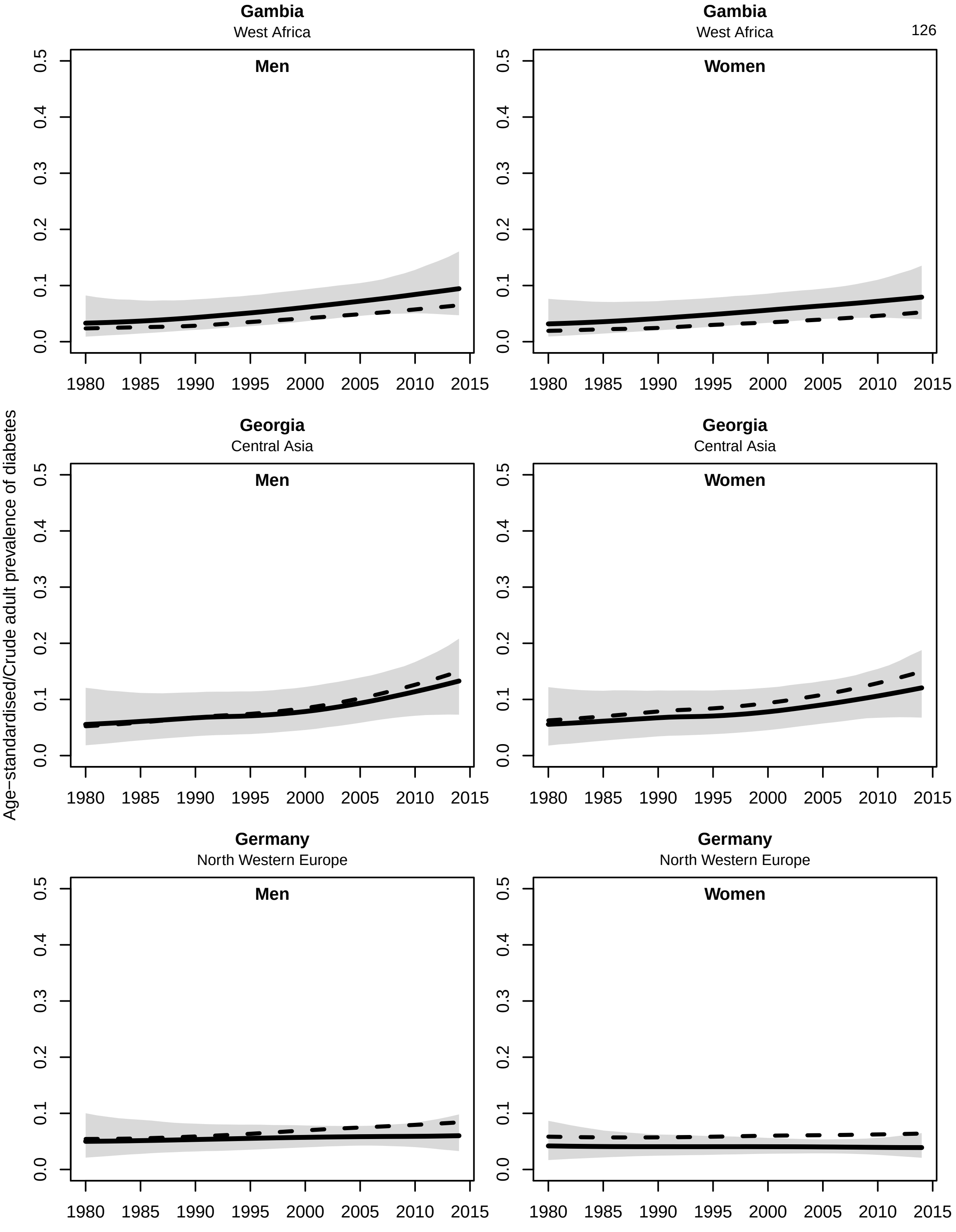
Gambia
West Africa
Men
Gambia
West Africa
Women
Georgia
Central Asia
Men
Georgia
Central Asia
Women
Germany
North Western Europe
Men
Germany
North Western Europe
Women
Age−standardised/Crude adult prevalence of diabetes
0.0
0.1
0.2
0.3
0.4
0.5
1980
1985
1990
1995
2000
2005
2010
2015

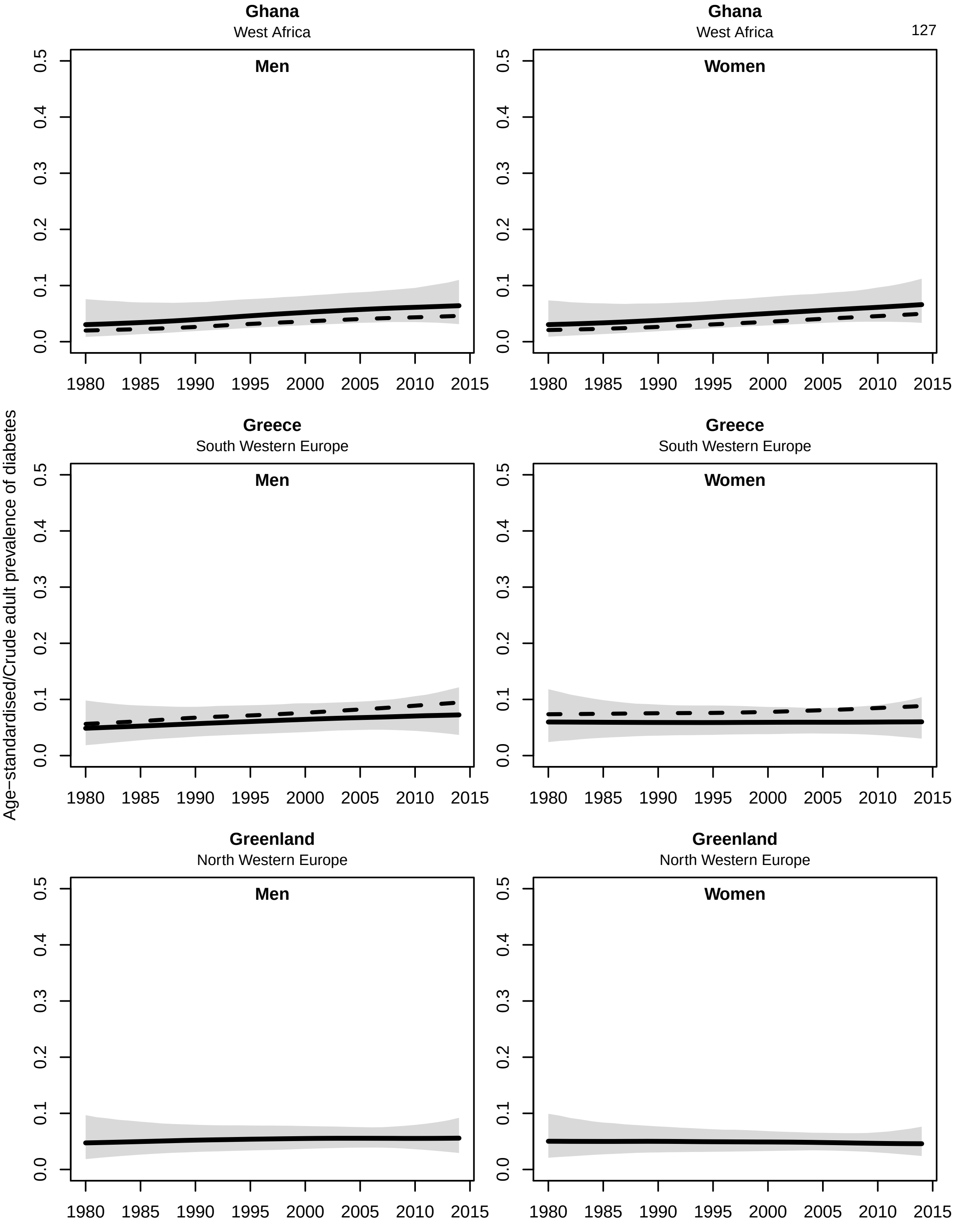
Ghana
West Africa
Men
Women
Greece
South Western Europe
Greenland
North Western Europe
Age−standardised/Crude adult prevalence of diabetes
0.0
0.1
0.2
0.3
0.4
0.5
1980
1985
1990
1995
2000
2005
2010
2015

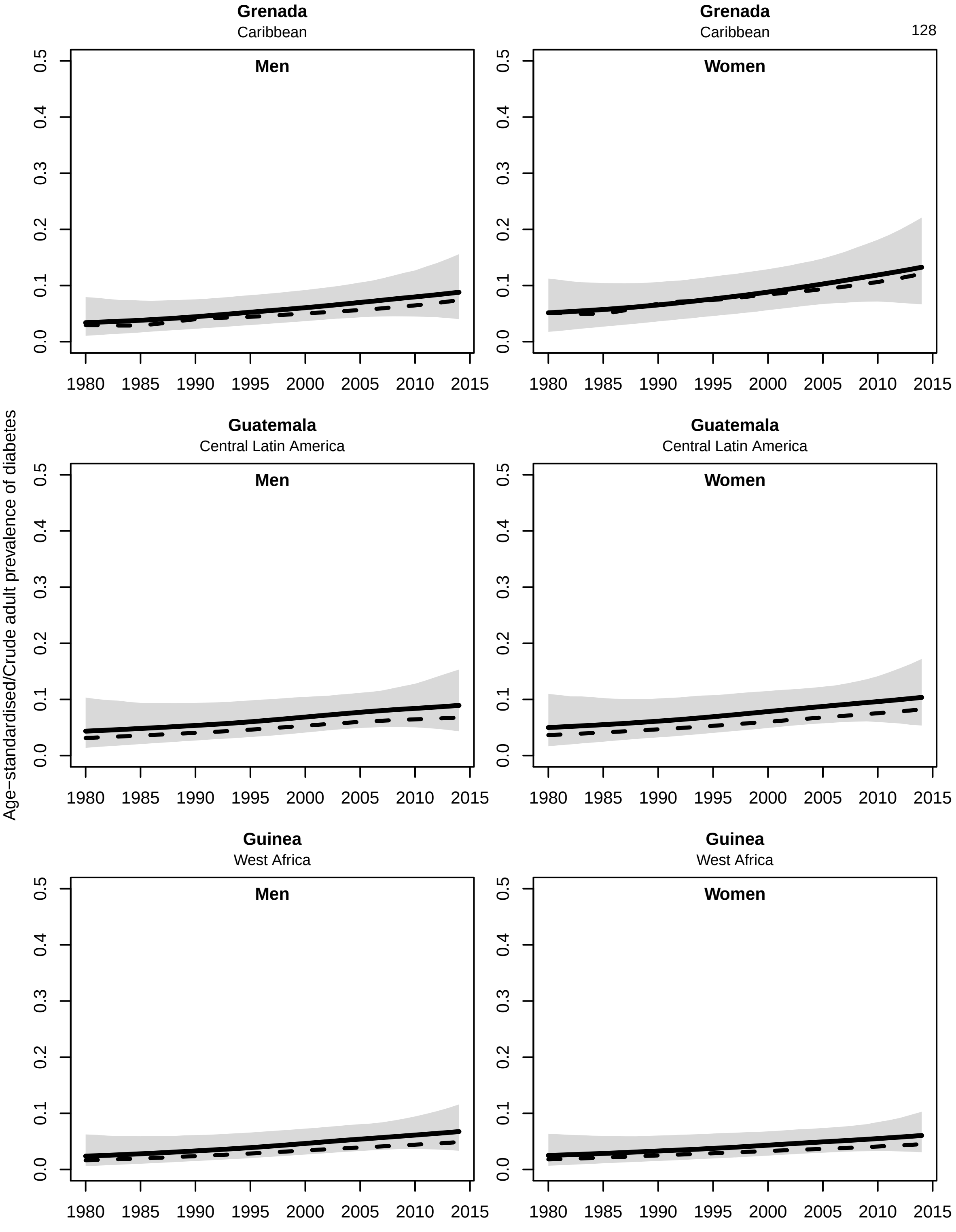
Grenada
Caribbean
Men
Grenada
Caribbean
Women
Guatemala
Central Latin America
Men
Guatemala
Central Latin America
Women
Guinea
West Africa
Men
Guinea
West Africa
Women
Age−standardised/Crude adult prevalence of diabetes
0.0
0.1
0.2
0.3
0.4
0.5
1980
1985
1990
1995
2000
2005
2010
2015

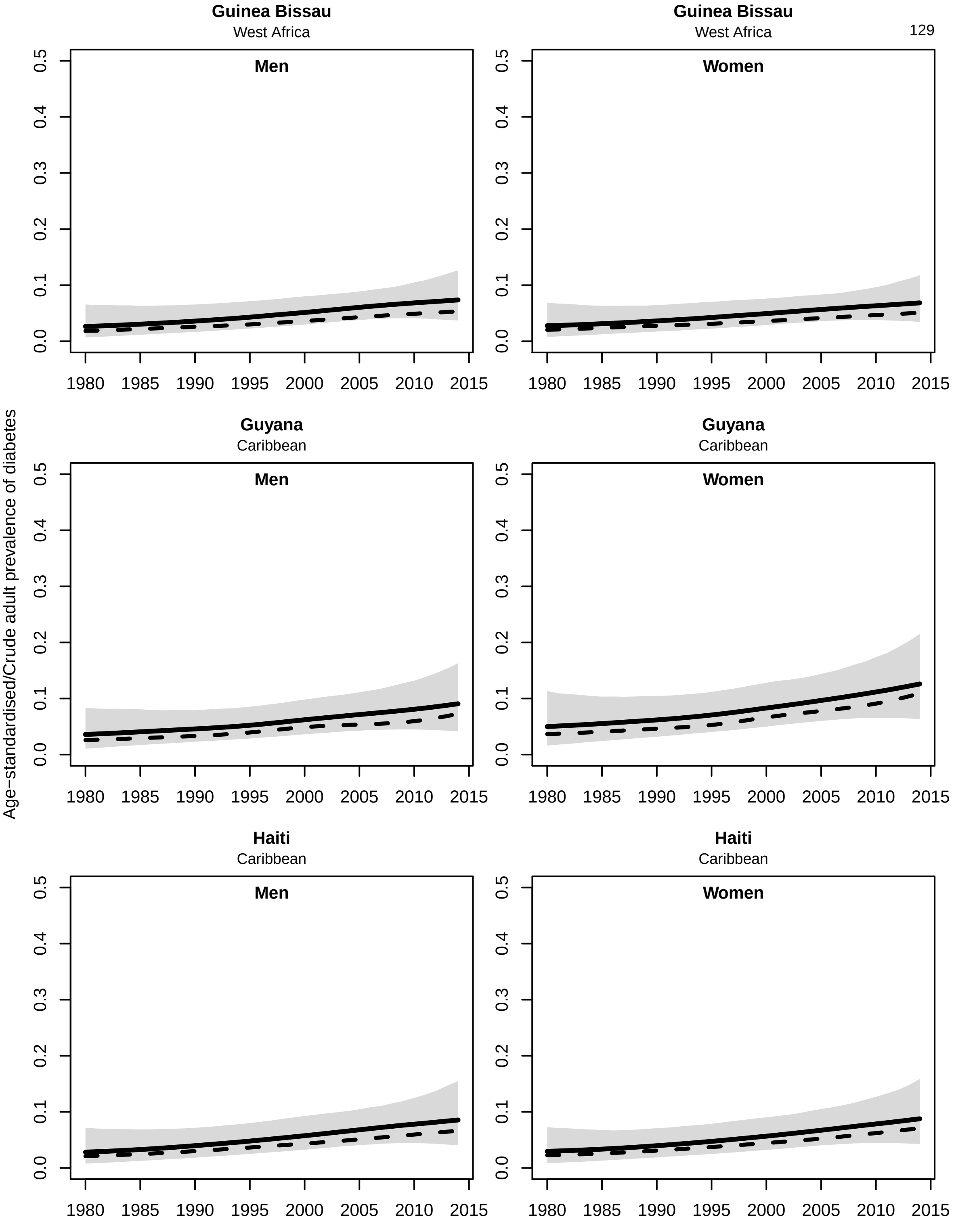
Guinea Bissau
West Africa
Men
Guinea Bissau
West Africa
Women
Guyana
Caribbean
Men
Guyana
Caribbean
Women
Haiti
Caribbean
Men
Haiti
Caribbean
Women
Age−standardised/Crude adult prevalence of diabetes
0.0
0.1
0.2
0.3
0.4
0.5
1980
1985
1990
1995
2000
2005
2010
2015

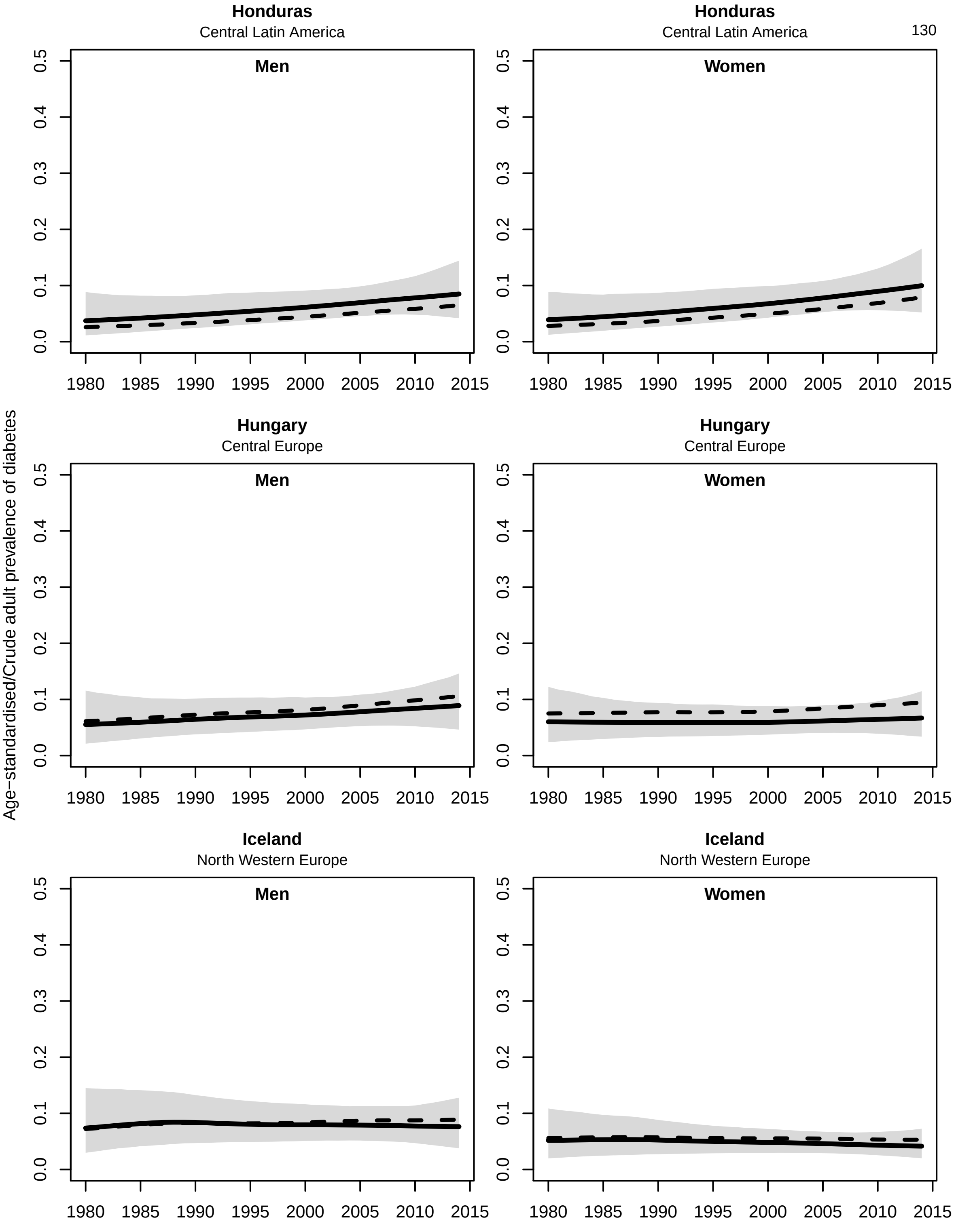
Honduras
Central Latin America
Men
Honduras
Central Latin America
Women
Hungary
Central Europe
Men
Hungary
Central Europe
Women
Iceland
North Western Europe
Men
Iceland
North Western Europe
Women
Age−standardised/Crude adult prevalence of diabetes
0.0
0.1
0.2
0.3
0.4
0.5
1980
1985
1990
1995
2000
2005
2010
2015

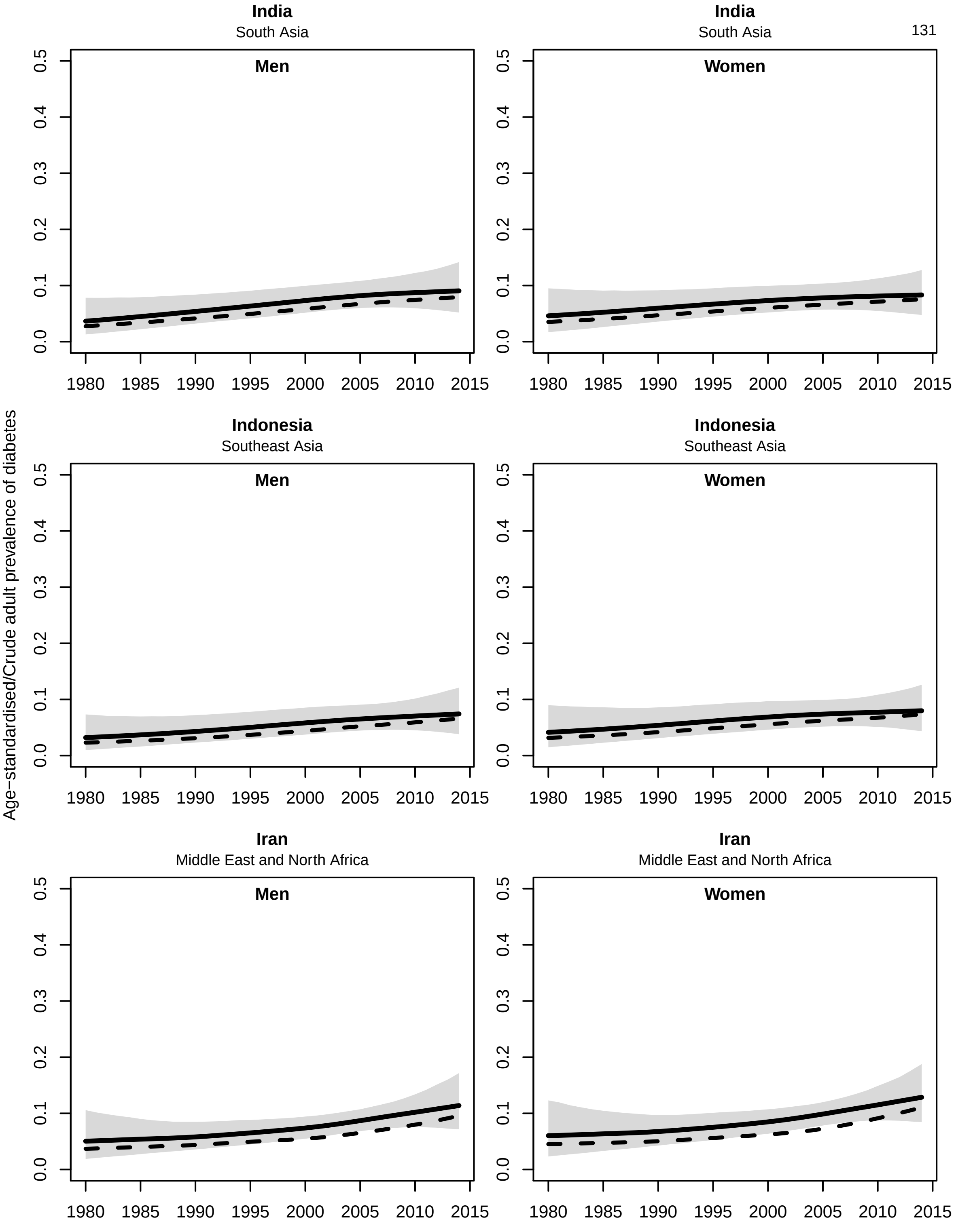
India
South Asia
Men
India
South Asia
Women
Indonesia
Southeast Asia
Men
Indonesia
Southeast Asia
Women
Iran
Middle East and North Africa
Men
Iran
Middle East and North Africa
Women
Age−standardised/Crude adult prevalence of diabetes
0.0
0.1
0.2
0.3
0.4
0.5
1980
1985
1990
1995
2000
2005
2010
2015

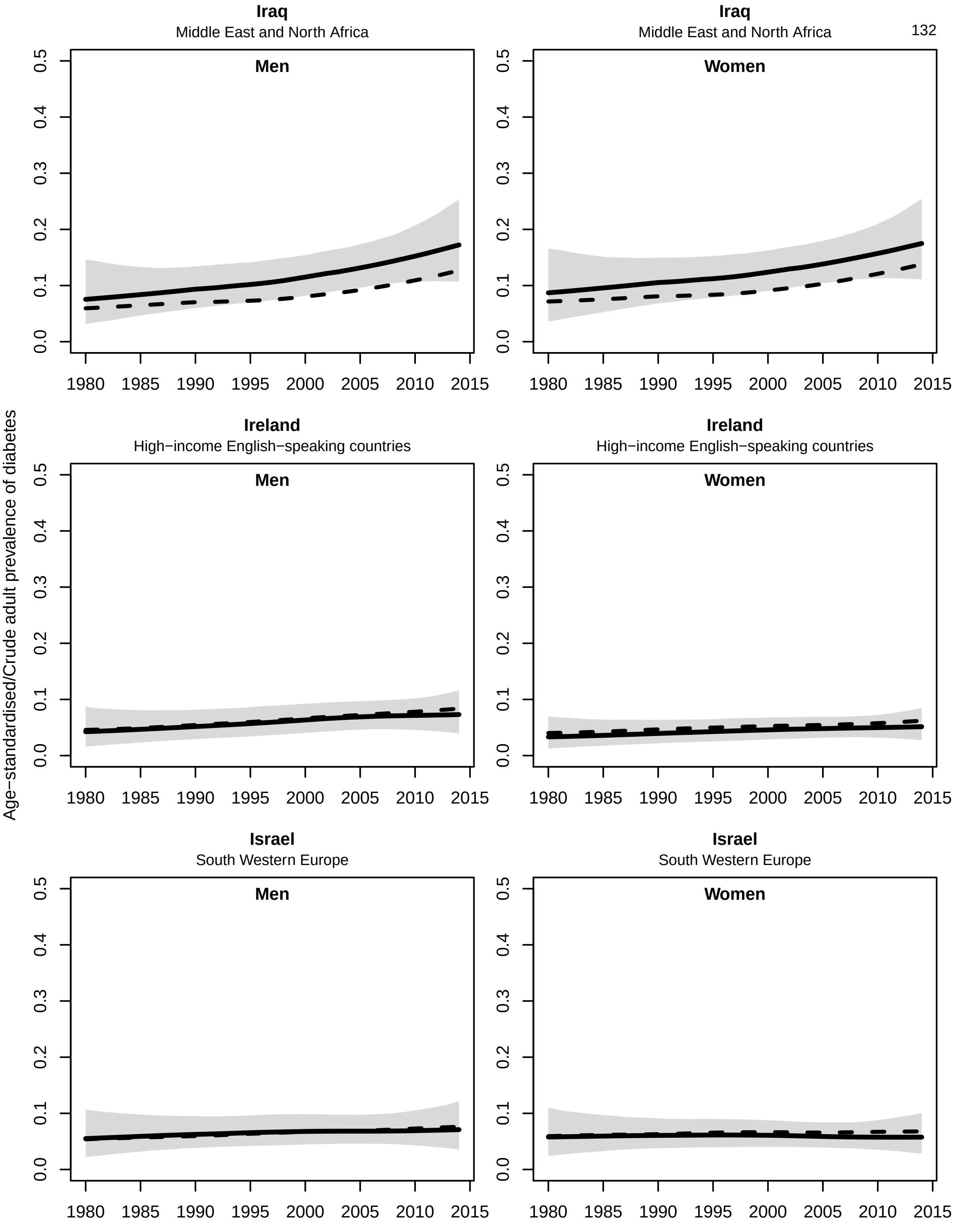
Iraq
Middle East and North Africa
Men
Iraq
Middle East and North Africa
Women
Ireland
High−income English−speaking countries
Men
Ireland
High−income English−speaking countries
Women
Israel
South Western Europe
Men
Israel
South Western Europe
Women
Age−standardised/Crude adult prevalence of diabetes
0.0
0.1
0.2
0.3
0.4
0.5
1980
1985
1990
1995
2000
2005
2010
2015

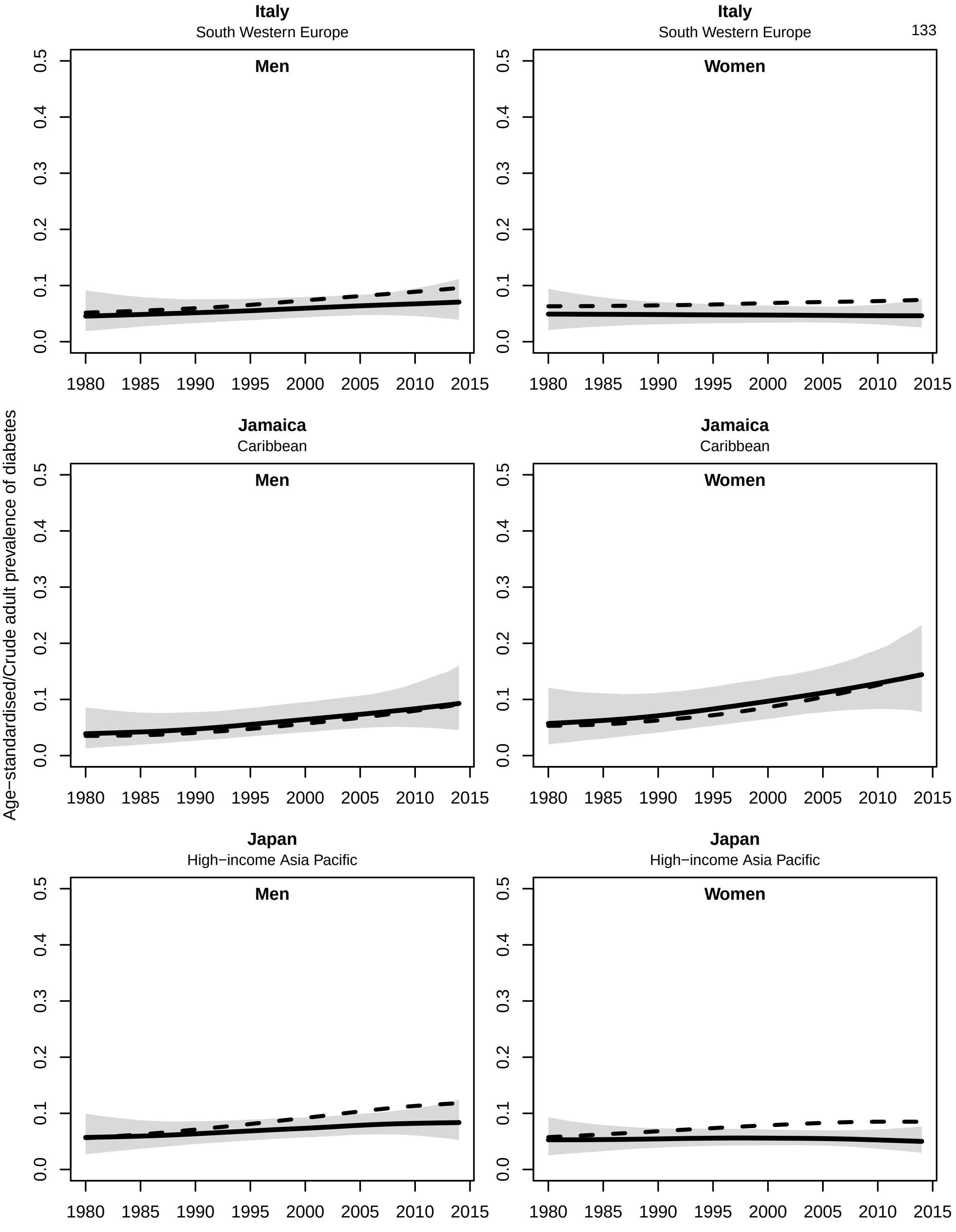
Italy
South Western Europe
Men
Italy
South Western Europe
Women
Jamaica
Caribbean
Men
Jamaica
Caribbean
Women
Japan
High−income Asia Pacific
Men
Japan
High−income Asia Pacific
Women
Age−standardised/Crude adult prevalence of diabetes
0.0 0.1 0.2 0.3 0.4 0.5
1980 1985 1990 1995 2000 2005 2010 2015

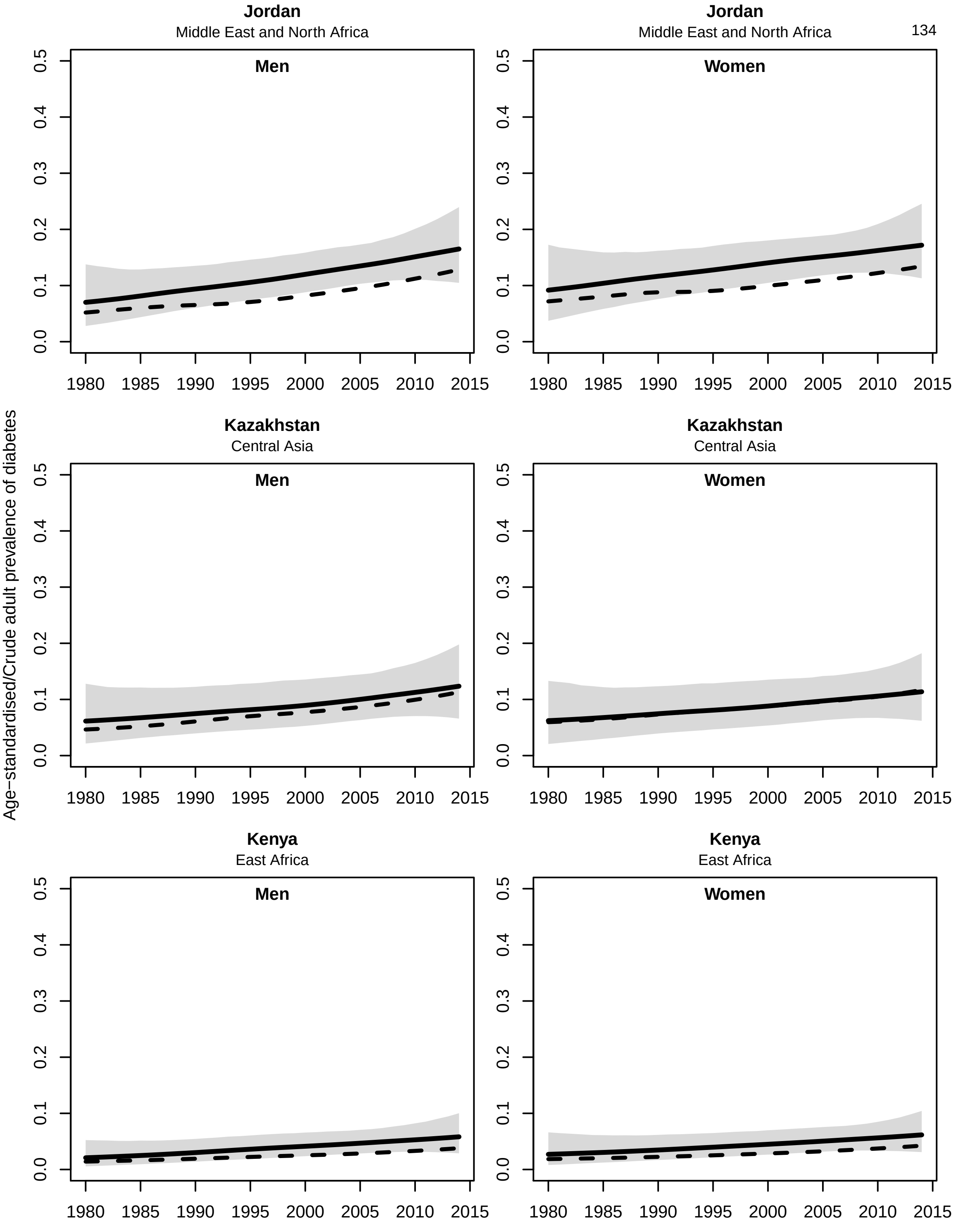
Jordan
Middle East and North Africa
Men
Jordan
Middle East and North Africa
Women
Kazakhstan
Central Asia
Men
Kazakhstan
Central Asia
Women
Kenya
East Africa
Men
Kenya
East Africa
Women
Age−standardised/Crude adult prevalence of diabetes
0.0
0.1
0.2
0.3
0.4
0.5
1980
1985
1990
1995
2000
2005
2010
2015

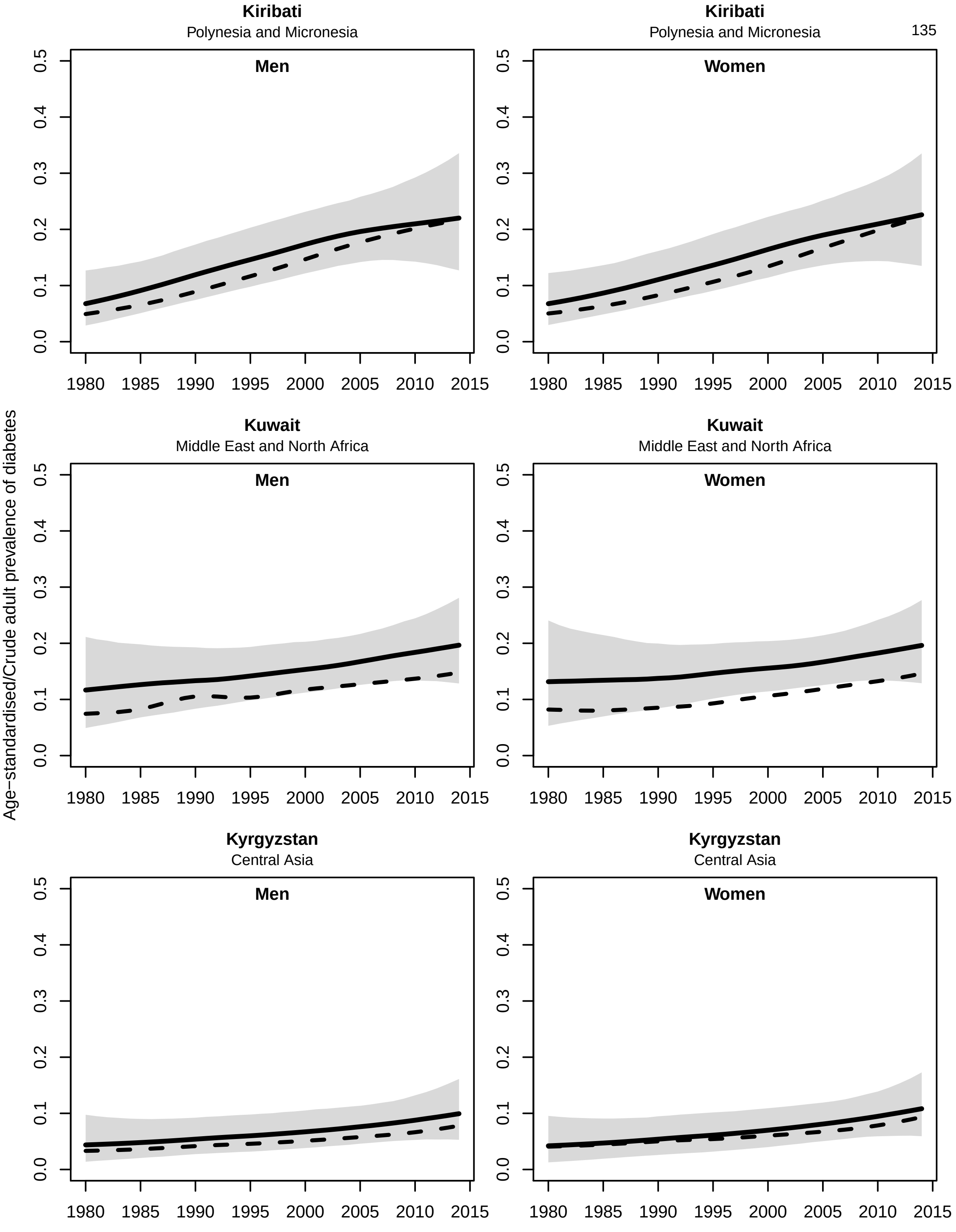
Kiribati
Polynesia and Micronesia
Men
Kiribati
Polynesia and Micronesia
Women
Kuwait
Middle East and North Africa
Men
Kuwait
Middle East and North Africa
Women
Kyrgyzstan
Central Asia
Men
Kyrgyzstan
Central Asia
Women
Age−standardised/Crude adult prevalence of diabetes
0.0
0.1
0.2
0.3
0.4
0.5
1980
1985
1990
1995
2000
2005
2010
2015

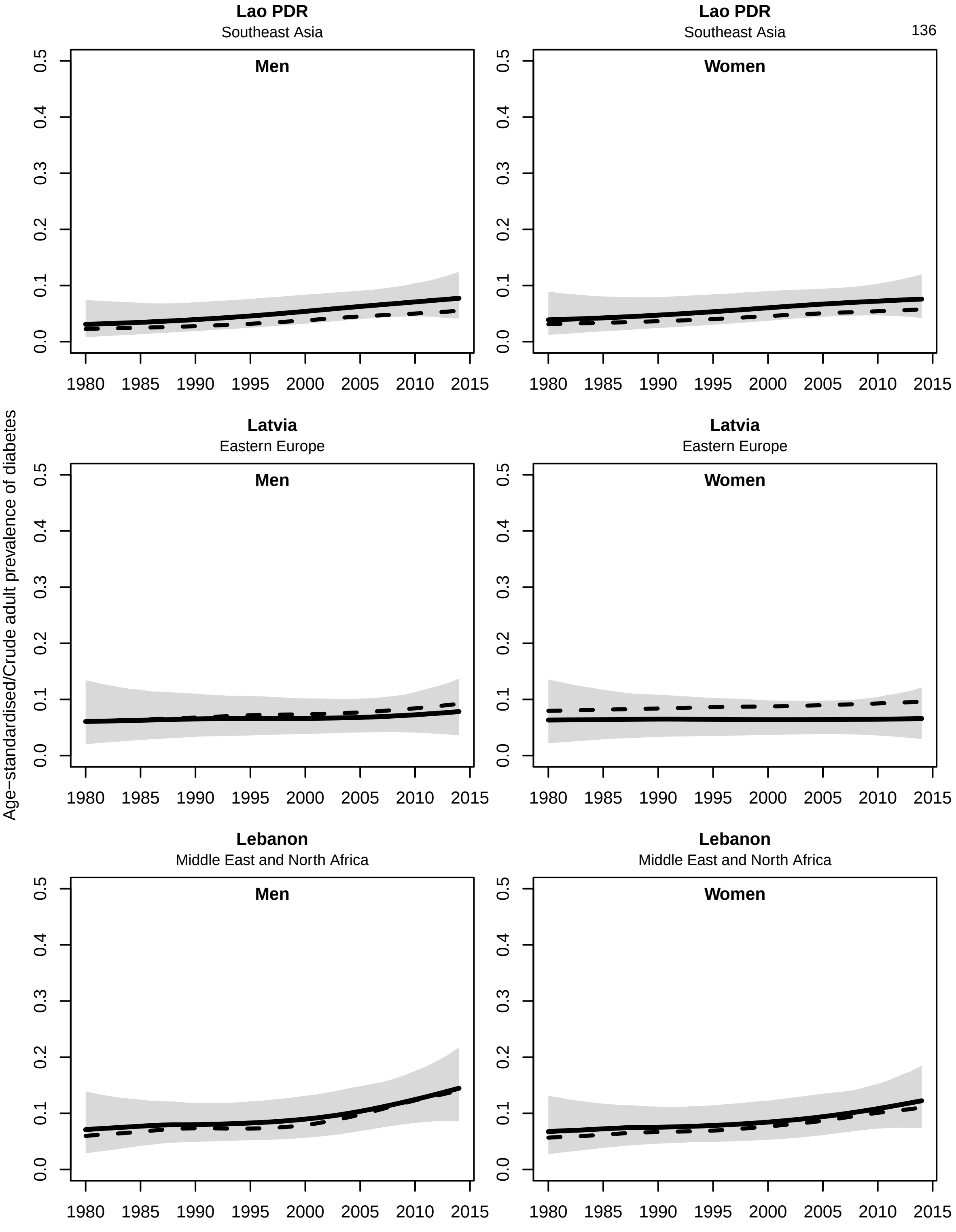

Lao PDR
Southeast Asia
Men
Lao PDR
Southeast Asia
Women
Latvia
Eastern Europe
Men
Latvia
Eastern Europe
Women
Lebanon
Middle East and North Africa
Men
Lebanon
Middle East and North Africa
Women
Age−standardised/Crude adult prevalence of diabetes
0.0
0.1
0.2
0.3
0.4
0.5
1980
1985
1990
1995
2000
2005
2010
2015

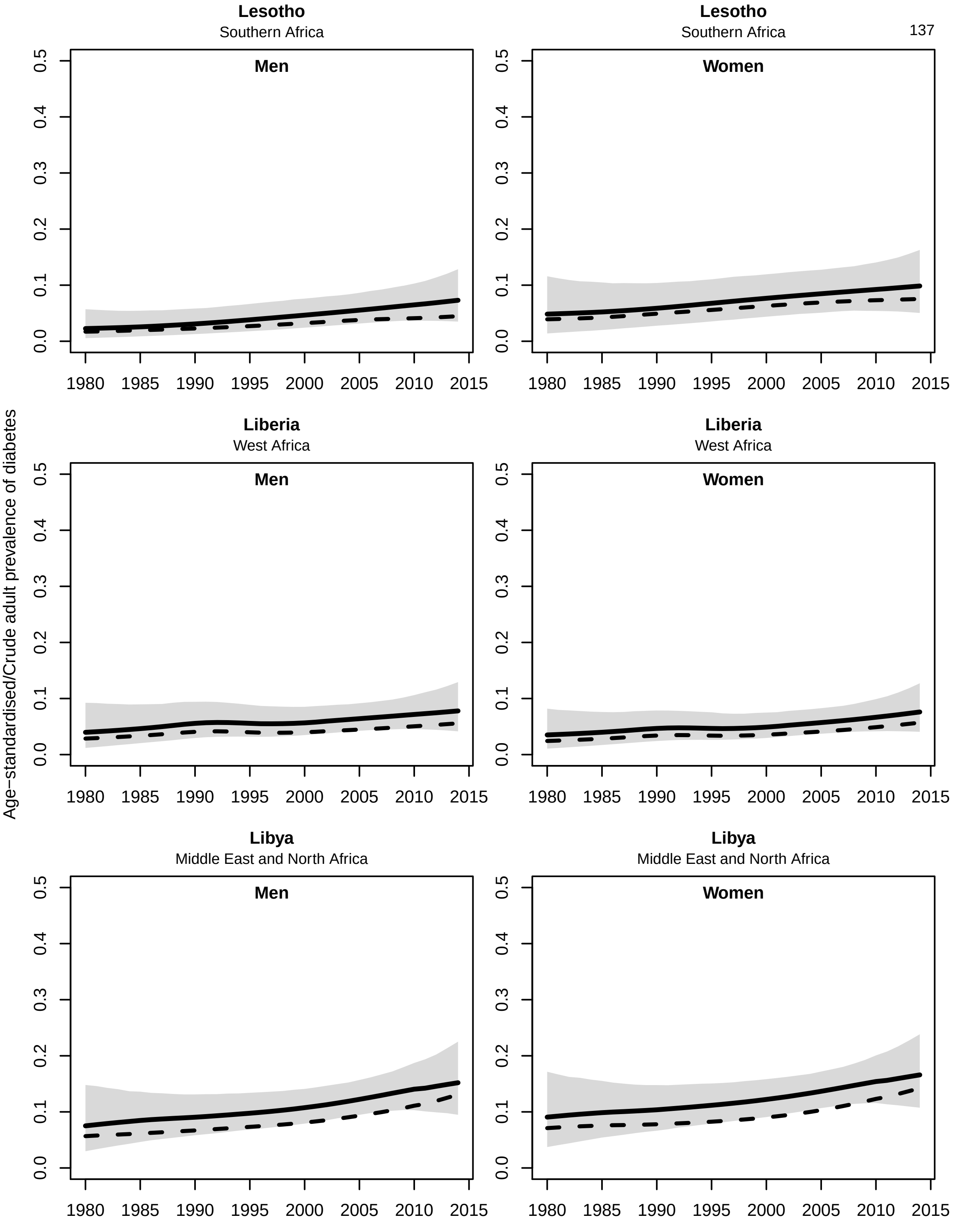
Lesotho
Southern Africa
Men
Lesotho
Southern Africa
Women
Liberia
West Africa
Men
Liberia
West Africa
Women
Libya
Middle East and North Africa
Men
Libya
Middle East and North Africa
Women
Age−standardised/Crude adult prevalence of diabetes
0.0
0.1
0.2
0.3
0.4
0.5
1980
1985
1990
1995
2000
2005
2010
2015

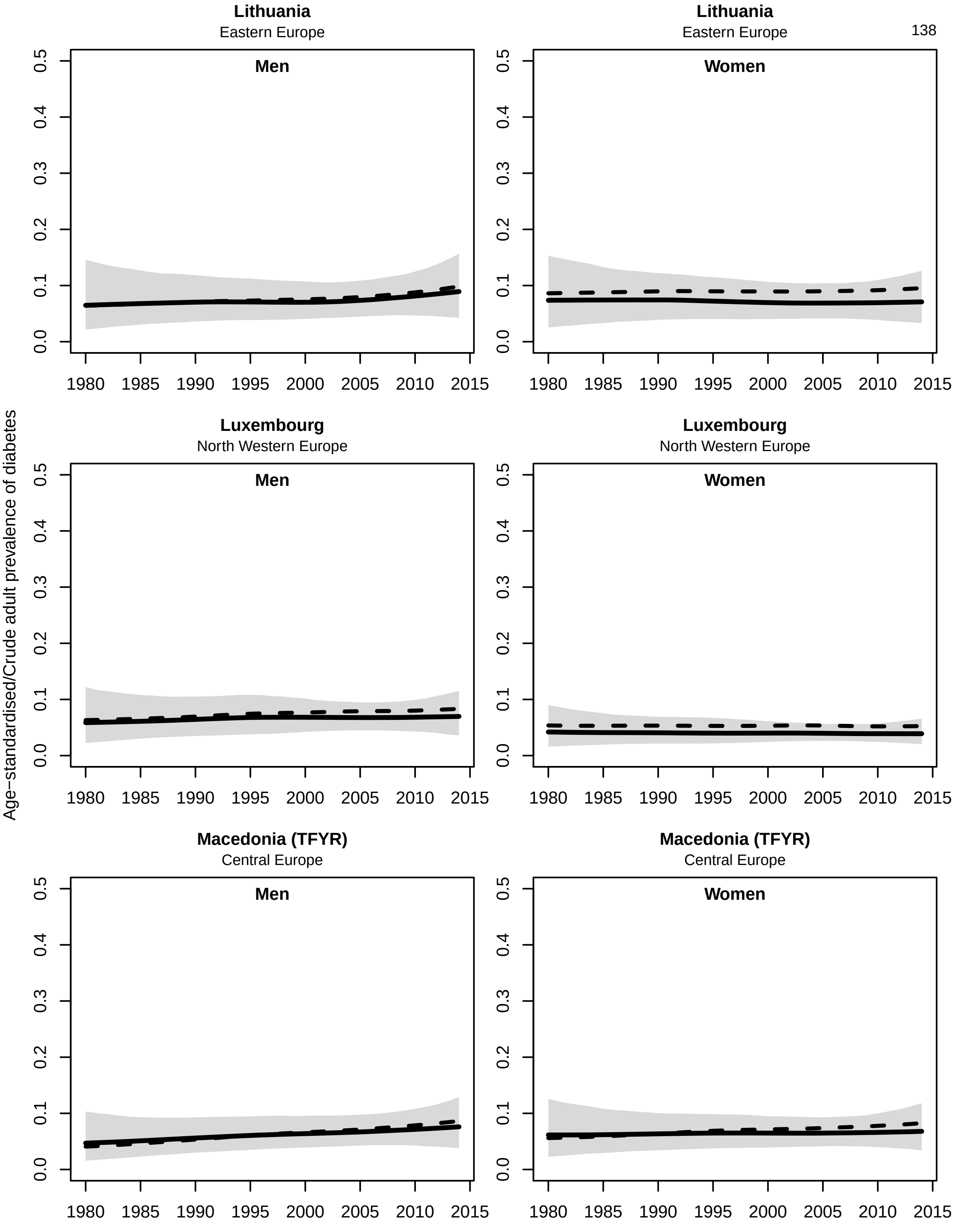
Lithuania
Eastern Europe
Men
Lithuania
Eastern Europe
Women
Luxembourg
North Western Europe
Men
Luxembourg
North Western Europe
Women
Macedonia (TFYR)
Central Europe
Men
Macedonia (TFYR)
Central Europe
Women
Age−standardised/Crude adult prevalence of diabetes
0.0
0.1
0.2
0.3
0.4
0.5
1980
1985
1990
1995
2000
2005
2010
2015

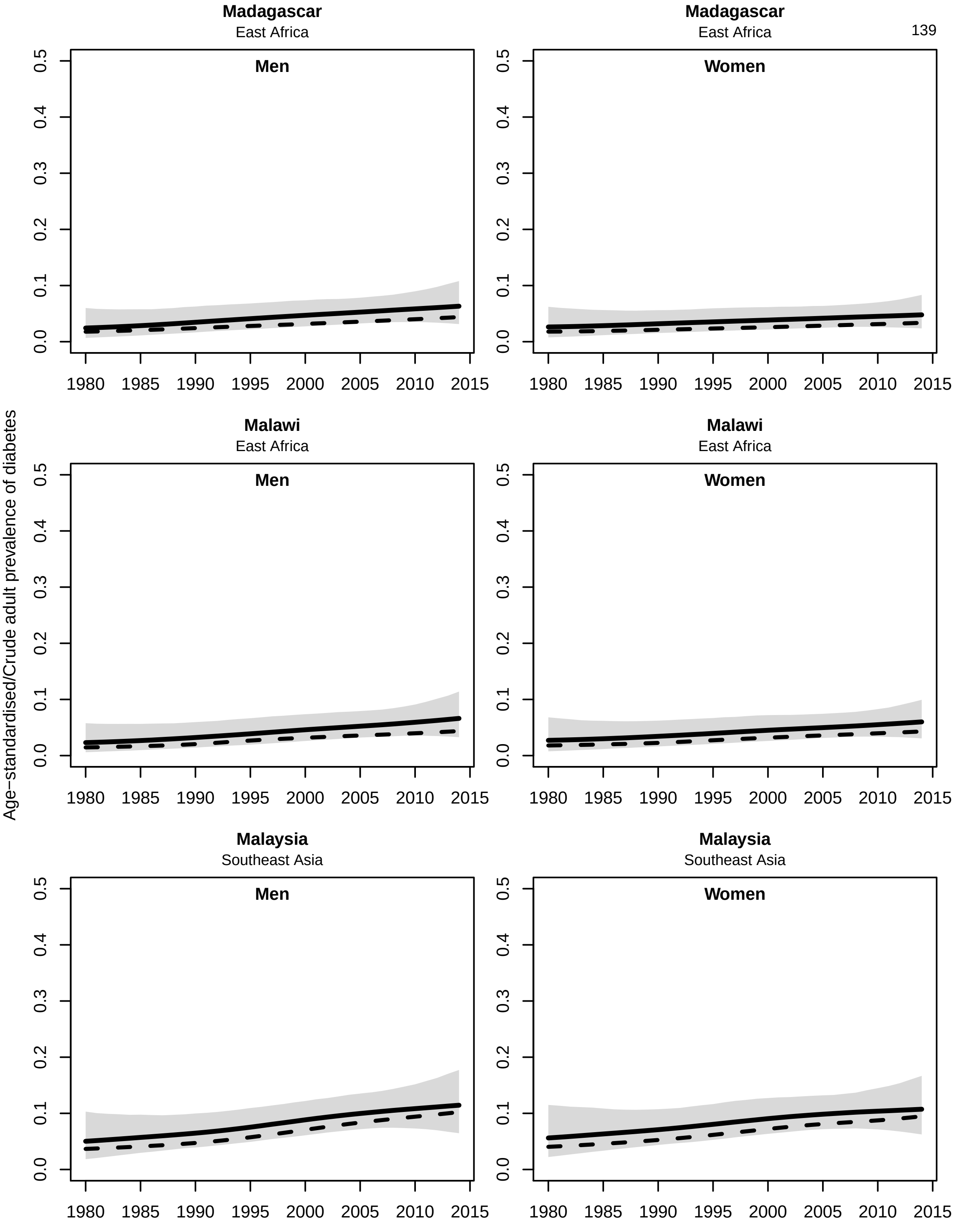

Madagascar
East Africa
Men
Madagascar
East Africa
Women
Malawi
East Africa
Men
Malawi
East Africa
Women
Malaysia
Southeast Asia
Men
Malaysia
Southeast Asia
Women
Age−standardised/Crude adult prevalence of diabetes
0.0
0.1
0.2
0.3
0.4
0.5
1980
1985
1990
1995
2000
2005
2010
2015

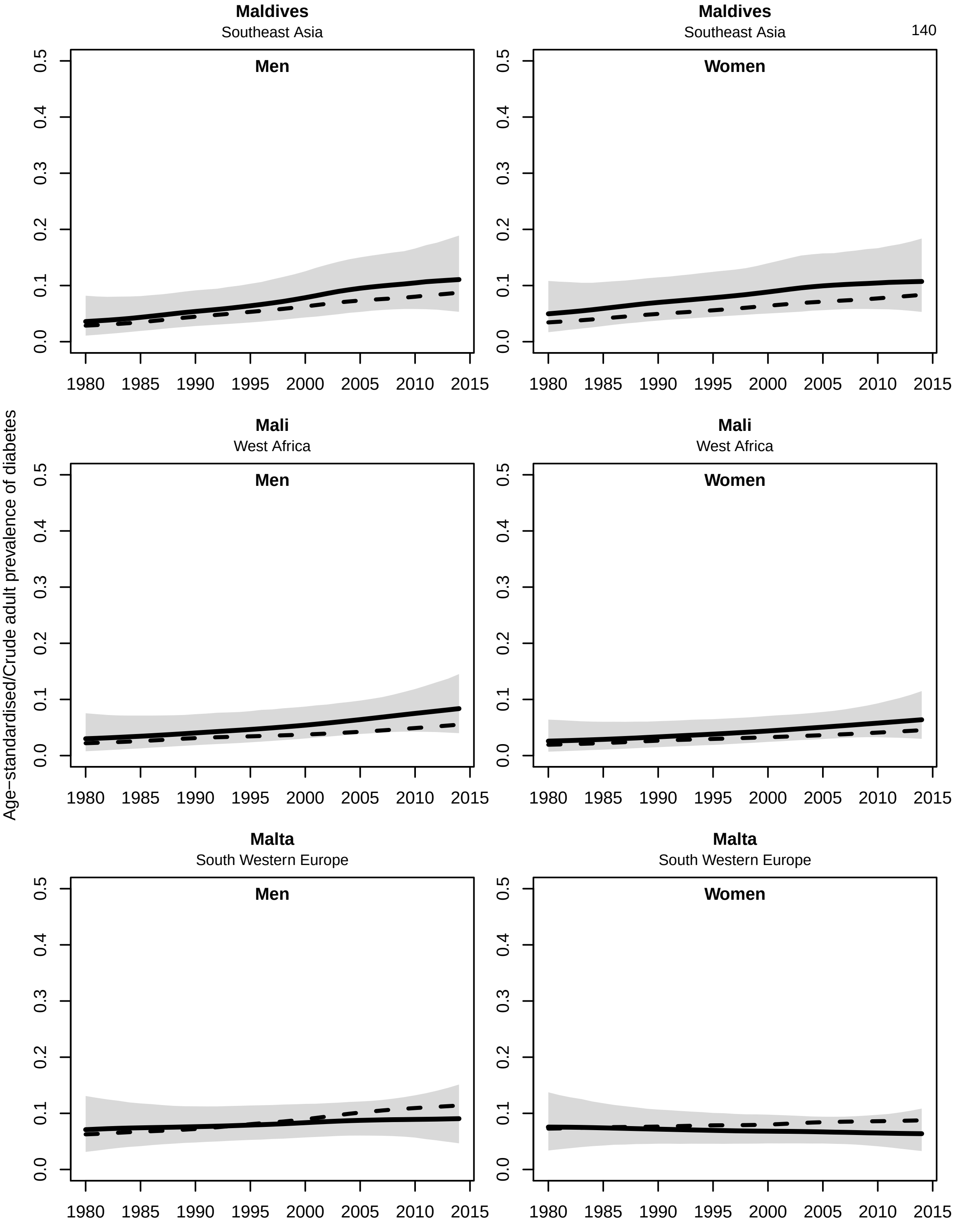
Maldives
Southeast Asia
Men
Maldives
Southeast Asia
Women
Mali
West Africa
Men
Mali
West Africa
Women
Malta
South Western Europe
Men
Malta
South Western Europe
Women
Age−standardised/Crude adult prevalence of diabetes
0.0
0.1
0.2
0.3
0.4
0.5
1980
1985
1990
1995
2000
2005
2010
2015

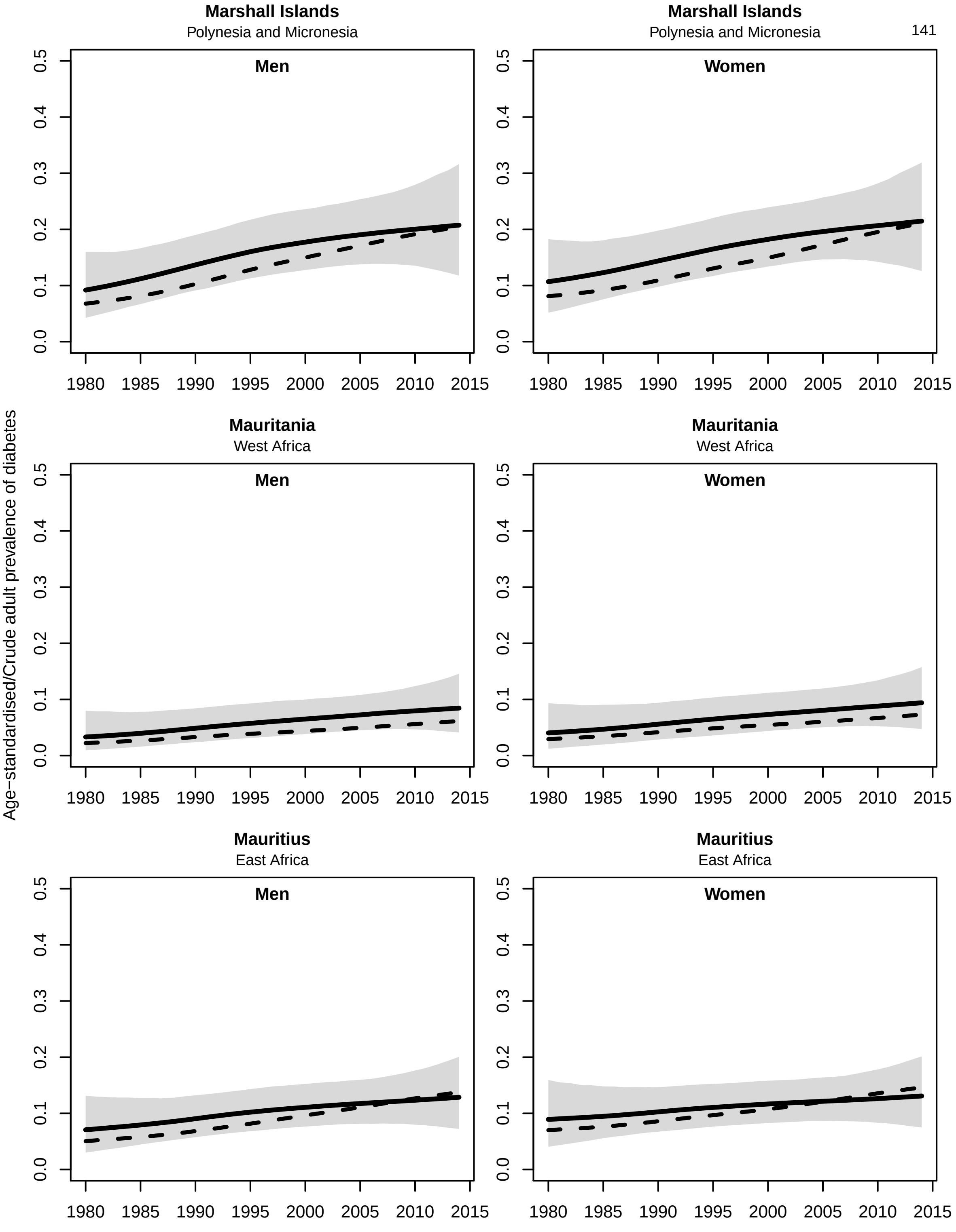
Marshall Islands
Polynesia and Micronesia
Men
Women
Mauritania
West Africa
Men
Women
Mauritius
East Africa
Men
Women
Age−standardised/Crude adult prevalence of diabetes
0.0
0.1
0.2
0.3
0.4
0.5
1980
1985
1990
1995
2000
2005
2010
2015

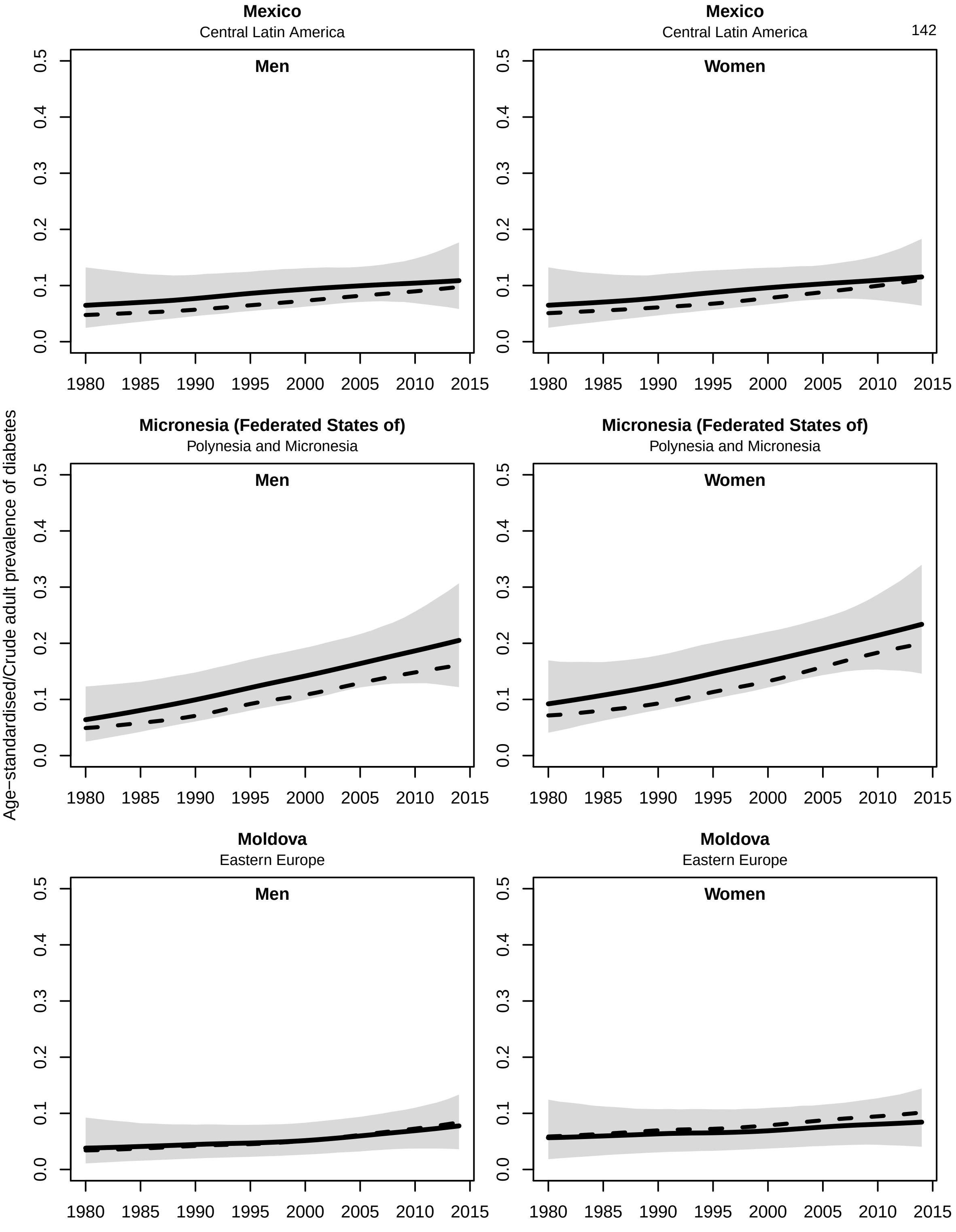
Mexico
Central Latin America
Men
Mexico
Central Latin America
Women
Micronesia (Federated States of)
Polynesia and Micronesia
Men
Micronesia (Federated States of)
Polynesia and Micronesia
Women
Moldova
Eastern Europe
Men
Moldova
Eastern Europe
Women
Age−standardised/Crude adult prevalence of diabetes
0.0
0.1
0.2
0.3
0.4
0.5
1980
1985
1990
1995
2000
2005
2010
2015

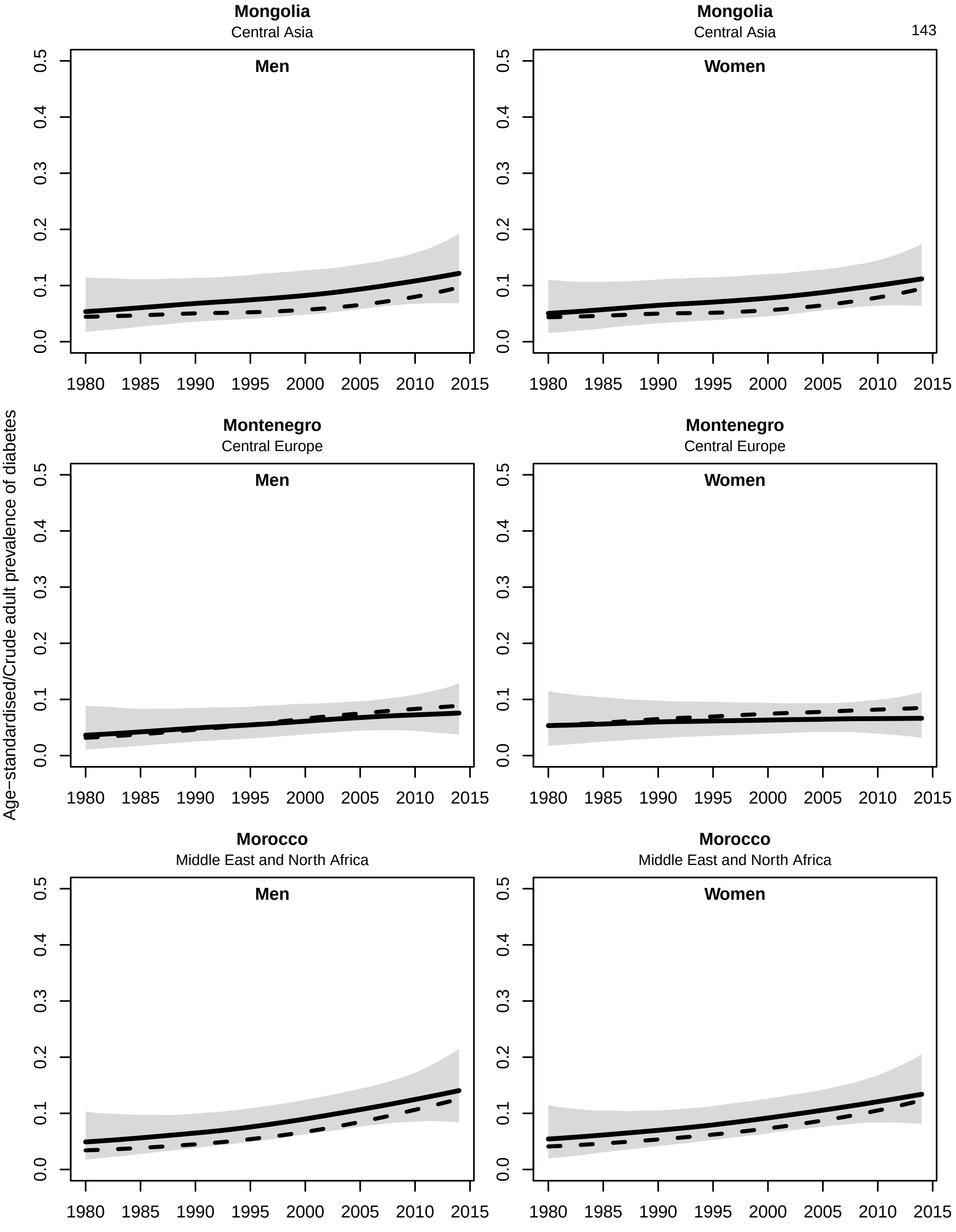
Mongolia
Central Asia
Men
Mongolia
Central Asia
Women
Montenegro
Central Europe
Men
Montenegro
Central Europe
Women
Morocco
Middle East and North Africa
Men
Morocco
Middle East and North Africa
Women
Age−standardised/Crude adult prevalence of diabetes
0.0
0.1
0.2
0.3
0.4
0.5
1980
1985
1990
1995
2000
2005
2010
2015

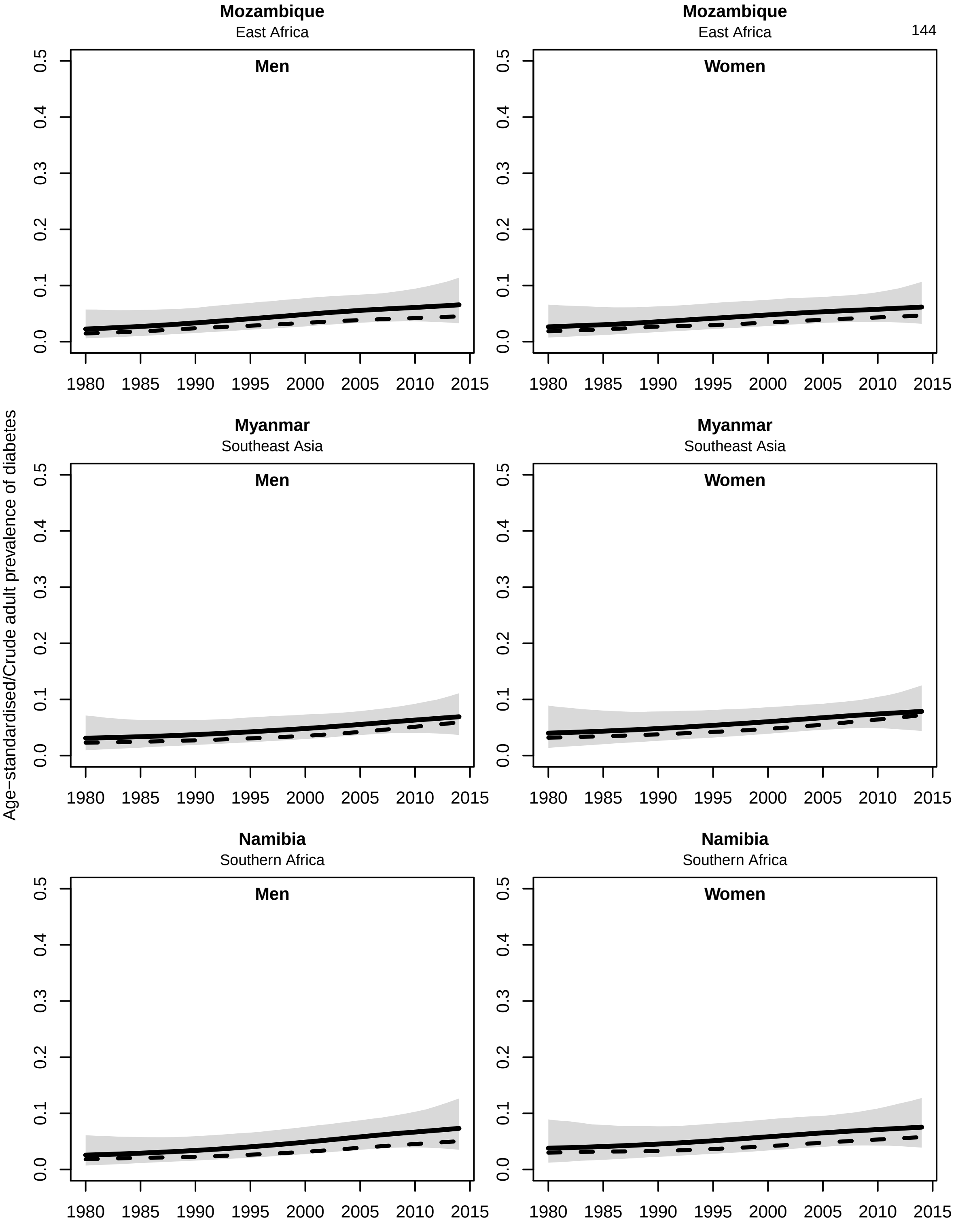
Mozambique
East Africa
Men
Mozambique
East Africa
Women
Myanmar
Southeast Asia
Men
Myanmar
Southeast Asia
Women
Namibia
Southern Africa
Men
Namibia
Southern Africa
Women
Age−standardised/Crude adult prevalence of diabetes
0.0
0.1
0.2
0.3
0.4
0.5
1980
1985
1990
1995
2000
2005
2010
2015

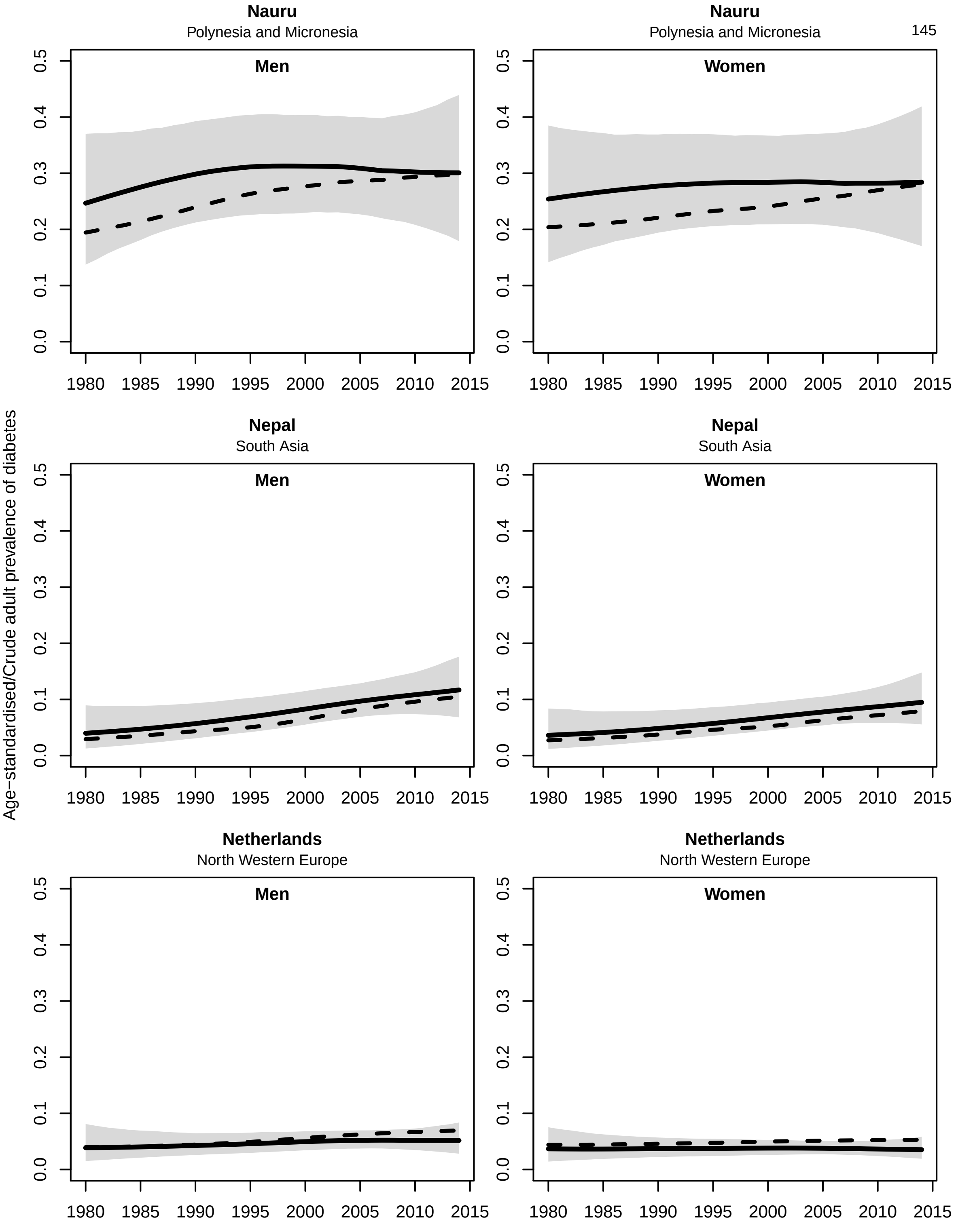
Nauru
Polynesia and Micronesia
Men
Nauru
Polynesia and Micronesia
Women
Nepal
South Asia
Men
Nepal
South Asia
Women
Netherlands
North Western Europe
Men
Netherlands
North Western Europe
Women
Age−standardised/Crude adult prevalence of diabetes
0.0
0.1
0.2
0.3
0.4
0.5
1980
1985
1990
1995
2000
2005
2010
2015

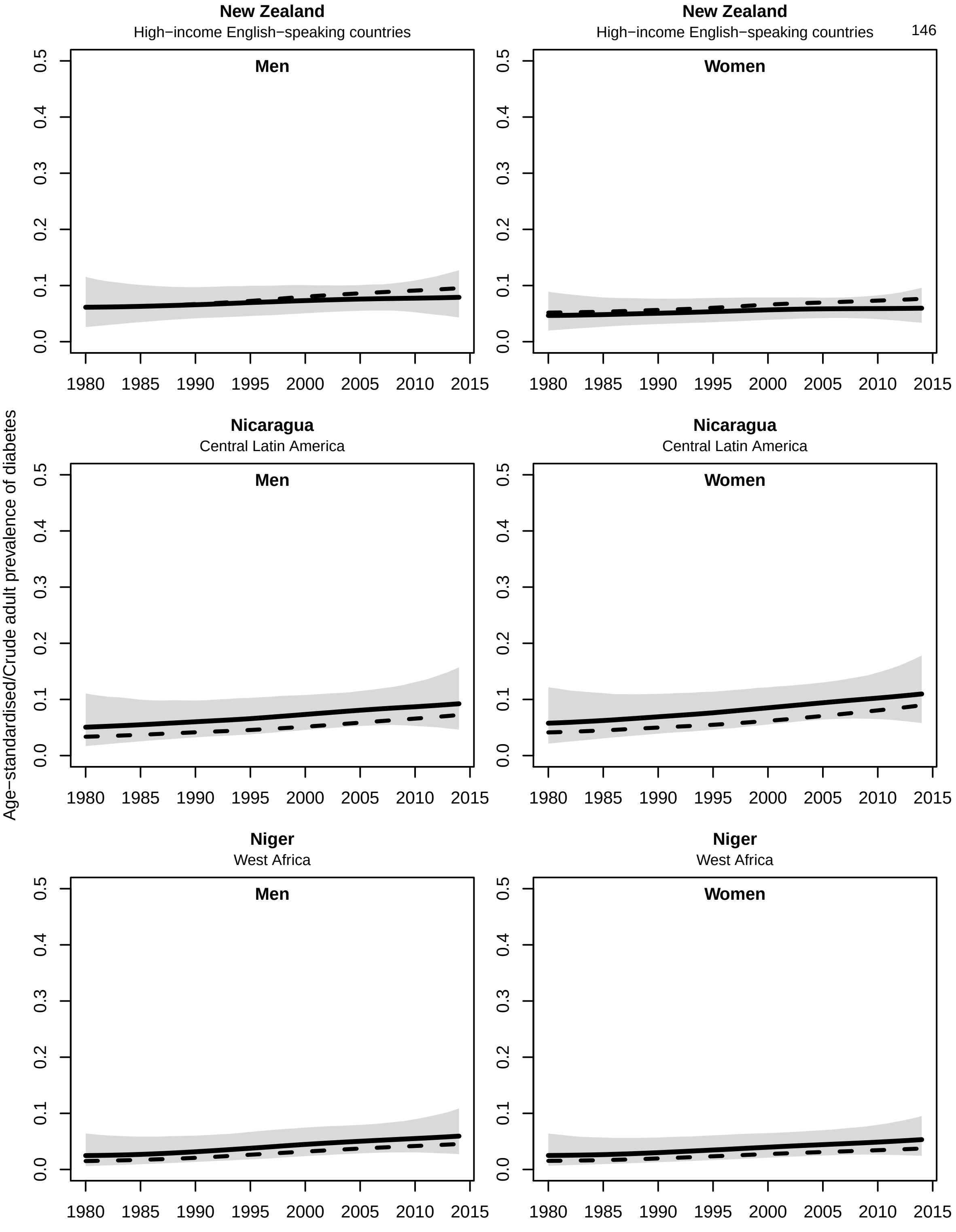
New Zealand
High−income English−speaking countries
Men
New Zealand
High−income English−speaking countries
Women
Nicaragua
Central Latin America
Men
Nicaragua
Central Latin America
Women
Niger
West Africa
Men
Niger
West Africa
Women
Age−standardised/Crude adult prevalence of diabetes
0.0
0.1
0.2
0.3
0.4
0.5
1980
1985
1990
1995
2000
2005
2010
2015

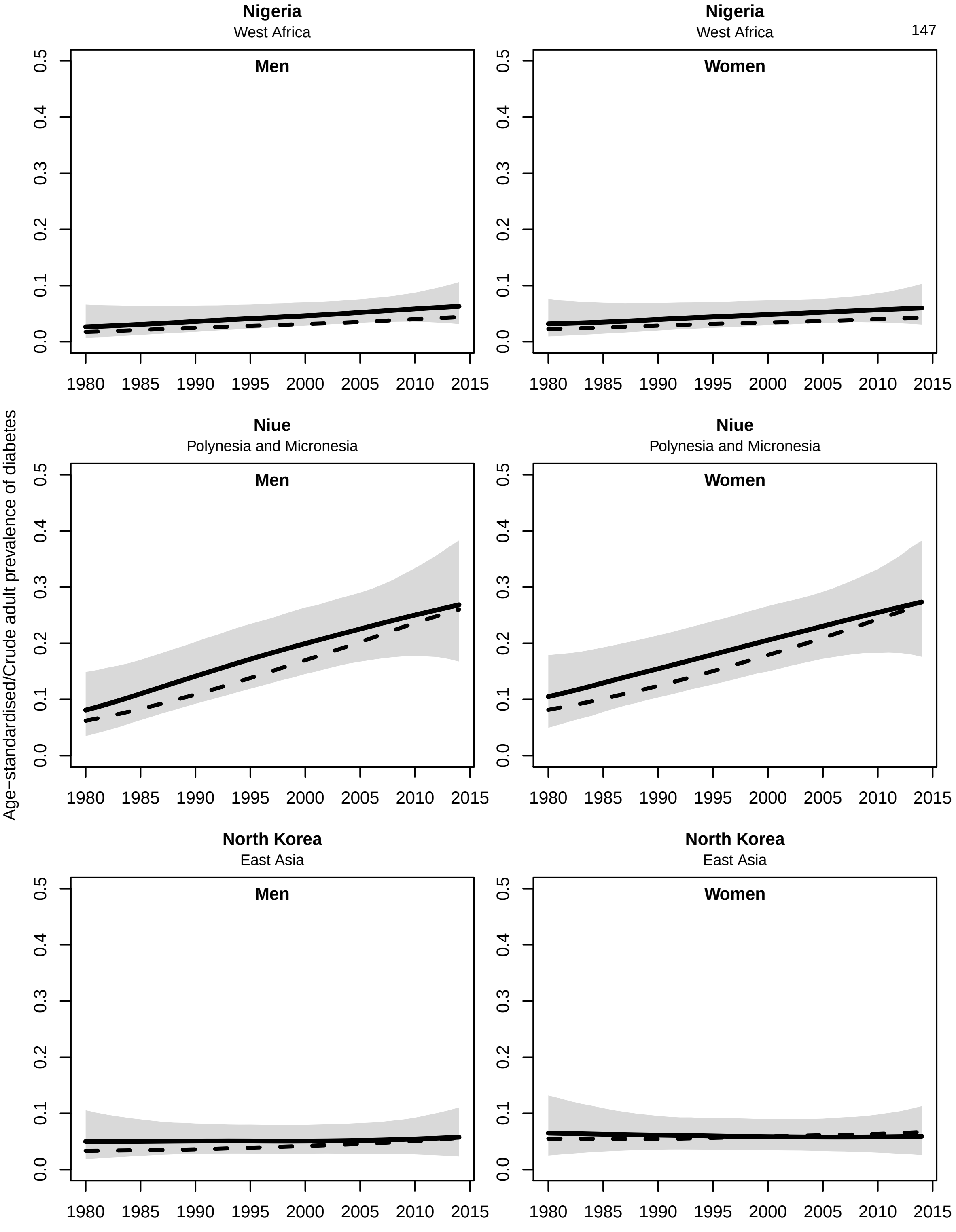
Nigeria
West Africa
Men
Nigeria
West Africa
Women
Niue
Polynesia and Micronesia
Men
Niue
Polynesia and Micronesia
Women
North Korea
East Asia
Men
North Korea
East Asia
Women
Age−standardised/Crude adult prevalence of diabetes
0.0
0.1
0.2
0.3
0.4
0.5
1980
1985
1990
1995
2000
2005
2010
2015

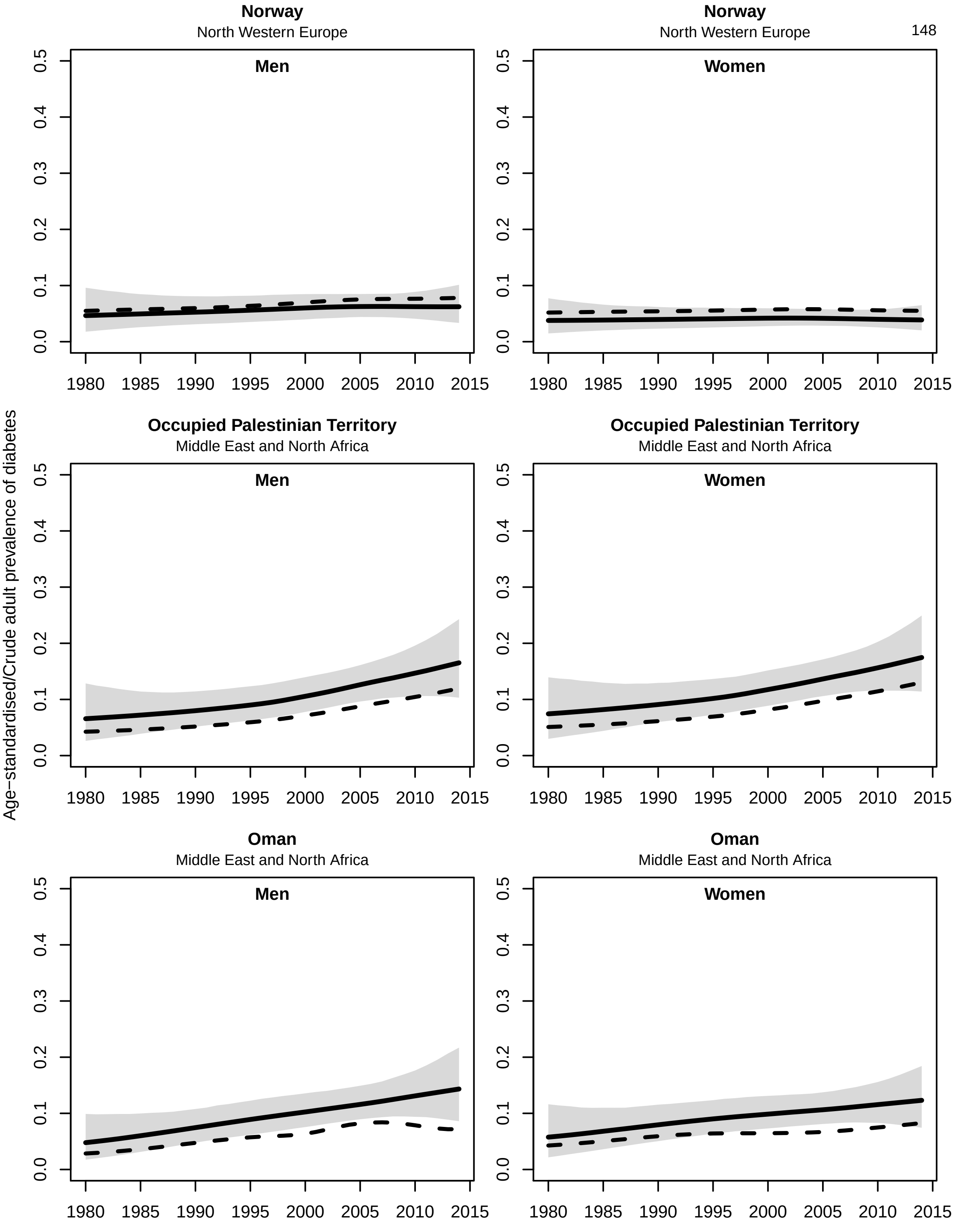
Norway
North Western Europe
Men
Norway
North Western Europe
Women
Occupied Palestinian Territory
Middle East and North Africa
Men
Occupied Palestinian Territory
Middle East and North Africa
Women
Oman
Middle East and North Africa
Men
Oman
Middle East and North Africa
Women
Age−standardised/Crude adult prevalence of diabetes
0.0
0.1
0.2
0.3
0.4
0.5
1980
1985
1990
1995
2000
2005
2010
2015

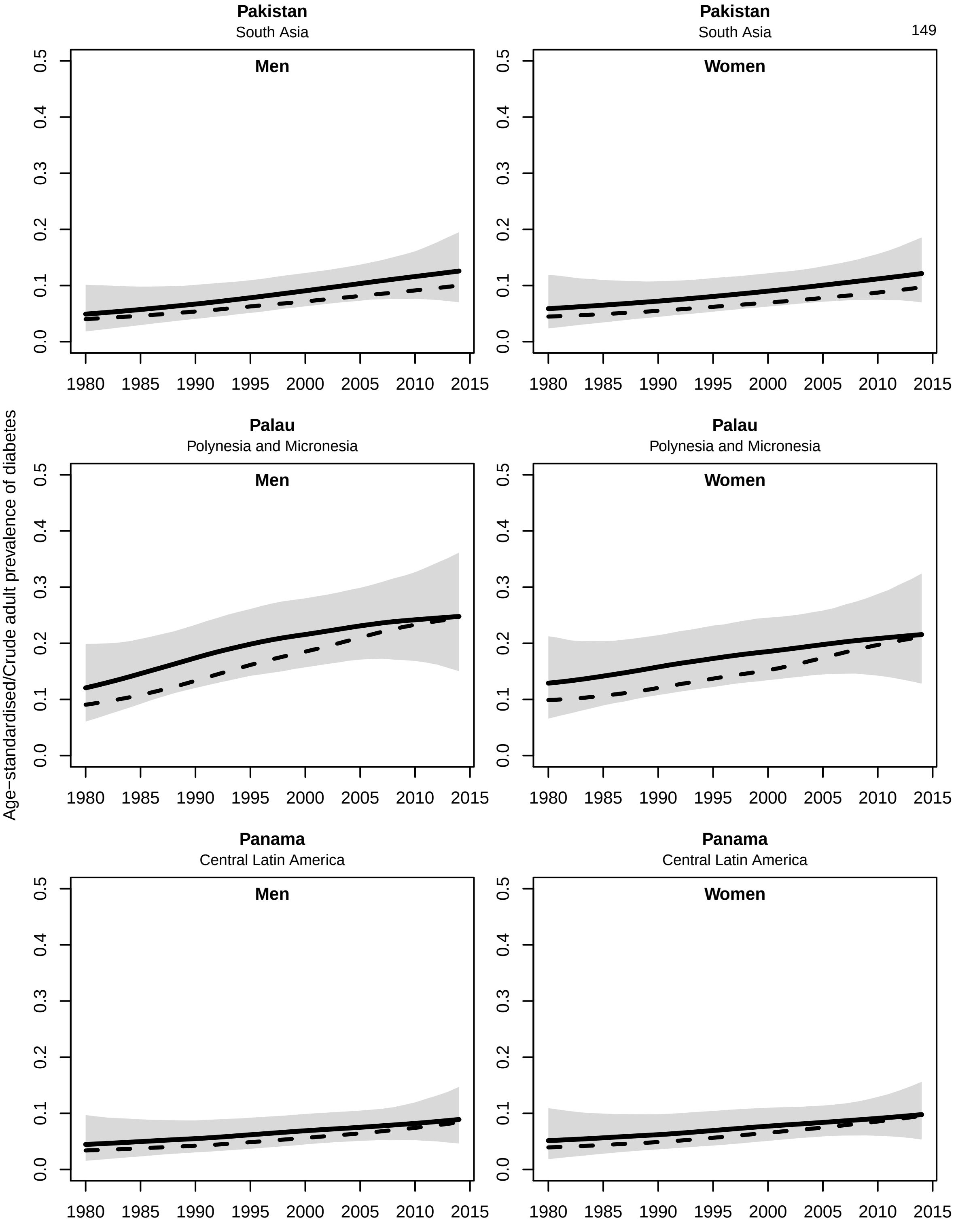
Pakistan
South Asia
Men
Pakistan
South Asia
Women
Palau
Polynesia and Micronesia
Men
Palau
Polynesia and Micronesia
Women
Panama
Central Latin America
Men
Panama
Central Latin America
Women
Age−standardised/Crude adult prevalence of diabetes
0.0
0.1
0.2
0.3
0.4
0.5
1980
1985
1990
1995
2000
2005
2010
2015

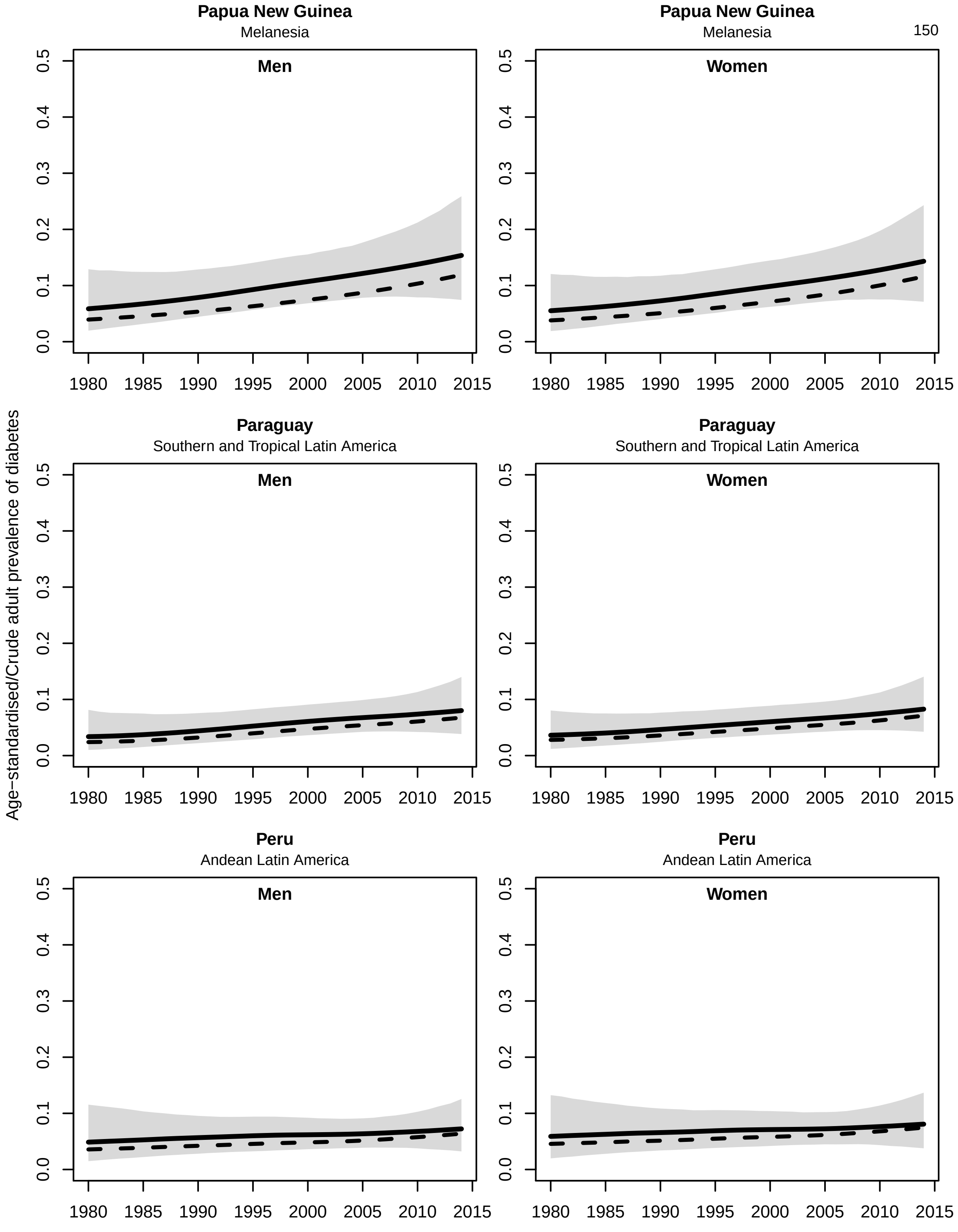
Papua New Guinea
Melanesia
Men
Papua New Guinea
Melanesia
Women
Paraguay
Southern and Tropical Latin America
Men
Paraguay
Southern and Tropical Latin America
Women
Peru
Andean Latin America
Men
Peru
Andean Latin America
Women
Age−standardised/Crude adult prevalence of diabetes
0.0
0.1
0.2
0.3
0.4
0.5
1980
1985
1990
1995
2000
2005
2010
2015

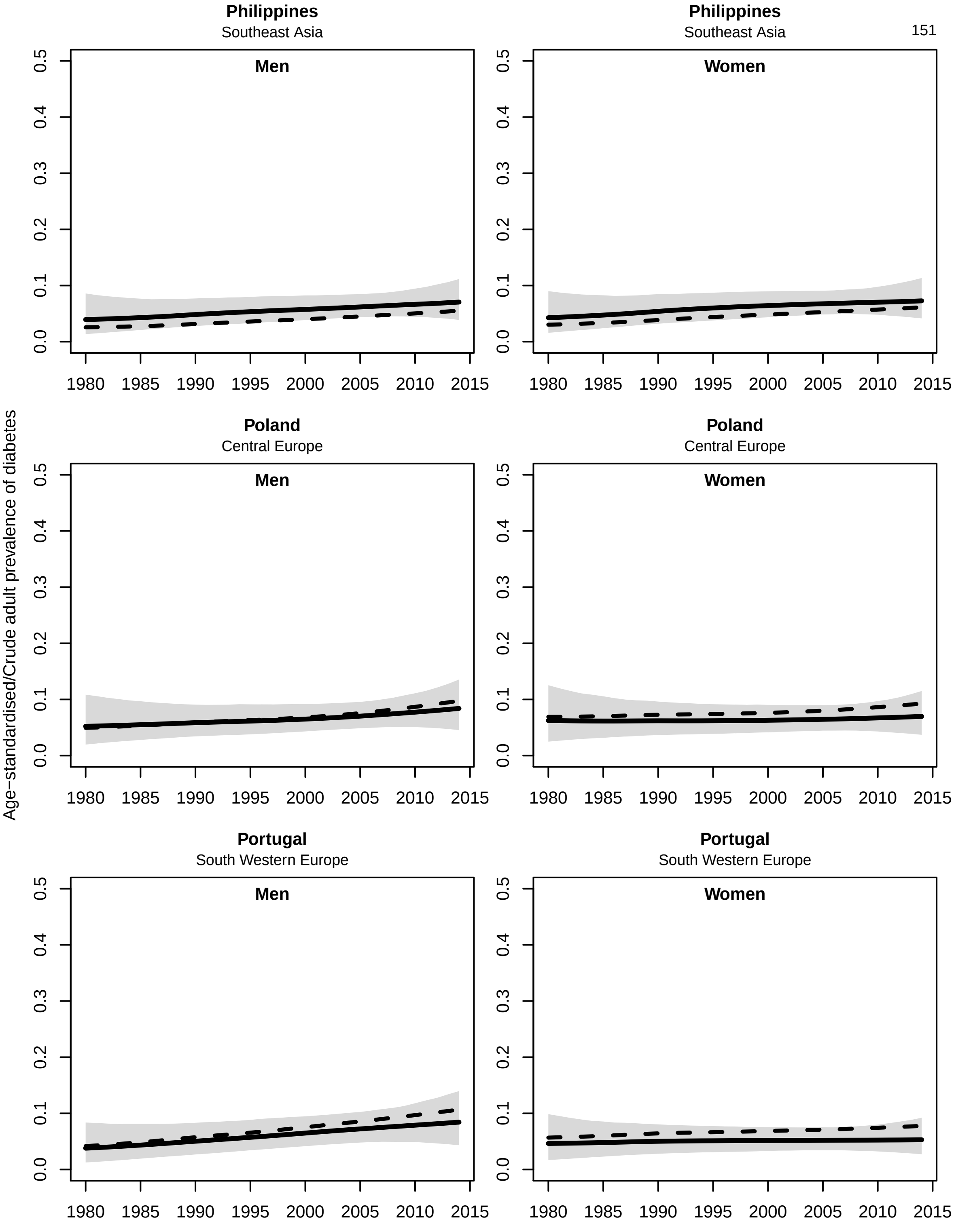
Philippines
Southeast Asia
Men
Philippines
Southeast Asia
Women
Poland
Central Europe
Men
Poland
Central Europe
Women
Portugal
South Western Europe
Men
Portugal
South Western Europe
Women
Age−standardised/Crude adult prevalence of diabetes
0.0
0.1
0.2
0.3
0.4
0.5
1980
1985
1990
1995
2000
2005
2010
2015

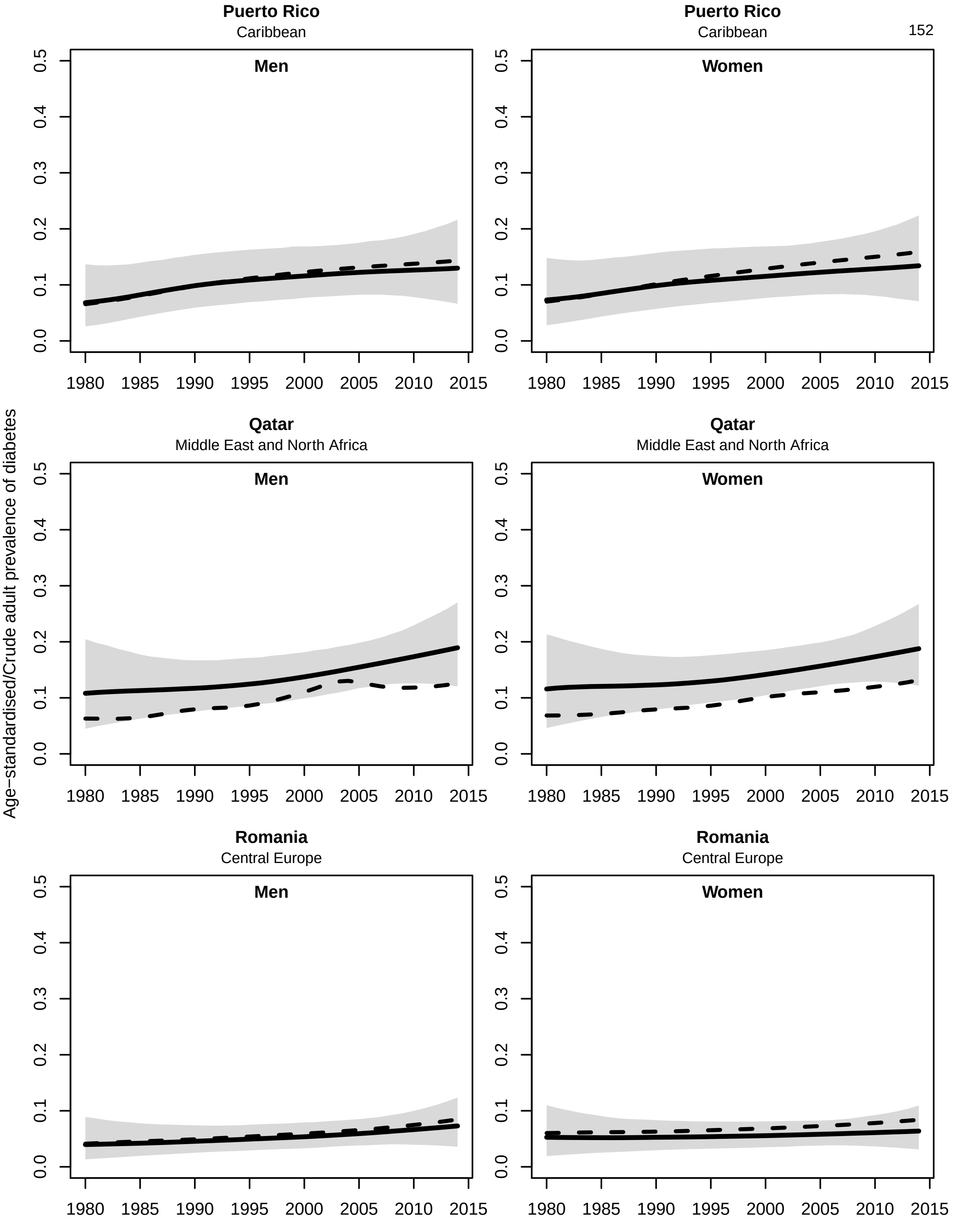
Puerto Rico
Caribbean
Men
Puerto Rico
Caribbean
Women
Qatar
Middle East and North Africa
Men
Qatar
Middle East and North Africa
Women
Romania
Central Europe
Men
Romania
Central Europe
Women
Age−standardised/Crude adult prevalence of diabetes
0.0
0.1
0.2
0.3
0.4
0.5
1980
1985
1990
1995
2000
2005
2010
2015

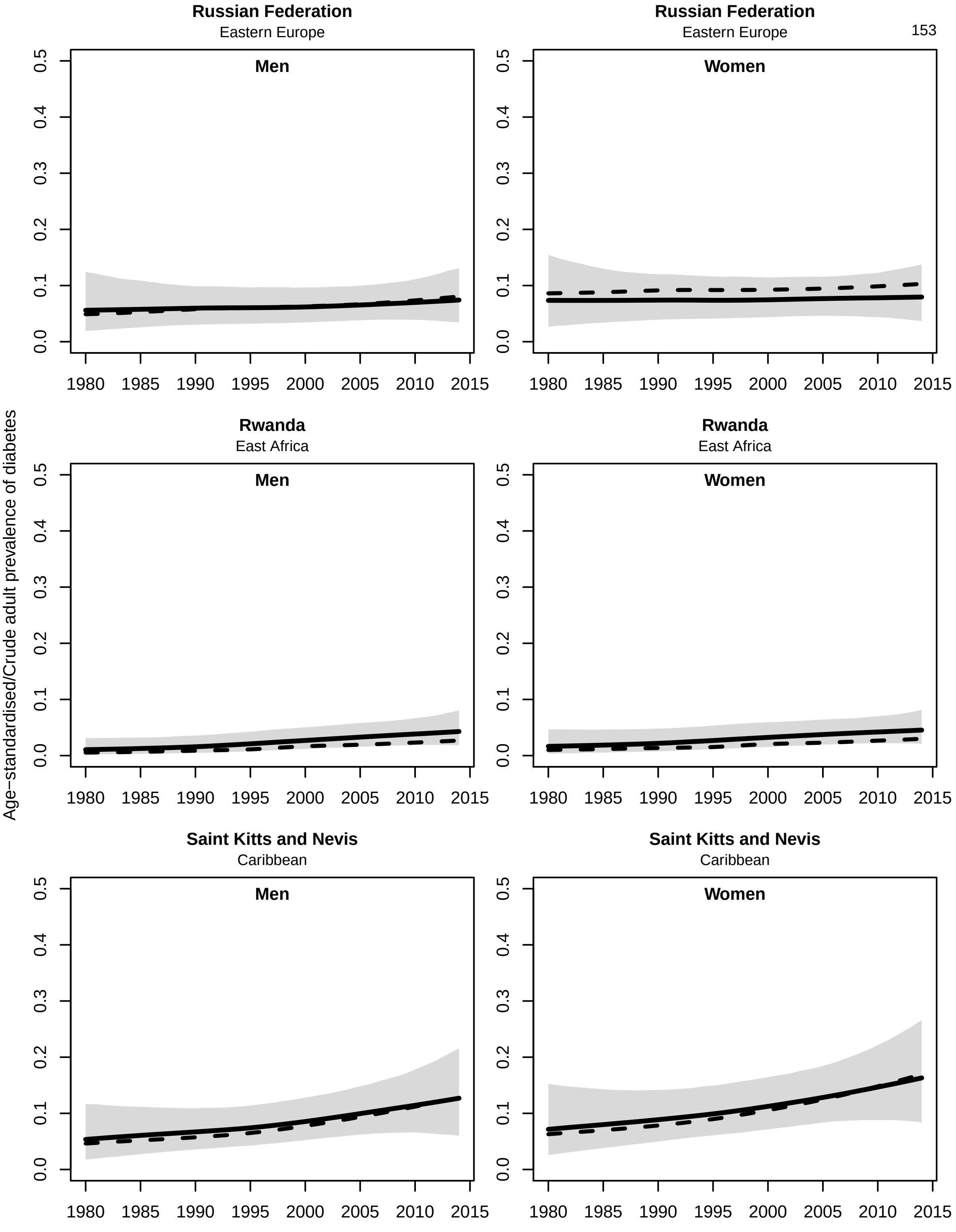
Russian Federation
Eastern Europe
Men
Russian Federation
Eastern Europe
Women
Rwanda
East Africa
Men
Rwanda
East Africa
Women
Saint Kitts and Nevis
Caribbean
Men
Saint Kitts and Nevis
Caribbean
Women
Age−standardised/Crude adult prevalence of diabetes
0.0
0.1
0.2
0.3
0.4
0.5
1980
1985
1990
1995
2000
2005
2010
2015

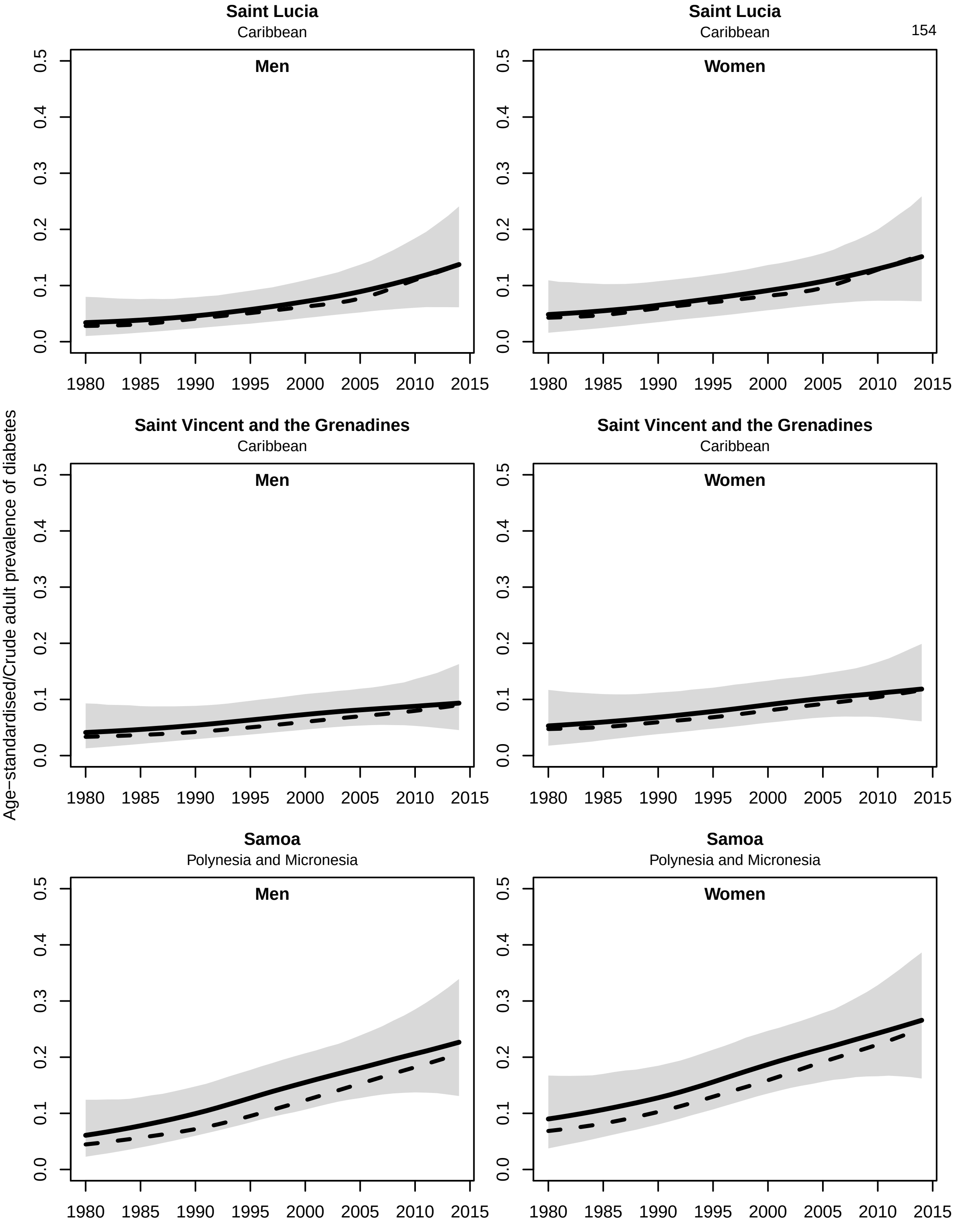
Saint Lucia
Caribbean
Men
Saint Lucia
Caribbean
Women
Saint Vincent and the Grenadines
Caribbean
Men
Saint Vincent and the Grenadines
Caribbean
Women
Samoa
Polynesia and Micronesia
Men
Samoa
Polynesia and Micronesia
Women
Age−standardised/Crude adult prevalence of diabetes
0.0
0.1
0.2
0.3
0.4
0.5
1980
1985
1990
1995
2000
2005
2010
2015

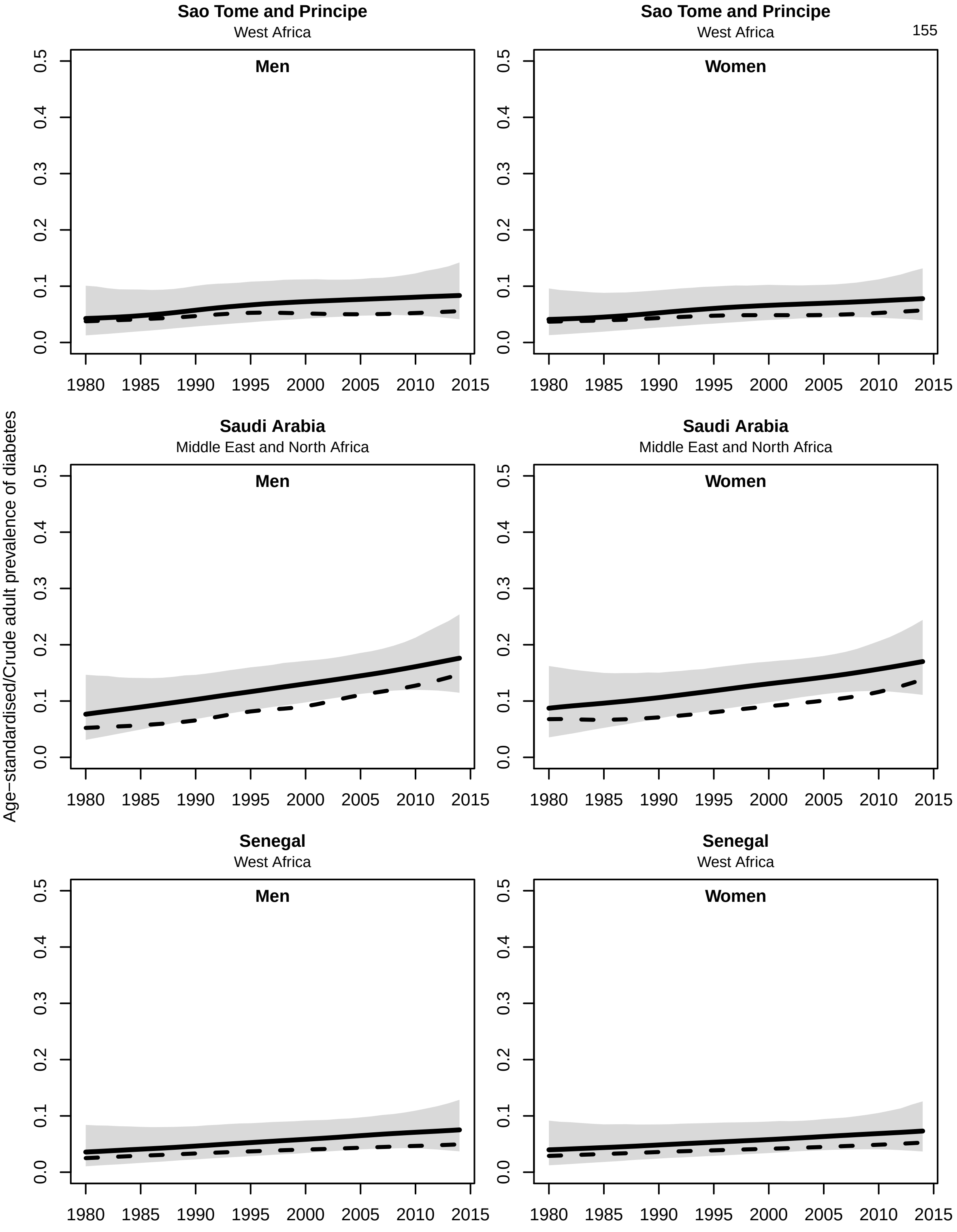
Sao Tome and Principe
West Africa
Men
Women
Saudi Arabia
Middle East and North Africa
Men
Women
Senegal
West Africa
Men
Women
Age−standardised/Crude adult prevalence of diabetes
0.0
0.1
0.2
0.3
0.4
0.5
1980
1985
1990
1995
2000
2005
2010
2015

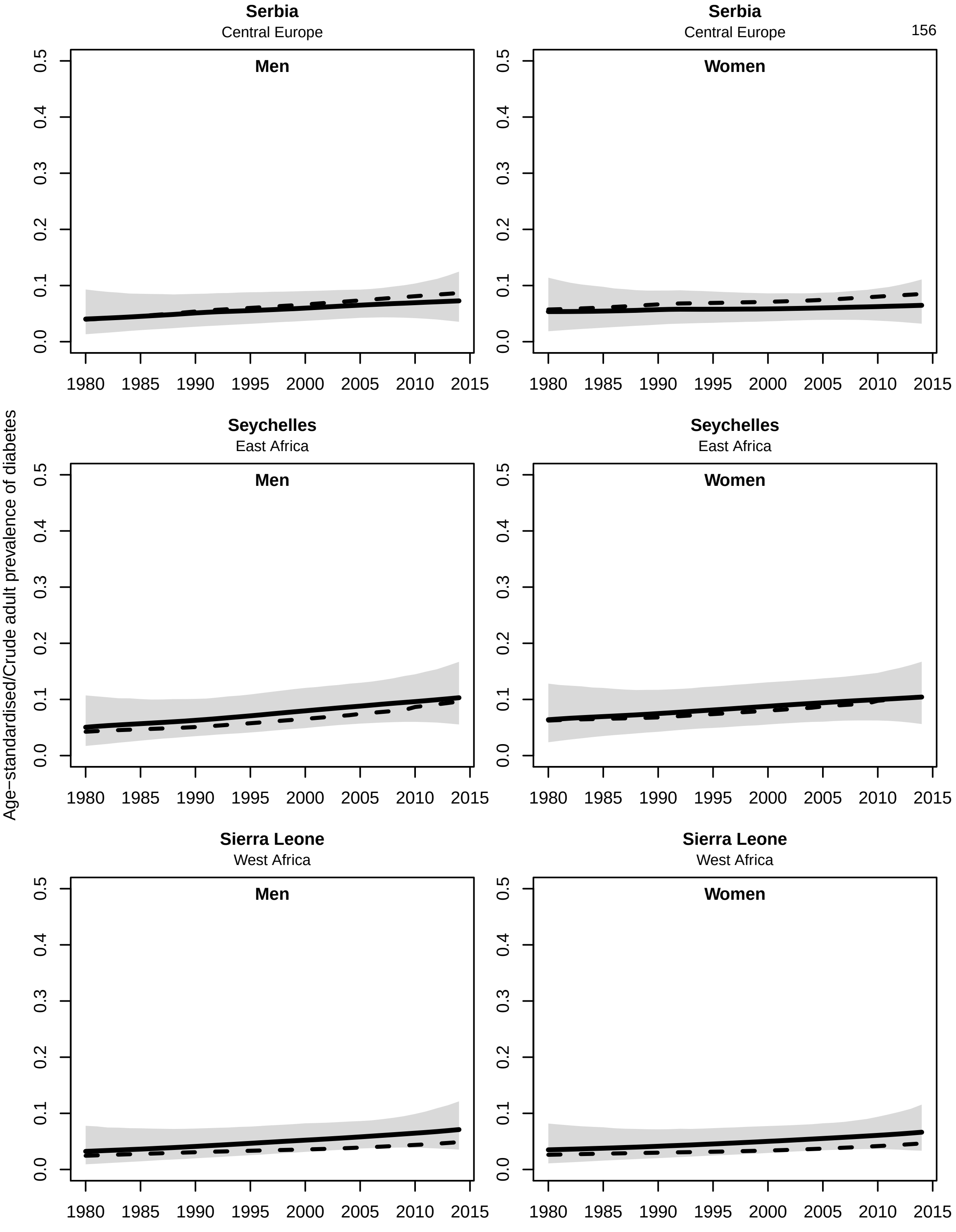
Serbia
Central Europe
Men
Serbia
Central Europe
Women
Seychelles
East Africa
Men
Seychelles
East Africa
Women
Sierra Leone
West Africa
Men
Sierra Leone
West Africa
Women
Age−standardised/Crude adult prevalence of diabetes
0.0
0.1
0.2
0.3
0.4
0.5
1980
1985
1990
1995
2000
2005
2010
2015

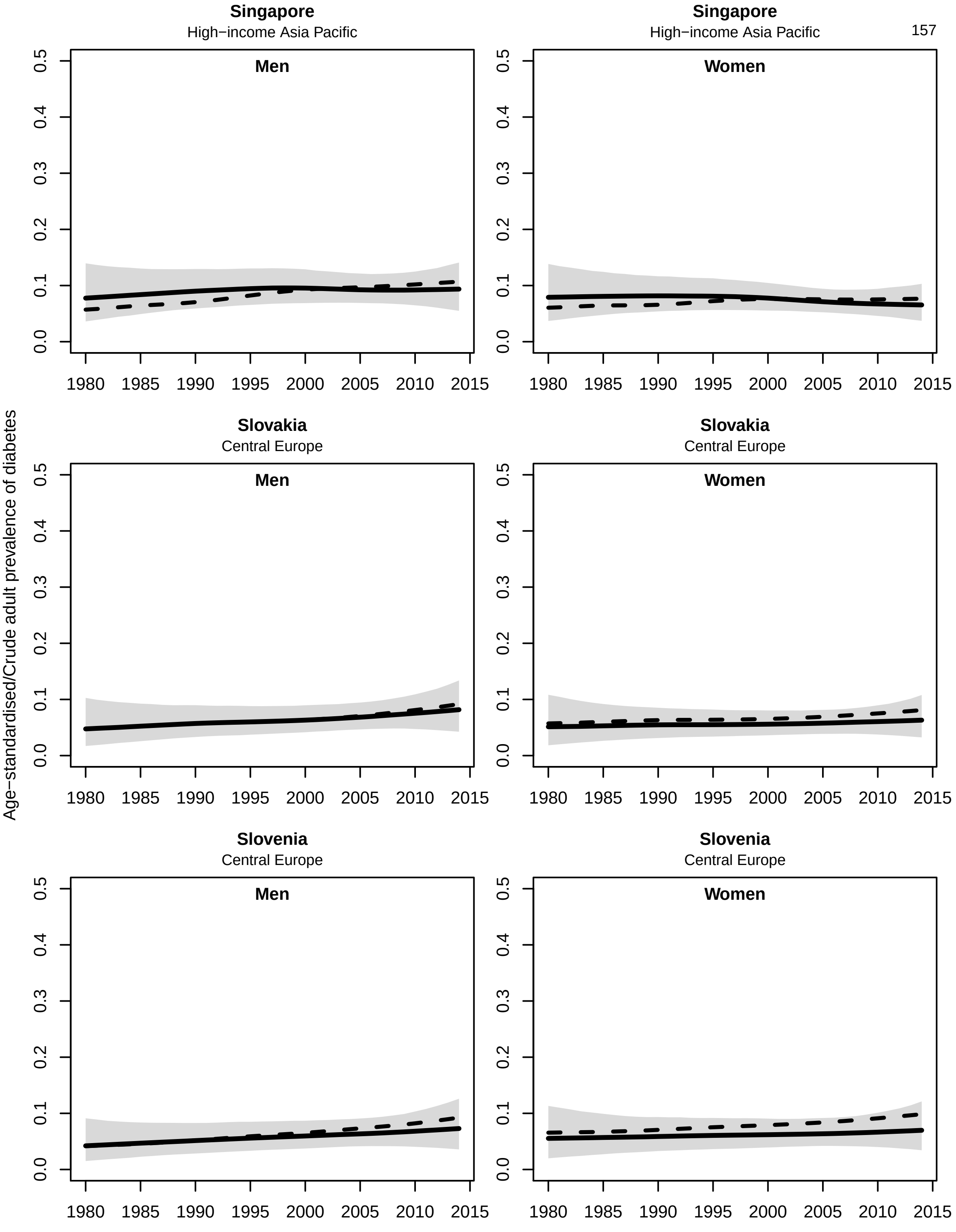
Singapore
High−income Asia Pacific
Men
Singapore
High−income Asia Pacific
Women
Slovakia
Central Europe
Men
Slovakia
Central Europe
Women
Slovenia
Central Europe
Men
Slovenia
Central Europe
Women
Age−standardised/Crude adult prevalence of diabetes
0.0
0.1
0.2
0.3
0.4
0.5
1980
1985
1990
1995
2000
2005
2010
2015

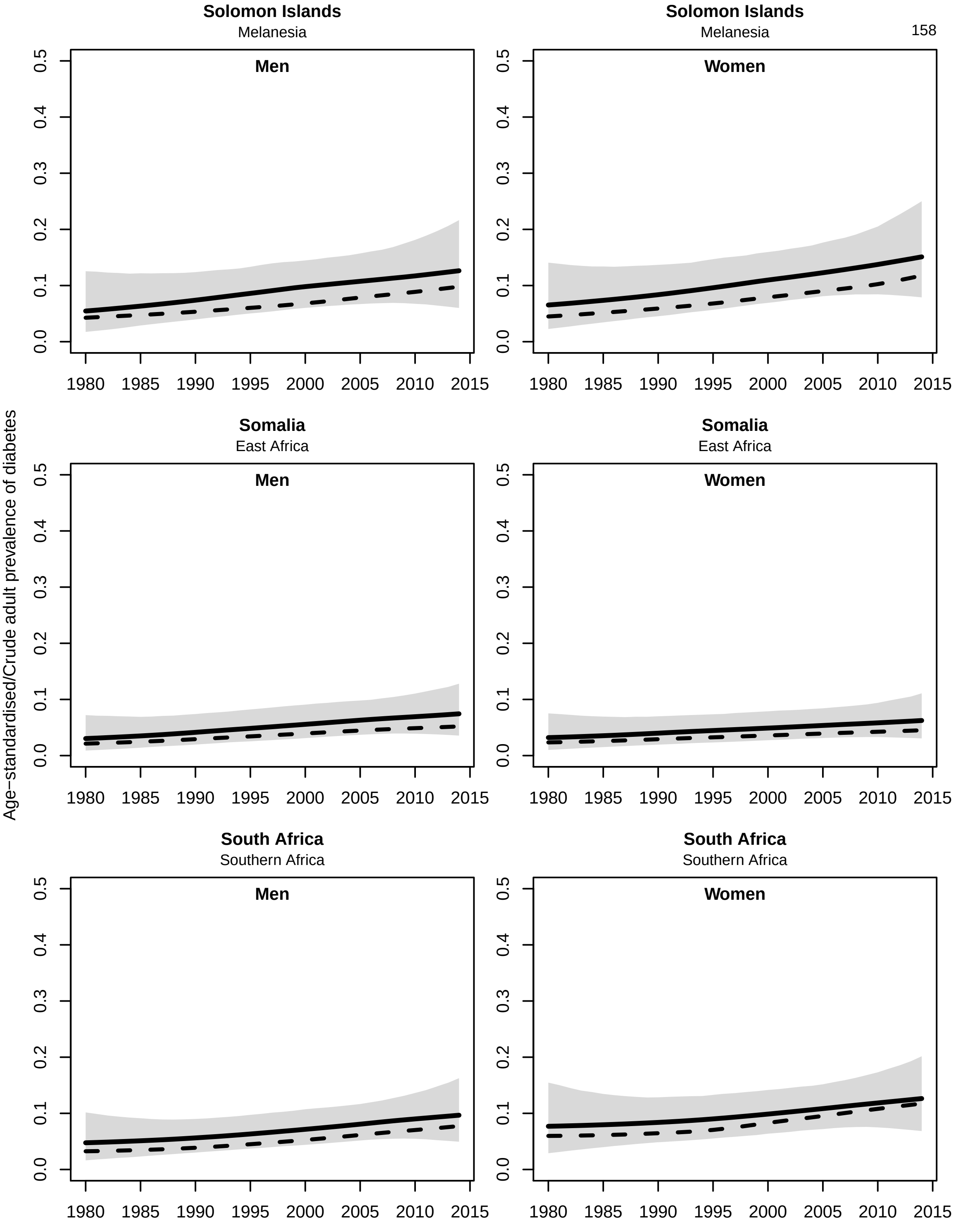
Solomon Islands
Melanesia
Men
Solomon Islands
Melanesia
Women
Somalia
East Africa
Men
Somalia
East Africa
Women
South Africa
Southern Africa
Men
South Africa
Southern Africa
Women
Age−standardised/Crude adult prevalence of diabetes
0.0
0.1
0.2
0.3
0.4
0.5
1980
1985
1990
1995
2000
2005
2010
2015

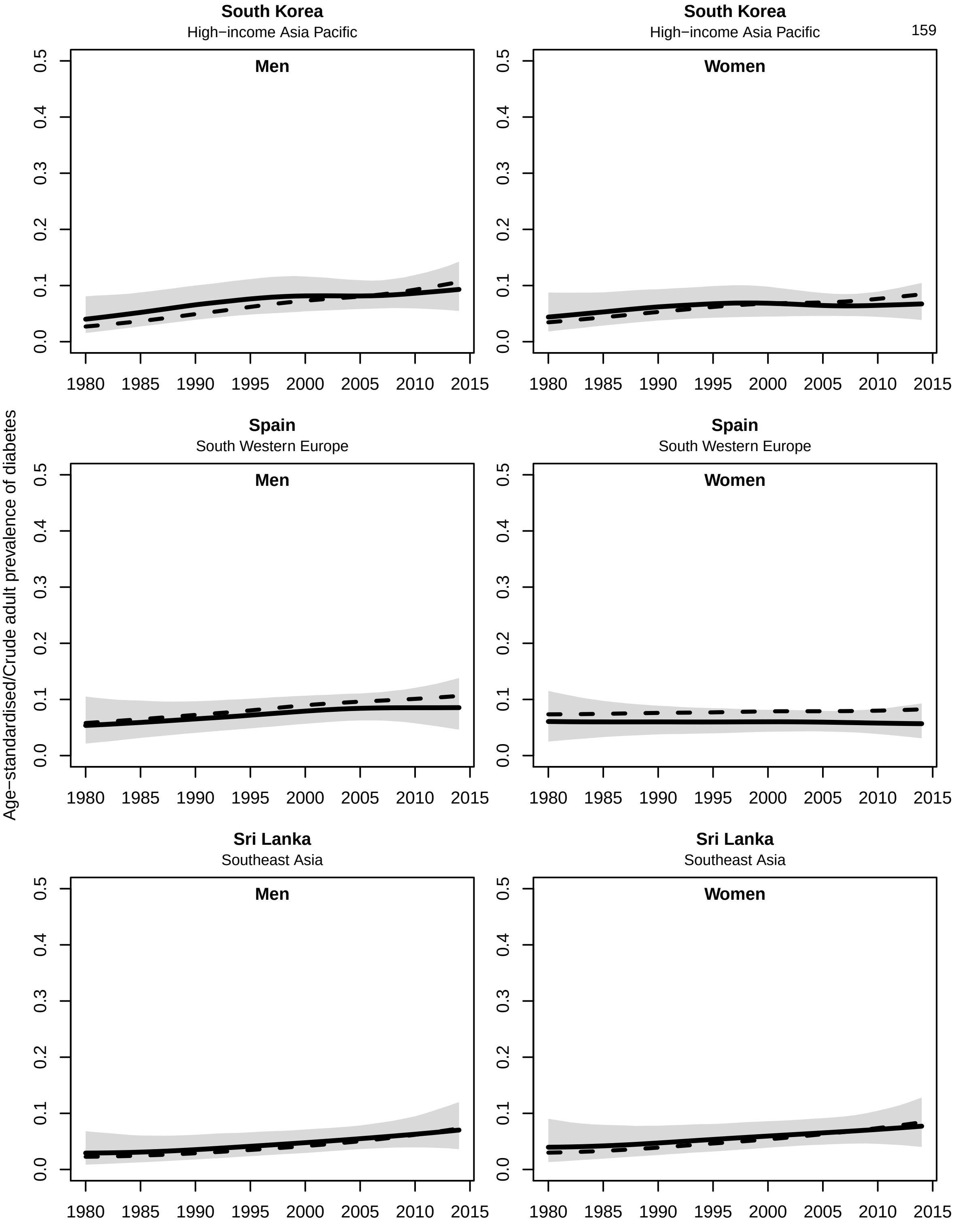
South Korea
High−income Asia Pacific
Men
South Korea
High−income Asia Pacific
Women
Spain
South Western Europe
Men
Spain
South Western Europe
Women
Sri Lanka
Southeast Asia
Men
Sri Lanka
Southeast Asia
Women
Age−standardised/Crude adult prevalence of diabetes
0.0
0.1
0.2
0.3
0.4
0.5
1980
1985
1990
1995
2000
2005
2010
2015

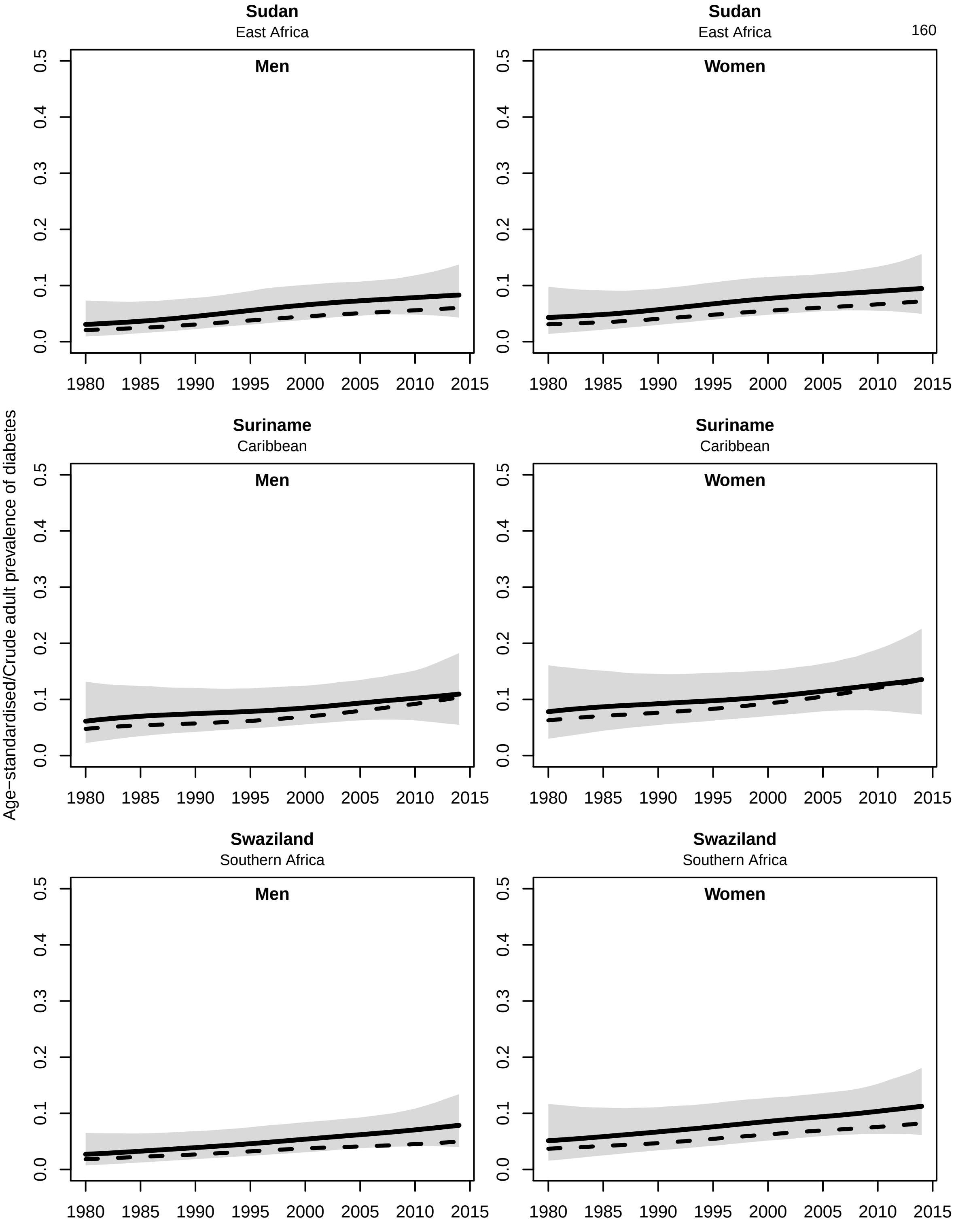
Sudan
East Africa
Men
Sudan
East Africa
Women
Suriname
Caribbean
Men
Suriname
Caribbean
Women
Swaziland
Southern Africa
Men
Swaziland
Southern Africa
Women
Age−standardised/Crude adult prevalence of diabetes
0.0
0.1
0.2
0.3
0.4
0.5
1980
1985
1990
1995
2000
2005
2010
2015

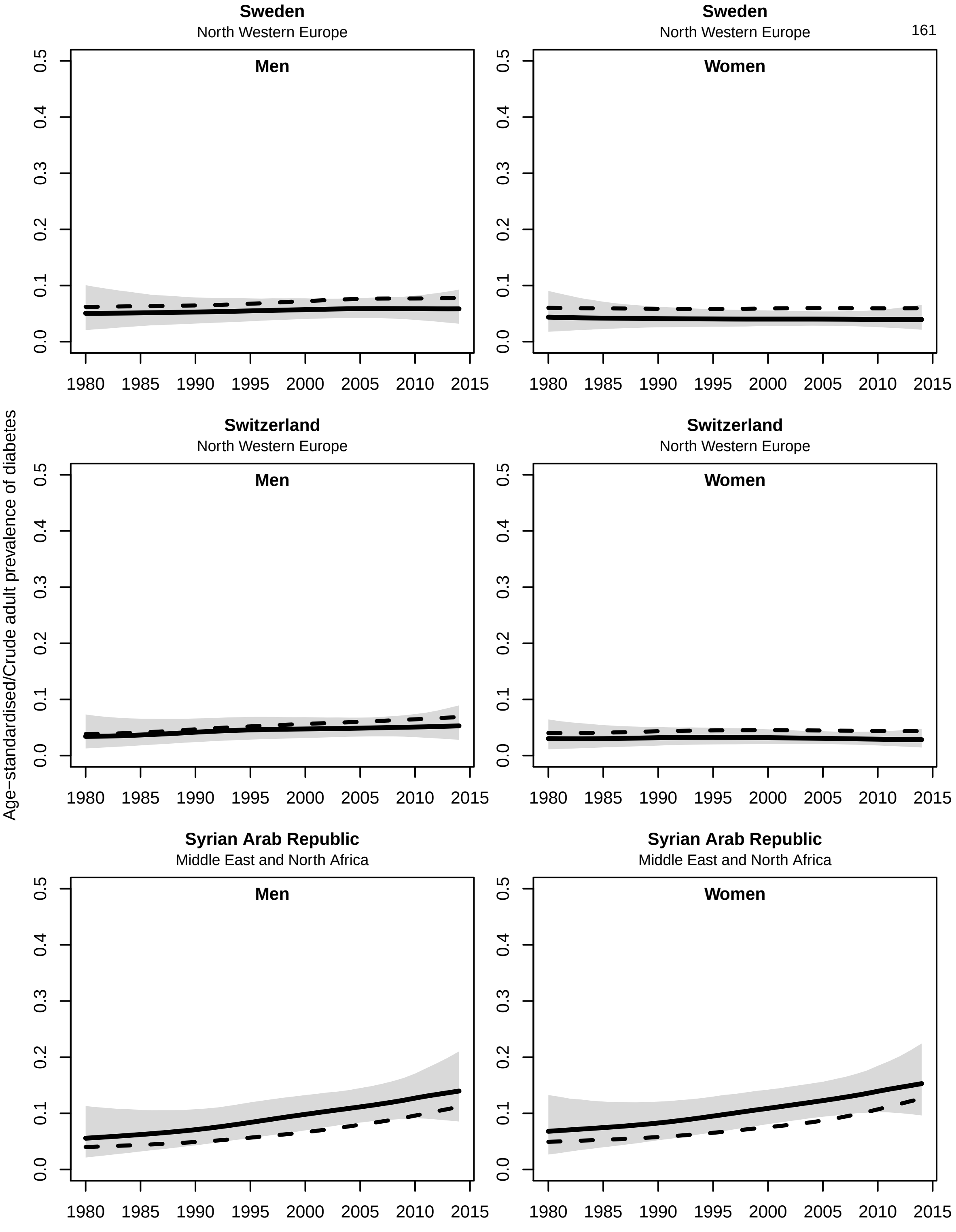
Sweden
North Western Europe
Men
Sweden
North Western Europe
Women
Switzerland
North Western Europe
Men
Switzerland
North Western Europe
Women
Syrian Arab Republic
Middle East and North Africa
Men
Syrian Arab Republic
Middle East and North Africa
Women
Age−standardised/Crude adult prevalence of diabetes
0.0
0.1
0.2
0.3
0.4
0.5
1980
1985
1990
1995
2000
2005
2010
2015

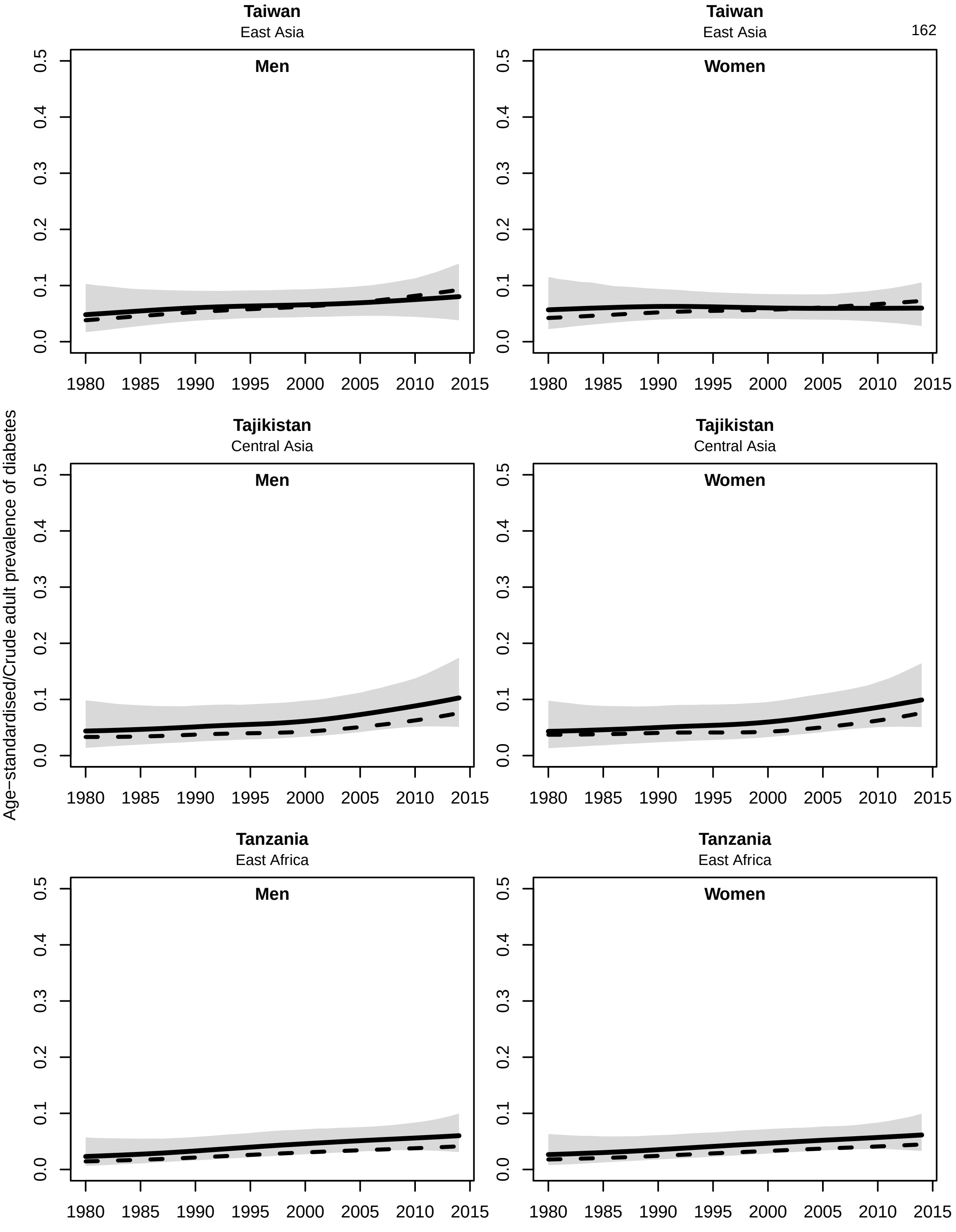
Taiwan
East Asia
Men
Taiwan
East Asia
Women
Tajikistan
Central Asia
Men
Tajikistan
Central Asia
Women
Tanzania
East Africa
Men
Tanzania
East Africa
Women
Age−standardised/Crude adult prevalence of diabetes
0.0
0.1
0.2
0.3
0.4
0.5
1980
1985
1990
1995
2000
2005
2010
2015

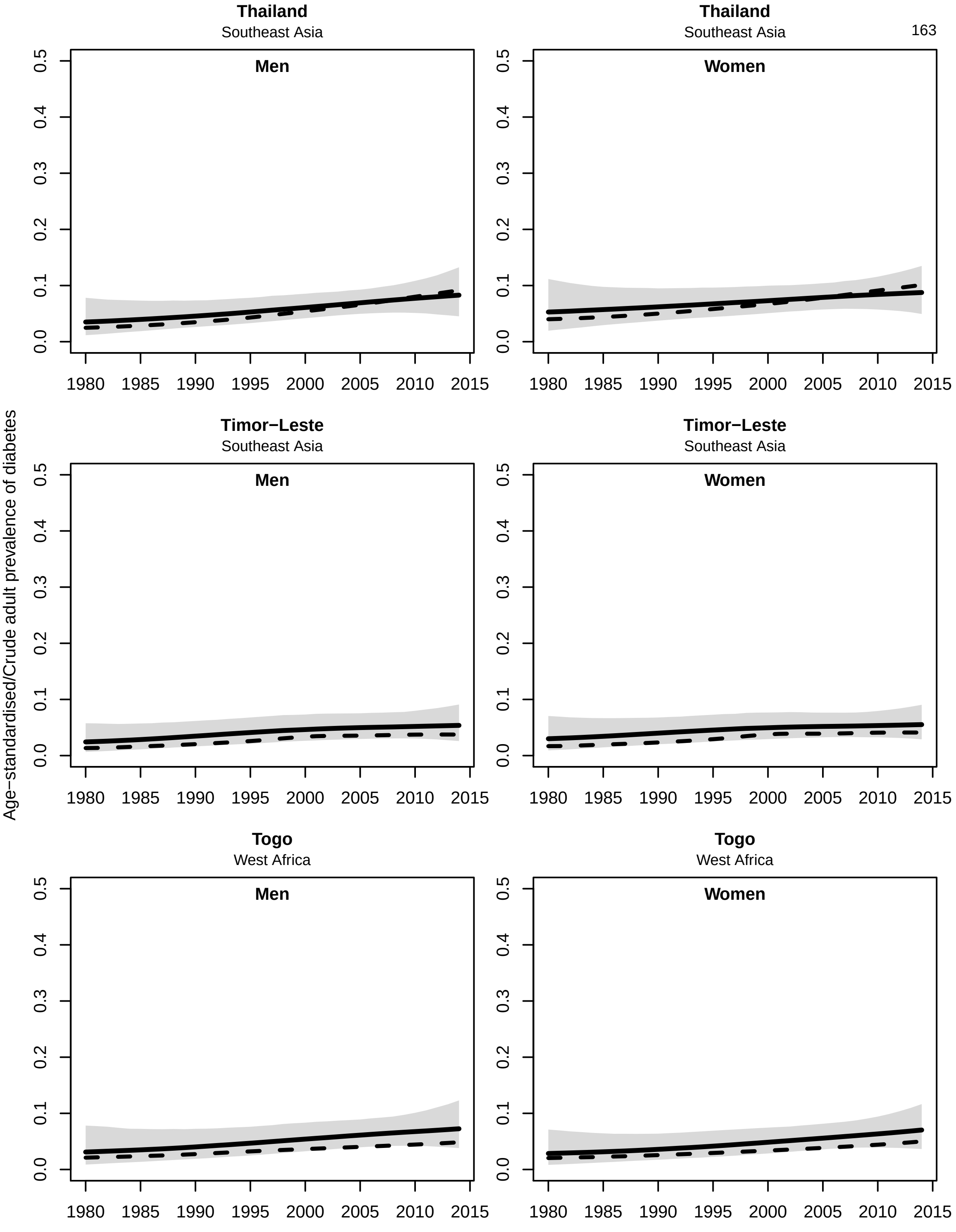
Thailand
Southeast Asia
Men
Thailand
Southeast Asia
Women
Timor−Leste
Southeast Asia
Men
Timor−Leste
Southeast Asia
Women
Togo
West Africa
Men
Togo
West Africa
Women
Age−standardised/Crude adult prevalence of diabetes
0.0
0.1
0.2
0.3
0.4
0.5
1980
1985
1990
1995
2000
2005
2010
2015

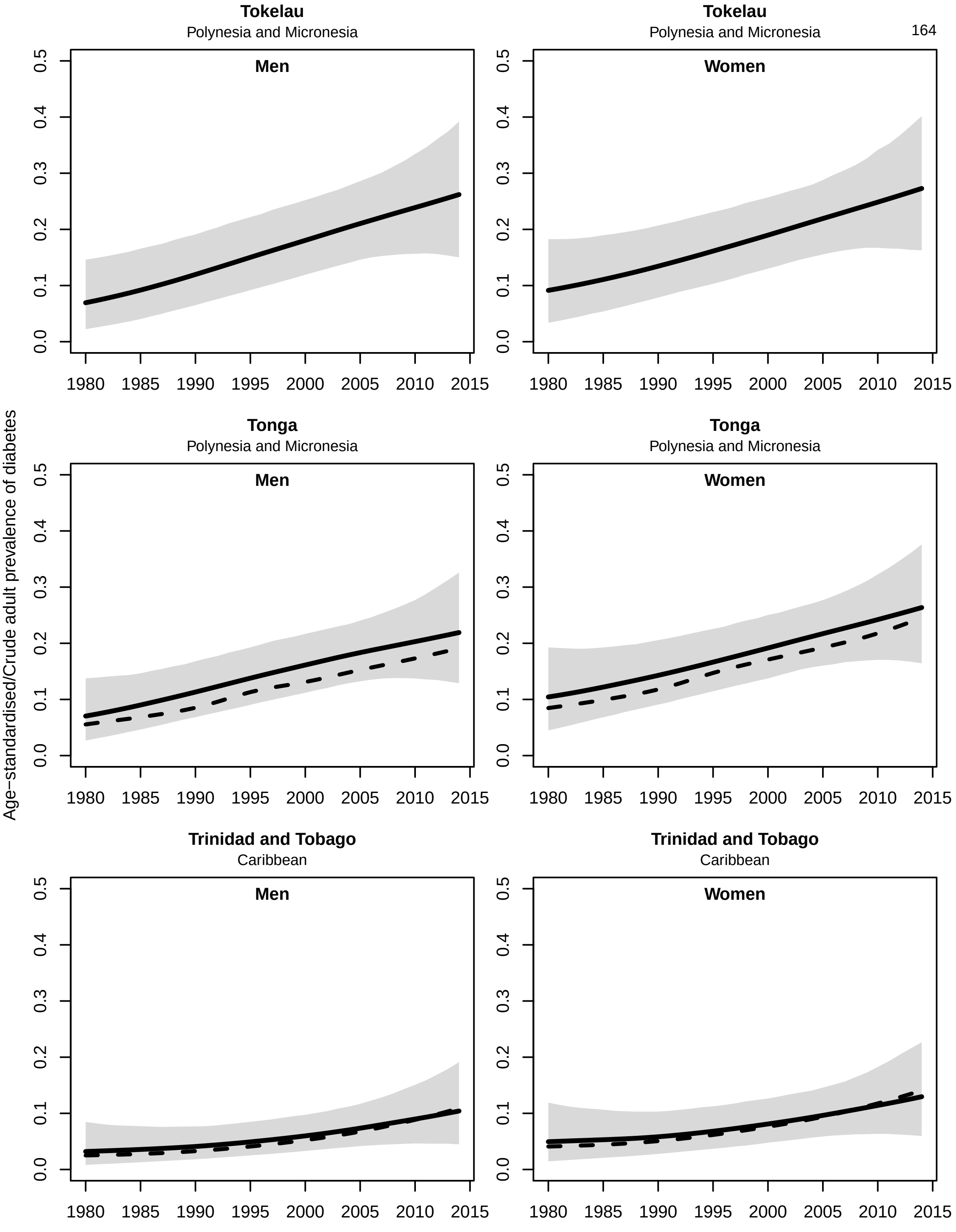
Tokelau
Polynesia and Micronesia
Men
Women
Tonga
Polynesia and Micronesia
Men
Women
Trinidad and Tobago
Caribbean
Men
Women
Age−standardised/Crude adult prevalence of diabetes
0.0
0.1
0.2
0.3
0.4
0.5
1980
1985
1990
1995
2000
2005
2010
2015

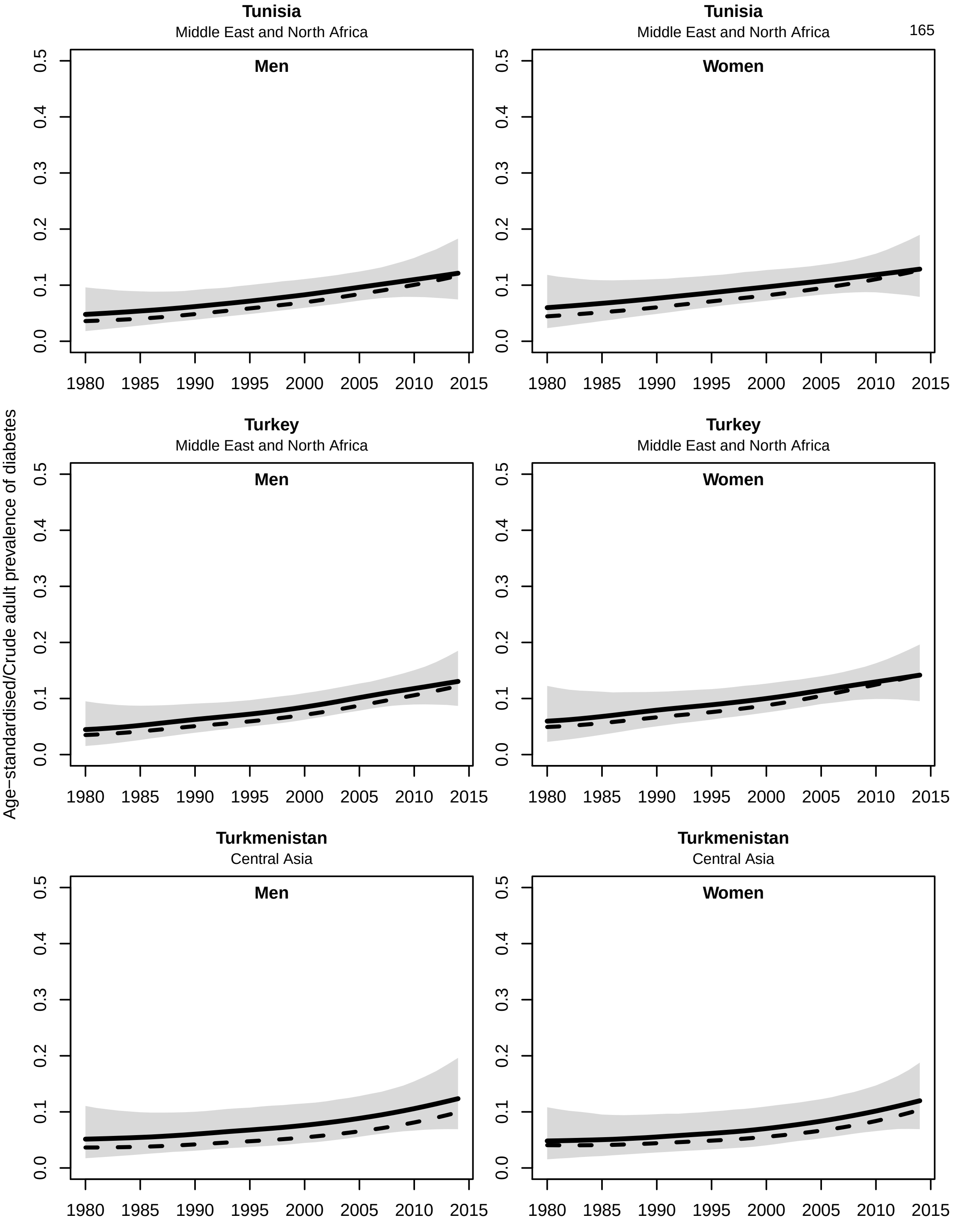
Tunisia
Middle East and North Africa
Men
Tunisia
Middle East and North Africa
Women
Turkey
Middle East and North Africa
Men
Turkey
Middle East and North Africa
Women
Turkmenistan
Central Asia
Men
Turkmenistan
Central Asia
Women
Age−standardised/Crude adult prevalence of diabetes
0.0
0.1
0.2
0.3
0.4
0.5
1980
1985
1990
1995
2000
2005
2010
2015

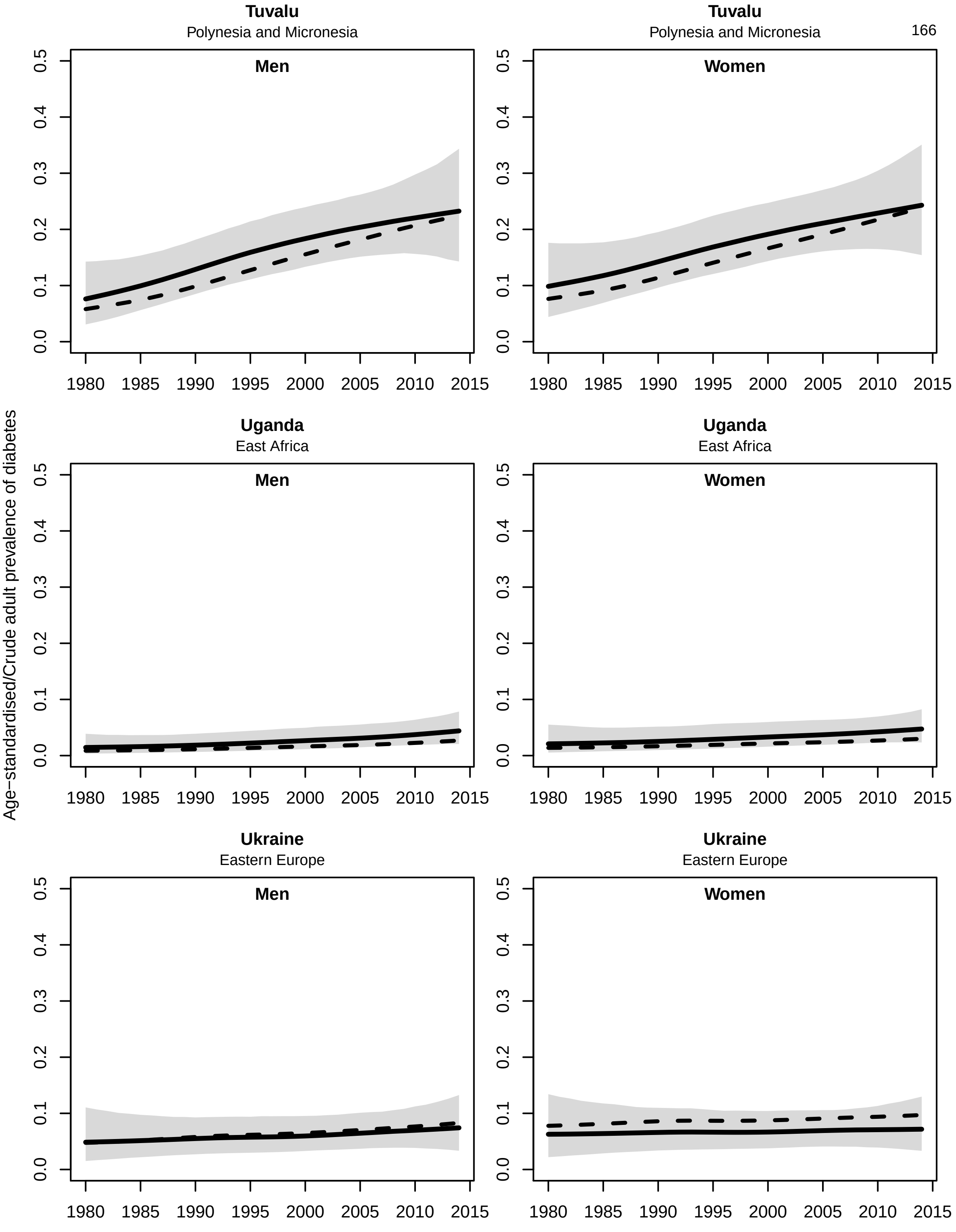

Tuvalu
Polynesia and Micronesia
Men
Women
Uganda
East Africa
Ukraine
Eastern Europe
Age−standardised/Crude adult prevalence of diabetes
0.0
0.1
0.2
0.3
0.4
0.5
1980
1985
1990
1995
2000
2005
2010
2015

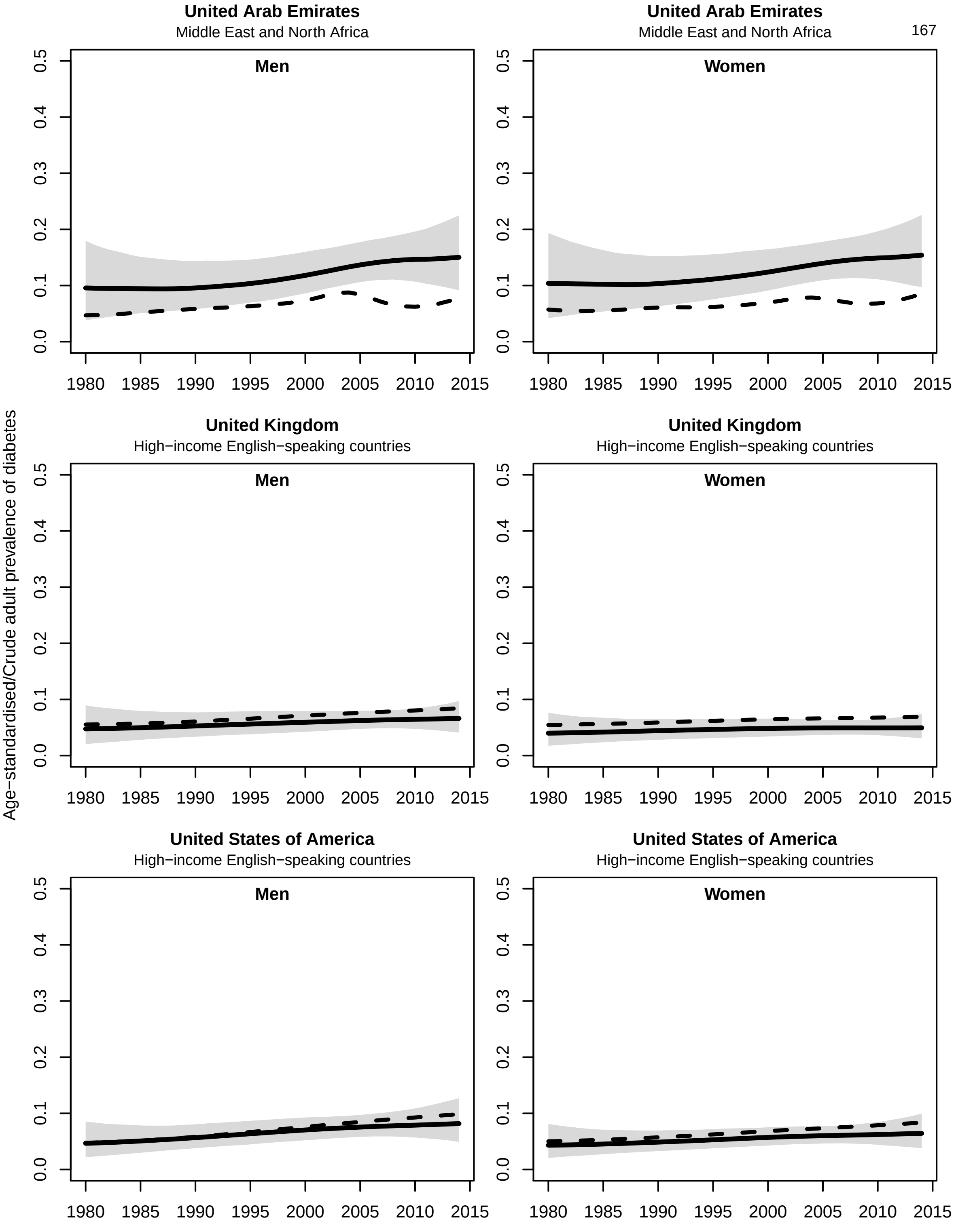
United Arab Emirates
Middle East and North Africa
Men
Women
United Kingdom
High−income English−speaking countries
United States of America
Age−standardised/Crude adult prevalence of diabetes
0.0
0.1
0.2
0.3
0.4
0.5
1980
1985
1990
1995
2000
2005
2010
2015

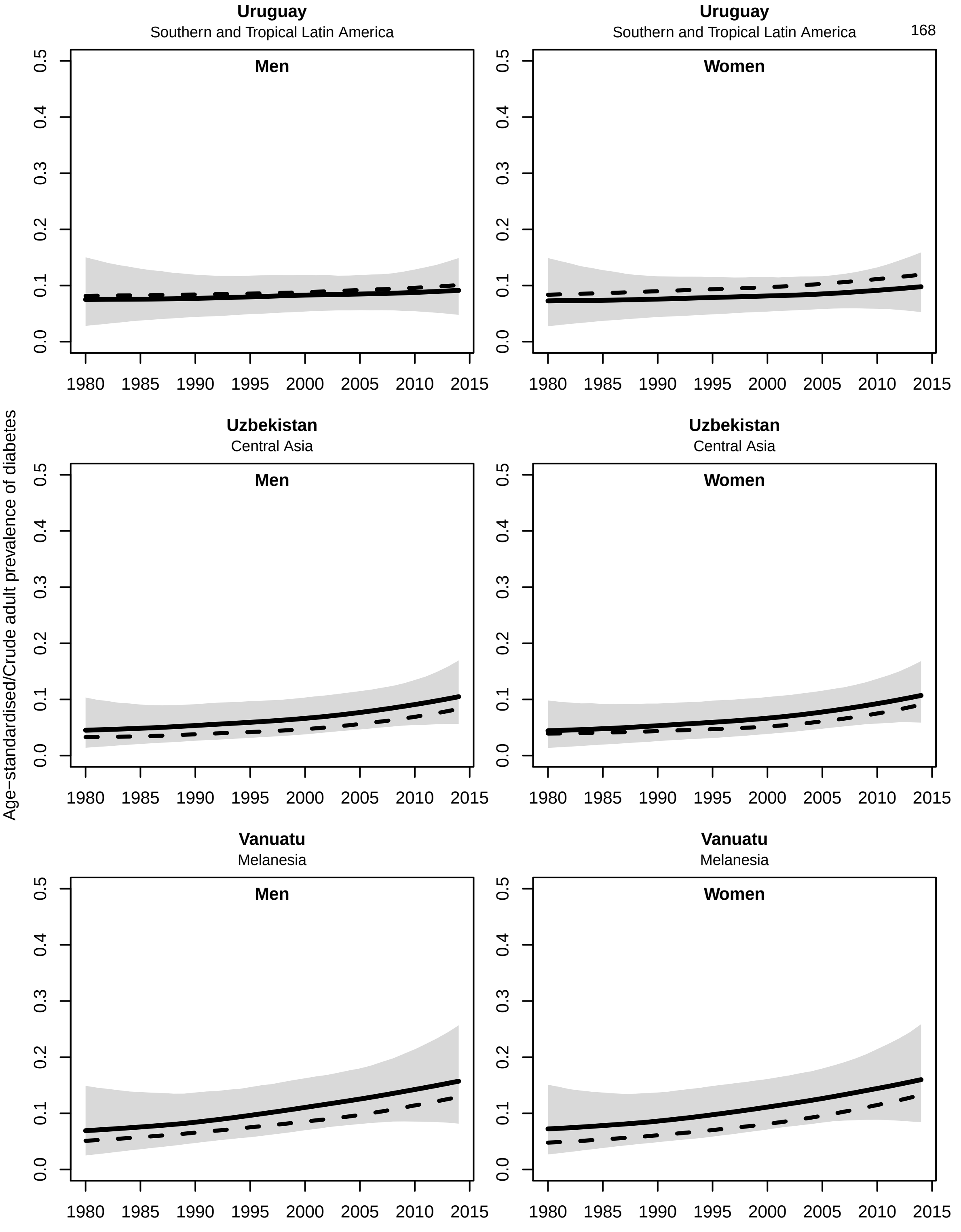
Uruguay
Southern and Tropical Latin America
Men
Uruguay
Southern and Tropical Latin America
Women
Uzbekistan
Central Asia
Men
Uzbekistan
Central Asia
Women
Vanuatu
Melanesia
Men
Vanuatu
Melanesia
Women
Age−standardised/Crude adult prevalence of diabetes
0.0
0.1
0.2
0.3
0.4
0.5
1980
1985
1990
1995
2000
2005
2010
2015

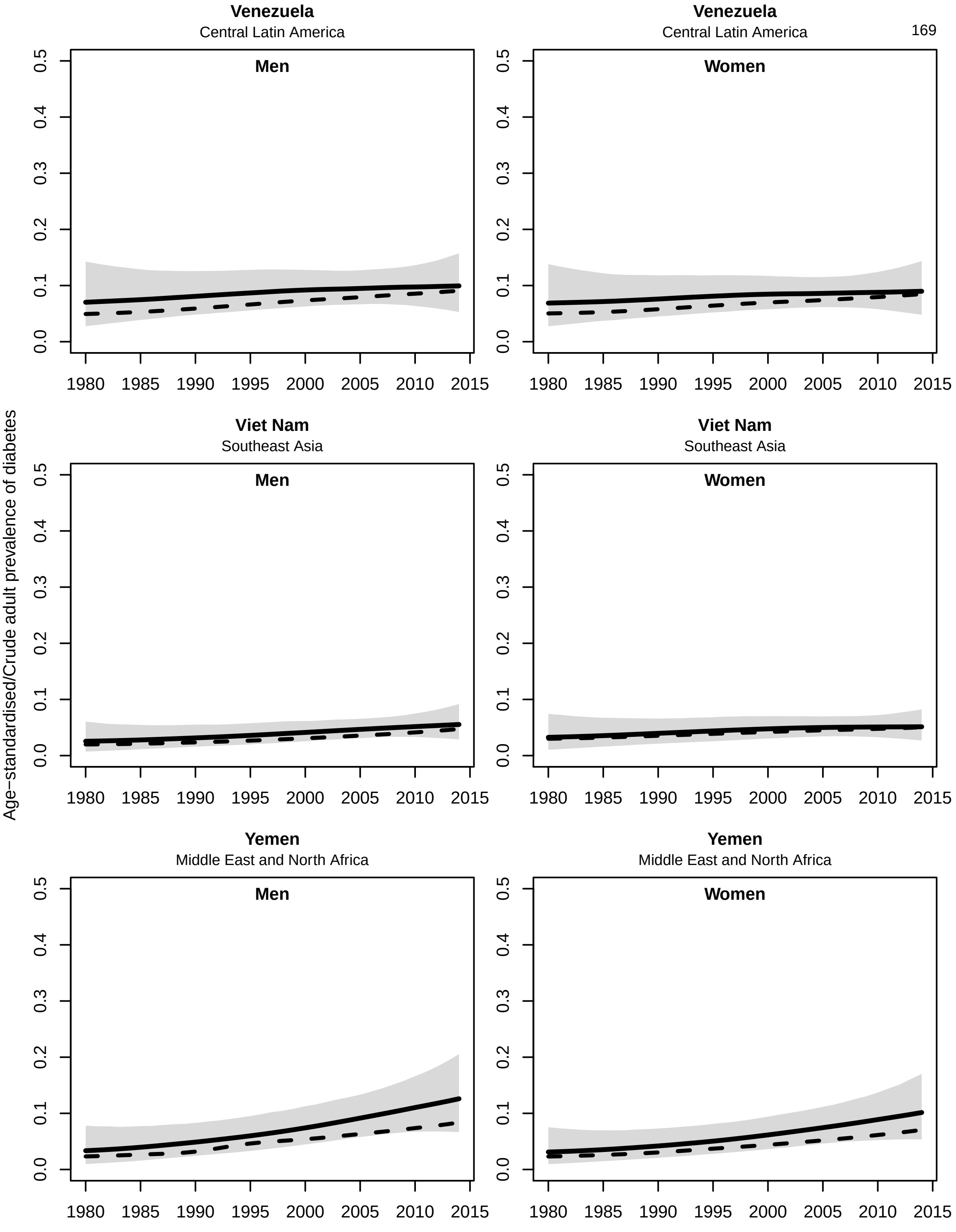
Venezuela
Central Latin America
Men
Venezuela
Central Latin America
Women
Viet Nam
Southeast Asia
Men
Viet Nam
Southeast Asia
Women
Yemen
Middle East and North Africa
Men
Yemen
Middle East and North Africa
Women
Age−standardised/Crude adult prevalence of diabetes
0.0
0.1
0.2
0.3
0.4
0.5
1980
1985
1990
1995
2000
2005
2010
2015

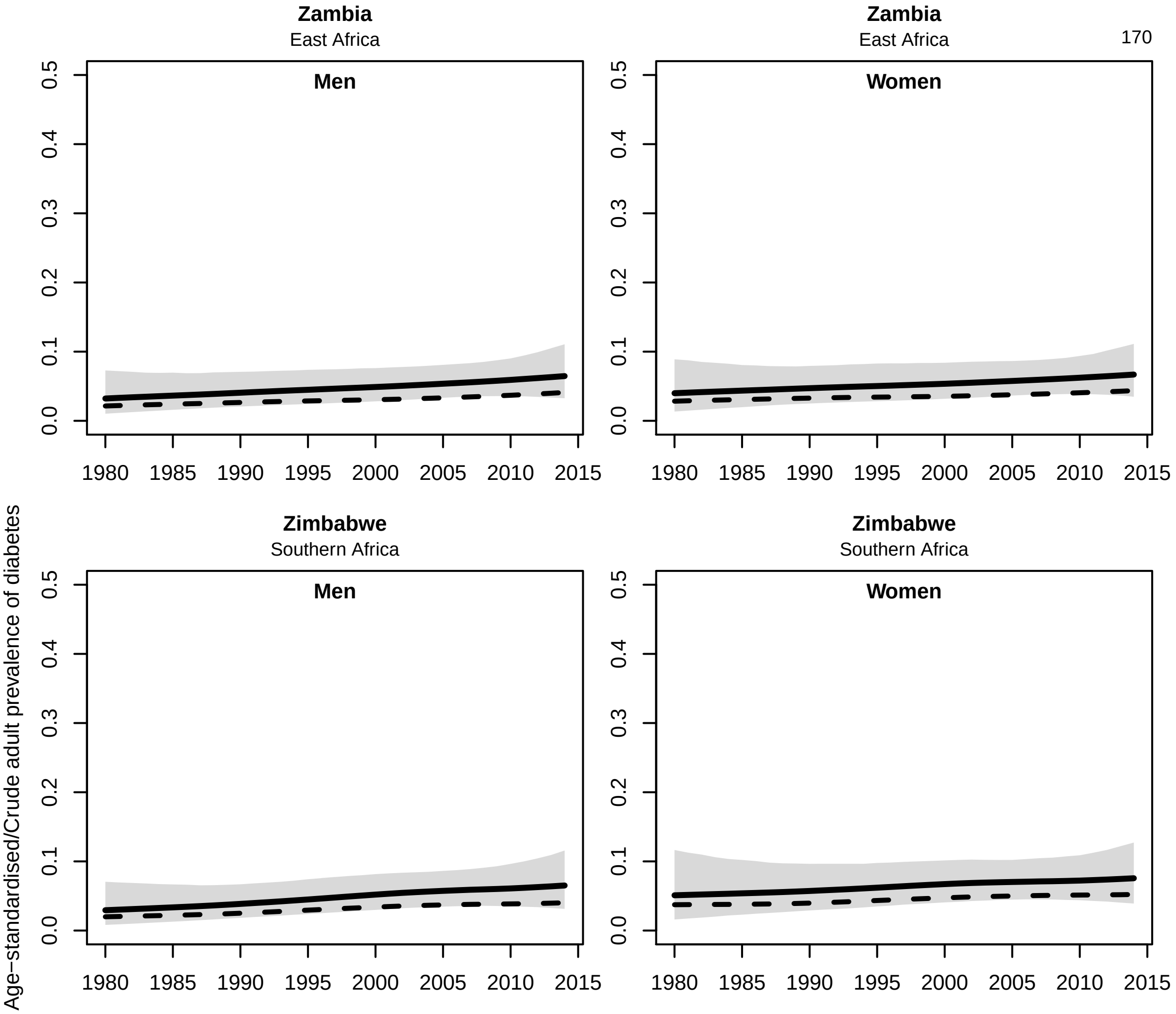
Zambia
East Africa
Men
Zambia
East Africa
Women
Zimbabwe
Southern Africa
Men
Zimbabwe
Southern Africa
Women
Age−standardised/Crude adult prevalence of diabetes
0.0
0.1
0.2
0.3
0.4
0.5
1980
1985
1990
1995
2000
2005
2010
2015

References

1. Danaei G, Finucane MM, Lu Y, et al. National, regional, and global trends in fasting plasma glucose and diabetes prevalence since 1980: systematic analysis of health examination surveys and epidemiological studies with 370 country-years and 2.7 million participants. *Lancet* 2011; **378**(9785): 31-40.
2. Schwarz G. Estimating Dimension of a Model. *Ann Stat* 1978; **6**(2): 461-4.
3. Sacks DB, Arnold M, Bakris GL, et al. Guidelines and recommendations for laboratory analysis in the diagnosis and management of diabetes mellitus. *Clin Chem* 2011; **57**(6): e1-e47.
4. McFadden D. Conditional Logit Analysis of Qualitative Choice Behavior. In: Zarembka P, ed. Frontiers in econometrics: Academic Press; 1974: 105-42.
